# Preferences for HIV testing services among men who have sex with men in the UK: A discrete choice experiment

**DOI:** 10.1371/journal.pmed.1002779

**Published:** 2019-04-11

**Authors:** Alec Miners, Tom Nadarzynski, Charles Witzel, Andrew N. Phillips, Valentina Cambiano, Alison J. Rodger, Carrie D. Llewellyn

**Affiliations:** 1 Faculty of Public Health and Policy, London School of Hygiene & Tropical Medicine, London, United Kingdom; 2 Department of Psychology, University of Southampton, Southampton, United Kingdom; 3 Royal South Hants Hospital, Solent NHS Trust, Southampton, United Kingdom; 4 Institute for Global Health, University College London, London, United Kingdom; 5 Department of Primary Care and Public Health, Brighton and Sussex Medical School, Brighton, United Kingdom; University of California, San Francisco, UNITED STATES

## Abstract

**Background:**

In the UK, approximately 4,200 men who have sex with men (MSM) are living with HIV but remain undiagnosed. Maximising the number of high-risk people testing for HIV is key to ensuring prompt treatment and preventing onward infection. This study assessed how different HIV test characteristics affect the choice of testing option, including remote testing (HIV self-testing or HIV self-sampling), in the UK, a country with universal access to healthcare.

**Methods and findings:**

Between 3 April and 11 May 2017, a cross-sectional online-questionnaire-based discrete choice experiment (DCE) was conducted in which respondents who expressed an interest in online material used by MSM were asked to imagine that they were at risk of HIV infection and to choose between different hypothetical HIV testing options, including the option not to test. A variety of different testing options with different defining characteristics were described so that the independent preference for each characteristic could be valued. The characteristics included where each test is taken, the sampling method, how the test is obtained, whether infections other than HIV are tested for, test accuracy, the cost of the test, the infection window period, and how long it takes to receive the test result. Participants were recruited and completed the instrument online, in order to include those not currently engaged with healthcare services. The main analysis was conducted using a latent class model (LCM), with results displayed as odds ratios (ORs) and probabilities. The ORs indicate the strength of preference for one characteristic relative to another (base) characteristic. In total, 620 respondents answered the DCE questions. Most respondents reported that they were white (93%) and were either gay or bisexual (99%). The LCM showed that there were 2 classes within the respondent sample that appeared to have different preferences for the testing options. The first group, which was likely to contain 86% of respondents, had a strong preference for face-to-face tests by healthcare professionals (HCPs) compared to remote testing (OR 6.4; 95% CI 5.6, 7.4) and viewed not testing as less preferable than remote testing (OR 0.10; 95% CI 0.09, 0.11). In the second group, which was likely to include 14% of participants, not testing was viewed as less desirable than remote testing (OR 0.56; 95% CI 0.53, 0.59) as were tests by HCPs compared to remote testing (OR 0.23; 95% CI 0.15, 0.36). In both classes, free remote tests instead of each test costing £30 was the test characteristic with the largest impact on the choice of testing option. Participants in the second group were more likely to have never previously tested and to be non-white than participants in the first group. The main study limitations were that the sample was recruited solely via social media, the study advert was viewed only by people expressing an interest in online material used by MSM, and the choices in the experiment were hypothetical rather than observed in the real world.

**Conclusions:**

Our results suggest that preferences in the context we examined are broadly dichotomous. One group, containing the majority of MSM, appears comfortable testing for HIV but prefers face-to-face testing by HCPs rather than remote testing. The other group is much smaller, but contains MSM who are more likely to be at high infection risk. For these people, the availability of remote testing has the potential to significantly increase net testing rates, particularly if provided for free.

## Abstract 2

In a discrete choice experiment, Carrie Llewellyn and co-workers investigate the preferences of men who have sex with men for provision of HIV testing.

## Author summary

**Why was this study done?:**

- HIV testing remains low in high-risk populations, such as men who have sex with men (MSM).
- HIV tests can be performed by healthcare professionals (HCPs) or remotely, using self-testing and self-sampling kits.
- However, the relative importance of the test characteristics most likely to optimise their use—such as the time it takes to receive a result and the cost of the tests—is not fully understood.

**What did the authors do and find?:**

- We assessed the strength of preference for different HIV test characteristics by asking men in the UK who expressed an interest in online material used by MSM to complete an online questionnaire.
- Participants were recruited using social media.
- Most respondents preferred to be tested by HCPs rather than to test remotely.
- The most important barrier to remote testing was its cost.

**What do these findings mean?:**

- In this study, HIV testing carried out by HCPs was found to be particularly valued by respondents.
- There was some evidence that remote testing might increase testing rates, albeit for a minority of respondents.
- Remote HIV testing should generally be viewed as supplementary to HCP-delivered services.

## Introduction

Although data show that there has been a steep decline in the incidence of HIV in some areas of the world due to combination prevention [1], recent European surveillance data show that the overall number of new HIV diagnoses has increased, particularly in men over the age of 50 years and among men who have sex with men (MSM) [2].

Guidelines produced by the World Health Organization [3] and other organisations [4] suggest that MSM should test for HIV at least once a year, and more frequently if at higher risk of infection. However, the rate at which MSM test for HIV in the UK remains sub-optimal [5], with 1 survey suggesting that 25% of MSM had never tested and 55% had not tested within the previous 12 months [6].

Reducing the time to diagnosis in people with HIV remains a key public health objective in many countries as 60%–80% of all new HIV transmissions are estimated to derive from people who are unaware of their infection status [7]. Moreover, late diagnosis continues to be associated with poorer health outcomes including premature death [8].

Over 80% of HIV tests taken by MSM in England are performed at UK specialist National Health Service (NHS) sexual health services, although testing is available in many different settings including general practice surgeries and community venues [9]. The UK legalised HIV self-testing (HIVST) in April 2014, with the first commercial kit (Biosure) available to purchase starting in 2015. In 2016, around 47,000 tests were performed using HIVST or HIV self-sampling (HIVSS) kits, although not all by MSM [9]. Collectively, we refer to HIVST and HIVSS as ‘remote testing’, but the key difference is that HIVSS kits need to be posted to a laboratory for the test to be conducted and the result returned (often by phone if positive) at a later date. For HIVST, there is no need to return a sample, as individuals perform the diagnostic themselves and interpret their own result. Both types of kits have been shown to be acceptable to people, including MSM [10,11]. In the UK, a number of local health authorities [12] and non-governmental organisations currently provide HIVSS kits free of charge, but HIVST kits are generally only available if purchased privately, at around £30 (around US$40) per kit.

The potential benefit of HIVSS and HIVST kits is that they could increase testing rates by lowering individual and structural barriers to testing, or to testing as frequently as recommended [10]. Indeed, these benefits have been demonstrated in randomised controlled trials (RCTs) among MSM and other populations [13,14]. However, a disadvantage is that the rate at which HIVSS kits are returned is sometimes low [15], and HIVST kits could lead to missed opportunities for detection of common bacterial sexually transmitted infections (STIs) if their use replaces regular clinic visits. Although this was not observed by Jamil et al. [13], Katz et al. [16] recently reported significantly less STI testing by RCT participants randomised to HIVST. There is also concern that the time to confirmatory testing, treatment, and counselling might be longer for people who receive a positive test result using HIVST, which requires the individual to initiate access to services, unlike testing using more traditional HCP-led services [17]. Last, the demand for remote testing options has generally remained low [18], meaning that the extent to which MSM and other communities would prefer to use HIVST and HIVSS kits over other testing options, or not testing, remains unclear. This extends to the kit characteristics most likely to optimise their use [19]. For example, existing RCTs have provided HIVST kits for free, meaning the impact of cost on the likelihood of testing is not fully understood [13,20].

We aimed to address this evidence gap by estimating how different test characteristics could impact the uptake of remote testing (HIVSS and HIVST kits) and the overall probability of testing among MSM in the UK. A further objective was to understand how preferences for the different remote testing kits and other methods of HIV testing vary by sociodemographic factors and risk levels.

## Methods

This cross-sectional discrete choice experiment (DCE) required participants to complete a questionnaire in which they were asked to imagine that ‘they had condomless anal sex with someone whose HIV status they were unsure of’, prior to answering a series of questions (S1 Text) in which they were required to choose which of 3 options they preferred: a ‘remote HIV test’, an ‘HIV test performed by a health care professional’, or ‘not to test’. The presented options were described according to a number of characteristics (known as ‘attributes’) such as how long it takes to get a result. Each attribute also had a number of ‘levels’, such as ‘in 10 minutes’ or ‘in 30 minutes’ for test results, that were varied by each question. The attribute levels were all alternative specific, meaning that, say, the value of taking a blood sample via a skin prick could differ depending on whether it was performed by a HCP or by the participants themselves.

The underlying concept in a DCE is that participants choose the option with the most preferable set of characteristics given the available choices. In this instance, the results indicate the strength of preference for each HIV test characteristic and the likelihood that each testing option is chosen, including the option of not testing. The method has its basis in random utility theory [21]; thus, it has a robust theoretical basis and is an approach that has previously been applied in a number of healthcare studies, including studies of HIV [22,23].

### Choice of attributes and levels

The 8 attributes and associated levels were almost exclusively based on the findings of a recent UK-based qualitative study involving 47 MSM [10] and the study group’s knowledge about the design of existing sexual health services, including HIVST and HIVSS options, in the UK (Table 1). Consideration was also given to the technical capabilities of existing and future remote testing options, such as the infection window period (the time following exposure before a test produces a reliable result). The qualitative study had been undertaken to inform the design of a recent UK-based RCT of remote testing options and was therefore highly relevant to our design needs, and basing DCE designs on qualitative study findings is also regarded as good practice [24]. The maximum remote testing cost of £30 per test was chosen as this is the current approximate retail price of HIVST kits in the UK.

**Table 1:** The discrete choice experiment attributes and levels.

Attribute	Label	Remote testing	HCP testing
Location of test	Location	Somewhere convenient such as your home^a^	Sexual health clinic^b^; general practice; community location such as an HIV charity; mobile clinic based at a bar, club, or sauna
Sampling method	Sample	Oral swab; blood drop via a skin prick^b^	Blood sample via syringe^b^; blood drop via a skin prick
How to obtain the test	Obtain	‘Click and collect’ from a pharmacy or health clinic^b^; order online and post	Drop in and wait^b^; book and attend an appointment
Also tests for infections such as syphilis and gonorrhoea	Infections	HIV only^b^; all infections	HIV only^b^; all infections
Test accuracy	Accuracy	A 95% chance the test result is accurate^b^; a 99% chance the test result is accurate	A 95% chance the test result is accurate^b^; a 99% chance the test result is accurate
Cost of test	Cost	£0; £10; £20; £30^b^	£0^a^
Infection window period	Window	4 weeks^b^; 12 weeks	4 weeks^a^
Wait for test results	Result	There and then in 10 minutes with advice online or via a free phone number^b^; there and then in 30 minutes with advice online or via a free phone number; post sample and receive a call in 3 days from a HCP and advice available from the HCP; post sample and receive a call in 7 days from a HCP and advice available from the the HCP	There and then in 10 minutes and advice available from the HCP^b^; there and then in 30 minutes and advice available from the HCP; receive a call the same day from a HCP and advice available from the HCP; receive a call in 3 days from a HCP and advice available from the HCP

^a^Denotes a fixed level, meaning its value is incorporated within the alternative-specific constant.

^b^Base category.

HCP, healthcare professional.

The selected attributes and levels (Table 1) consisted of (1) testing location (remote testing: 1 level fixed at ‘somewhere convenient’; HCP testing: 4 levels), (2) sampling method (remote testing: 2 levels; HCP testing: 2 levels), (3) how to obtain the test (remote testing: 2 levels; HCP testing: 2 levels), (4) the inclusion of tests for bacterial STIs other than HIV (remote testing: 2 levels; HCP testing: 2 levels), (5) test accuracy as a percentage (remote testing: 2 levels; HCP testing: 2 levels), (6) test cost (remote testing: 4 levels; HCP testing: fixed at £0), (7) infection window period (because the design already included an ‘accuracy’ attribute, the window period was simply described as the time a person would have to wait before a test could be taken—remote testing: 2 levels; HCP testing: fixed at 4 weeks), and (8) the waiting time for a test result (remote testing: 4 levels; HCP testing: 4 levels). This final attribute combined information on how the results were returned and how advice was accessed following a positive or negative test result: These constructs were merged together because the level on one was conditional on the value of another, meaning their independent effects could not be isolated. For example, using an HIVSS kit means that results are returned after several days rather than within a few minutes, and that advice about the result is available via the HCP who returns the result. On the other hand, the use of an HIVST kit implies that results are obtained immediately, but that advice about the result is only available via online media or a free phone number rather than from the person returning the sample results.

### DCE instrument design

The instrument was designed using a D-efficient approach with 24 choice tasks (questions) using Ngene software [25], ensuring that preferences for each of the attribute levels could be independently assessed. However, the 24 DCE questions were divided into 2 sets of 12 DCE questions to reduce the number of questions each participant needed to complete—a process known as blocking [26].

Participants were also asked to provide information on sociodemographic and risk factors including age (continuous variable), gender (male; transgender; other/prefer not to say), sexuality (gay; bisexual; heterosexual/straight; prefer not to say), highest educational qualification (none; O levels/GCSE [secondary education to age 16 years]; A levels [education to age 18 years]; university degree or higher; other), ethnicity (white; black African; Asian; mixed race; other), current HIV pre-exposure prophylaxis [PrEP] use (yes; no), condomless anal sex (CLS) with a new or casual partner (never; <3 months ago; 3–6 months ago; 6 months–2 years ago; 2–5 years ago; 5+ years ago), ever previously tested for HIV (yes; no), and ever used an HIVST or HIVSS kit (yes; no; unsure). The responses of the first 10 respondents were analysed to ensure that participants understood the task and that the instrument produced logical results (e.g., lower remote testing costs were preferred to higher costs). A further check for grammatical errors and ambiguities in the text was also made at this point—none were identified.

### Data collection

Between 3 April and 11 May 2017, participants were recruited via a paid advert placed on Facebook for a total cost of £610, and explicitly aimed at a broad range of MSM. The advert was presented only to men living in the UK who expressed an interest in online material used by MSM. People who clicked on the embedded link were redirected to a webpage containing the survey. All participants were required to state that they were at least 16 years old and without a positive HIV diagnosis. No other exclusion criteria were applied. Individuals were offered the chance to win a £50 voucher as an incentive to participate. Ethical approval for the study was obtained from Brighton and Sussex Medical School (16/026/LLE) and the London School of Hygiene & Tropical Medicine (11876). Participant informed consent was gathered by means of an online tick box. The study protocol is provided as S1 Protocol. This study is reported as per the Strengthening the Reporting of Observational Studies in Epidemiology (STROBE) guidelines (S1 Checklist).

Determining sample sizes in advance of conducting DCEs is difficult, not least because the questionnaire design is unknown at the study’s outset. However, as it has been suggested that a reasonable sample size is 300, we aimed to recruit at least 600 participants with complete answers (2 × 300, given the blocking) [27].

### Statistical analysis

All attribute levels were dummy-coded except when estimating the alternative-specific constant (ASC), when effects coding was used in order to avoid confounding with the base level. The 2 ASCs in this instance represent the extent to which respondents preferred ‘not to test’ or ‘HCP testing’ compared to ‘remote testing’, independently of the attribute levels. The results are presented as odds ratios (ORs) relative to the relevant base category (Table 1) and the probabilities of uptake for the remote testing option. Reported standard errors are adjusted in all instances to account for the potential clustering in participant responses.

Many different models can be used to analyse DCE results, but following general advice, we first used the most basic type—the conditional logit (CLOGIT) model [28] (see S1 Appendix). Unlike in standard logistic regression, the results from CLOGIT models are ‘conditional’ on the information relating to all the choice options as this information is grouped before analysis. Here, for example, results for each completed question are represented by 3 rows of data, with each containing the attribute levels for each choice (HCP testing, remote testing, and not testing), with an additional variable indicating which of the 3 options was chosen. In this sense, CLOGIT models are analogous to matched case–control approaches and investigate the relationship between a choice (case), options that were not chosen (controls), and a set of predictive factors (attribute levels).

The CLOGIT model is the simplest form of analysis. Although it is recommended to be used for the initial analysis [26], there are 2 main limitations with it. First, it includes the simplifying ‘independence of irrelevant alternatives’ assumption. Under this assumption, the likelihood of choosing each option changes by the same proportion if any of them are omitted from the analysis. However, this is unlikely to be realistic in our study since the HCP and remote testing options are much more similar to each other (i.e., they both involve testing) than the choice not to test. Second, the CLOGIT model produces results for the ‘average’ individual, meaning that no allowance is made for the possibility that different groups of people within the sample (e.g., different age groups) might have varying preferences—this is known as ‘preference heterogeneity’.

As an alternative to the CLOGIT approach, we also analysed the results using a latent class model (LCM), as it simultaneously relaxes the independence of irrelevant alternatives assumption and allows potential preference heterogeneity to be examined. LCMs are specifically recommended if groups of respondents with similar preferences (here for testing options) are anticipated.

LCMs assume there are subgroups of individuals (classes) with similar preferences, and that the likelihood of class membership can be related to observed variables. The potential predictors of class membership in this analysis were age (as a continuous variable), ethnicity (white; non-white), currently taking PrEP (yes; no), educational qualification (none; GCSEs; A levels; university degree or higher), and risk status (never tested for HIV and never had CLS; never tested and previously had CLS; previously tested and never had CLS; previously tested and had CLS). The number of classes in the LCM was based on minimisation of Akaike’s information criterion (AIC) and the production of stable/meaningful standard errors. The CLOGIT and LCM analyses were undertaken using Stata version 15 [29] and NLOGIT 5 [30], respectively.

## Results

A total of 1,285 respondents clicked on the link and started to complete the survey. However, 618 did not complete the DCE questions, and 47 were excluded as they reported an HIV-positive diagnosis. The analysis sample therefore contained 620 participants. There was no evidence to suggest that people who did versus did not complete the DCE questions differed in terms of factors such as age (*p =* 0.69), self-reported sexual preference (*p =* 0.44), or ethnicity (*p =* 0.44). However, there was evidence suggesting people with higher educational qualifications were more likely than those with lower or no educational qualifications to complete the DCE questions (*p <* 0.001), to have tested for HIV at least once in the past (*p <* 0.001), and to have used an HIVST or HIVSS kit (*p =* 0.001).

The mean age of the analysis sample was reported to be 31.1 years, and most respondents stated they were white (93%) and were either gay or bisexual (99%) (Table 2). Almost 22% of respondents reported they had never tested for HIV, and 3% were currently receiving PrEP. A substantial proportion of participants chose the same testing option (203/620, 33%) for all 12 DCE questions, mainly testing by a HCP (166/620, 27%).

**Table 2:** Sample characteristics.

Characteristic	Completed DCE questions *N* = 620^a^	Did not complete DCE questions *N* = 618^a^	*p*-Value
**Age (years), mean [SE]**	31.1 [0.47]	31.4 [0.60]	0.69^b^
**Transgender identity**	14 (2.3)	8 (1.8)	0.67^c^
**Sexuality**			
Gay	528 (85.2)	376 (83.8)	
Bisexual	84 (13.6)	61 (13.6)	
Heterosexual	4 (0.7)	6 (1.3)	
Prefer not to say	4 (0.7)	6 (1.3)	0.44^d^
**Education**			
None	32 (5.2)	34 (7.6)	
O levels/GCSE	91 (14.7)	126 (28.1)	
A levels	174 (28.1)	114 (25.4)	
University degree or higher	300 (48.4)	151 (33.6)	
Other	23 (3.7)	24 (5.4)	<0.001^d^
**Ethnicity**			
White	574 (92.6)	421 (93.8)	
Black African	7 (1.1)	2 (0.5)	
Asian	19 (3.1)	9 (2.0)	
Mixed race	20 (3.2)	17 (3.8)	0.44^c^
**Ever tested for HIV**			
No	134 (21.6)	152 (37.8)	
Yes	486 (78.4)	250 (62.2)	<0.001^d^
**Last tested for HIV**			
<3 months	160 (25.8)	63 (15.7)	
3–6 months	104 (16.8)	40 (10.0)	
6 months to 2 years	130 (21.0)	76 (18.9)	
2 to 5 years	55 (8.9)	38 (9.5)	
>5 years	34 (5.5)	30 (7.5)	
Prefer not to say	3 (0.5)	3 (0.8)	
Never	134 (21.6)	152 (37.8)	<0.001^c^
**Currently on PrEP**			
No	585 (97.0)	345 (96.1)	
Yes	18 (3.0)	14 (3.9)	0.44^d^
**Last condomless anal sex (CLS)**			
Never	135 (21.8)	104 (27.7)	
<3 months	182 (29.4)	109 (29.0)	
3–6 months	68 (11.0)	37 (9.8)	
6 months to 2 years	100 (16.1)	49 (13.0)	
>2 years	132 (21.3)	67 (17.8)	
Prefer not to say	3 (0.5)	10 (2.7)	0.01^c^
**Number of new or casual CLS partners in last year**			
None	295 (47.6)	191 (50.8)	
1	149 (24.0)	97 (25.8)	
2–4	129 (20.8)	64 (17.0)	
5–10	25 (4.0)	14 (3.7)	
>10	22 (3.6)	10 (2.7)	0.53^d^
**Ever used an HIVST or HIVSS kit**			
No/unsure	462 (74.5)	335 (83.3)	
Yes	158 (25.5)	67 (16.7)	0.001^d^

Values are number (percent) unless otherwise indicated.

^a^*N* may vary due to missing answers.

^b^*t* test.

^c^Fisher’s exact test.

^d^Chi-squared test.

DCE, discrete choice experiment; HIVSS, HIV self-sampling; HIVST, HIV self-testing; PrEP, pre-exposure prophylaxis.

### Model results

The signs on the coefficients from both the CLOGIT and LCM analyses were generally in the expected direction, giving some reassurance as to the validity of both models. For example, participants consistently preferred shorter to longer waiting times for test results and lower to higher costs of buying a remote testing kit. Hereafter we only report the results of the LCM analysis because, in addition to being the more theoretically appropriate model to use, it predicted a higher number of correct responses than the CLOGIT model: 77.6% versus 74.8%. Thus, it was considered a better fit to the data.

**Fig 1:**
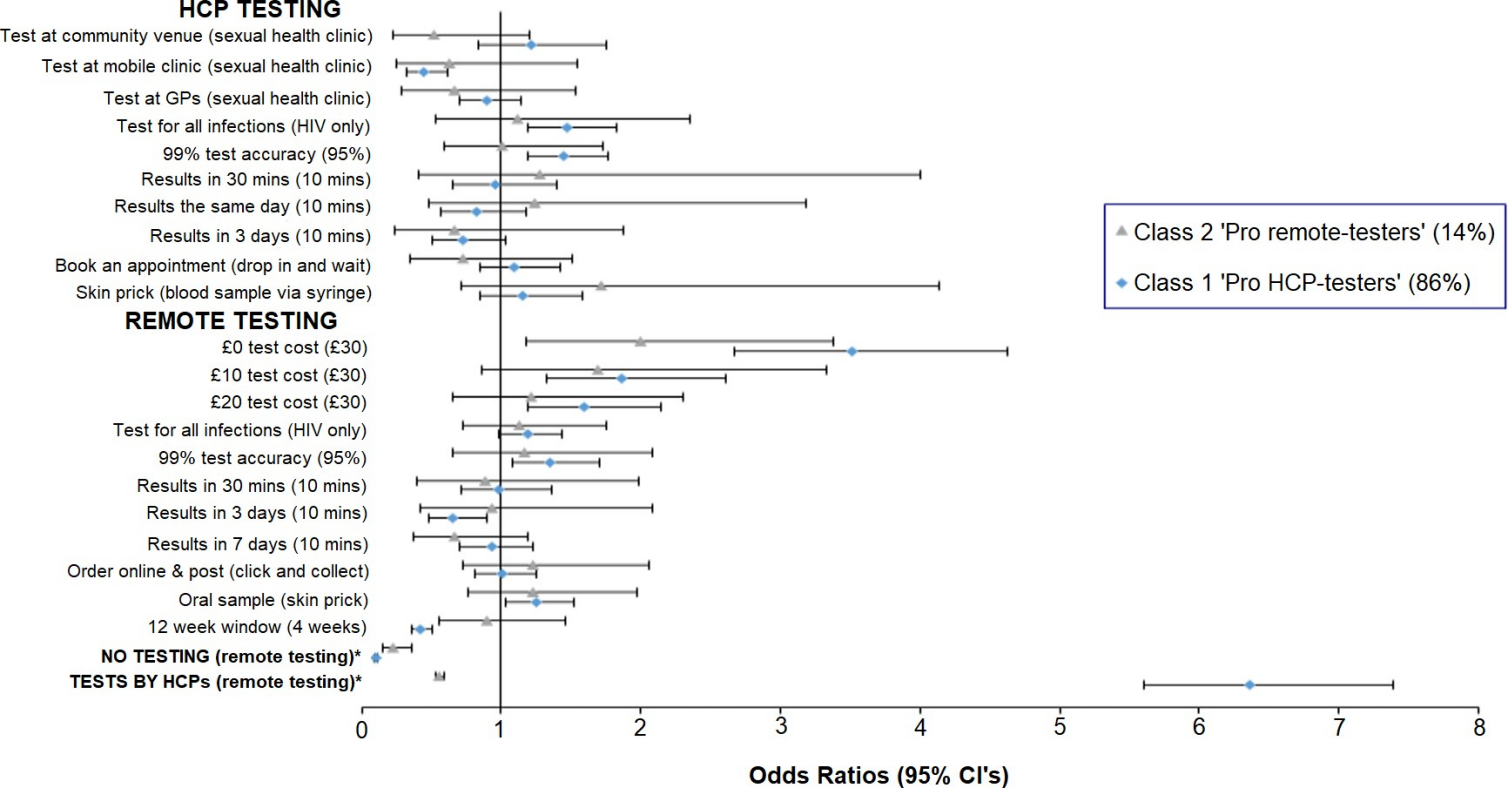
HIV testing preferences—Results from the latent class model. Levels in parentheses denote the base category; levels with the same base category are part of the same attribute. Odds ratios above 1 indicate a preference for the stated attribute level, whereas values below 1 indicate a preference for the base category. *Odds ratios for the alternative-specific constants, which were derived using effects coding. The alternative-specific constants indicate the strength of preference for no testing and HCP testing, both compared to remote testing. GP, general practice; HCP, healthcare professional.

The LCM identified 2 classes, with 86% and 14% of participants likely to be in classes 1 and 2, respectively—the standard errors became noticeably unstable if 3 or more classes were used, and the AIC increased. The ASCs showed that those in class 1 had a strong preference for HCP testing compared to remote testing (OR 6.4; 95% CI 5.6, 7.4). They also viewed not testing as undesirable compared to remote testing (OR 0.10; 95% CI 0.09, 0.11)—we refer to this group as ‘pro HCP testers’ (Fig 1). Remote tests being free rather than £30 per test (OR 3.52; 95% CI 2.67, 4.63) was the largest predictor of the choice of test option (HCP, remote, or not testing), although reductions to £10 (OR 1.86; 95% CI 1.33, 2.61) and £20 (OR 1.59; 95% CI 1.19, 2.14) were also valued. Other remote testing characteristics that were valued included kits being 99% rather than 95% accurate (OR 1.35; 95% CI 1.08, 1.70) and using oral swabs rather than a blood drop from a skin prick (OR 1.25; 95% CI 1.03, 1.52). However, a 12-week rather than 4-week window period was strongly disliked (OR 0.43; 95% CI 0.35, 0.51), as were test results being returned in 3 days rather than 10 minutes (OR 0.66; 95% CI 0.48, 0.90).

In terms of HCP testing, class 1 members again expressed a preference for tests that were 99% rather than 95% accurate (OR 1.45; 95% CI 1.20, 1.77) and tests for all listed infections rather than just HIV (OR 1.47; 95% CI 1.19, 1.83). They also conveyed a dislike for tests at mobile clinics compared with tests performed at sexual health clinics (OR 0.45; 95% CI 0.33, 0.61).

For class 2 members, not testing was viewed as less desirable than remote testing (OR 0.56; 95% CI 0.53, 0.59), as were tests by HCPs compared to remote testing (OR 0.23; 95% CI 0.15, 0.36). Similarly to class 1 members, those in class 2 expressed a preference for free remote tests to those costing £30 (OR 2.0; 95% CI 1.18, 3.37). We refer to this group as ‘pro remote testers’. Preferences for the remaining remote and HCP testing characteristics followed a similar pattern to those of class 1 members, although in all cases the *p*-value associated with each OR was >0.05.

Participants were more likely to be pro remote testers than pro HCP testers if they were currently taking PrEP (*p =* 0.007), compared to those who were not, or if they had never previously tested and never had CLS (*p =* 0.03) or had never previously tested and had CLS (*p =* 0.002), compared to those who had previously tested. However, respondents were more likely to be pro HCP testers if they were white rather than non-white (*p =* 0.04). None of the remaining characteristics were predictive of class membership (*p* > 0.05 in all instances).

The individual remote test characteristics altered the probability of preferring remote or HCP testing to a greater or lesser extent, but had only a relatively small impact on the overall probability of preferring not testing (Table 3). For example, reducing the remote test cost from £30 (its base level) to £0 per kit increased the probability of preferring remote testing by 15.3%, but the probability of preferring not testing only decreased by 2%. The main change associated with the £0 cost of remote testing was a reduction in the probability of preferring HCP testing by 13.3%.

**Table 3:** The predicted impact of changing remote test characteristics on the average probability of choosing different testing options, derived from the latent class model.

Characteristic	Base	Percent change		
		Remote testing	HCP testing	No test
12-week infection window	4 weeks	−8.8	7.7	1.0
Oral sample	Skin prick	2.9	−2.4	−0.5
Order online and post	Click and collect	0.8	−0.6	−0.2
Results in 7 days	10 minutes	−2.1	1.8	0.3
Results in 3 days	10 minutes	−4.1	3.5	0.6
Results in 30 minutes	10 minutes	−0.5	0.4	0.1
99% accurate test	95%	3.5	−3.0	−0.5
Test for HIV and other infections	HIV only	2.1	−1.8	−0.3
£0 per kit	£30	15.3	−13.3	−2.0
£10 per kit	£30	7.0	−5.8	−1.2
£20 per kit	£30	4.4	−3.6	−0.8

Overall probability of choice: remote testing 21%, HCP testing 75%, and no test 4%.

HCP, healthcare professional.

## Discussion

A DCE was conducted to assess MSM preferences for different HIV test characteristics. Participants were recruited via social media using an advert directed at people who had expressed an interest in online material used by MSM. The results from this study suggest that most MSM living in the UK are comfortable testing for HIV, but generally prefer face-to-face tests by HCPs to using HIVST or HIVSS kits. Altering some of the remote test characteristics significantly affected the likelihood of choosing either a remote or HCP testing approach, but generally had a small impact on the overall probability of not testing. This said, there appears to be a minority of MSM for whom the availability of HIVST and HIVSS kits has the potential to significantly increase the uptake and frequency of testing, which includes people at increased risk of undetected infection.

Our results suggest that the main barrier to remote testing use was its purchase cost, followed by the length of window period. Existing studies among MSM and other populations have drawn similar conclusions [10,11,31–34], but our findings additionally suggest that most people would still prefer HCP to remote testing even if the latter was free and available with a 4-week window period. This is because the majority of participants placed such a high value on HCP testing over and above the specific test characteristics that we investigated. Our study does not say why this is so, but others have previously highlighted the importance to MSM of the general opportunity HCP testing affords for interaction with HCPs [35,36]. Given that the majority of MSM preferred face-to-face testing services, although generally not at mobile clinics, maintaining a mixed landscape of testing modalities is clearly of importance in ensuring that their testing needs are met. Remote testing options in this context should therefore be understood as supplementary to HCP-led services.

While HCP testing was generally preferred to remote testing, our LCM results support previous evidence [35] in showing that a minority (14%) of MSM prefer remote testing (pro remote testers) to both HCP testing and not testing, meaning that it has the potential to increase net testing frequency [13]. However, even for people who are likely to be in this group, providing remote tests free rather than each costing £30 is likely to further increase their use.

The analysis also showed that people who reported receiving PrEP generally preferred remote testing, but in the absence of this option they preferred not to test than to be tested by a HCP. At first glance this finding might be a concern, but, importantly, participants were asked to choose whether and how they would prefer to test following CLS. Thus, given this context, and that people on PrEP are advised to test regularly for HIV and other infections, it is plausible that respondents simply viewed additional HCP testing as unnecessary. In addition to being more likely to be PrEP users, pro remote testers were more likely to be people who had never previously tested for HIV and people who self-identified as being of non-white ethnicity. These results are consistent with the existing evidence [35] and further suggest that remote testing options could be effective methods of reaching people at high risk of infection who are facing particularly elevated barriers to testing [37]. This includes black African MSM, who are a priority group for intervention because they have a significantly higher HIV prevalence and incidence than other groups [9].

Remote testing involves using either an HIVST or an HIVSS kit. The main advantage of HIVST kits is that they provide results within a relatively short time period. However, some are recommended for use 3 months after potential exposure, and they are not currently provided free by the UK NHS. Using the LCM results for HIVST and HIVSS kits respectively costing £30 and £0, providing results in 10 minutes and 7 days (the extreme options in the design), and having a window period of 12 and 4 weeks shows that while participants continued to generally prefer tests by HCPs, they were more likely to choose to self-sample than self-test (likelihood of choice: 37% versus 13%). Reducing the HIVST window period to 4 weeks increased the probability of remote testing using an HIVST kit (37% versus 20%), but HIVST kits only became more preferable than HIVSS kits when also provided free (40% versus 37%).

Even though the general limitation that DCEs require participants to make hypothetical rather than observed choices is acknowledged, there is growing evidence that they do predict actual behaviour [38–40]. Participants were recruited via social media but because the advert was only viewed by people expressing an interest in online material used by MSM, as identified by the use of key words, the sample could have excluded men with concerns about disclosing their sexual orientation, who may have the most pronounced HIV prevention needs [41]. Although this means there are potential limitations to the generalisability of our sample, previous research has shown that social media advertisements are capable of attracting MSM who are less likely to report a gay identity, male-only partnerships, and recent HIV testing [42]. We recruited a broad group of men who expressed an interest in gay online material on their Facebook account. We acknowledge that sexual orientation identity is not always consistent with behaviour or gender identity. In line with common use in the literature, our use of the term MSM may include a minority of people who have never had sex with a man, who identify as non-binary, or who have a heterosexual identity.

Another limitation with our study is the simplification of assessing concerns about test accuracy, which was stated as the choice between a 95% and 99% accurate test. Because of this, we are uncertain as to how particular issues around the sensitivity and specificity of the tests could affect individuals’ preferences for testing. Participants were asked to complete the DCE questions imagining they had recently had CLS. However, Witzel et al. suggest there are 2 other main reasons MSM might consider HIV testing: as reassurance when there is doubt or anxiety related to HIV and in response to peer-related social norms [36]. Thus, finally, we acknowledge that our results might not be generalizable to all contexts in which testing could be contemplated.

There are also strengths to this study. Participants were recruited via social media advertisements because of their wide national reach and common use by MSM [43] and to include people who had not previously tested for HIV. Moreover, the composition of our sample is broadly comparable to that of a recent large survey of MSM in the UK, suggesting it might be representative of this population [44].

### Conclusions

Ensuring that MSM test regularly for HIV is key to reducing late diagnosis and its associated poorer health outcomes, higher costs, and further transmissions. While it is recognised that the factors mediating the decisions of MSM about HIV testing are complex [35], our results suggest that preferences in the context we examined are divided. One group, containing the majority of MSM, appears comfortable testing for HIV but in nearly all circumstances they prefer face-to-face testing by HCPs compared to using HIVST or HIVSS kits. The other group is smaller in comparison but, importantly, contains MSM who are more likely to be at high infection risk. For these people, the availability of and access to HIVST and HIVSS kits has the potential to significantly increase net testing rates, especially if provided for free. We believe that policy makers and commissioners should be attentive to these preferences, ensuring that a wide mix of services is available to meet the diverse testing needs of MSM. This is a potentially critical issue given the temptation to reduce the provision of more costly HCP-led testing services in favour of remote testing options.

## Supporting information

S1 Checklist STROBE checklist. (DOCX)

S1 Appendix Appendix. (DOCX)

S1 Protocol Research protocol. (DOCX)

S1 Text Questionnaires. (DOCX)

## Abbreviations

AIC: Akaike’s information criterion
ASC: alternative-specific constant
CLOGIT: conditional logit
CLS: condomless anal sex
DCE: discrete choice experiment
HCP: healthcare professional
HIVSS: HIV self-sampling
HIVST: HIV self-testing
LCM: latent class model
MSM: men who have sex with men
NHS: National Health Service
OR: odds ratio
PrEP: pre-exposure prophylaxis
RCT: randomised controlled trial
STI: sexually transmitted infection
