# Supplementary material for: Preferences for HIV testing services among men who have sex with men in the UK: A discrete choice experiment
Source: PLoS Med. 2019 Apr 11;16(4):e1002779. doi: 10.1371/journal.pmed.1002779 (PMC6459507; doi:10.1371/journal.pmed.1002779)
Supplement: S1 Checklist — (DOCX) [file pmed.1002779.s001.docx]

**STROBE 2007 (v4) Statement—Checklist of items that should be included in reports of *cohort studies***

| **Section/Topic** | Item # | Recommendation | Reported on page # |
| --- | --- | --- | --- |
| **Title and abstract** | 1 | (*a*) Indicate the study’s design with a commonly used term in the title or the abstract | See title and abstract |
|  |  | (*b*) Provide in the abstract an informative and balanced summary of what was done and what was found | See abstract |
| Introduction | | |  |
| Background/rationale | 2 | Explain the scientific background and rationale for the investigation being reported | See introduction paragraphs 1-5 |
| Objectives | 3 | State specific objectives, including any prespecified hypotheses | See introduction paragraph 6 |
| Methods | | |  |
| Study design | 4 | Present key elements of study design early in the paper | See methods section paragraph 1 |
| Setting | 5 | Describe the setting, locations, and relevant dates, including periods of recruitment, exposure, follow-up, and data collection | See methods section paragraphs 7 and 8 |
| Participants | 6 | (*a*) Give the eligibility criteria, and the sources and methods of selection of participants. Describe methods of follow-up | See methods section paragraphs 7 and 8 |
|  |  | (*b*) For matched studies, give matching criteria and number of exposed and unexposed | n/a |
| Variables | 7 | Clearly define all outcomes, exposures, predictors, potential confounders, and effect modifiers. Give diagnostic criteria, if applicable | Only odds ratios and associated probabilities are reported. See ‘statistical analysis’ paragraph 1 |
| Data sources/ measurement | 8* | For each variable of interest, give sources of data and details of methods of assessment (measurement). Describe comparability of assessment methods if there is more than one group | See under ‘DCE instrument design’ paragraph 2, and under ‘data collection’ paragraph 1 |
| Bias | 9 | Describe any efforts to address potential sources of bias | Respondents who completed the main study questions were compared to those who did not complete them. The first paragraph in the results section |
| Study size | 10 | Explain how the study size was arrived at | See under ‘data collection’ paragraph 2 |
| Quantitative variables | 11 | Explain how quantitative variables were handled in the analyses. If applicable, describe which groupings were chosen and why | See pages 7-9 |
| Statistical methods | 12 | (*a*) Describe all statistical methods, including those used to control for confounding | See under ‘statistical analysis’ paragraphs 1-5 |
|  |  | (*b*) Describe any methods used to examine subgroups and interactions | See under ‘statistical analysis’ paragraphs 1-5 |
|  |  | (*c*) Explain how missing data were addressed | n/a |
|  |  | (*d*) If applicable, explain how loss to follow-up was addressed | n/a |
|  |  | (*e*) Describe any sensitivity analyses | n/a |
| Results | | |  |
| Participants | 13* | (a) Report numbers of individuals at each stage of study—eg numbers potentially eligible, examined for eligibility, confirmed eligible, included in the study, completing follow-up, and analysed | See the first paragraph in the results section |
|  |  | (b) Give reasons for non-participation at each stage | n/a |
|  |  | (c) Consider use of a flow diagram | n/a |
| Descriptive data | 14* | (a) Give characteristics of study participants (eg demographic, clinical, social) and information on exposures and potential confounders | See table 2 for a full list |
|  |  | (b) Indicate number of participants with missing data for each variable of interest | There was no missing data for the people who completed the DCE questions |
|  |  | (c) Summarise follow-up time (eg, average and total amount) | n/a |
| Outcome data | 15* | Report numbers of outcome events or summary measures over time | n/a |
| Main results | 16 | (*a*) Give unadjusted estimates and, if applicable, confounder-adjusted estimates and their precision (eg, 95% confidence interval). Make clear which confounders were adjusted for and why they were included | See all the paragraphs under ‘model results’ and Figure 2 |
|  |  | (*b*) Report category boundaries when continuous variables were categorized | n/a |
|  |  | (*c*) If relevant, consider translating estimates of relative risk into absolute risk for a meaningful time period | n/a |
| Other analyses | 17 | Report other analyses done—eg analyses of subgroups and interactions, and sensitivity analyses | n/a |
| Discussion |  |  |  |
| Key results | 18 | Summarise key results with reference to study objectives | See the first paragraph in the discussion section |
| Limitations | 19 | Discuss limitations of the study, taking into account sources of potential bias or imprecision. Discuss both direction and magnitude of any potential bias | See the discussion section paragraph 6 |
| Interpretation | 20 | Give a cautious overall interpretation of results considering objectives, limitations, multiplicity of analyses, results from similar studies, and other relevant evidence | See the paragraphs 1 and 2 in the discussion section |
| Generalisability | 21 | Discuss the generalisability (external validity) of the study results | See the discussion section paragraph 6 |
| Other information |  |  |  |
| Funding | 22 | Give the source of funding and the role of the funders for the present study and, if applicable, for the original study on which the present article is based | A funding statement is included in the manuscript – no funding was received for this project |
