# Supplementary material for: Preferences for HIV testing services among men who have sex with men in the UK: A discrete choice experiment
Source: PLoS Med. 2019 Apr 11;16(4):e1002779. doi: 10.1371/journal.pmed.1002779 (PMC6459507; doi:10.1371/journal.pmed.1002779)
Supplement: S1 Appendix — (DOCX) [file pmed.1002779.s002.docx]

**Appendix**

For further reading, see Hensher DA, Rose JM and Greene, WH. Applied Choice Analysis. Second Edition, Cambridge University Press, Padstow, 2015.

Model specification

The basic functional form is derived from random utility theory. For each discrete choice question, participants were assumed to choose the option (remote testing, testing by a health care professional [HCP] or no testing) that maximized their utility levels, where the utility of each choice was estimated as:

$$U_{nsj}=V_{nsj}+\varepsilon_{nsj}$$

Where *Unsj* is the overall utility for decision maker *n* in choice situation (question) *s* for alternative *j* (here remote testing, testing by HCP] or no testing). *Vns*j is the observed part of the utility function that results from attributes for each alternative, and *Ɛnsj* is a unobserved residual component of utility.

The purpose of the discrete choice experiment (DCE) is to estimate *Vnsj*, which can be represented as:

$$V_{nsj}=\sum_{k=1}^{K} \beta_{k}x_{nsjk}$$

Where *xnsjk* is a set of k attributes (characteristics) in the DCE design (such as the cost of remote-testing, the window period etc), each with a *β* coefficient to be estimated (denoting the strength of preference for each attribute). The function can be estimated with the inclusion of a set of ‘alternative specific constants (ASCs)’. In our example, two ASCs are included which represent the strength of relative preference for HCP-testing compared to remote-testing, and remote-testing compared to no testing, independently of the other attribute levels. ASCs are usually included in DCE designs when there is reason to be believe the choices provide utility to decision makers that is not captured by the attributes.

In the conditional logit (clogit) analysis, the probability of alternative *j* being chosen (because it has the highest utility) is given by:

$${Probability}_{j}=\frac{\exp{(V}_{nsj})}{\sum_{j=1}^{J} {(\beta}_{k}x_{nsjk})} j=1,\ldots j$$

In other words the probability an individual will choose option *j*, which is described by the design attributes, is conditional on the set of available *J* options and how they have been designed too. The important point to note is that any individual will only choose option *i* if it produces the highest level of utility across all options. When it does, it will by definition have the highest probability of being chosen.

In the latent class model, class specific-utility functions are estimated. They are calculated in a similar way to the CLOGIT model but are conditional on the likelihood of belonging to a given class. In turn, the probability of being in each class is determined by a set of measurable characteristics such as age and sexual history.
