# Supplementary material for: Preferences for HIV testing services among men who have sex with men in the UK: A discrete choice experiment
Source: PLoS Med. 2019 Apr 11;16(4):e1002779. doi: 10.1371/journal.pmed.1002779 (PMC6459507; doi:10.1371/journal.pmed.1002779)
Supplement: S1 Protocol — (DOCX) [file pmed.1002779.s003.docx]

**RESEARCH PROTOCOL**

| **How do gay and other men who have sex with men value HIV self-testing and -sampling kits - a pilot study? A discrete choice experiment** | |
| --- | --- |
| **SHORT TITLE:** | The HIV Prevention DCE |
| **VERSION NUMBER*:** | v1.0 |
| **DATE:** | 05/06/2016 |

1. **Chief Investigator**

| **Chief investigator** | |
| --- | --- |
| **Name and Title:** | Dr Carrie Llewellyn |
| **Post :** | Associate Professor in Applied Behavioural Medicine |
| **Department/ institution:** | Division of Public Health and Primary Care  Brighton and Sussex Medical School |
| **Address:** | Mayfield House  Falmer, Brighton, BN1 9PH |
| **Telephone number:** | 01273 642 187 |
| **Email:** | c.d.llewellyn@bsms.ac.uk |

| 1. **Background Information** |
| --- |
| There is a clear national need for a renewed focus on preventing HIV infection. This is because there are approximately 110,000 people living with HIV in the UK, of whom 25% are unaware they are infected [1]. Approximately 6,000 new cases of HIV were diagnosed in 2014, with over half in men who have sex with men (MSM) meaning that as a group they are at high risk of infection [1]. HIV testing, uptake and frequency remains sub-optimal. Recent community surveys suggest that approximately 25% of MSM have never tested for HIV, 55% have not tested in the previous year and less than a quarter of MSM at higher risk of HIV infection are testing at the recommended frequency of every three months [2, 3]. It is also estimated that 60% to 80% of all new HIV transmissions come from people who are unaware they are infected [[3](#_ENREF_3)].  Decreasing the time from HIV infection to diagnosis is important for a number of reasons. First, people living with HIV in resource rich countries now have a near-normal life span if diagnosed and treated promptly [4]. However, the proportion in whom diagnosis does not occur until a late stage (CD4<350 cells/mm^3^ within 3 months of diagnosis) remains above 40% of all new diagnoses [1]. This group have a ten-fold increased risk of death in the year following diagnosis compared to those promptly diagnosed [5]. Second, despite major treatment advances and greatly improved prognosis, HIV infection continues to significantly impact on quality-of-life [6]. Third, effective treatment for HIV supresses the virus, meaning the transmission risk is greatly reduced [7-9]. Last, each newly diagnosed case of HIV costs approximately £360,000 to treat over a person’s lifetime [10], although they are significantly higher in people who are diagnosed late [11, 12]. Thus, earlier diagnosis is likely to maintain people’s health, prevent further infections and help to reduce future NHS costs.  There are multiple possible approaches to HIV prevention, but it is presently unclear to local and national policy makers which approach or combination of approaches offers the greatest benefit given the cost. Until recently interventions have mainly focussed on condom use. This remains critically important, but additional approaches are needed, not least because while condom use is common, consistent condom use is much rarer. Recently, NHS England have approved initiation of ART in people regardless of CD4 count to improve PLWHIVs health but also to reduce infectivity (treatment for prevention). Another approach is pre-exposure prophylaxis (PreP, where HIV negative people take HIV drugs regularly to prevent infection) and post exposure prophylaxis (PEP) but these are only available in a very limited way on the NHS currently.  In 2014, over 1 million HIV tests were performed in the UK at sexual health- or genitourinary-clinics [[2](#_ENREF_2)]. But HIV tests are also performed at a wide range of outreach services and at GP surgeries. A more recent option is for people to test themselves for HIV using either a STK or SSK. The key difference between the two types of kit is that SSK samples need to be returned to a laboratory for the test to be conducted and the result is then returned by phone or text generally a week later. For STKs the result is obtained almost immediately. It became legal to sell STKs in England, Wales and Scotland in April 2014. Only one STK currently has a European CE mark, thus can be actively sold in the UK (Biosure). It can be purchased online and from some high-street pharmacies for around £30-35. However, other companies may seek certification in the future and their products can already be purchased privately from websites hosted overseas. The sensitivity and specificity of the STKs and SSKs are generally similar but they vary in terms of a number of important characteristics such as the time it takes for a test result (1 to 20 minutes for STKs, normally a week for SSKs), how the sample is taken (oral or blood) and the ‘window period’ (the time between a person becoming infected and HIV infection being detectable, usually 1 to 3 months).  There are potential advantages and disadvantages of STK and SSKs compared with other HIV testing options. The major perceived benefit is that they could increase testing rates among gay men who are either 1) uncomfortable visiting a traditional clinic or 2) do not have the time or opportunity to test as frequently as recommended. However, there are some important potential disadvantages. First, regular clinic-based sexual health screens (which include testing for all other STIs as well as HIV) might be substituted for HIV self-testing / sampling. Second, people who test positive for HIV may not subsequently link to HIV care for confirmatory testing / treatment, which is less likely to happen if using routine clinical services [[17](#_ENREF_17)]. There is also considerable uncertainty over the potential demand for these kits and their characteristics that are most likely to optimise their use [[18](#_ENREF_18)]. |

| 1. **Aims and Objectives** |
| --- |
| The primary objective of this study is to assess the circumstances under which gay men would test for HIV using self-testing kits (STKs) or self-sampling kit (SSKs). The basic principle underpinning it is that people are more likely to use health services if the relevant communities are involved in their design. In this instance, this means assessing MSMs preferences for different HIV testing options, and understanding how different service designs are likely to influence uptake. The specific objectives are to assess:   - Preferences for HIV self-testing and self-sampling options among gay men - How preferences vary by socio-demographic factors (e.g. sexual identity), risk levels (e.g. condomless sex with partners of unknown infection status), partner status (e.g. long-term) and previous testing behaviour - The potential rate of substitution between self- and clinic-based testing - How the price of self-testing / sampling impacts on demand |

| 1. **Existing Literature** |
| --- |
| Two recent literature reviews have assessed the acceptability and preferences for HIV self-testing [[17](#_ENREF_17), [21](#_ENREF_21)]. They broadly conclude that it is acceptable in key populations. However, the reviews are of limited use in terms of understanding how the uptake of testing options might be affected by different self-testing characteristics as the studies were not designed to answer this question [[22-25](#_ENREF_22)]. Two studies, however, did have this capability. Both were DCEs that included HIV self-testing as an option. The first was set in a resource poor setting and thus has limited relevance in a UK context [[26](#_ENREF_26)]. The second, was performed in the US in 1999 (n=365, 50% of whom were gay men) [[27](#_ENREF_27)]. However, it too is of limited value, since it was performed in an era when most HIV treatment options had limited efficacy and excluded important characteristics such as the ‘window period’. |

| 1. **Study Design and Methodology** |
| --- |
| We aim to address this evidence gap by performing a questionnaire based discrete choices experiment (DCE) [[19](#_ENREF_19)]. DCEs are a patient-centred theoretically robust method of estimating potential uptake given different service design characteristics. In this instance the DCE will be with online recruitment and data capture. Participants will be required to choose between a number of hypothetical HIV testing options, or a decision not to test.  **Recruitment**  Self-identified men who have sex with men, aged ≥16; resident in the UK; who have not received a positive HIV diagnosis, will be recruited. This may include transgender populations who are eligible and are under researched.  A Facebook advertisement tool will be utilised to recruit participants using digital media (www.facebook.com/business). Facebook is the biggest social media for networking and information. Every account holder has their own ‘wall’, which presents information about members of its networking groups. The Facebook advertisement facilitates the distribution of targeted adverts on an individual’s wall. For example, Facebook allows businesses to create various adverts on their products so they can be viewed by certain types of Facebook members, such as self-declared men who have interest in men. The main aim of the Facebook advertisement is to drive online sales, promote websites and mobile applications, and raise brand awareness. Facebook has also been utilised to recruit survey participants in previous studies (Nelson et al., 2014; Zhang et al., 2015). MSM are considered a ‘hard-to-reach’ population, due to difficulties with disclosure and openness of sexual orientation, so the online recruitment method would allow access to men who might not access gay-oriented facilities such as sexual health clinic. Also, this method will not require face-to-face recruitment offering confidentiality and privacy.  Our study advert will only be distributed to men who publically self-declared to be interested in men on Facebook, hence all users are already aware of their sexual orientation. The study advert will also specify that we are looking to recruit men who are sexually attracted to men and their participation will be voluntary. Once, interested advert viewers click on the link provided, they will be directed to an online survey (hosted by SurveyMonkey.com) and given more details about the study and an option to consent. Potential participants will need to indicate if they understood the study and whether they are willing to take part by ticking a box on an online survey. Participants that do not tick the required box will not be able to complete the survey.  **Sample size**  This is not possible to state in advance of developing the instrument. However, it is generally accepted that DCE studies should recruit around 300 participants [[29](#_ENREF_29)], we will aim to recruit at least this number, and is a realistic figure using Facebook.  **Analysis plan**  The data will be analysing using conditional logistic regression models and displayed as odds ratios. However, as the results represent an average response, we will also undertake latent class modelling to see if factors such as age are associated with preferences.  **Project timetable**  Data collection will be from the time ethical approval is granted up to the end of December. The questionnaire (in two blocks) has already been development. The analysis code has already been developed. An additional two months will then be needed to analyse the data and write up the manuscript.  **Ethical Approval**  Ethical approval will be sought from both Brighton and Sussex Medical School, where the PI is based, and LSHTM. |

**REFERENCES**

[1. Public Health England.](1.Public Health England. HIV in the United Kingdom: 2014 report. 2014.2.Public Health England. HIV new diagnosis, treatment and care: 2015 report.3.Williamson LM, Dodds JP, Mercey DE, et al. Sexual risk behaviour and knowledge of HIV status among community samples of gay men in the UK. Sexual risk behaviour and knowledge of HIV status among community samples of gay men in the UK 2008;22:1063-70.4.Holt M, Rawstorne P, Wilkinson J, et al. HIV testing, gay community involvement and internet use: social and behavioural correlates of HIV testing among Australian men who have sex with men. HIV testing, gay community involvement and internet use: social and behavioural correlates of HIV testing among Australian men who have sex with men 2012;16:13-22.5.Zablotska I, Holt M, de Wit J, et al. Gay men who are not getting tested for HIV. Gay men who are not getting tested for HIV 2012;16:1887-94.6.Witzel TC, Melendez-Torres GJ, Hickson F, et al. HIV testing history and preferences for future tests among gay men, bisexual men and other MSM in England. HIV testing history and preferences for future tests among gay men, bisexual men and other MSM in England In submission.7.UNSW Australia. 2015. Annual report of trends in behaviour 2015. HIV/AIDS, hepatitis and sexually transmissible infections in Australia [Online]. Available: https://csrh.arts.unsw.edu.au/media/CSRHFile/CSRH_Annual_Report_of_Trends_in_Behaviour_2015.pdf.8.Phillips AN, Cambiano V, Miners A, et al. Potential impact on HIV incidence of higher HIV testing rates and earlier antiretroviral therapy initiation in MSM. Potential impact on HIV incidence of higher HIV testing rates and earlier antiretroviral therapy initiation in MSM 2015;29:1855-62.9.Nakagawa F, Lodwick RK, Smith CJ, et al. Projected life expectancy of people with HIV according to timing of diagnosis. Projected life expectancy of people with HIV according to timing of diagnosis 2012;26:335-43.10.Chadborn TR, Delpech VC, Sabin CA, et al. The late diagnosis and consequent short-term mortality of HIV-infected heterosexuals (England and Wales, 2000–2004). The late diagnosis and consequent short-term mortality of HIV-infected heterosexuals (England and Wales, 2000–2004) 2006;20:2371-2379.11.Miners A, Phillips A, Kreif N, et al. Health-related quality-of-life of people with HIV in the era of combination antiretroviral treatment: a cross-sectional comparison with the general population. Health-related quality-of-life of people with HIV in the era of combination antiretroviral treatment: a cross-sectional comparison with the general population 2014;1:e32–e40.12.Cohen MS, Chen YQ, McCauley M, et al. Prevention of HIV-1 Infection with Early Antiretroviral Therapy. Prevention of HIV-1 Infection with Early Antiretroviral Therapy 2011;365:493-505.13.Marks G, Crepaz N, and Janssen RS. Estimating sexual transmission of HIV from persons aware and unaware that they are infected with the virus in the USA. Estimating sexual transmission of HIV from persons aware and unaware that they are infected with the virus in the USA 2006;20:1447-50.14.Nakagawa F, Miners A, Smith CJ, et al. Projected Lifetime Healthcare Costs Associated with HIV Infection. Projected Lifetime Healthcare Costs Associated with HIV Infection 2015;10:e0125018.15.Fleishman JA, Yehia BR, Moore RD, et al. The Economic Burden of Late Entry Into Medical Care for Patients With HIV Infection. The Economic Burden of Late Entry Into Medical Care for Patients With HIV Infection 2010;48:1071-1079.16.Krentz HB and Gill MJ. The Direct Medical Costs of Late Presentation (<350/mm(3)) of HIV Infection over a 15-Year Period. The Direct Medical Costs of Late Presentation (<350/mm(3)) of HIV Infection over a 15-Year Period 2012;2012:757135.17.Krause J, Subklew-Sehume F, Kenyon C, et al. Acceptability of HIV self-testing: a systematic literature review. Acceptability of HIV self-testing: a systematic literature review 2013;13:735.18.Frye V, Wilton L, Hirshfied S, et al. \"Just Because It's Out There, People Aren't Going to Use It.\" HIV Self-Testing Among Young, Black MSM, and Transgender Women. \"Just Because It's Out There, People Aren't Going to Use It.\" HIV Self-Testing Among Young, Black MSM, and Transgender Women 2015.19.Figueroa C, Johnson C, Verster A, et al. Attitudes and Acceptability on HIV Self-testing Among Key Populations: A Literature Review. Attitudes and Acceptability on HIV Self-testing Among Key Populations: A Literature Review 2015.20.Bavinton BR, Brown G, Hurley M, et al. Which gay men would increase their frequency of HIV testing with home self-testing? Which gay men would increase their frequency of HIV testing with home self-testing? 2013;17:2084-92.21.Hensen B, Lewis JJ, Schaap A, et al. Factors associated with HIV-testing and acceptance of an offer of home-based testing by men in rural Zambia. Factors associated with HIV-testing and acceptance of an offer of home-based testing by men in rural Zambia 2015;19:492-504.22.Lee VJ, Tan SC, Earnest A, et al. User acceptability and feasibility of self-testing with HIV rapid tests. User acceptability and feasibility of self-testing with HIV rapid tests 2007;45:449-53.23.Terris-Prestholt F, Hanson K, MacPhail C, et al. How much demand for New HIV prevention technologies can we really expect? Results from a discrete choice experiment in South Africa. How much demand for New HIV prevention technologies can we really expect? Results from a discrete choice experiment in South Africa 2013;8:e83193.24.Ostermann J, Njau B, Brown DS, et al. Heterogeneous HIV Testing Preferences in an Urban Setting in Tanzania: Results from a Discrete Choice Experiment. Heterogeneous HIV Testing Preferences in an Urban Setting in Tanzania: Results from a Discrete Choice Experiment 2014;9:e92100.25.Phillips KA, Maddala T, and Johnson FR. Measuring preferences for health care interventions using conjoint analysis: an application to HIV testing. Measuring preferences for health care interventions using conjoint analysis: an application to HIV testing 2002;37:1681-1705.26.Ryan M. Discrete choice experiments in health care. BMJ 2004;328:360-361.27.Terris-Prestholt F, Quaiffe M, and Vickerman P. Parameterising user uptake in economic evaluations: the role of discrete choice experiments. Parameterising user uptake in economic evaluations: the role of discrete choice experiments In press.28.Philip P, Hickson F, Bonell C, et al. Men who have sex with men in Britain: comparison of estimates from a probability sample and community-based surveys. Men who have sex with men in Britain: comparison of estimates from a probability sample and community-based surveys In submission.29.Orme B. Sample size issues for conjoint analysis, in Getting started with conjoint analysis: strategies for product design and pricing research. 2010, Research Publishers LLC: Madison, Wisconson.) *[HIV in the United Kingdom: 2014 report](1.Public Health England. HIV in the United Kingdom: 2014 report. 2014.2.Public Health England. HIV new diagnosis, treatment and care: 2015 report.3.Williamson LM, Dodds JP, Mercey DE, et al. Sexual risk behaviour and knowledge of HIV status among community samples of gay men in the UK. Sexual risk behaviour and knowledge of HIV status among community samples of gay men in the UK 2008;22:1063-70.4.Holt M, Rawstorne P, Wilkinson J, et al. HIV testing, gay community involvement and internet use: social and behavioural correlates of HIV testing among Australian men who have sex with men. HIV testing, gay community involvement and internet use: social and behavioural correlates of HIV testing among Australian men who have sex with men 2012;16:13-22.5.Zablotska I, Holt M, de Wit J, et al. Gay men who are not getting tested for HIV. Gay men who are not getting tested for HIV 2012;16:1887-94.6.Witzel TC, Melendez-Torres GJ, Hickson F, et al. HIV testing history and preferences for future tests among gay men, bisexual men and other MSM in England. HIV testing history and preferences for future tests among gay men, bisexual men and other MSM in England In submission.7.UNSW Australia. 2015. Annual report of trends in behaviour 2015. HIV/AIDS, hepatitis and sexually transmissible infections in Australia [Online]. Available: https://csrh.arts.unsw.edu.au/media/CSRHFile/CSRH_Annual_Report_of_Trends_in_Behaviour_2015.pdf.8.Phillips AN, Cambiano V, Miners A, et al. Potential impact on HIV incidence of higher HIV testing rates and earlier antiretroviral therapy initiation in MSM. Potential impact on HIV incidence of higher HIV testing rates and earlier antiretroviral therapy initiation in MSM 2015;29:1855-62.9.Nakagawa F, Lodwick RK, Smith CJ, et al. Projected life expectancy of people with HIV according to timing of diagnosis. Projected life expectancy of people with HIV according to timing of diagnosis 2012;26:335-43.10.Chadborn TR, Delpech VC, Sabin CA, et al. The late diagnosis and consequent short-term mortality of HIV-infected heterosexuals (England and Wales, 2000–2004). The late diagnosis and consequent short-term mortality of HIV-infected heterosexuals (England and Wales, 2000–2004) 2006;20:2371-2379.11.Miners A, Phillips A, Kreif N, et al. Health-related quality-of-life of people with HIV in the era of combination antiretroviral treatment: a cross-sectional comparison with the general population. Health-related quality-of-life of people with HIV in the era of combination antiretroviral treatment: a cross-sectional comparison with the general population 2014;1:e32–e40.12.Cohen MS, Chen YQ, McCauley M, et al. Prevention of HIV-1 Infection with Early Antiretroviral Therapy. Prevention of HIV-1 Infection with Early Antiretroviral Therapy 2011;365:493-505.13.Marks G, Crepaz N, and Janssen RS. Estimating sexual transmission of HIV from persons aware and unaware that they are infected with the virus in the USA. Estimating sexual transmission of HIV from persons aware and unaware that they are infected with the virus in the USA 2006;20:1447-50.14.Nakagawa F, Miners A, Smith CJ, et al. Projected Lifetime Healthcare Costs Associated with HIV Infection. Projected Lifetime Healthcare Costs Associated with HIV Infection 2015;10:e0125018.15.Fleishman JA, Yehia BR, Moore RD, et al. The Economic Burden of Late Entry Into Medical Care for Patients With HIV Infection. The Economic Burden of Late Entry Into Medical Care for Patients With HIV Infection 2010;48:1071-1079.16.Krentz HB and Gill MJ. The Direct Medical Costs of Late Presentation (<350/mm(3)) of HIV Infection over a 15-Year Period. The Direct Medical Costs of Late Presentation (<350/mm(3)) of HIV Infection over a 15-Year Period 2012;2012:757135.17.Krause J, Subklew-Sehume F, Kenyon C, et al. Acceptability of HIV self-testing: a systematic literature review. Acceptability of HIV self-testing: a systematic literature review 2013;13:735.18.Frye V, Wilton L, Hirshfied S, et al. \"Just Because It's Out There, People Aren't Going to Use It.\" HIV Self-Testing Among Young, Black MSM, and Transgender Women. \"Just Because It's Out There, People Aren't Going to Use It.\" HIV Self-Testing Among Young, Black MSM, and Transgender Women 2015.19.Figueroa C, Johnson C, Verster A, et al. Attitudes and Acceptability on HIV Self-testing Among Key Populations: A Literature Review. Attitudes and Acceptability on HIV Self-testing Among Key Populations: A Literature Review 2015.20.Bavinton BR, Brown G, Hurley M, et al. Which gay men would increase their frequency of HIV testing with home self-testing? Which gay men would increase their frequency of HIV testing with home self-testing? 2013;17:2084-92.21.Hensen B, Lewis JJ, Schaap A, et al. Factors associated with HIV-testing and acceptance of an offer of home-based testing by men in rural Zambia. Factors associated with HIV-testing and acceptance of an offer of home-based testing by men in rural Zambia 2015;19:492-504.22.Lee VJ, Tan SC, Earnest A, et al. User acceptability and feasibility of self-testing with HIV rapid tests. User acceptability and feasibility of self-testing with HIV rapid tests 2007;45:449-53.23.Terris-Prestholt F, Hanson K, MacPhail C, et al. How much demand for New HIV prevention technologies can we really expect? Results from a discrete choice experiment in South Africa. How much demand for New HIV prevention technologies can we really expect? Results from a discrete choice experiment in South Africa 2013;8:e83193.24.Ostermann J, Njau B, Brown DS, et al. Heterogeneous HIV Testing Preferences in an Urban Setting in Tanzania: Results from a Discrete Choice Experiment. Heterogeneous HIV Testing Preferences in an Urban Setting in Tanzania: Results from a Discrete Choice Experiment 2014;9:e92100.25.Phillips KA, Maddala T, and Johnson FR. Measuring preferences for health care interventions using conjoint analysis: an application to HIV testing. Measuring preferences for health care interventions using conjoint analysis: an application to HIV testing 2002;37:1681-1705.26.Ryan M. Discrete choice experiments in health care. BMJ 2004;328:360-361.27.Terris-Prestholt F, Quaiffe M, and Vickerman P. Parameterising user uptake in economic evaluations: the role of discrete choice experiments. Parameterising user uptake in economic evaluations: the role of discrete choice experiments In press.28.Philip P, Hickson F, Bonell C, et al. Men who have sex with men in Britain: comparison of estimates from a probability sample and community-based surveys. Men who have sex with men in Britain: comparison of estimates from a probability sample and community-based surveys In submission.29.Orme B. Sample size issues for conjoint analysis, in Getting started with conjoint analysis: strategies for product design and pricing research. 2010, Research Publishers LLC: Madison, Wisconson.)*[. 2014.](1.Public Health England. HIV in the United Kingdom: 2014 report. 2014.2.Public Health England. HIV new diagnosis, treatment and care: 2015 report.3.Williamson LM, Dodds JP, Mercey DE, et al. Sexual risk behaviour and knowledge of HIV status among community samples of gay men in the UK. Sexual risk behaviour and knowledge of HIV status among community samples of gay men in the UK 2008;22:1063-70.4.Holt M, Rawstorne P, Wilkinson J, et al. HIV testing, gay community involvement and internet use: social and behavioural correlates of HIV testing among Australian men who have sex with men. HIV testing, gay community involvement and internet use: social and behavioural correlates of HIV testing among Australian men who have sex with men 2012;16:13-22.5.Zablotska I, Holt M, de Wit J, et al. Gay men who are not getting tested for HIV. Gay men who are not getting tested for HIV 2012;16:1887-94.6.Witzel TC, Melendez-Torres GJ, Hickson F, et al. HIV testing history and preferences for future tests among gay men, bisexual men and other MSM in England. HIV testing history and preferences for future tests among gay men, bisexual men and other MSM in England In submission.7.UNSW Australia. 2015. Annual report of trends in behaviour 2015. HIV/AIDS, hepatitis and sexually transmissible infections in Australia [Online]. Available: https://csrh.arts.unsw.edu.au/media/CSRHFile/CSRH_Annual_Report_of_Trends_in_Behaviour_2015.pdf.8.Phillips AN, Cambiano V, Miners A, et al. Potential impact on HIV incidence of higher HIV testing rates and earlier antiretroviral therapy initiation in MSM. Potential impact on HIV incidence of higher HIV testing rates and earlier antiretroviral therapy initiation in MSM 2015;29:1855-62.9.Nakagawa F, Lodwick RK, Smith CJ, et al. Projected life expectancy of people with HIV according to timing of diagnosis. Projected life expectancy of people with HIV according to timing of diagnosis 2012;26:335-43.10.Chadborn TR, Delpech VC, Sabin CA, et al. The late diagnosis and consequent short-term mortality of HIV-infected heterosexuals (England and Wales, 2000–2004). The late diagnosis and consequent short-term mortality of HIV-infected heterosexuals (England and Wales, 2000–2004) 2006;20:2371-2379.11.Miners A, Phillips A, Kreif N, et al. Health-related quality-of-life of people with HIV in the era of combination antiretroviral treatment: a cross-sectional comparison with the general population. Health-related quality-of-life of people with HIV in the era of combination antiretroviral treatment: a cross-sectional comparison with the general population 2014;1:e32–e40.12.Cohen MS, Chen YQ, McCauley M, et al. Prevention of HIV-1 Infection with Early Antiretroviral Therapy. Prevention of HIV-1 Infection with Early Antiretroviral Therapy 2011;365:493-505.13.Marks G, Crepaz N, and Janssen RS. Estimating sexual transmission of HIV from persons aware and unaware that they are infected with the virus in the USA. Estimating sexual transmission of HIV from persons aware and unaware that they are infected with the virus in the USA 2006;20:1447-50.14.Nakagawa F, Miners A, Smith CJ, et al. Projected Lifetime Healthcare Costs Associated with HIV Infection. Projected Lifetime Healthcare Costs Associated with HIV Infection 2015;10:e0125018.15.Fleishman JA, Yehia BR, Moore RD, et al. The Economic Burden of Late Entry Into Medical Care for Patients With HIV Infection. The Economic Burden of Late Entry Into Medical Care for Patients With HIV Infection 2010;48:1071-1079.16.Krentz HB and Gill MJ. The Direct Medical Costs of Late Presentation (<350/mm(3)) of HIV Infection over a 15-Year Period. The Direct Medical Costs of Late Presentation (<350/mm(3)) of HIV Infection over a 15-Year Period 2012;2012:757135.17.Krause J, Subklew-Sehume F, Kenyon C, et al. Acceptability of HIV self-testing: a systematic literature review. Acceptability of HIV self-testing: a systematic literature review 2013;13:735.18.Frye V, Wilton L, Hirshfied S, et al. \"Just Because It's Out There, People Aren't Going to Use It.\" HIV Self-Testing Among Young, Black MSM, and Transgender Women. \"Just Because It's Out There, People Aren't Going to Use It.\" HIV Self-Testing Among Young, Black MSM, and Transgender Women 2015.19.Figueroa C, Johnson C, Verster A, et al. Attitudes and Acceptability on HIV Self-testing Among Key Populations: A Literature Review. Attitudes and Acceptability on HIV Self-testing Among Key Populations: A Literature Review 2015.20.Bavinton BR, Brown G, Hurley M, et al. Which gay men would increase their frequency of HIV testing with home self-testing? Which gay men would increase their frequency of HIV testing with home self-testing? 2013;17:2084-92.21.Hensen B, Lewis JJ, Schaap A, et al. Factors associated with HIV-testing and acceptance of an offer of home-based testing by men in rural Zambia. Factors associated with HIV-testing and acceptance of an offer of home-based testing by men in rural Zambia 2015;19:492-504.22.Lee VJ, Tan SC, Earnest A, et al. User acceptability and feasibility of self-testing with HIV rapid tests. User acceptability and feasibility of self-testing with HIV rapid tests 2007;45:449-53.23.Terris-Prestholt F, Hanson K, MacPhail C, et al. How much demand for New HIV prevention technologies can we really expect? Results from a discrete choice experiment in South Africa. How much demand for New HIV prevention technologies can we really expect? Results from a discrete choice experiment in South Africa 2013;8:e83193.24.Ostermann J, Njau B, Brown DS, et al. Heterogeneous HIV Testing Preferences in an Urban Setting in Tanzania: Results from a Discrete Choice Experiment. Heterogeneous HIV Testing Preferences in an Urban Setting in Tanzania: Results from a Discrete Choice Experiment 2014;9:e92100.25.Phillips KA, Maddala T, and Johnson FR. Measuring preferences for health care interventions using conjoint analysis: an application to HIV testing. Measuring preferences for health care interventions using conjoint analysis: an application to HIV testing 2002;37:1681-1705.26.Ryan M. Discrete choice experiments in health care. BMJ 2004;328:360-361.27.Terris-Prestholt F, Quaiffe M, and Vickerman P. Parameterising user uptake in economic evaluations: the role of discrete choice experiments. Parameterising user uptake in economic evaluations: the role of discrete choice experiments In press.28.Philip P, Hickson F, Bonell C, et al. Men who have sex with men in Britain: comparison of estimates from a probability sample and community-based surveys. Men who have sex with men in Britain: comparison of estimates from a probability sample and community-based surveys In submission.29.Orme B. Sample size issues for conjoint analysis, in Getting started with conjoint analysis: strategies for product design and pricing research. 2010, Research Publishers LLC: Madison, Wisconson.)

[2. Public Health England.](1.Public Health England. HIV in the United Kingdom: 2014 report. 2014.2.Public Health England. HIV new diagnosis, treatment and care: 2015 report.3.Williamson LM, Dodds JP, Mercey DE, et al. Sexual risk behaviour and knowledge of HIV status among community samples of gay men in the UK. Sexual risk behaviour and knowledge of HIV status among community samples of gay men in the UK 2008;22:1063-70.4.Holt M, Rawstorne P, Wilkinson J, et al. HIV testing, gay community involvement and internet use: social and behavioural correlates of HIV testing among Australian men who have sex with men. HIV testing, gay community involvement and internet use: social and behavioural correlates of HIV testing among Australian men who have sex with men 2012;16:13-22.5.Zablotska I, Holt M, de Wit J, et al. Gay men who are not getting tested for HIV. Gay men who are not getting tested for HIV 2012;16:1887-94.6.Witzel TC, Melendez-Torres GJ, Hickson F, et al. HIV testing history and preferences for future tests among gay men, bisexual men and other MSM in England. HIV testing history and preferences for future tests among gay men, bisexual men and other MSM in England In submission.7.UNSW Australia. 2015. Annual report of trends in behaviour 2015. HIV/AIDS, hepatitis and sexually transmissible infections in Australia [Online]. Available: https://csrh.arts.unsw.edu.au/media/CSRHFile/CSRH_Annual_Report_of_Trends_in_Behaviour_2015.pdf.8.Phillips AN, Cambiano V, Miners A, et al. Potential impact on HIV incidence of higher HIV testing rates and earlier antiretroviral therapy initiation in MSM. Potential impact on HIV incidence of higher HIV testing rates and earlier antiretroviral therapy initiation in MSM 2015;29:1855-62.9.Nakagawa F, Lodwick RK, Smith CJ, et al. Projected life expectancy of people with HIV according to timing of diagnosis. Projected life expectancy of people with HIV according to timing of diagnosis 2012;26:335-43.10.Chadborn TR, Delpech VC, Sabin CA, et al. The late diagnosis and consequent short-term mortality of HIV-infected heterosexuals (England and Wales, 2000–2004). The late diagnosis and consequent short-term mortality of HIV-infected heterosexuals (England and Wales, 2000–2004) 2006;20:2371-2379.11.Miners A, Phillips A, Kreif N, et al. Health-related quality-of-life of people with HIV in the era of combination antiretroviral treatment: a cross-sectional comparison with the general population. Health-related quality-of-life of people with HIV in the era of combination antiretroviral treatment: a cross-sectional comparison with the general population 2014;1:e32–e40.12.Cohen MS, Chen YQ, McCauley M, et al. Prevention of HIV-1 Infection with Early Antiretroviral Therapy. Prevention of HIV-1 Infection with Early Antiretroviral Therapy 2011;365:493-505.13.Marks G, Crepaz N, and Janssen RS. Estimating sexual transmission of HIV from persons aware and unaware that they are infected with the virus in the USA. Estimating sexual transmission of HIV from persons aware and unaware that they are infected with the virus in the USA 2006;20:1447-50.14.Nakagawa F, Miners A, Smith CJ, et al. Projected Lifetime Healthcare Costs Associated with HIV Infection. Projected Lifetime Healthcare Costs Associated with HIV Infection 2015;10:e0125018.15.Fleishman JA, Yehia BR, Moore RD, et al. The Economic Burden of Late Entry Into Medical Care for Patients With HIV Infection. The Economic Burden of Late Entry Into Medical Care for Patients With HIV Infection 2010;48:1071-1079.16.Krentz HB and Gill MJ. The Direct Medical Costs of Late Presentation (<350/mm(3)) of HIV Infection over a 15-Year Period. The Direct Medical Costs of Late Presentation (<350/mm(3)) of HIV Infection over a 15-Year Period 2012;2012:757135.17.Krause J, Subklew-Sehume F, Kenyon C, et al. Acceptability of HIV self-testing: a systematic literature review. Acceptability of HIV self-testing: a systematic literature review 2013;13:735.18.Frye V, Wilton L, Hirshfied S, et al. \"Just Because It's Out There, People Aren't Going to Use It.\" HIV Self-Testing Among Young, Black MSM, and Transgender Women. \"Just Because It's Out There, People Aren't Going to Use It.\" HIV Self-Testing Among Young, Black MSM, and Transgender Women 2015.19.Figueroa C, Johnson C, Verster A, et al. Attitudes and Acceptability on HIV Self-testing Among Key Populations: A Literature Review. Attitudes and Acceptability on HIV Self-testing Among Key Populations: A Literature Review 2015.20.Bavinton BR, Brown G, Hurley M, et al. Which gay men would increase their frequency of HIV testing with home self-testing? Which gay men would increase their frequency of HIV testing with home self-testing? 2013;17:2084-92.21.Hensen B, Lewis JJ, Schaap A, et al. Factors associated with HIV-testing and acceptance of an offer of home-based testing by men in rural Zambia. Factors associated with HIV-testing and acceptance of an offer of home-based testing by men in rural Zambia 2015;19:492-504.22.Lee VJ, Tan SC, Earnest A, et al. User acceptability and feasibility of self-testing with HIV rapid tests. User acceptability and feasibility of self-testing with HIV rapid tests 2007;45:449-53.23.Terris-Prestholt F, Hanson K, MacPhail C, et al. How much demand for New HIV prevention technologies can we really expect? Results from a discrete choice experiment in South Africa. How much demand for New HIV prevention technologies can we really expect? Results from a discrete choice experiment in South Africa 2013;8:e83193.24.Ostermann J, Njau B, Brown DS, et al. Heterogeneous HIV Testing Preferences in an Urban Setting in Tanzania: Results from a Discrete Choice Experiment. Heterogeneous HIV Testing Preferences in an Urban Setting in Tanzania: Results from a Discrete Choice Experiment 2014;9:e92100.25.Phillips KA, Maddala T, and Johnson FR. Measuring preferences for health care interventions using conjoint analysis: an application to HIV testing. Measuring preferences for health care interventions using conjoint analysis: an application to HIV testing 2002;37:1681-1705.26.Ryan M. Discrete choice experiments in health care. BMJ 2004;328:360-361.27.Terris-Prestholt F, Quaiffe M, and Vickerman P. Parameterising user uptake in economic evaluations: the role of discrete choice experiments. Parameterising user uptake in economic evaluations: the role of discrete choice experiments In press.28.Philip P, Hickson F, Bonell C, et al. Men who have sex with men in Britain: comparison of estimates from a probability sample and community-based surveys. Men who have sex with men in Britain: comparison of estimates from a probability sample and community-based surveys In submission.29.Orme B. Sample size issues for conjoint analysis, in Getting started with conjoint analysis: strategies for product design and pricing research. 2010, Research Publishers LLC: Madison, Wisconson.) *[HIV new diagnosis, treatment and care: 2015 report](1.Public Health England. HIV in the United Kingdom: 2014 report. 2014.2.Public Health England. HIV new diagnosis, treatment and care: 2015 report.3.Williamson LM, Dodds JP, Mercey DE, et al. Sexual risk behaviour and knowledge of HIV status among community samples of gay men in the UK. Sexual risk behaviour and knowledge of HIV status among community samples of gay men in the UK 2008;22:1063-70.4.Holt M, Rawstorne P, Wilkinson J, et al. HIV testing, gay community involvement and internet use: social and behavioural correlates of HIV testing among Australian men who have sex with men. HIV testing, gay community involvement and internet use: social and behavioural correlates of HIV testing among Australian men who have sex with men 2012;16:13-22.5.Zablotska I, Holt M, de Wit J, et al. Gay men who are not getting tested for HIV. Gay men who are not getting tested for HIV 2012;16:1887-94.6.Witzel TC, Melendez-Torres GJ, Hickson F, et al. HIV testing history and preferences for future tests among gay men, bisexual men and other MSM in England. HIV testing history and preferences for future tests among gay men, bisexual men and other MSM in England In submission.7.UNSW Australia. 2015. Annual report of trends in behaviour 2015. HIV/AIDS, hepatitis and sexually transmissible infections in Australia [Online]. Available: https://csrh.arts.unsw.edu.au/media/CSRHFile/CSRH_Annual_Report_of_Trends_in_Behaviour_2015.pdf.8.Phillips AN, Cambiano V, Miners A, et al. Potential impact on HIV incidence of higher HIV testing rates and earlier antiretroviral therapy initiation in MSM. Potential impact on HIV incidence of higher HIV testing rates and earlier antiretroviral therapy initiation in MSM 2015;29:1855-62.9.Nakagawa F, Lodwick RK, Smith CJ, et al. Projected life expectancy of people with HIV according to timing of diagnosis. Projected life expectancy of people with HIV according to timing of diagnosis 2012;26:335-43.10.Chadborn TR, Delpech VC, Sabin CA, et al. The late diagnosis and consequent short-term mortality of HIV-infected heterosexuals (England and Wales, 2000–2004). The late diagnosis and consequent short-term mortality of HIV-infected heterosexuals (England and Wales, 2000–2004) 2006;20:2371-2379.11.Miners A, Phillips A, Kreif N, et al. Health-related quality-of-life of people with HIV in the era of combination antiretroviral treatment: a cross-sectional comparison with the general population. Health-related quality-of-life of people with HIV in the era of combination antiretroviral treatment: a cross-sectional comparison with the general population 2014;1:e32–e40.12.Cohen MS, Chen YQ, McCauley M, et al. Prevention of HIV-1 Infection with Early Antiretroviral Therapy. Prevention of HIV-1 Infection with Early Antiretroviral Therapy 2011;365:493-505.13.Marks G, Crepaz N, and Janssen RS. Estimating sexual transmission of HIV from persons aware and unaware that they are infected with the virus in the USA. Estimating sexual transmission of HIV from persons aware and unaware that they are infected with the virus in the USA 2006;20:1447-50.14.Nakagawa F, Miners A, Smith CJ, et al. Projected Lifetime Healthcare Costs Associated with HIV Infection. Projected Lifetime Healthcare Costs Associated with HIV Infection 2015;10:e0125018.15.Fleishman JA, Yehia BR, Moore RD, et al. The Economic Burden of Late Entry Into Medical Care for Patients With HIV Infection. The Economic Burden of Late Entry Into Medical Care for Patients With HIV Infection 2010;48:1071-1079.16.Krentz HB and Gill MJ. The Direct Medical Costs of Late Presentation (<350/mm(3)) of HIV Infection over a 15-Year Period. The Direct Medical Costs of Late Presentation (<350/mm(3)) of HIV Infection over a 15-Year Period 2012;2012:757135.17.Krause J, Subklew-Sehume F, Kenyon C, et al. Acceptability of HIV self-testing: a systematic literature review. Acceptability of HIV self-testing: a systematic literature review 2013;13:735.18.Frye V, Wilton L, Hirshfied S, et al. \"Just Because It's Out There, People Aren't Going to Use It.\" HIV Self-Testing Among Young, Black MSM, and Transgender Women. \"Just Because It's Out There, People Aren't Going to Use It.\" HIV Self-Testing Among Young, Black MSM, and Transgender Women 2015.19.Figueroa C, Johnson C, Verster A, et al. Attitudes and Acceptability on HIV Self-testing Among Key Populations: A Literature Review. Attitudes and Acceptability on HIV Self-testing Among Key Populations: A Literature Review 2015.20.Bavinton BR, Brown G, Hurley M, et al. Which gay men would increase their frequency of HIV testing with home self-testing? Which gay men would increase their frequency of HIV testing with home self-testing? 2013;17:2084-92.21.Hensen B, Lewis JJ, Schaap A, et al. Factors associated with HIV-testing and acceptance of an offer of home-based testing by men in rural Zambia. Factors associated with HIV-testing and acceptance of an offer of home-based testing by men in rural Zambia 2015;19:492-504.22.Lee VJ, Tan SC, Earnest A, et al. User acceptability and feasibility of self-testing with HIV rapid tests. User acceptability and feasibility of self-testing with HIV rapid tests 2007;45:449-53.23.Terris-Prestholt F, Hanson K, MacPhail C, et al. How much demand for New HIV prevention technologies can we really expect? Results from a discrete choice experiment in South Africa. How much demand for New HIV prevention technologies can we really expect? Results from a discrete choice experiment in South Africa 2013;8:e83193.24.Ostermann J, Njau B, Brown DS, et al. Heterogeneous HIV Testing Preferences in an Urban Setting in Tanzania: Results from a Discrete Choice Experiment. Heterogeneous HIV Testing Preferences in an Urban Setting in Tanzania: Results from a Discrete Choice Experiment 2014;9:e92100.25.Phillips KA, Maddala T, and Johnson FR. Measuring preferences for health care interventions using conjoint analysis: an application to HIV testing. Measuring preferences for health care interventions using conjoint analysis: an application to HIV testing 2002;37:1681-1705.26.Ryan M. Discrete choice experiments in health care. BMJ 2004;328:360-361.27.Terris-Prestholt F, Quaiffe M, and Vickerman P. Parameterising user uptake in economic evaluations: the role of discrete choice experiments. Parameterising user uptake in economic evaluations: the role of discrete choice experiments In press.28.Philip P, Hickson F, Bonell C, et al. Men who have sex with men in Britain: comparison of estimates from a probability sample and community-based surveys. Men who have sex with men in Britain: comparison of estimates from a probability sample and community-based surveys In submission.29.Orme B. Sample size issues for conjoint analysis, in Getting started with conjoint analysis: strategies for product design and pricing research. 2010, Research Publishers LLC: Madison, Wisconson.)*[.](1.Public Health England. HIV in the United Kingdom: 2014 report. 2014.2.Public Health England. HIV new diagnosis, treatment and care: 2015 report.3.Williamson LM, Dodds JP, Mercey DE, et al. Sexual risk behaviour and knowledge of HIV status among community samples of gay men in the UK. Sexual risk behaviour and knowledge of HIV status among community samples of gay men in the UK 2008;22:1063-70.4.Holt M, Rawstorne P, Wilkinson J, et al. HIV testing, gay community involvement and internet use: social and behavioural correlates of HIV testing among Australian men who have sex with men. HIV testing, gay community involvement and internet use: social and behavioural correlates of HIV testing among Australian men who have sex with men 2012;16:13-22.5.Zablotska I, Holt M, de Wit J, et al. Gay men who are not getting tested for HIV. Gay men who are not getting tested for HIV 2012;16:1887-94.6.Witzel TC, Melendez-Torres GJ, Hickson F, et al. HIV testing history and preferences for future tests among gay men, bisexual men and other MSM in England. HIV testing history and preferences for future tests among gay men, bisexual men and other MSM in England In submission.7.UNSW Australia. 2015. Annual report of trends in behaviour 2015. HIV/AIDS, hepatitis and sexually transmissible infections in Australia [Online]. Available: https://csrh.arts.unsw.edu.au/media/CSRHFile/CSRH_Annual_Report_of_Trends_in_Behaviour_2015.pdf.8.Phillips AN, Cambiano V, Miners A, et al. Potential impact on HIV incidence of higher HIV testing rates and earlier antiretroviral therapy initiation in MSM. Potential impact on HIV incidence of higher HIV testing rates and earlier antiretroviral therapy initiation in MSM 2015;29:1855-62.9.Nakagawa F, Lodwick RK, Smith CJ, et al. Projected life expectancy of people with HIV according to timing of diagnosis. Projected life expectancy of people with HIV according to timing of diagnosis 2012;26:335-43.10.Chadborn TR, Delpech VC, Sabin CA, et al. The late diagnosis and consequent short-term mortality of HIV-infected heterosexuals (England and Wales, 2000–2004). The late diagnosis and consequent short-term mortality of HIV-infected heterosexuals (England and Wales, 2000–2004) 2006;20:2371-2379.11.Miners A, Phillips A, Kreif N, et al. Health-related quality-of-life of people with HIV in the era of combination antiretroviral treatment: a cross-sectional comparison with the general population. Health-related quality-of-life of people with HIV in the era of combination antiretroviral treatment: a cross-sectional comparison with the general population 2014;1:e32–e40.12.Cohen MS, Chen YQ, McCauley M, et al. Prevention of HIV-1 Infection with Early Antiretroviral Therapy. Prevention of HIV-1 Infection with Early Antiretroviral Therapy 2011;365:493-505.13.Marks G, Crepaz N, and Janssen RS. Estimating sexual transmission of HIV from persons aware and unaware that they are infected with the virus in the USA. Estimating sexual transmission of HIV from persons aware and unaware that they are infected with the virus in the USA 2006;20:1447-50.14.Nakagawa F, Miners A, Smith CJ, et al. Projected Lifetime Healthcare Costs Associated with HIV Infection. Projected Lifetime Healthcare Costs Associated with HIV Infection 2015;10:e0125018.15.Fleishman JA, Yehia BR, Moore RD, et al. The Economic Burden of Late Entry Into Medical Care for Patients With HIV Infection. The Economic Burden of Late Entry Into Medical Care for Patients With HIV Infection 2010;48:1071-1079.16.Krentz HB and Gill MJ. The Direct Medical Costs of Late Presentation (<350/mm(3)) of HIV Infection over a 15-Year Period. The Direct Medical Costs of Late Presentation (<350/mm(3)) of HIV Infection over a 15-Year Period 2012;2012:757135.17.Krause J, Subklew-Sehume F, Kenyon C, et al. Acceptability of HIV self-testing: a systematic literature review. Acceptability of HIV self-testing: a systematic literature review 2013;13:735.18.Frye V, Wilton L, Hirshfied S, et al. \"Just Because It's Out There, People Aren't Going to Use It.\" HIV Self-Testing Among Young, Black MSM, and Transgender Women. \"Just Because It's Out There, People Aren't Going to Use It.\" HIV Self-Testing Among Young, Black MSM, and Transgender Women 2015.19.Figueroa C, Johnson C, Verster A, et al. Attitudes and Acceptability on HIV Self-testing Among Key Populations: A Literature Review. Attitudes and Acceptability on HIV Self-testing Among Key Populations: A Literature Review 2015.20.Bavinton BR, Brown G, Hurley M, et al. Which gay men would increase their frequency of HIV testing with home self-testing? Which gay men would increase their frequency of HIV testing with home self-testing? 2013;17:2084-92.21.Hensen B, Lewis JJ, Schaap A, et al. Factors associated with HIV-testing and acceptance of an offer of home-based testing by men in rural Zambia. Factors associated with HIV-testing and acceptance of an offer of home-based testing by men in rural Zambia 2015;19:492-504.22.Lee VJ, Tan SC, Earnest A, et al. User acceptability and feasibility of self-testing with HIV rapid tests. User acceptability and feasibility of self-testing with HIV rapid tests 2007;45:449-53.23.Terris-Prestholt F, Hanson K, MacPhail C, et al. How much demand for New HIV prevention technologies can we really expect? Results from a discrete choice experiment in South Africa. How much demand for New HIV prevention technologies can we really expect? Results from a discrete choice experiment in South Africa 2013;8:e83193.24.Ostermann J, Njau B, Brown DS, et al. Heterogeneous HIV Testing Preferences in an Urban Setting in Tanzania: Results from a Discrete Choice Experiment. Heterogeneous HIV Testing Preferences in an Urban Setting in Tanzania: Results from a Discrete Choice Experiment 2014;9:e92100.25.Phillips KA, Maddala T, and Johnson FR. Measuring preferences for health care interventions using conjoint analysis: an application to HIV testing. Measuring preferences for health care interventions using conjoint analysis: an application to HIV testing 2002;37:1681-1705.26.Ryan M. Discrete choice experiments in health care. BMJ 2004;328:360-361.27.Terris-Prestholt F, Quaiffe M, and Vickerman P. Parameterising user uptake in economic evaluations: the role of discrete choice experiments. Parameterising user uptake in economic evaluations: the role of discrete choice experiments In press.28.Philip P, Hickson F, Bonell C, et al. Men who have sex with men in Britain: comparison of estimates from a probability sample and community-based surveys. Men who have sex with men in Britain: comparison of estimates from a probability sample and community-based surveys In submission.29.Orme B. Sample size issues for conjoint analysis, in Getting started with conjoint analysis: strategies for product design and pricing research. 2010, Research Publishers LLC: Madison, Wisconson.)

[3. Williamson LM, Dodds JP, Mercey DE, et al. Sexual risk behaviour and knowledge of HIV status among community samples of gay men in the UK](1.Public Health England. HIV in the United Kingdom: 2014 report. 2014.2.Public Health England. HIV new diagnosis, treatment and care: 2015 report.3.Williamson LM, Dodds JP, Mercey DE, et al. Sexual risk behaviour and knowledge of HIV status among community samples of gay men in the UK. Sexual risk behaviour and knowledge of HIV status among community samples of gay men in the UK 2008;22:1063-70.4.Holt M, Rawstorne P, Wilkinson J, et al. HIV testing, gay community involvement and internet use: social and behavioural correlates of HIV testing among Australian men who have sex with men. HIV testing, gay community involvement and internet use: social and behavioural correlates of HIV testing among Australian men who have sex with men 2012;16:13-22.5.Zablotska I, Holt M, de Wit J, et al. Gay men who are not getting tested for HIV. Gay men who are not getting tested for HIV 2012;16:1887-94.6.Witzel TC, Melendez-Torres GJ, Hickson F, et al. HIV testing history and preferences for future tests among gay men, bisexual men and other MSM in England. HIV testing history and preferences for future tests among gay men, bisexual men and other MSM in England In submission.7.UNSW Australia. 2015. Annual report of trends in behaviour 2015. HIV/AIDS, hepatitis and sexually transmissible infections in Australia [Online]. Available: https://csrh.arts.unsw.edu.au/media/CSRHFile/CSRH_Annual_Report_of_Trends_in_Behaviour_2015.pdf.8.Phillips AN, Cambiano V, Miners A, et al. Potential impact on HIV incidence of higher HIV testing rates and earlier antiretroviral therapy initiation in MSM. Potential impact on HIV incidence of higher HIV testing rates and earlier antiretroviral therapy initiation in MSM 2015;29:1855-62.9.Nakagawa F, Lodwick RK, Smith CJ, et al. Projected life expectancy of people with HIV according to timing of diagnosis. Projected life expectancy of people with HIV according to timing of diagnosis 2012;26:335-43.10.Chadborn TR, Delpech VC, Sabin CA, et al. The late diagnosis and consequent short-term mortality of HIV-infected heterosexuals (England and Wales, 2000–2004). The late diagnosis and consequent short-term mortality of HIV-infected heterosexuals (England and Wales, 2000–2004) 2006;20:2371-2379.11.Miners A, Phillips A, Kreif N, et al. Health-related quality-of-life of people with HIV in the era of combination antiretroviral treatment: a cross-sectional comparison with the general population. Health-related quality-of-life of people with HIV in the era of combination antiretroviral treatment: a cross-sectional comparison with the general population 2014;1:e32–e40.12.Cohen MS, Chen YQ, McCauley M, et al. Prevention of HIV-1 Infection with Early Antiretroviral Therapy. Prevention of HIV-1 Infection with Early Antiretroviral Therapy 2011;365:493-505.13.Marks G, Crepaz N, and Janssen RS. Estimating sexual transmission of HIV from persons aware and unaware that they are infected with the virus in the USA. Estimating sexual transmission of HIV from persons aware and unaware that they are infected with the virus in the USA 2006;20:1447-50.14.Nakagawa F, Miners A, Smith CJ, et al. Projected Lifetime Healthcare Costs Associated with HIV Infection. Projected Lifetime Healthcare Costs Associated with HIV Infection 2015;10:e0125018.15.Fleishman JA, Yehia BR, Moore RD, et al. The Economic Burden of Late Entry Into Medical Care for Patients With HIV Infection. The Economic Burden of Late Entry Into Medical Care for Patients With HIV Infection 2010;48:1071-1079.16.Krentz HB and Gill MJ. The Direct Medical Costs of Late Presentation (<350/mm(3)) of HIV Infection over a 15-Year Period. The Direct Medical Costs of Late Presentation (<350/mm(3)) of HIV Infection over a 15-Year Period 2012;2012:757135.17.Krause J, Subklew-Sehume F, Kenyon C, et al. Acceptability of HIV self-testing: a systematic literature review. Acceptability of HIV self-testing: a systematic literature review 2013;13:735.18.Frye V, Wilton L, Hirshfied S, et al. \"Just Because It's Out There, People Aren't Going to Use It.\" HIV Self-Testing Among Young, Black MSM, and Transgender Women. \"Just Because It's Out There, People Aren't Going to Use It.\" HIV Self-Testing Among Young, Black MSM, and Transgender Women 2015.19.Figueroa C, Johnson C, Verster A, et al. Attitudes and Acceptability on HIV Self-testing Among Key Populations: A Literature Review. Attitudes and Acceptability on HIV Self-testing Among Key Populations: A Literature Review 2015.20.Bavinton BR, Brown G, Hurley M, et al. Which gay men would increase their frequency of HIV testing with home self-testing? Which gay men would increase their frequency of HIV testing with home self-testing? 2013;17:2084-92.21.Hensen B, Lewis JJ, Schaap A, et al. Factors associated with HIV-testing and acceptance of an offer of home-based testing by men in rural Zambia. Factors associated with HIV-testing and acceptance of an offer of home-based testing by men in rural Zambia 2015;19:492-504.22.Lee VJ, Tan SC, Earnest A, et al. User acceptability and feasibility of self-testing with HIV rapid tests. User acceptability and feasibility of self-testing with HIV rapid tests 2007;45:449-53.23.Terris-Prestholt F, Hanson K, MacPhail C, et al. How much demand for New HIV prevention technologies can we really expect? Results from a discrete choice experiment in South Africa. How much demand for New HIV prevention technologies can we really expect? Results from a discrete choice experiment in South Africa 2013;8:e83193.24.Ostermann J, Njau B, Brown DS, et al. Heterogeneous HIV Testing Preferences in an Urban Setting in Tanzania: Results from a Discrete Choice Experiment. Heterogeneous HIV Testing Preferences in an Urban Setting in Tanzania: Results from a Discrete Choice Experiment 2014;9:e92100.25.Phillips KA, Maddala T, and Johnson FR. Measuring preferences for health care interventions using conjoint analysis: an application to HIV testing. Measuring preferences for health care interventions using conjoint analysis: an application to HIV testing 2002;37:1681-1705.26.Ryan M. Discrete choice experiments in health care. BMJ 2004;328:360-361.27.Terris-Prestholt F, Quaiffe M, and Vickerman P. Parameterising user uptake in economic evaluations: the role of discrete choice experiments. Parameterising user uptake in economic evaluations: the role of discrete choice experiments In press.28.Philip P, Hickson F, Bonell C, et al. Men who have sex with men in Britain: comparison of estimates from a probability sample and community-based surveys. Men who have sex with men in Britain: comparison of estimates from a probability sample and community-based surveys In submission.29.Orme B. Sample size issues for conjoint analysis, in Getting started with conjoint analysis: strategies for product design and pricing research. 2010, Research Publishers LLC: Madison, Wisconson.)*[.](1.Public Health England. HIV in the United Kingdom: 2014 report. 2014.2.Public Health England. HIV new diagnosis, treatment and care: 2015 report.3.Williamson LM, Dodds JP, Mercey DE, et al. Sexual risk behaviour and knowledge of HIV status among community samples of gay men in the UK. Sexual risk behaviour and knowledge of HIV status among community samples of gay men in the UK 2008;22:1063-70.4.Holt M, Rawstorne P, Wilkinson J, et al. HIV testing, gay community involvement and internet use: social and behavioural correlates of HIV testing among Australian men who have sex with men. HIV testing, gay community involvement and internet use: social and behavioural correlates of HIV testing among Australian men who have sex with men 2012;16:13-22.5.Zablotska I, Holt M, de Wit J, et al. Gay men who are not getting tested for HIV. Gay men who are not getting tested for HIV 2012;16:1887-94.6.Witzel TC, Melendez-Torres GJ, Hickson F, et al. HIV testing history and preferences for future tests among gay men, bisexual men and other MSM in England. HIV testing history and preferences for future tests among gay men, bisexual men and other MSM in England In submission.7.UNSW Australia. 2015. Annual report of trends in behaviour 2015. HIV/AIDS, hepatitis and sexually transmissible infections in Australia [Online]. Available: https://csrh.arts.unsw.edu.au/media/CSRHFile/CSRH_Annual_Report_of_Trends_in_Behaviour_2015.pdf.8.Phillips AN, Cambiano V, Miners A, et al. Potential impact on HIV incidence of higher HIV testing rates and earlier antiretroviral therapy initiation in MSM. Potential impact on HIV incidence of higher HIV testing rates and earlier antiretroviral therapy initiation in MSM 2015;29:1855-62.9.Nakagawa F, Lodwick RK, Smith CJ, et al. Projected life expectancy of people with HIV according to timing of diagnosis. Projected life expectancy of people with HIV according to timing of diagnosis 2012;26:335-43.10.Chadborn TR, Delpech VC, Sabin CA, et al. The late diagnosis and consequent short-term mortality of HIV-infected heterosexuals (England and Wales, 2000–2004). The late diagnosis and consequent short-term mortality of HIV-infected heterosexuals (England and Wales, 2000–2004) 2006;20:2371-2379.11.Miners A, Phillips A, Kreif N, et al. Health-related quality-of-life of people with HIV in the era of combination antiretroviral treatment: a cross-sectional comparison with the general population. Health-related quality-of-life of people with HIV in the era of combination antiretroviral treatment: a cross-sectional comparison with the general population 2014;1:e32–e40.12.Cohen MS, Chen YQ, McCauley M, et al. Prevention of HIV-1 Infection with Early Antiretroviral Therapy. Prevention of HIV-1 Infection with Early Antiretroviral Therapy 2011;365:493-505.13.Marks G, Crepaz N, and Janssen RS. Estimating sexual transmission of HIV from persons aware and unaware that they are infected with the virus in the USA. Estimating sexual transmission of HIV from persons aware and unaware that they are infected with the virus in the USA 2006;20:1447-50.14.Nakagawa F, Miners A, Smith CJ, et al. Projected Lifetime Healthcare Costs Associated with HIV Infection. Projected Lifetime Healthcare Costs Associated with HIV Infection 2015;10:e0125018.15.Fleishman JA, Yehia BR, Moore RD, et al. The Economic Burden of Late Entry Into Medical Care for Patients With HIV Infection. The Economic Burden of Late Entry Into Medical Care for Patients With HIV Infection 2010;48:1071-1079.16.Krentz HB and Gill MJ. The Direct Medical Costs of Late Presentation (<350/mm(3)) of HIV Infection over a 15-Year Period. The Direct Medical Costs of Late Presentation (<350/mm(3)) of HIV Infection over a 15-Year Period 2012;2012:757135.17.Krause J, Subklew-Sehume F, Kenyon C, et al. Acceptability of HIV self-testing: a systematic literature review. Acceptability of HIV self-testing: a systematic literature review 2013;13:735.18.Frye V, Wilton L, Hirshfied S, et al. \"Just Because It's Out There, People Aren't Going to Use It.\" HIV Self-Testing Among Young, Black MSM, and Transgender Women. \"Just Because It's Out There, People Aren't Going to Use It.\" HIV Self-Testing Among Young, Black MSM, and Transgender Women 2015.19.Figueroa C, Johnson C, Verster A, et al. Attitudes and Acceptability on HIV Self-testing Among Key Populations: A Literature Review. Attitudes and Acceptability on HIV Self-testing Among Key Populations: A Literature Review 2015.20.Bavinton BR, Brown G, Hurley M, et al. Which gay men would increase their frequency of HIV testing with home self-testing? Which gay men would increase their frequency of HIV testing with home self-testing? 2013;17:2084-92.21.Hensen B, Lewis JJ, Schaap A, et al. Factors associated with HIV-testing and acceptance of an offer of home-based testing by men in rural Zambia. Factors associated with HIV-testing and acceptance of an offer of home-based testing by men in rural Zambia 2015;19:492-504.22.Lee VJ, Tan SC, Earnest A, et al. User acceptability and feasibility of self-testing with HIV rapid tests. User acceptability and feasibility of self-testing with HIV rapid tests 2007;45:449-53.23.Terris-Prestholt F, Hanson K, MacPhail C, et al. How much demand for New HIV prevention technologies can we really expect? Results from a discrete choice experiment in South Africa. How much demand for New HIV prevention technologies can we really expect? Results from a discrete choice experiment in South Africa 2013;8:e83193.24.Ostermann J, Njau B, Brown DS, et al. Heterogeneous HIV Testing Preferences in an Urban Setting in Tanzania: Results from a Discrete Choice Experiment. Heterogeneous HIV Testing Preferences in an Urban Setting in Tanzania: Results from a Discrete Choice Experiment 2014;9:e92100.25.Phillips KA, Maddala T, and Johnson FR. Measuring preferences for health care interventions using conjoint analysis: an application to HIV testing. Measuring preferences for health care interventions using conjoint analysis: an application to HIV testing 2002;37:1681-1705.26.Ryan M. Discrete choice experiments in health care. BMJ 2004;328:360-361.27.Terris-Prestholt F, Quaiffe M, and Vickerman P. Parameterising user uptake in economic evaluations: the role of discrete choice experiments. Parameterising user uptake in economic evaluations: the role of discrete choice experiments In press.28.Philip P, Hickson F, Bonell C, et al. Men who have sex with men in Britain: comparison of estimates from a probability sample and community-based surveys. Men who have sex with men in Britain: comparison of estimates from a probability sample and community-based surveys In submission.29.Orme B. Sample size issues for conjoint analysis, in Getting started with conjoint analysis: strategies for product design and pricing research. 2010, Research Publishers LLC: Madison, Wisconson.)**[Sexual risk behaviour and knowledge of HIV status among community samples of gay men in the UK](1.Public Health England. HIV in the United Kingdom: 2014 report. 2014.2.Public Health England. HIV new diagnosis, treatment and care: 2015 report.3.Williamson LM, Dodds JP, Mercey DE, et al. Sexual risk behaviour and knowledge of HIV status among community samples of gay men in the UK. Sexual risk behaviour and knowledge of HIV status among community samples of gay men in the UK 2008;22:1063-70.4.Holt M, Rawstorne P, Wilkinson J, et al. HIV testing, gay community involvement and internet use: social and behavioural correlates of HIV testing among Australian men who have sex with men. HIV testing, gay community involvement and internet use: social and behavioural correlates of HIV testing among Australian men who have sex with men 2012;16:13-22.5.Zablotska I, Holt M, de Wit J, et al. Gay men who are not getting tested for HIV. Gay men who are not getting tested for HIV 2012;16:1887-94.6.Witzel TC, Melendez-Torres GJ, Hickson F, et al. HIV testing history and preferences for future tests among gay men, bisexual men and other MSM in England. HIV testing history and preferences for future tests among gay men, bisexual men and other MSM in England In submission.7.UNSW Australia. 2015. Annual report of trends in behaviour 2015. HIV/AIDS, hepatitis and sexually transmissible infections in Australia [Online]. Available: https://csrh.arts.unsw.edu.au/media/CSRHFile/CSRH_Annual_Report_of_Trends_in_Behaviour_2015.pdf.8.Phillips AN, Cambiano V, Miners A, et al. Potential impact on HIV incidence of higher HIV testing rates and earlier antiretroviral therapy initiation in MSM. Potential impact on HIV incidence of higher HIV testing rates and earlier antiretroviral therapy initiation in MSM 2015;29:1855-62.9.Nakagawa F, Lodwick RK, Smith CJ, et al. Projected life expectancy of people with HIV according to timing of diagnosis. Projected life expectancy of people with HIV according to timing of diagnosis 2012;26:335-43.10.Chadborn TR, Delpech VC, Sabin CA, et al. The late diagnosis and consequent short-term mortality of HIV-infected heterosexuals (England and Wales, 2000–2004). The late diagnosis and consequent short-term mortality of HIV-infected heterosexuals (England and Wales, 2000–2004) 2006;20:2371-2379.11.Miners A, Phillips A, Kreif N, et al. Health-related quality-of-life of people with HIV in the era of combination antiretroviral treatment: a cross-sectional comparison with the general population. Health-related quality-of-life of people with HIV in the era of combination antiretroviral treatment: a cross-sectional comparison with the general population 2014;1:e32–e40.12.Cohen MS, Chen YQ, McCauley M, et al. Prevention of HIV-1 Infection with Early Antiretroviral Therapy. Prevention of HIV-1 Infection with Early Antiretroviral Therapy 2011;365:493-505.13.Marks G, Crepaz N, and Janssen RS. Estimating sexual transmission of HIV from persons aware and unaware that they are infected with the virus in the USA. Estimating sexual transmission of HIV from persons aware and unaware that they are infected with the virus in the USA 2006;20:1447-50.14.Nakagawa F, Miners A, Smith CJ, et al. Projected Lifetime Healthcare Costs Associated with HIV Infection. Projected Lifetime Healthcare Costs Associated with HIV Infection 2015;10:e0125018.15.Fleishman JA, Yehia BR, Moore RD, et al. The Economic Burden of Late Entry Into Medical Care for Patients With HIV Infection. The Economic Burden of Late Entry Into Medical Care for Patients With HIV Infection 2010;48:1071-1079.16.Krentz HB and Gill MJ. The Direct Medical Costs of Late Presentation (<350/mm(3)) of HIV Infection over a 15-Year Period. The Direct Medical Costs of Late Presentation (<350/mm(3)) of HIV Infection over a 15-Year Period 2012;2012:757135.17.Krause J, Subklew-Sehume F, Kenyon C, et al. Acceptability of HIV self-testing: a systematic literature review. Acceptability of HIV self-testing: a systematic literature review 2013;13:735.18.Frye V, Wilton L, Hirshfied S, et al. \"Just Because It's Out There, People Aren't Going to Use It.\" HIV Self-Testing Among Young, Black MSM, and Transgender Women. \"Just Because It's Out There, People Aren't Going to Use It.\" HIV Self-Testing Among Young, Black MSM, and Transgender Women 2015.19.Figueroa C, Johnson C, Verster A, et al. Attitudes and Acceptability on HIV Self-testing Among Key Populations: A Literature Review. Attitudes and Acceptability on HIV Self-testing Among Key Populations: A Literature Review 2015.20.Bavinton BR, Brown G, Hurley M, et al. Which gay men would increase their frequency of HIV testing with home self-testing? Which gay men would increase their frequency of HIV testing with home self-testing? 2013;17:2084-92.21.Hensen B, Lewis JJ, Schaap A, et al. Factors associated with HIV-testing and acceptance of an offer of home-based testing by men in rural Zambia. Factors associated with HIV-testing and acceptance of an offer of home-based testing by men in rural Zambia 2015;19:492-504.22.Lee VJ, Tan SC, Earnest A, et al. User acceptability and feasibility of self-testing with HIV rapid tests. User acceptability and feasibility of self-testing with HIV rapid tests 2007;45:449-53.23.Terris-Prestholt F, Hanson K, MacPhail C, et al. How much demand for New HIV prevention technologies can we really expect? Results from a discrete choice experiment in South Africa. How much demand for New HIV prevention technologies can we really expect? Results from a discrete choice experiment in South Africa 2013;8:e83193.24.Ostermann J, Njau B, Brown DS, et al. Heterogeneous HIV Testing Preferences in an Urban Setting in Tanzania: Results from a Discrete Choice Experiment. Heterogeneous HIV Testing Preferences in an Urban Setting in Tanzania: Results from a Discrete Choice Experiment 2014;9:e92100.25.Phillips KA, Maddala T, and Johnson FR. Measuring preferences for health care interventions using conjoint analysis: an application to HIV testing. Measuring preferences for health care interventions using conjoint analysis: an application to HIV testing 2002;37:1681-1705.26.Ryan M. Discrete choice experiments in health care. BMJ 2004;328:360-361.27.Terris-Prestholt F, Quaiffe M, and Vickerman P. Parameterising user uptake in economic evaluations: the role of discrete choice experiments. Parameterising user uptake in economic evaluations: the role of discrete choice experiments In press.28.Philip P, Hickson F, Bonell C, et al. Men who have sex with men in Britain: comparison of estimates from a probability sample and community-based surveys. Men who have sex with men in Britain: comparison of estimates from a probability sample and community-based surveys In submission.29.Orme B. Sample size issues for conjoint analysis, in Getting started with conjoint analysis: strategies for product design and pricing research. 2010, Research Publishers LLC: Madison, Wisconson.)* [2008;](1.Public Health England. HIV in the United Kingdom: 2014 report. 2014.2.Public Health England. HIV new diagnosis, treatment and care: 2015 report.3.Williamson LM, Dodds JP, Mercey DE, et al. Sexual risk behaviour and knowledge of HIV status among community samples of gay men in the UK. Sexual risk behaviour and knowledge of HIV status among community samples of gay men in the UK 2008;22:1063-70.4.Holt M, Rawstorne P, Wilkinson J, et al. HIV testing, gay community involvement and internet use: social and behavioural correlates of HIV testing among Australian men who have sex with men. HIV testing, gay community involvement and internet use: social and behavioural correlates of HIV testing among Australian men who have sex with men 2012;16:13-22.5.Zablotska I, Holt M, de Wit J, et al. Gay men who are not getting tested for HIV. Gay men who are not getting tested for HIV 2012;16:1887-94.6.Witzel TC, Melendez-Torres GJ, Hickson F, et al. HIV testing history and preferences for future tests among gay men, bisexual men and other MSM in England. HIV testing history and preferences for future tests among gay men, bisexual men and other MSM in England In submission.7.UNSW Australia. 2015. Annual report of trends in behaviour 2015. HIV/AIDS, hepatitis and sexually transmissible infections in Australia [Online]. Available: https://csrh.arts.unsw.edu.au/media/CSRHFile/CSRH_Annual_Report_of_Trends_in_Behaviour_2015.pdf.8.Phillips AN, Cambiano V, Miners A, et al. Potential impact on HIV incidence of higher HIV testing rates and earlier antiretroviral therapy initiation in MSM. Potential impact on HIV incidence of higher HIV testing rates and earlier antiretroviral therapy initiation in MSM 2015;29:1855-62.9.Nakagawa F, Lodwick RK, Smith CJ, et al. Projected life expectancy of people with HIV according to timing of diagnosis. Projected life expectancy of people with HIV according to timing of diagnosis 2012;26:335-43.10.Chadborn TR, Delpech VC, Sabin CA, et al. The late diagnosis and consequent short-term mortality of HIV-infected heterosexuals (England and Wales, 2000–2004). The late diagnosis and consequent short-term mortality of HIV-infected heterosexuals (England and Wales, 2000–2004) 2006;20:2371-2379.11.Miners A, Phillips A, Kreif N, et al. Health-related quality-of-life of people with HIV in the era of combination antiretroviral treatment: a cross-sectional comparison with the general population. Health-related quality-of-life of people with HIV in the era of combination antiretroviral treatment: a cross-sectional comparison with the general population 2014;1:e32–e40.12.Cohen MS, Chen YQ, McCauley M, et al. Prevention of HIV-1 Infection with Early Antiretroviral Therapy. Prevention of HIV-1 Infection with Early Antiretroviral Therapy 2011;365:493-505.13.Marks G, Crepaz N, and Janssen RS. Estimating sexual transmission of HIV from persons aware and unaware that they are infected with the virus in the USA. Estimating sexual transmission of HIV from persons aware and unaware that they are infected with the virus in the USA 2006;20:1447-50.14.Nakagawa F, Miners A, Smith CJ, et al. Projected Lifetime Healthcare Costs Associated with HIV Infection. Projected Lifetime Healthcare Costs Associated with HIV Infection 2015;10:e0125018.15.Fleishman JA, Yehia BR, Moore RD, et al. The Economic Burden of Late Entry Into Medical Care for Patients With HIV Infection. The Economic Burden of Late Entry Into Medical Care for Patients With HIV Infection 2010;48:1071-1079.16.Krentz HB and Gill MJ. The Direct Medical Costs of Late Presentation (<350/mm(3)) of HIV Infection over a 15-Year Period. The Direct Medical Costs of Late Presentation (<350/mm(3)) of HIV Infection over a 15-Year Period 2012;2012:757135.17.Krause J, Subklew-Sehume F, Kenyon C, et al. Acceptability of HIV self-testing: a systematic literature review. Acceptability of HIV self-testing: a systematic literature review 2013;13:735.18.Frye V, Wilton L, Hirshfied S, et al. \"Just Because It's Out There, People Aren't Going to Use It.\" HIV Self-Testing Among Young, Black MSM, and Transgender Women. \"Just Because It's Out There, People Aren't Going to Use It.\" HIV Self-Testing Among Young, Black MSM, and Transgender Women 2015.19.Figueroa C, Johnson C, Verster A, et al. Attitudes and Acceptability on HIV Self-testing Among Key Populations: A Literature Review. Attitudes and Acceptability on HIV Self-testing Among Key Populations: A Literature Review 2015.20.Bavinton BR, Brown G, Hurley M, et al. Which gay men would increase their frequency of HIV testing with home self-testing? Which gay men would increase their frequency of HIV testing with home self-testing? 2013;17:2084-92.21.Hensen B, Lewis JJ, Schaap A, et al. Factors associated with HIV-testing and acceptance of an offer of home-based testing by men in rural Zambia. Factors associated with HIV-testing and acceptance of an offer of home-based testing by men in rural Zambia 2015;19:492-504.22.Lee VJ, Tan SC, Earnest A, et al. User acceptability and feasibility of self-testing with HIV rapid tests. User acceptability and feasibility of self-testing with HIV rapid tests 2007;45:449-53.23.Terris-Prestholt F, Hanson K, MacPhail C, et al. How much demand for New HIV prevention technologies can we really expect? Results from a discrete choice experiment in South Africa. How much demand for New HIV prevention technologies can we really expect? Results from a discrete choice experiment in South Africa 2013;8:e83193.24.Ostermann J, Njau B, Brown DS, et al. Heterogeneous HIV Testing Preferences in an Urban Setting in Tanzania: Results from a Discrete Choice Experiment. Heterogeneous HIV Testing Preferences in an Urban Setting in Tanzania: Results from a Discrete Choice Experiment 2014;9:e92100.25.Phillips KA, Maddala T, and Johnson FR. Measuring preferences for health care interventions using conjoint analysis: an application to HIV testing. Measuring preferences for health care interventions using conjoint analysis: an application to HIV testing 2002;37:1681-1705.26.Ryan M. Discrete choice experiments in health care. BMJ 2004;328:360-361.27.Terris-Prestholt F, Quaiffe M, and Vickerman P. Parameterising user uptake in economic evaluations: the role of discrete choice experiments. Parameterising user uptake in economic evaluations: the role of discrete choice experiments In press.28.Philip P, Hickson F, Bonell C, et al. Men who have sex with men in Britain: comparison of estimates from a probability sample and community-based surveys. Men who have sex with men in Britain: comparison of estimates from a probability sample and community-based surveys In submission.29.Orme B. Sample size issues for conjoint analysis, in Getting started with conjoint analysis: strategies for product design and pricing research. 2010, Research Publishers LLC: Madison, Wisconson.)**[22](1.Public Health England. HIV in the United Kingdom: 2014 report. 2014.2.Public Health England. HIV new diagnosis, treatment and care: 2015 report.3.Williamson LM, Dodds JP, Mercey DE, et al. Sexual risk behaviour and knowledge of HIV status among community samples of gay men in the UK. Sexual risk behaviour and knowledge of HIV status among community samples of gay men in the UK 2008;22:1063-70.4.Holt M, Rawstorne P, Wilkinson J, et al. HIV testing, gay community involvement and internet use: social and behavioural correlates of HIV testing among Australian men who have sex with men. HIV testing, gay community involvement and internet use: social and behavioural correlates of HIV testing among Australian men who have sex with men 2012;16:13-22.5.Zablotska I, Holt M, de Wit J, et al. Gay men who are not getting tested for HIV. Gay men who are not getting tested for HIV 2012;16:1887-94.6.Witzel TC, Melendez-Torres GJ, Hickson F, et al. HIV testing history and preferences for future tests among gay men, bisexual men and other MSM in England. HIV testing history and preferences for future tests among gay men, bisexual men and other MSM in England In submission.7.UNSW Australia. 2015. Annual report of trends in behaviour 2015. HIV/AIDS, hepatitis and sexually transmissible infections in Australia [Online]. Available: https://csrh.arts.unsw.edu.au/media/CSRHFile/CSRH_Annual_Report_of_Trends_in_Behaviour_2015.pdf.8.Phillips AN, Cambiano V, Miners A, et al. Potential impact on HIV incidence of higher HIV testing rates and earlier antiretroviral therapy initiation in MSM. Potential impact on HIV incidence of higher HIV testing rates and earlier antiretroviral therapy initiation in MSM 2015;29:1855-62.9.Nakagawa F, Lodwick RK, Smith CJ, et al. Projected life expectancy of people with HIV according to timing of diagnosis. Projected life expectancy of people with HIV according to timing of diagnosis 2012;26:335-43.10.Chadborn TR, Delpech VC, Sabin CA, et al. The late diagnosis and consequent short-term mortality of HIV-infected heterosexuals (England and Wales, 2000–2004). The late diagnosis and consequent short-term mortality of HIV-infected heterosexuals (England and Wales, 2000–2004) 2006;20:2371-2379.11.Miners A, Phillips A, Kreif N, et al. Health-related quality-of-life of people with HIV in the era of combination antiretroviral treatment: a cross-sectional comparison with the general population. Health-related quality-of-life of people with HIV in the era of combination antiretroviral treatment: a cross-sectional comparison with the general population 2014;1:e32–e40.12.Cohen MS, Chen YQ, McCauley M, et al. Prevention of HIV-1 Infection with Early Antiretroviral Therapy. Prevention of HIV-1 Infection with Early Antiretroviral Therapy 2011;365:493-505.13.Marks G, Crepaz N, and Janssen RS. Estimating sexual transmission of HIV from persons aware and unaware that they are infected with the virus in the USA. Estimating sexual transmission of HIV from persons aware and unaware that they are infected with the virus in the USA 2006;20:1447-50.14.Nakagawa F, Miners A, Smith CJ, et al. Projected Lifetime Healthcare Costs Associated with HIV Infection. Projected Lifetime Healthcare Costs Associated with HIV Infection 2015;10:e0125018.15.Fleishman JA, Yehia BR, Moore RD, et al. The Economic Burden of Late Entry Into Medical Care for Patients With HIV Infection. The Economic Burden of Late Entry Into Medical Care for Patients With HIV Infection 2010;48:1071-1079.16.Krentz HB and Gill MJ. The Direct Medical Costs of Late Presentation (<350/mm(3)) of HIV Infection over a 15-Year Period. The Direct Medical Costs of Late Presentation (<350/mm(3)) of HIV Infection over a 15-Year Period 2012;2012:757135.17.Krause J, Subklew-Sehume F, Kenyon C, et al. Acceptability of HIV self-testing: a systematic literature review. Acceptability of HIV self-testing: a systematic literature review 2013;13:735.18.Frye V, Wilton L, Hirshfied S, et al. \"Just Because It's Out There, People Aren't Going to Use It.\" HIV Self-Testing Among Young, Black MSM, and Transgender Women. \"Just Because It's Out There, People Aren't Going to Use It.\" HIV Self-Testing Among Young, Black MSM, and Transgender Women 2015.19.Figueroa C, Johnson C, Verster A, et al. Attitudes and Acceptability on HIV Self-testing Among Key Populations: A Literature Review. Attitudes and Acceptability on HIV Self-testing Among Key Populations: A Literature Review 2015.20.Bavinton BR, Brown G, Hurley M, et al. Which gay men would increase their frequency of HIV testing with home self-testing? Which gay men would increase their frequency of HIV testing with home self-testing? 2013;17:2084-92.21.Hensen B, Lewis JJ, Schaap A, et al. Factors associated with HIV-testing and acceptance of an offer of home-based testing by men in rural Zambia. Factors associated with HIV-testing and acceptance of an offer of home-based testing by men in rural Zambia 2015;19:492-504.22.Lee VJ, Tan SC, Earnest A, et al. User acceptability and feasibility of self-testing with HIV rapid tests. User acceptability and feasibility of self-testing with HIV rapid tests 2007;45:449-53.23.Terris-Prestholt F, Hanson K, MacPhail C, et al. How much demand for New HIV prevention technologies can we really expect? Results from a discrete choice experiment in South Africa. How much demand for New HIV prevention technologies can we really expect? Results from a discrete choice experiment in South Africa 2013;8:e83193.24.Ostermann J, Njau B, Brown DS, et al. Heterogeneous HIV Testing Preferences in an Urban Setting in Tanzania: Results from a Discrete Choice Experiment. Heterogeneous HIV Testing Preferences in an Urban Setting in Tanzania: Results from a Discrete Choice Experiment 2014;9:e92100.25.Phillips KA, Maddala T, and Johnson FR. Measuring preferences for health care interventions using conjoint analysis: an application to HIV testing. Measuring preferences for health care interventions using conjoint analysis: an application to HIV testing 2002;37:1681-1705.26.Ryan M. Discrete choice experiments in health care. BMJ 2004;328:360-361.27.Terris-Prestholt F, Quaiffe M, and Vickerman P. Parameterising user uptake in economic evaluations: the role of discrete choice experiments. Parameterising user uptake in economic evaluations: the role of discrete choice experiments In press.28.Philip P, Hickson F, Bonell C, et al. Men who have sex with men in Britain: comparison of estimates from a probability sample and community-based surveys. Men who have sex with men in Britain: comparison of estimates from a probability sample and community-based surveys In submission.29.Orme B. Sample size issues for conjoint analysis, in Getting started with conjoint analysis: strategies for product design and pricing research. 2010, Research Publishers LLC: Madison, Wisconson.)**[:1063-70.](1.Public Health England. HIV in the United Kingdom: 2014 report. 2014.2.Public Health England. HIV new diagnosis, treatment and care: 2015 report.3.Williamson LM, Dodds JP, Mercey DE, et al. Sexual risk behaviour and knowledge of HIV status among community samples of gay men in the UK. Sexual risk behaviour and knowledge of HIV status among community samples of gay men in the UK 2008;22:1063-70.4.Holt M, Rawstorne P, Wilkinson J, et al. HIV testing, gay community involvement and internet use: social and behavioural correlates of HIV testing among Australian men who have sex with men. HIV testing, gay community involvement and internet use: social and behavioural correlates of HIV testing among Australian men who have sex with men 2012;16:13-22.5.Zablotska I, Holt M, de Wit J, et al. Gay men who are not getting tested for HIV. Gay men who are not getting tested for HIV 2012;16:1887-94.6.Witzel TC, Melendez-Torres GJ, Hickson F, et al. HIV testing history and preferences for future tests among gay men, bisexual men and other MSM in England. HIV testing history and preferences for future tests among gay men, bisexual men and other MSM in England In submission.7.UNSW Australia. 2015. Annual report of trends in behaviour 2015. HIV/AIDS, hepatitis and sexually transmissible infections in Australia [Online]. Available: https://csrh.arts.unsw.edu.au/media/CSRHFile/CSRH_Annual_Report_of_Trends_in_Behaviour_2015.pdf.8.Phillips AN, Cambiano V, Miners A, et al. Potential impact on HIV incidence of higher HIV testing rates and earlier antiretroviral therapy initiation in MSM. Potential impact on HIV incidence of higher HIV testing rates and earlier antiretroviral therapy initiation in MSM 2015;29:1855-62.9.Nakagawa F, Lodwick RK, Smith CJ, et al. Projected life expectancy of people with HIV according to timing of diagnosis. Projected life expectancy of people with HIV according to timing of diagnosis 2012;26:335-43.10.Chadborn TR, Delpech VC, Sabin CA, et al. The late diagnosis and consequent short-term mortality of HIV-infected heterosexuals (England and Wales, 2000–2004). The late diagnosis and consequent short-term mortality of HIV-infected heterosexuals (England and Wales, 2000–2004) 2006;20:2371-2379.11.Miners A, Phillips A, Kreif N, et al. Health-related quality-of-life of people with HIV in the era of combination antiretroviral treatment: a cross-sectional comparison with the general population. Health-related quality-of-life of people with HIV in the era of combination antiretroviral treatment: a cross-sectional comparison with the general population 2014;1:e32–e40.12.Cohen MS, Chen YQ, McCauley M, et al. Prevention of HIV-1 Infection with Early Antiretroviral Therapy. Prevention of HIV-1 Infection with Early Antiretroviral Therapy 2011;365:493-505.13.Marks G, Crepaz N, and Janssen RS. Estimating sexual transmission of HIV from persons aware and unaware that they are infected with the virus in the USA. Estimating sexual transmission of HIV from persons aware and unaware that they are infected with the virus in the USA 2006;20:1447-50.14.Nakagawa F, Miners A, Smith CJ, et al. Projected Lifetime Healthcare Costs Associated with HIV Infection. Projected Lifetime Healthcare Costs Associated with HIV Infection 2015;10:e0125018.15.Fleishman JA, Yehia BR, Moore RD, et al. The Economic Burden of Late Entry Into Medical Care for Patients With HIV Infection. The Economic Burden of Late Entry Into Medical Care for Patients With HIV Infection 2010;48:1071-1079.16.Krentz HB and Gill MJ. The Direct Medical Costs of Late Presentation (<350/mm(3)) of HIV Infection over a 15-Year Period. The Direct Medical Costs of Late Presentation (<350/mm(3)) of HIV Infection over a 15-Year Period 2012;2012:757135.17.Krause J, Subklew-Sehume F, Kenyon C, et al. Acceptability of HIV self-testing: a systematic literature review. Acceptability of HIV self-testing: a systematic literature review 2013;13:735.18.Frye V, Wilton L, Hirshfied S, et al. \"Just Because It's Out There, People Aren't Going to Use It.\" HIV Self-Testing Among Young, Black MSM, and Transgender Women. \"Just Because It's Out There, People Aren't Going to Use It.\" HIV Self-Testing Among Young, Black MSM, and Transgender Women 2015.19.Figueroa C, Johnson C, Verster A, et al. Attitudes and Acceptability on HIV Self-testing Among Key Populations: A Literature Review. Attitudes and Acceptability on HIV Self-testing Among Key Populations: A Literature Review 2015.20.Bavinton BR, Brown G, Hurley M, et al. Which gay men would increase their frequency of HIV testing with home self-testing? Which gay men would increase their frequency of HIV testing with home self-testing? 2013;17:2084-92.21.Hensen B, Lewis JJ, Schaap A, et al. Factors associated with HIV-testing and acceptance of an offer of home-based testing by men in rural Zambia. Factors associated with HIV-testing and acceptance of an offer of home-based testing by men in rural Zambia 2015;19:492-504.22.Lee VJ, Tan SC, Earnest A, et al. User acceptability and feasibility of self-testing with HIV rapid tests. User acceptability and feasibility of self-testing with HIV rapid tests 2007;45:449-53.23.Terris-Prestholt F, Hanson K, MacPhail C, et al. How much demand for New HIV prevention technologies can we really expect? Results from a discrete choice experiment in South Africa. How much demand for New HIV prevention technologies can we really expect? Results from a discrete choice experiment in South Africa 2013;8:e83193.24.Ostermann J, Njau B, Brown DS, et al. Heterogeneous HIV Testing Preferences in an Urban Setting in Tanzania: Results from a Discrete Choice Experiment. Heterogeneous HIV Testing Preferences in an Urban Setting in Tanzania: Results from a Discrete Choice Experiment 2014;9:e92100.25.Phillips KA, Maddala T, and Johnson FR. Measuring preferences for health care interventions using conjoint analysis: an application to HIV testing. Measuring preferences for health care interventions using conjoint analysis: an application to HIV testing 2002;37:1681-1705.26.Ryan M. Discrete choice experiments in health care. BMJ 2004;328:360-361.27.Terris-Prestholt F, Quaiffe M, and Vickerman P. Parameterising user uptake in economic evaluations: the role of discrete choice experiments. Parameterising user uptake in economic evaluations: the role of discrete choice experiments In press.28.Philip P, Hickson F, Bonell C, et al. Men who have sex with men in Britain: comparison of estimates from a probability sample and community-based surveys. Men who have sex with men in Britain: comparison of estimates from a probability sample and community-based surveys In submission.29.Orme B. Sample size issues for conjoint analysis, in Getting started with conjoint analysis: strategies for product design and pricing research. 2010, Research Publishers LLC: Madison, Wisconson.)

[4. Holt M, Rawstorne P, Wilkinson J, et al. HIV testing, gay community involvement and internet use: social and behavioural correlates of HIV testing among Australian men who have sex with men](1.Public Health England. HIV in the United Kingdom: 2014 report. 2014.2.Public Health England. HIV new diagnosis, treatment and care: 2015 report.3.Williamson LM, Dodds JP, Mercey DE, et al. Sexual risk behaviour and knowledge of HIV status among community samples of gay men in the UK. Sexual risk behaviour and knowledge of HIV status among community samples of gay men in the UK 2008;22:1063-70.4.Holt M, Rawstorne P, Wilkinson J, et al. HIV testing, gay community involvement and internet use: social and behavioural correlates of HIV testing among Australian men who have sex with men. HIV testing, gay community involvement and internet use: social and behavioural correlates of HIV testing among Australian men who have sex with men 2012;16:13-22.5.Zablotska I, Holt M, de Wit J, et al. Gay men who are not getting tested for HIV. Gay men who are not getting tested for HIV 2012;16:1887-94.6.Witzel TC, Melendez-Torres GJ, Hickson F, et al. HIV testing history and preferences for future tests among gay men, bisexual men and other MSM in England. HIV testing history and preferences for future tests among gay men, bisexual men and other MSM in England In submission.7.UNSW Australia. 2015. Annual report of trends in behaviour 2015. HIV/AIDS, hepatitis and sexually transmissible infections in Australia [Online]. Available: https://csrh.arts.unsw.edu.au/media/CSRHFile/CSRH_Annual_Report_of_Trends_in_Behaviour_2015.pdf.8.Phillips AN, Cambiano V, Miners A, et al. Potential impact on HIV incidence of higher HIV testing rates and earlier antiretroviral therapy initiation in MSM. Potential impact on HIV incidence of higher HIV testing rates and earlier antiretroviral therapy initiation in MSM 2015;29:1855-62.9.Nakagawa F, Lodwick RK, Smith CJ, et al. Projected life expectancy of people with HIV according to timing of diagnosis. Projected life expectancy of people with HIV according to timing of diagnosis 2012;26:335-43.10.Chadborn TR, Delpech VC, Sabin CA, et al. The late diagnosis and consequent short-term mortality of HIV-infected heterosexuals (England and Wales, 2000–2004). The late diagnosis and consequent short-term mortality of HIV-infected heterosexuals (England and Wales, 2000–2004) 2006;20:2371-2379.11.Miners A, Phillips A, Kreif N, et al. Health-related quality-of-life of people with HIV in the era of combination antiretroviral treatment: a cross-sectional comparison with the general population. Health-related quality-of-life of people with HIV in the era of combination antiretroviral treatment: a cross-sectional comparison with the general population 2014;1:e32–e40.12.Cohen MS, Chen YQ, McCauley M, et al. Prevention of HIV-1 Infection with Early Antiretroviral Therapy. Prevention of HIV-1 Infection with Early Antiretroviral Therapy 2011;365:493-505.13.Marks G, Crepaz N, and Janssen RS. Estimating sexual transmission of HIV from persons aware and unaware that they are infected with the virus in the USA. Estimating sexual transmission of HIV from persons aware and unaware that they are infected with the virus in the USA 2006;20:1447-50.14.Nakagawa F, Miners A, Smith CJ, et al. Projected Lifetime Healthcare Costs Associated with HIV Infection. Projected Lifetime Healthcare Costs Associated with HIV Infection 2015;10:e0125018.15.Fleishman JA, Yehia BR, Moore RD, et al. The Economic Burden of Late Entry Into Medical Care for Patients With HIV Infection. The Economic Burden of Late Entry Into Medical Care for Patients With HIV Infection 2010;48:1071-1079.16.Krentz HB and Gill MJ. The Direct Medical Costs of Late Presentation (<350/mm(3)) of HIV Infection over a 15-Year Period. The Direct Medical Costs of Late Presentation (<350/mm(3)) of HIV Infection over a 15-Year Period 2012;2012:757135.17.Krause J, Subklew-Sehume F, Kenyon C, et al. Acceptability of HIV self-testing: a systematic literature review. Acceptability of HIV self-testing: a systematic literature review 2013;13:735.18.Frye V, Wilton L, Hirshfied S, et al. \"Just Because It's Out There, People Aren't Going to Use It.\" HIV Self-Testing Among Young, Black MSM, and Transgender Women. \"Just Because It's Out There, People Aren't Going to Use It.\" HIV Self-Testing Among Young, Black MSM, and Transgender Women 2015.19.Figueroa C, Johnson C, Verster A, et al. Attitudes and Acceptability on HIV Self-testing Among Key Populations: A Literature Review. Attitudes and Acceptability on HIV Self-testing Among Key Populations: A Literature Review 2015.20.Bavinton BR, Brown G, Hurley M, et al. Which gay men would increase their frequency of HIV testing with home self-testing? Which gay men would increase their frequency of HIV testing with home self-testing? 2013;17:2084-92.21.Hensen B, Lewis JJ, Schaap A, et al. Factors associated with HIV-testing and acceptance of an offer of home-based testing by men in rural Zambia. Factors associated with HIV-testing and acceptance of an offer of home-based testing by men in rural Zambia 2015;19:492-504.22.Lee VJ, Tan SC, Earnest A, et al. User acceptability and feasibility of self-testing with HIV rapid tests. User acceptability and feasibility of self-testing with HIV rapid tests 2007;45:449-53.23.Terris-Prestholt F, Hanson K, MacPhail C, et al. How much demand for New HIV prevention technologies can we really expect? Results from a discrete choice experiment in South Africa. How much demand for New HIV prevention technologies can we really expect? Results from a discrete choice experiment in South Africa 2013;8:e83193.24.Ostermann J, Njau B, Brown DS, et al. Heterogeneous HIV Testing Preferences in an Urban Setting in Tanzania: Results from a Discrete Choice Experiment. Heterogeneous HIV Testing Preferences in an Urban Setting in Tanzania: Results from a Discrete Choice Experiment 2014;9:e92100.25.Phillips KA, Maddala T, and Johnson FR. Measuring preferences for health care interventions using conjoint analysis: an application to HIV testing. Measuring preferences for health care interventions using conjoint analysis: an application to HIV testing 2002;37:1681-1705.26.Ryan M. Discrete choice experiments in health care. BMJ 2004;328:360-361.27.Terris-Prestholt F, Quaiffe M, and Vickerman P. Parameterising user uptake in economic evaluations: the role of discrete choice experiments. Parameterising user uptake in economic evaluations: the role of discrete choice experiments In press.28.Philip P, Hickson F, Bonell C, et al. Men who have sex with men in Britain: comparison of estimates from a probability sample and community-based surveys. Men who have sex with men in Britain: comparison of estimates from a probability sample and community-based surveys In submission.29.Orme B. Sample size issues for conjoint analysis, in Getting started with conjoint analysis: strategies for product design and pricing research. 2010, Research Publishers LLC: Madison, Wisconson.)*[.](1.Public Health England. HIV in the United Kingdom: 2014 report. 2014.2.Public Health England. HIV new diagnosis, treatment and care: 2015 report.3.Williamson LM, Dodds JP, Mercey DE, et al. Sexual risk behaviour and knowledge of HIV status among community samples of gay men in the UK. Sexual risk behaviour and knowledge of HIV status among community samples of gay men in the UK 2008;22:1063-70.4.Holt M, Rawstorne P, Wilkinson J, et al. HIV testing, gay community involvement and internet use: social and behavioural correlates of HIV testing among Australian men who have sex with men. HIV testing, gay community involvement and internet use: social and behavioural correlates of HIV testing among Australian men who have sex with men 2012;16:13-22.5.Zablotska I, Holt M, de Wit J, et al. Gay men who are not getting tested for HIV. Gay men who are not getting tested for HIV 2012;16:1887-94.6.Witzel TC, Melendez-Torres GJ, Hickson F, et al. HIV testing history and preferences for future tests among gay men, bisexual men and other MSM in England. HIV testing history and preferences for future tests among gay men, bisexual men and other MSM in England In submission.7.UNSW Australia. 2015. Annual report of trends in behaviour 2015. HIV/AIDS, hepatitis and sexually transmissible infections in Australia [Online]. Available: https://csrh.arts.unsw.edu.au/media/CSRHFile/CSRH_Annual_Report_of_Trends_in_Behaviour_2015.pdf.8.Phillips AN, Cambiano V, Miners A, et al. Potential impact on HIV incidence of higher HIV testing rates and earlier antiretroviral therapy initiation in MSM. Potential impact on HIV incidence of higher HIV testing rates and earlier antiretroviral therapy initiation in MSM 2015;29:1855-62.9.Nakagawa F, Lodwick RK, Smith CJ, et al. Projected life expectancy of people with HIV according to timing of diagnosis. Projected life expectancy of people with HIV according to timing of diagnosis 2012;26:335-43.10.Chadborn TR, Delpech VC, Sabin CA, et al. The late diagnosis and consequent short-term mortality of HIV-infected heterosexuals (England and Wales, 2000–2004). The late diagnosis and consequent short-term mortality of HIV-infected heterosexuals (England and Wales, 2000–2004) 2006;20:2371-2379.11.Miners A, Phillips A, Kreif N, et al. Health-related quality-of-life of people with HIV in the era of combination antiretroviral treatment: a cross-sectional comparison with the general population. Health-related quality-of-life of people with HIV in the era of combination antiretroviral treatment: a cross-sectional comparison with the general population 2014;1:e32–e40.12.Cohen MS, Chen YQ, McCauley M, et al. Prevention of HIV-1 Infection with Early Antiretroviral Therapy. Prevention of HIV-1 Infection with Early Antiretroviral Therapy 2011;365:493-505.13.Marks G, Crepaz N, and Janssen RS. Estimating sexual transmission of HIV from persons aware and unaware that they are infected with the virus in the USA. Estimating sexual transmission of HIV from persons aware and unaware that they are infected with the virus in the USA 2006;20:1447-50.14.Nakagawa F, Miners A, Smith CJ, et al. Projected Lifetime Healthcare Costs Associated with HIV Infection. Projected Lifetime Healthcare Costs Associated with HIV Infection 2015;10:e0125018.15.Fleishman JA, Yehia BR, Moore RD, et al. The Economic Burden of Late Entry Into Medical Care for Patients With HIV Infection. The Economic Burden of Late Entry Into Medical Care for Patients With HIV Infection 2010;48:1071-1079.16.Krentz HB and Gill MJ. The Direct Medical Costs of Late Presentation (<350/mm(3)) of HIV Infection over a 15-Year Period. The Direct Medical Costs of Late Presentation (<350/mm(3)) of HIV Infection over a 15-Year Period 2012;2012:757135.17.Krause J, Subklew-Sehume F, Kenyon C, et al. Acceptability of HIV self-testing: a systematic literature review. Acceptability of HIV self-testing: a systematic literature review 2013;13:735.18.Frye V, Wilton L, Hirshfied S, et al. \"Just Because It's Out There, People Aren't Going to Use It.\" HIV Self-Testing Among Young, Black MSM, and Transgender Women. \"Just Because It's Out There, People Aren't Going to Use It.\" HIV Self-Testing Among Young, Black MSM, and Transgender Women 2015.19.Figueroa C, Johnson C, Verster A, et al. Attitudes and Acceptability on HIV Self-testing Among Key Populations: A Literature Review. Attitudes and Acceptability on HIV Self-testing Among Key Populations: A Literature Review 2015.20.Bavinton BR, Brown G, Hurley M, et al. Which gay men would increase their frequency of HIV testing with home self-testing? Which gay men would increase their frequency of HIV testing with home self-testing? 2013;17:2084-92.21.Hensen B, Lewis JJ, Schaap A, et al. Factors associated with HIV-testing and acceptance of an offer of home-based testing by men in rural Zambia. Factors associated with HIV-testing and acceptance of an offer of home-based testing by men in rural Zambia 2015;19:492-504.22.Lee VJ, Tan SC, Earnest A, et al. User acceptability and feasibility of self-testing with HIV rapid tests. User acceptability and feasibility of self-testing with HIV rapid tests 2007;45:449-53.23.Terris-Prestholt F, Hanson K, MacPhail C, et al. How much demand for New HIV prevention technologies can we really expect? Results from a discrete choice experiment in South Africa. How much demand for New HIV prevention technologies can we really expect? Results from a discrete choice experiment in South Africa 2013;8:e83193.24.Ostermann J, Njau B, Brown DS, et al. Heterogeneous HIV Testing Preferences in an Urban Setting in Tanzania: Results from a Discrete Choice Experiment. Heterogeneous HIV Testing Preferences in an Urban Setting in Tanzania: Results from a Discrete Choice Experiment 2014;9:e92100.25.Phillips KA, Maddala T, and Johnson FR. Measuring preferences for health care interventions using conjoint analysis: an application to HIV testing. Measuring preferences for health care interventions using conjoint analysis: an application to HIV testing 2002;37:1681-1705.26.Ryan M. Discrete choice experiments in health care. BMJ 2004;328:360-361.27.Terris-Prestholt F, Quaiffe M, and Vickerman P. Parameterising user uptake in economic evaluations: the role of discrete choice experiments. Parameterising user uptake in economic evaluations: the role of discrete choice experiments In press.28.Philip P, Hickson F, Bonell C, et al. Men who have sex with men in Britain: comparison of estimates from a probability sample and community-based surveys. Men who have sex with men in Britain: comparison of estimates from a probability sample and community-based surveys In submission.29.Orme B. Sample size issues for conjoint analysis, in Getting started with conjoint analysis: strategies for product design and pricing research. 2010, Research Publishers LLC: Madison, Wisconson.)**[HIV testing, gay community involvement and internet use: social and behavioural correlates of HIV testing among Australian men who have sex with men](1.Public Health England. HIV in the United Kingdom: 2014 report. 2014.2.Public Health England. HIV new diagnosis, treatment and care: 2015 report.3.Williamson LM, Dodds JP, Mercey DE, et al. Sexual risk behaviour and knowledge of HIV status among community samples of gay men in the UK. Sexual risk behaviour and knowledge of HIV status among community samples of gay men in the UK 2008;22:1063-70.4.Holt M, Rawstorne P, Wilkinson J, et al. HIV testing, gay community involvement and internet use: social and behavioural correlates of HIV testing among Australian men who have sex with men. HIV testing, gay community involvement and internet use: social and behavioural correlates of HIV testing among Australian men who have sex with men 2012;16:13-22.5.Zablotska I, Holt M, de Wit J, et al. Gay men who are not getting tested for HIV. Gay men who are not getting tested for HIV 2012;16:1887-94.6.Witzel TC, Melendez-Torres GJ, Hickson F, et al. HIV testing history and preferences for future tests among gay men, bisexual men and other MSM in England. HIV testing history and preferences for future tests among gay men, bisexual men and other MSM in England In submission.7.UNSW Australia. 2015. Annual report of trends in behaviour 2015. HIV/AIDS, hepatitis and sexually transmissible infections in Australia [Online]. Available: https://csrh.arts.unsw.edu.au/media/CSRHFile/CSRH_Annual_Report_of_Trends_in_Behaviour_2015.pdf.8.Phillips AN, Cambiano V, Miners A, et al. Potential impact on HIV incidence of higher HIV testing rates and earlier antiretroviral therapy initiation in MSM. Potential impact on HIV incidence of higher HIV testing rates and earlier antiretroviral therapy initiation in MSM 2015;29:1855-62.9.Nakagawa F, Lodwick RK, Smith CJ, et al. Projected life expectancy of people with HIV according to timing of diagnosis. Projected life expectancy of people with HIV according to timing of diagnosis 2012;26:335-43.10.Chadborn TR, Delpech VC, Sabin CA, et al. The late diagnosis and consequent short-term mortality of HIV-infected heterosexuals (England and Wales, 2000–2004). The late diagnosis and consequent short-term mortality of HIV-infected heterosexuals (England and Wales, 2000–2004) 2006;20:2371-2379.11.Miners A, Phillips A, Kreif N, et al. Health-related quality-of-life of people with HIV in the era of combination antiretroviral treatment: a cross-sectional comparison with the general population. Health-related quality-of-life of people with HIV in the era of combination antiretroviral treatment: a cross-sectional comparison with the general population 2014;1:e32–e40.12.Cohen MS, Chen YQ, McCauley M, et al. Prevention of HIV-1 Infection with Early Antiretroviral Therapy. Prevention of HIV-1 Infection with Early Antiretroviral Therapy 2011;365:493-505.13.Marks G, Crepaz N, and Janssen RS. Estimating sexual transmission of HIV from persons aware and unaware that they are infected with the virus in the USA. Estimating sexual transmission of HIV from persons aware and unaware that they are infected with the virus in the USA 2006;20:1447-50.14.Nakagawa F, Miners A, Smith CJ, et al. Projected Lifetime Healthcare Costs Associated with HIV Infection. Projected Lifetime Healthcare Costs Associated with HIV Infection 2015;10:e0125018.15.Fleishman JA, Yehia BR, Moore RD, et al. The Economic Burden of Late Entry Into Medical Care for Patients With HIV Infection. The Economic Burden of Late Entry Into Medical Care for Patients With HIV Infection 2010;48:1071-1079.16.Krentz HB and Gill MJ. The Direct Medical Costs of Late Presentation (<350/mm(3)) of HIV Infection over a 15-Year Period. The Direct Medical Costs of Late Presentation (<350/mm(3)) of HIV Infection over a 15-Year Period 2012;2012:757135.17.Krause J, Subklew-Sehume F, Kenyon C, et al. Acceptability of HIV self-testing: a systematic literature review. Acceptability of HIV self-testing: a systematic literature review 2013;13:735.18.Frye V, Wilton L, Hirshfied S, et al. \"Just Because It's Out There, People Aren't Going to Use It.\" HIV Self-Testing Among Young, Black MSM, and Transgender Women. \"Just Because It's Out There, People Aren't Going to Use It.\" HIV Self-Testing Among Young, Black MSM, and Transgender Women 2015.19.Figueroa C, Johnson C, Verster A, et al. Attitudes and Acceptability on HIV Self-testing Among Key Populations: A Literature Review. Attitudes and Acceptability on HIV Self-testing Among Key Populations: A Literature Review 2015.20.Bavinton BR, Brown G, Hurley M, et al. Which gay men would increase their frequency of HIV testing with home self-testing? Which gay men would increase their frequency of HIV testing with home self-testing? 2013;17:2084-92.21.Hensen B, Lewis JJ, Schaap A, et al. Factors associated with HIV-testing and acceptance of an offer of home-based testing by men in rural Zambia. Factors associated with HIV-testing and acceptance of an offer of home-based testing by men in rural Zambia 2015;19:492-504.22.Lee VJ, Tan SC, Earnest A, et al. User acceptability and feasibility of self-testing with HIV rapid tests. User acceptability and feasibility of self-testing with HIV rapid tests 2007;45:449-53.23.Terris-Prestholt F, Hanson K, MacPhail C, et al. How much demand for New HIV prevention technologies can we really expect? Results from a discrete choice experiment in South Africa. How much demand for New HIV prevention technologies can we really expect? Results from a discrete choice experiment in South Africa 2013;8:e83193.24.Ostermann J, Njau B, Brown DS, et al. Heterogeneous HIV Testing Preferences in an Urban Setting in Tanzania: Results from a Discrete Choice Experiment. Heterogeneous HIV Testing Preferences in an Urban Setting in Tanzania: Results from a Discrete Choice Experiment 2014;9:e92100.25.Phillips KA, Maddala T, and Johnson FR. Measuring preferences for health care interventions using conjoint analysis: an application to HIV testing. Measuring preferences for health care interventions using conjoint analysis: an application to HIV testing 2002;37:1681-1705.26.Ryan M. Discrete choice experiments in health care. BMJ 2004;328:360-361.27.Terris-Prestholt F, Quaiffe M, and Vickerman P. Parameterising user uptake in economic evaluations: the role of discrete choice experiments. Parameterising user uptake in economic evaluations: the role of discrete choice experiments In press.28.Philip P, Hickson F, Bonell C, et al. Men who have sex with men in Britain: comparison of estimates from a probability sample and community-based surveys. Men who have sex with men in Britain: comparison of estimates from a probability sample and community-based surveys In submission.29.Orme B. Sample size issues for conjoint analysis, in Getting started with conjoint analysis: strategies for product design and pricing research. 2010, Research Publishers LLC: Madison, Wisconson.)* [2012;](1.Public Health England. HIV in the United Kingdom: 2014 report. 2014.2.Public Health England. HIV new diagnosis, treatment and care: 2015 report.3.Williamson LM, Dodds JP, Mercey DE, et al. Sexual risk behaviour and knowledge of HIV status among community samples of gay men in the UK. Sexual risk behaviour and knowledge of HIV status among community samples of gay men in the UK 2008;22:1063-70.4.Holt M, Rawstorne P, Wilkinson J, et al. HIV testing, gay community involvement and internet use: social and behavioural correlates of HIV testing among Australian men who have sex with men. HIV testing, gay community involvement and internet use: social and behavioural correlates of HIV testing among Australian men who have sex with men 2012;16:13-22.5.Zablotska I, Holt M, de Wit J, et al. Gay men who are not getting tested for HIV. Gay men who are not getting tested for HIV 2012;16:1887-94.6.Witzel TC, Melendez-Torres GJ, Hickson F, et al. HIV testing history and preferences for future tests among gay men, bisexual men and other MSM in England. HIV testing history and preferences for future tests among gay men, bisexual men and other MSM in England In submission.7.UNSW Australia. 2015. Annual report of trends in behaviour 2015. HIV/AIDS, hepatitis and sexually transmissible infections in Australia [Online]. Available: https://csrh.arts.unsw.edu.au/media/CSRHFile/CSRH_Annual_Report_of_Trends_in_Behaviour_2015.pdf.8.Phillips AN, Cambiano V, Miners A, et al. Potential impact on HIV incidence of higher HIV testing rates and earlier antiretroviral therapy initiation in MSM. Potential impact on HIV incidence of higher HIV testing rates and earlier antiretroviral therapy initiation in MSM 2015;29:1855-62.9.Nakagawa F, Lodwick RK, Smith CJ, et al. Projected life expectancy of people with HIV according to timing of diagnosis. Projected life expectancy of people with HIV according to timing of diagnosis 2012;26:335-43.10.Chadborn TR, Delpech VC, Sabin CA, et al. The late diagnosis and consequent short-term mortality of HIV-infected heterosexuals (England and Wales, 2000–2004). The late diagnosis and consequent short-term mortality of HIV-infected heterosexuals (England and Wales, 2000–2004) 2006;20:2371-2379.11.Miners A, Phillips A, Kreif N, et al. Health-related quality-of-life of people with HIV in the era of combination antiretroviral treatment: a cross-sectional comparison with the general population. Health-related quality-of-life of people with HIV in the era of combination antiretroviral treatment: a cross-sectional comparison with the general population 2014;1:e32–e40.12.Cohen MS, Chen YQ, McCauley M, et al. Prevention of HIV-1 Infection with Early Antiretroviral Therapy. Prevention of HIV-1 Infection with Early Antiretroviral Therapy 2011;365:493-505.13.Marks G, Crepaz N, and Janssen RS. Estimating sexual transmission of HIV from persons aware and unaware that they are infected with the virus in the USA. Estimating sexual transmission of HIV from persons aware and unaware that they are infected with the virus in the USA 2006;20:1447-50.14.Nakagawa F, Miners A, Smith CJ, et al. Projected Lifetime Healthcare Costs Associated with HIV Infection. Projected Lifetime Healthcare Costs Associated with HIV Infection 2015;10:e0125018.15.Fleishman JA, Yehia BR, Moore RD, et al. The Economic Burden of Late Entry Into Medical Care for Patients With HIV Infection. The Economic Burden of Late Entry Into Medical Care for Patients With HIV Infection 2010;48:1071-1079.16.Krentz HB and Gill MJ. The Direct Medical Costs of Late Presentation (<350/mm(3)) of HIV Infection over a 15-Year Period. The Direct Medical Costs of Late Presentation (<350/mm(3)) of HIV Infection over a 15-Year Period 2012;2012:757135.17.Krause J, Subklew-Sehume F, Kenyon C, et al. Acceptability of HIV self-testing: a systematic literature review. Acceptability of HIV self-testing: a systematic literature review 2013;13:735.18.Frye V, Wilton L, Hirshfied S, et al. \"Just Because It's Out There, People Aren't Going to Use It.\" HIV Self-Testing Among Young, Black MSM, and Transgender Women. \"Just Because It's Out There, People Aren't Going to Use It.\" HIV Self-Testing Among Young, Black MSM, and Transgender Women 2015.19.Figueroa C, Johnson C, Verster A, et al. Attitudes and Acceptability on HIV Self-testing Among Key Populations: A Literature Review. Attitudes and Acceptability on HIV Self-testing Among Key Populations: A Literature Review 2015.20.Bavinton BR, Brown G, Hurley M, et al. Which gay men would increase their frequency of HIV testing with home self-testing? Which gay men would increase their frequency of HIV testing with home self-testing? 2013;17:2084-92.21.Hensen B, Lewis JJ, Schaap A, et al. Factors associated with HIV-testing and acceptance of an offer of home-based testing by men in rural Zambia. Factors associated with HIV-testing and acceptance of an offer of home-based testing by men in rural Zambia 2015;19:492-504.22.Lee VJ, Tan SC, Earnest A, et al. User acceptability and feasibility of self-testing with HIV rapid tests. User acceptability and feasibility of self-testing with HIV rapid tests 2007;45:449-53.23.Terris-Prestholt F, Hanson K, MacPhail C, et al. How much demand for New HIV prevention technologies can we really expect? Results from a discrete choice experiment in South Africa. How much demand for New HIV prevention technologies can we really expect? Results from a discrete choice experiment in South Africa 2013;8:e83193.24.Ostermann J, Njau B, Brown DS, et al. Heterogeneous HIV Testing Preferences in an Urban Setting in Tanzania: Results from a Discrete Choice Experiment. Heterogeneous HIV Testing Preferences in an Urban Setting in Tanzania: Results from a Discrete Choice Experiment 2014;9:e92100.25.Phillips KA, Maddala T, and Johnson FR. Measuring preferences for health care interventions using conjoint analysis: an application to HIV testing. Measuring preferences for health care interventions using conjoint analysis: an application to HIV testing 2002;37:1681-1705.26.Ryan M. Discrete choice experiments in health care. BMJ 2004;328:360-361.27.Terris-Prestholt F, Quaiffe M, and Vickerman P. Parameterising user uptake in economic evaluations: the role of discrete choice experiments. Parameterising user uptake in economic evaluations: the role of discrete choice experiments In press.28.Philip P, Hickson F, Bonell C, et al. Men who have sex with men in Britain: comparison of estimates from a probability sample and community-based surveys. Men who have sex with men in Britain: comparison of estimates from a probability sample and community-based surveys In submission.29.Orme B. Sample size issues for conjoint analysis, in Getting started with conjoint analysis: strategies for product design and pricing research. 2010, Research Publishers LLC: Madison, Wisconson.)**[16](1.Public Health England. HIV in the United Kingdom: 2014 report. 2014.2.Public Health England. HIV new diagnosis, treatment and care: 2015 report.3.Williamson LM, Dodds JP, Mercey DE, et al. Sexual risk behaviour and knowledge of HIV status among community samples of gay men in the UK. Sexual risk behaviour and knowledge of HIV status among community samples of gay men in the UK 2008;22:1063-70.4.Holt M, Rawstorne P, Wilkinson J, et al. HIV testing, gay community involvement and internet use: social and behavioural correlates of HIV testing among Australian men who have sex with men. HIV testing, gay community involvement and internet use: social and behavioural correlates of HIV testing among Australian men who have sex with men 2012;16:13-22.5.Zablotska I, Holt M, de Wit J, et al. Gay men who are not getting tested for HIV. Gay men who are not getting tested for HIV 2012;16:1887-94.6.Witzel TC, Melendez-Torres GJ, Hickson F, et al. HIV testing history and preferences for future tests among gay men, bisexual men and other MSM in England. HIV testing history and preferences for future tests among gay men, bisexual men and other MSM in England In submission.7.UNSW Australia. 2015. Annual report of trends in behaviour 2015. HIV/AIDS, hepatitis and sexually transmissible infections in Australia [Online]. Available: https://csrh.arts.unsw.edu.au/media/CSRHFile/CSRH_Annual_Report_of_Trends_in_Behaviour_2015.pdf.8.Phillips AN, Cambiano V, Miners A, et al. Potential impact on HIV incidence of higher HIV testing rates and earlier antiretroviral therapy initiation in MSM. Potential impact on HIV incidence of higher HIV testing rates and earlier antiretroviral therapy initiation in MSM 2015;29:1855-62.9.Nakagawa F, Lodwick RK, Smith CJ, et al. Projected life expectancy of people with HIV according to timing of diagnosis. Projected life expectancy of people with HIV according to timing of diagnosis 2012;26:335-43.10.Chadborn TR, Delpech VC, Sabin CA, et al. The late diagnosis and consequent short-term mortality of HIV-infected heterosexuals (England and Wales, 2000–2004). The late diagnosis and consequent short-term mortality of HIV-infected heterosexuals (England and Wales, 2000–2004) 2006;20:2371-2379.11.Miners A, Phillips A, Kreif N, et al. Health-related quality-of-life of people with HIV in the era of combination antiretroviral treatment: a cross-sectional comparison with the general population. Health-related quality-of-life of people with HIV in the era of combination antiretroviral treatment: a cross-sectional comparison with the general population 2014;1:e32–e40.12.Cohen MS, Chen YQ, McCauley M, et al. Prevention of HIV-1 Infection with Early Antiretroviral Therapy. Prevention of HIV-1 Infection with Early Antiretroviral Therapy 2011;365:493-505.13.Marks G, Crepaz N, and Janssen RS. Estimating sexual transmission of HIV from persons aware and unaware that they are infected with the virus in the USA. Estimating sexual transmission of HIV from persons aware and unaware that they are infected with the virus in the USA 2006;20:1447-50.14.Nakagawa F, Miners A, Smith CJ, et al. Projected Lifetime Healthcare Costs Associated with HIV Infection. Projected Lifetime Healthcare Costs Associated with HIV Infection 2015;10:e0125018.15.Fleishman JA, Yehia BR, Moore RD, et al. The Economic Burden of Late Entry Into Medical Care for Patients With HIV Infection. The Economic Burden of Late Entry Into Medical Care for Patients With HIV Infection 2010;48:1071-1079.16.Krentz HB and Gill MJ. The Direct Medical Costs of Late Presentation (<350/mm(3)) of HIV Infection over a 15-Year Period. The Direct Medical Costs of Late Presentation (<350/mm(3)) of HIV Infection over a 15-Year Period 2012;2012:757135.17.Krause J, Subklew-Sehume F, Kenyon C, et al. Acceptability of HIV self-testing: a systematic literature review. Acceptability of HIV self-testing: a systematic literature review 2013;13:735.18.Frye V, Wilton L, Hirshfied S, et al. \"Just Because It's Out There, People Aren't Going to Use It.\" HIV Self-Testing Among Young, Black MSM, and Transgender Women. \"Just Because It's Out There, People Aren't Going to Use It.\" HIV Self-Testing Among Young, Black MSM, and Transgender Women 2015.19.Figueroa C, Johnson C, Verster A, et al. Attitudes and Acceptability on HIV Self-testing Among Key Populations: A Literature Review. Attitudes and Acceptability on HIV Self-testing Among Key Populations: A Literature Review 2015.20.Bavinton BR, Brown G, Hurley M, et al. Which gay men would increase their frequency of HIV testing with home self-testing? Which gay men would increase their frequency of HIV testing with home self-testing? 2013;17:2084-92.21.Hensen B, Lewis JJ, Schaap A, et al. Factors associated with HIV-testing and acceptance of an offer of home-based testing by men in rural Zambia. Factors associated with HIV-testing and acceptance of an offer of home-based testing by men in rural Zambia 2015;19:492-504.22.Lee VJ, Tan SC, Earnest A, et al. User acceptability and feasibility of self-testing with HIV rapid tests. User acceptability and feasibility of self-testing with HIV rapid tests 2007;45:449-53.23.Terris-Prestholt F, Hanson K, MacPhail C, et al. How much demand for New HIV prevention technologies can we really expect? Results from a discrete choice experiment in South Africa. How much demand for New HIV prevention technologies can we really expect? Results from a discrete choice experiment in South Africa 2013;8:e83193.24.Ostermann J, Njau B, Brown DS, et al. Heterogeneous HIV Testing Preferences in an Urban Setting in Tanzania: Results from a Discrete Choice Experiment. Heterogeneous HIV Testing Preferences in an Urban Setting in Tanzania: Results from a Discrete Choice Experiment 2014;9:e92100.25.Phillips KA, Maddala T, and Johnson FR. Measuring preferences for health care interventions using conjoint analysis: an application to HIV testing. Measuring preferences for health care interventions using conjoint analysis: an application to HIV testing 2002;37:1681-1705.26.Ryan M. Discrete choice experiments in health care. BMJ 2004;328:360-361.27.Terris-Prestholt F, Quaiffe M, and Vickerman P. Parameterising user uptake in economic evaluations: the role of discrete choice experiments. Parameterising user uptake in economic evaluations: the role of discrete choice experiments In press.28.Philip P, Hickson F, Bonell C, et al. Men who have sex with men in Britain: comparison of estimates from a probability sample and community-based surveys. Men who have sex with men in Britain: comparison of estimates from a probability sample and community-based surveys In submission.29.Orme B. Sample size issues for conjoint analysis, in Getting started with conjoint analysis: strategies for product design and pricing research. 2010, Research Publishers LLC: Madison, Wisconson.)**[:13-22.](1.Public Health England. HIV in the United Kingdom: 2014 report. 2014.2.Public Health England. HIV new diagnosis, treatment and care: 2015 report.3.Williamson LM, Dodds JP, Mercey DE, et al. Sexual risk behaviour and knowledge of HIV status among community samples of gay men in the UK. Sexual risk behaviour and knowledge of HIV status among community samples of gay men in the UK 2008;22:1063-70.4.Holt M, Rawstorne P, Wilkinson J, et al. HIV testing, gay community involvement and internet use: social and behavioural correlates of HIV testing among Australian men who have sex with men. HIV testing, gay community involvement and internet use: social and behavioural correlates of HIV testing among Australian men who have sex with men 2012;16:13-22.5.Zablotska I, Holt M, de Wit J, et al. Gay men who are not getting tested for HIV. Gay men who are not getting tested for HIV 2012;16:1887-94.6.Witzel TC, Melendez-Torres GJ, Hickson F, et al. HIV testing history and preferences for future tests among gay men, bisexual men and other MSM in England. HIV testing history and preferences for future tests among gay men, bisexual men and other MSM in England In submission.7.UNSW Australia. 2015. Annual report of trends in behaviour 2015. HIV/AIDS, hepatitis and sexually transmissible infections in Australia [Online]. Available: https://csrh.arts.unsw.edu.au/media/CSRHFile/CSRH_Annual_Report_of_Trends_in_Behaviour_2015.pdf.8.Phillips AN, Cambiano V, Miners A, et al. Potential impact on HIV incidence of higher HIV testing rates and earlier antiretroviral therapy initiation in MSM. Potential impact on HIV incidence of higher HIV testing rates and earlier antiretroviral therapy initiation in MSM 2015;29:1855-62.9.Nakagawa F, Lodwick RK, Smith CJ, et al. Projected life expectancy of people with HIV according to timing of diagnosis. Projected life expectancy of people with HIV according to timing of diagnosis 2012;26:335-43.10.Chadborn TR, Delpech VC, Sabin CA, et al. The late diagnosis and consequent short-term mortality of HIV-infected heterosexuals (England and Wales, 2000–2004). The late diagnosis and consequent short-term mortality of HIV-infected heterosexuals (England and Wales, 2000–2004) 2006;20:2371-2379.11.Miners A, Phillips A, Kreif N, et al. Health-related quality-of-life of people with HIV in the era of combination antiretroviral treatment: a cross-sectional comparison with the general population. Health-related quality-of-life of people with HIV in the era of combination antiretroviral treatment: a cross-sectional comparison with the general population 2014;1:e32–e40.12.Cohen MS, Chen YQ, McCauley M, et al. Prevention of HIV-1 Infection with Early Antiretroviral Therapy. Prevention of HIV-1 Infection with Early Antiretroviral Therapy 2011;365:493-505.13.Marks G, Crepaz N, and Janssen RS. Estimating sexual transmission of HIV from persons aware and unaware that they are infected with the virus in the USA. Estimating sexual transmission of HIV from persons aware and unaware that they are infected with the virus in the USA 2006;20:1447-50.14.Nakagawa F, Miners A, Smith CJ, et al. Projected Lifetime Healthcare Costs Associated with HIV Infection. Projected Lifetime Healthcare Costs Associated with HIV Infection 2015;10:e0125018.15.Fleishman JA, Yehia BR, Moore RD, et al. The Economic Burden of Late Entry Into Medical Care for Patients With HIV Infection. The Economic Burden of Late Entry Into Medical Care for Patients With HIV Infection 2010;48:1071-1079.16.Krentz HB and Gill MJ. The Direct Medical Costs of Late Presentation (<350/mm(3)) of HIV Infection over a 15-Year Period. The Direct Medical Costs of Late Presentation (<350/mm(3)) of HIV Infection over a 15-Year Period 2012;2012:757135.17.Krause J, Subklew-Sehume F, Kenyon C, et al. Acceptability of HIV self-testing: a systematic literature review. Acceptability of HIV self-testing: a systematic literature review 2013;13:735.18.Frye V, Wilton L, Hirshfied S, et al. \"Just Because It's Out There, People Aren't Going to Use It.\" HIV Self-Testing Among Young, Black MSM, and Transgender Women. \"Just Because It's Out There, People Aren't Going to Use It.\" HIV Self-Testing Among Young, Black MSM, and Transgender Women 2015.19.Figueroa C, Johnson C, Verster A, et al. Attitudes and Acceptability on HIV Self-testing Among Key Populations: A Literature Review. Attitudes and Acceptability on HIV Self-testing Among Key Populations: A Literature Review 2015.20.Bavinton BR, Brown G, Hurley M, et al. Which gay men would increase their frequency of HIV testing with home self-testing? Which gay men would increase their frequency of HIV testing with home self-testing? 2013;17:2084-92.21.Hensen B, Lewis JJ, Schaap A, et al. Factors associated with HIV-testing and acceptance of an offer of home-based testing by men in rural Zambia. Factors associated with HIV-testing and acceptance of an offer of home-based testing by men in rural Zambia 2015;19:492-504.22.Lee VJ, Tan SC, Earnest A, et al. User acceptability and feasibility of self-testing with HIV rapid tests. User acceptability and feasibility of self-testing with HIV rapid tests 2007;45:449-53.23.Terris-Prestholt F, Hanson K, MacPhail C, et al. How much demand for New HIV prevention technologies can we really expect? Results from a discrete choice experiment in South Africa. How much demand for New HIV prevention technologies can we really expect? Results from a discrete choice experiment in South Africa 2013;8:e83193.24.Ostermann J, Njau B, Brown DS, et al. Heterogeneous HIV Testing Preferences in an Urban Setting in Tanzania: Results from a Discrete Choice Experiment. Heterogeneous HIV Testing Preferences in an Urban Setting in Tanzania: Results from a Discrete Choice Experiment 2014;9:e92100.25.Phillips KA, Maddala T, and Johnson FR. Measuring preferences for health care interventions using conjoint analysis: an application to HIV testing. Measuring preferences for health care interventions using conjoint analysis: an application to HIV testing 2002;37:1681-1705.26.Ryan M. Discrete choice experiments in health care. BMJ 2004;328:360-361.27.Terris-Prestholt F, Quaiffe M, and Vickerman P. Parameterising user uptake in economic evaluations: the role of discrete choice experiments. Parameterising user uptake in economic evaluations: the role of discrete choice experiments In press.28.Philip P, Hickson F, Bonell C, et al. Men who have sex with men in Britain: comparison of estimates from a probability sample and community-based surveys. Men who have sex with men in Britain: comparison of estimates from a probability sample and community-based surveys In submission.29.Orme B. Sample size issues for conjoint analysis, in Getting started with conjoint analysis: strategies for product design and pricing research. 2010, Research Publishers LLC: Madison, Wisconson.)

[5. Zablotska I, Holt M, de Wit J, et al. Gay men who are not getting tested for HIV](1.Public Health England. HIV in the United Kingdom: 2014 report. 2014.2.Public Health England. HIV new diagnosis, treatment and care: 2015 report.3.Williamson LM, Dodds JP, Mercey DE, et al. Sexual risk behaviour and knowledge of HIV status among community samples of gay men in the UK. Sexual risk behaviour and knowledge of HIV status among community samples of gay men in the UK 2008;22:1063-70.4.Holt M, Rawstorne P, Wilkinson J, et al. HIV testing, gay community involvement and internet use: social and behavioural correlates of HIV testing among Australian men who have sex with men. HIV testing, gay community involvement and internet use: social and behavioural correlates of HIV testing among Australian men who have sex with men 2012;16:13-22.5.Zablotska I, Holt M, de Wit J, et al. Gay men who are not getting tested for HIV. Gay men who are not getting tested for HIV 2012;16:1887-94.6.Witzel TC, Melendez-Torres GJ, Hickson F, et al. HIV testing history and preferences for future tests among gay men, bisexual men and other MSM in England. HIV testing history and preferences for future tests among gay men, bisexual men and other MSM in England In submission.7.UNSW Australia. 2015. Annual report of trends in behaviour 2015. HIV/AIDS, hepatitis and sexually transmissible infections in Australia [Online]. Available: https://csrh.arts.unsw.edu.au/media/CSRHFile/CSRH_Annual_Report_of_Trends_in_Behaviour_2015.pdf.8.Phillips AN, Cambiano V, Miners A, et al. Potential impact on HIV incidence of higher HIV testing rates and earlier antiretroviral therapy initiation in MSM. Potential impact on HIV incidence of higher HIV testing rates and earlier antiretroviral therapy initiation in MSM 2015;29:1855-62.9.Nakagawa F, Lodwick RK, Smith CJ, et al. Projected life expectancy of people with HIV according to timing of diagnosis. Projected life expectancy of people with HIV according to timing of diagnosis 2012;26:335-43.10.Chadborn TR, Delpech VC, Sabin CA, et al. The late diagnosis and consequent short-term mortality of HIV-infected heterosexuals (England and Wales, 2000–2004). The late diagnosis and consequent short-term mortality of HIV-infected heterosexuals (England and Wales, 2000–2004) 2006;20:2371-2379.11.Miners A, Phillips A, Kreif N, et al. Health-related quality-of-life of people with HIV in the era of combination antiretroviral treatment: a cross-sectional comparison with the general population. Health-related quality-of-life of people with HIV in the era of combination antiretroviral treatment: a cross-sectional comparison with the general population 2014;1:e32–e40.12.Cohen MS, Chen YQ, McCauley M, et al. Prevention of HIV-1 Infection with Early Antiretroviral Therapy. Prevention of HIV-1 Infection with Early Antiretroviral Therapy 2011;365:493-505.13.Marks G, Crepaz N, and Janssen RS. Estimating sexual transmission of HIV from persons aware and unaware that they are infected with the virus in the USA. Estimating sexual transmission of HIV from persons aware and unaware that they are infected with the virus in the USA 2006;20:1447-50.14.Nakagawa F, Miners A, Smith CJ, et al. Projected Lifetime Healthcare Costs Associated with HIV Infection. Projected Lifetime Healthcare Costs Associated with HIV Infection 2015;10:e0125018.15.Fleishman JA, Yehia BR, Moore RD, et al. The Economic Burden of Late Entry Into Medical Care for Patients With HIV Infection. The Economic Burden of Late Entry Into Medical Care for Patients With HIV Infection 2010;48:1071-1079.16.Krentz HB and Gill MJ. The Direct Medical Costs of Late Presentation (<350/mm(3)) of HIV Infection over a 15-Year Period. The Direct Medical Costs of Late Presentation (<350/mm(3)) of HIV Infection over a 15-Year Period 2012;2012:757135.17.Krause J, Subklew-Sehume F, Kenyon C, et al. Acceptability of HIV self-testing: a systematic literature review. Acceptability of HIV self-testing: a systematic literature review 2013;13:735.18.Frye V, Wilton L, Hirshfied S, et al. \"Just Because It's Out There, People Aren't Going to Use It.\" HIV Self-Testing Among Young, Black MSM, and Transgender Women. \"Just Because It's Out There, People Aren't Going to Use It.\" HIV Self-Testing Among Young, Black MSM, and Transgender Women 2015.19.Figueroa C, Johnson C, Verster A, et al. Attitudes and Acceptability on HIV Self-testing Among Key Populations: A Literature Review. Attitudes and Acceptability on HIV Self-testing Among Key Populations: A Literature Review 2015.20.Bavinton BR, Brown G, Hurley M, et al. Which gay men would increase their frequency of HIV testing with home self-testing? Which gay men would increase their frequency of HIV testing with home self-testing? 2013;17:2084-92.21.Hensen B, Lewis JJ, Schaap A, et al. Factors associated with HIV-testing and acceptance of an offer of home-based testing by men in rural Zambia. Factors associated with HIV-testing and acceptance of an offer of home-based testing by men in rural Zambia 2015;19:492-504.22.Lee VJ, Tan SC, Earnest A, et al. User acceptability and feasibility of self-testing with HIV rapid tests. User acceptability and feasibility of self-testing with HIV rapid tests 2007;45:449-53.23.Terris-Prestholt F, Hanson K, MacPhail C, et al. How much demand for New HIV prevention technologies can we really expect? Results from a discrete choice experiment in South Africa. How much demand for New HIV prevention technologies can we really expect? Results from a discrete choice experiment in South Africa 2013;8:e83193.24.Ostermann J, Njau B, Brown DS, et al. Heterogeneous HIV Testing Preferences in an Urban Setting in Tanzania: Results from a Discrete Choice Experiment. Heterogeneous HIV Testing Preferences in an Urban Setting in Tanzania: Results from a Discrete Choice Experiment 2014;9:e92100.25.Phillips KA, Maddala T, and Johnson FR. Measuring preferences for health care interventions using conjoint analysis: an application to HIV testing. Measuring preferences for health care interventions using conjoint analysis: an application to HIV testing 2002;37:1681-1705.26.Ryan M. Discrete choice experiments in health care. BMJ 2004;328:360-361.27.Terris-Prestholt F, Quaiffe M, and Vickerman P. Parameterising user uptake in economic evaluations: the role of discrete choice experiments. Parameterising user uptake in economic evaluations: the role of discrete choice experiments In press.28.Philip P, Hickson F, Bonell C, et al. Men who have sex with men in Britain: comparison of estimates from a probability sample and community-based surveys. Men who have sex with men in Britain: comparison of estimates from a probability sample and community-based surveys In submission.29.Orme B. Sample size issues for conjoint analysis, in Getting started with conjoint analysis: strategies for product design and pricing research. 2010, Research Publishers LLC: Madison, Wisconson.)*[.](1.Public Health England. HIV in the United Kingdom: 2014 report. 2014.2.Public Health England. HIV new diagnosis, treatment and care: 2015 report.3.Williamson LM, Dodds JP, Mercey DE, et al. Sexual risk behaviour and knowledge of HIV status among community samples of gay men in the UK. Sexual risk behaviour and knowledge of HIV status among community samples of gay men in the UK 2008;22:1063-70.4.Holt M, Rawstorne P, Wilkinson J, et al. HIV testing, gay community involvement and internet use: social and behavioural correlates of HIV testing among Australian men who have sex with men. HIV testing, gay community involvement and internet use: social and behavioural correlates of HIV testing among Australian men who have sex with men 2012;16:13-22.5.Zablotska I, Holt M, de Wit J, et al. Gay men who are not getting tested for HIV. Gay men who are not getting tested for HIV 2012;16:1887-94.6.Witzel TC, Melendez-Torres GJ, Hickson F, et al. HIV testing history and preferences for future tests among gay men, bisexual men and other MSM in England. HIV testing history and preferences for future tests among gay men, bisexual men and other MSM in England In submission.7.UNSW Australia. 2015. Annual report of trends in behaviour 2015. HIV/AIDS, hepatitis and sexually transmissible infections in Australia [Online]. Available: https://csrh.arts.unsw.edu.au/media/CSRHFile/CSRH_Annual_Report_of_Trends_in_Behaviour_2015.pdf.8.Phillips AN, Cambiano V, Miners A, et al. Potential impact on HIV incidence of higher HIV testing rates and earlier antiretroviral therapy initiation in MSM. Potential impact on HIV incidence of higher HIV testing rates and earlier antiretroviral therapy initiation in MSM 2015;29:1855-62.9.Nakagawa F, Lodwick RK, Smith CJ, et al. Projected life expectancy of people with HIV according to timing of diagnosis. Projected life expectancy of people with HIV according to timing of diagnosis 2012;26:335-43.10.Chadborn TR, Delpech VC, Sabin CA, et al. The late diagnosis and consequent short-term mortality of HIV-infected heterosexuals (England and Wales, 2000–2004). The late diagnosis and consequent short-term mortality of HIV-infected heterosexuals (England and Wales, 2000–2004) 2006;20:2371-2379.11.Miners A, Phillips A, Kreif N, et al. Health-related quality-of-life of people with HIV in the era of combination antiretroviral treatment: a cross-sectional comparison with the general population. Health-related quality-of-life of people with HIV in the era of combination antiretroviral treatment: a cross-sectional comparison with the general population 2014;1:e32–e40.12.Cohen MS, Chen YQ, McCauley M, et al. Prevention of HIV-1 Infection with Early Antiretroviral Therapy. Prevention of HIV-1 Infection with Early Antiretroviral Therapy 2011;365:493-505.13.Marks G, Crepaz N, and Janssen RS. Estimating sexual transmission of HIV from persons aware and unaware that they are infected with the virus in the USA. Estimating sexual transmission of HIV from persons aware and unaware that they are infected with the virus in the USA 2006;20:1447-50.14.Nakagawa F, Miners A, Smith CJ, et al. Projected Lifetime Healthcare Costs Associated with HIV Infection. Projected Lifetime Healthcare Costs Associated with HIV Infection 2015;10:e0125018.15.Fleishman JA, Yehia BR, Moore RD, et al. The Economic Burden of Late Entry Into Medical Care for Patients With HIV Infection. The Economic Burden of Late Entry Into Medical Care for Patients With HIV Infection 2010;48:1071-1079.16.Krentz HB and Gill MJ. The Direct Medical Costs of Late Presentation (<350/mm(3)) of HIV Infection over a 15-Year Period. The Direct Medical Costs of Late Presentation (<350/mm(3)) of HIV Infection over a 15-Year Period 2012;2012:757135.17.Krause J, Subklew-Sehume F, Kenyon C, et al. Acceptability of HIV self-testing: a systematic literature review. Acceptability of HIV self-testing: a systematic literature review 2013;13:735.18.Frye V, Wilton L, Hirshfied S, et al. \"Just Because It's Out There, People Aren't Going to Use It.\" HIV Self-Testing Among Young, Black MSM, and Transgender Women. \"Just Because It's Out There, People Aren't Going to Use It.\" HIV Self-Testing Among Young, Black MSM, and Transgender Women 2015.19.Figueroa C, Johnson C, Verster A, et al. Attitudes and Acceptability on HIV Self-testing Among Key Populations: A Literature Review. Attitudes and Acceptability on HIV Self-testing Among Key Populations: A Literature Review 2015.20.Bavinton BR, Brown G, Hurley M, et al. Which gay men would increase their frequency of HIV testing with home self-testing? Which gay men would increase their frequency of HIV testing with home self-testing? 2013;17:2084-92.21.Hensen B, Lewis JJ, Schaap A, et al. Factors associated with HIV-testing and acceptance of an offer of home-based testing by men in rural Zambia. Factors associated with HIV-testing and acceptance of an offer of home-based testing by men in rural Zambia 2015;19:492-504.22.Lee VJ, Tan SC, Earnest A, et al. User acceptability and feasibility of self-testing with HIV rapid tests. User acceptability and feasibility of self-testing with HIV rapid tests 2007;45:449-53.23.Terris-Prestholt F, Hanson K, MacPhail C, et al. How much demand for New HIV prevention technologies can we really expect? Results from a discrete choice experiment in South Africa. How much demand for New HIV prevention technologies can we really expect? Results from a discrete choice experiment in South Africa 2013;8:e83193.24.Ostermann J, Njau B, Brown DS, et al. Heterogeneous HIV Testing Preferences in an Urban Setting in Tanzania: Results from a Discrete Choice Experiment. Heterogeneous HIV Testing Preferences in an Urban Setting in Tanzania: Results from a Discrete Choice Experiment 2014;9:e92100.25.Phillips KA, Maddala T, and Johnson FR. Measuring preferences for health care interventions using conjoint analysis: an application to HIV testing. Measuring preferences for health care interventions using conjoint analysis: an application to HIV testing 2002;37:1681-1705.26.Ryan M. Discrete choice experiments in health care. BMJ 2004;328:360-361.27.Terris-Prestholt F, Quaiffe M, and Vickerman P. Parameterising user uptake in economic evaluations: the role of discrete choice experiments. Parameterising user uptake in economic evaluations: the role of discrete choice experiments In press.28.Philip P, Hickson F, Bonell C, et al. Men who have sex with men in Britain: comparison of estimates from a probability sample and community-based surveys. Men who have sex with men in Britain: comparison of estimates from a probability sample and community-based surveys In submission.29.Orme B. Sample size issues for conjoint analysis, in Getting started with conjoint analysis: strategies for product design and pricing research. 2010, Research Publishers LLC: Madison, Wisconson.)**[Gay men who are not getting tested for HIV](1.Public Health England. HIV in the United Kingdom: 2014 report. 2014.2.Public Health England. HIV new diagnosis, treatment and care: 2015 report.3.Williamson LM, Dodds JP, Mercey DE, et al. Sexual risk behaviour and knowledge of HIV status among community samples of gay men in the UK. Sexual risk behaviour and knowledge of HIV status among community samples of gay men in the UK 2008;22:1063-70.4.Holt M, Rawstorne P, Wilkinson J, et al. HIV testing, gay community involvement and internet use: social and behavioural correlates of HIV testing among Australian men who have sex with men. HIV testing, gay community involvement and internet use: social and behavioural correlates of HIV testing among Australian men who have sex with men 2012;16:13-22.5.Zablotska I, Holt M, de Wit J, et al. Gay men who are not getting tested for HIV. Gay men who are not getting tested for HIV 2012;16:1887-94.6.Witzel TC, Melendez-Torres GJ, Hickson F, et al. HIV testing history and preferences for future tests among gay men, bisexual men and other MSM in England. HIV testing history and preferences for future tests among gay men, bisexual men and other MSM in England In submission.7.UNSW Australia. 2015. Annual report of trends in behaviour 2015. HIV/AIDS, hepatitis and sexually transmissible infections in Australia [Online]. Available: https://csrh.arts.unsw.edu.au/media/CSRHFile/CSRH_Annual_Report_of_Trends_in_Behaviour_2015.pdf.8.Phillips AN, Cambiano V, Miners A, et al. Potential impact on HIV incidence of higher HIV testing rates and earlier antiretroviral therapy initiation in MSM. Potential impact on HIV incidence of higher HIV testing rates and earlier antiretroviral therapy initiation in MSM 2015;29:1855-62.9.Nakagawa F, Lodwick RK, Smith CJ, et al. Projected life expectancy of people with HIV according to timing of diagnosis. Projected life expectancy of people with HIV according to timing of diagnosis 2012;26:335-43.10.Chadborn TR, Delpech VC, Sabin CA, et al. The late diagnosis and consequent short-term mortality of HIV-infected heterosexuals (England and Wales, 2000–2004). The late diagnosis and consequent short-term mortality of HIV-infected heterosexuals (England and Wales, 2000–2004) 2006;20:2371-2379.11.Miners A, Phillips A, Kreif N, et al. Health-related quality-of-life of people with HIV in the era of combination antiretroviral treatment: a cross-sectional comparison with the general population. Health-related quality-of-life of people with HIV in the era of combination antiretroviral treatment: a cross-sectional comparison with the general population 2014;1:e32–e40.12.Cohen MS, Chen YQ, McCauley M, et al. Prevention of HIV-1 Infection with Early Antiretroviral Therapy. Prevention of HIV-1 Infection with Early Antiretroviral Therapy 2011;365:493-505.13.Marks G, Crepaz N, and Janssen RS. Estimating sexual transmission of HIV from persons aware and unaware that they are infected with the virus in the USA. Estimating sexual transmission of HIV from persons aware and unaware that they are infected with the virus in the USA 2006;20:1447-50.14.Nakagawa F, Miners A, Smith CJ, et al. Projected Lifetime Healthcare Costs Associated with HIV Infection. Projected Lifetime Healthcare Costs Associated with HIV Infection 2015;10:e0125018.15.Fleishman JA, Yehia BR, Moore RD, et al. The Economic Burden of Late Entry Into Medical Care for Patients With HIV Infection. The Economic Burden of Late Entry Into Medical Care for Patients With HIV Infection 2010;48:1071-1079.16.Krentz HB and Gill MJ. The Direct Medical Costs of Late Presentation (<350/mm(3)) of HIV Infection over a 15-Year Period. The Direct Medical Costs of Late Presentation (<350/mm(3)) of HIV Infection over a 15-Year Period 2012;2012:757135.17.Krause J, Subklew-Sehume F, Kenyon C, et al. Acceptability of HIV self-testing: a systematic literature review. Acceptability of HIV self-testing: a systematic literature review 2013;13:735.18.Frye V, Wilton L, Hirshfied S, et al. \"Just Because It's Out There, People Aren't Going to Use It.\" HIV Self-Testing Among Young, Black MSM, and Transgender Women. \"Just Because It's Out There, People Aren't Going to Use It.\" HIV Self-Testing Among Young, Black MSM, and Transgender Women 2015.19.Figueroa C, Johnson C, Verster A, et al. Attitudes and Acceptability on HIV Self-testing Among Key Populations: A Literature Review. Attitudes and Acceptability on HIV Self-testing Among Key Populations: A Literature Review 2015.20.Bavinton BR, Brown G, Hurley M, et al. Which gay men would increase their frequency of HIV testing with home self-testing? Which gay men would increase their frequency of HIV testing with home self-testing? 2013;17:2084-92.21.Hensen B, Lewis JJ, Schaap A, et al. Factors associated with HIV-testing and acceptance of an offer of home-based testing by men in rural Zambia. Factors associated with HIV-testing and acceptance of an offer of home-based testing by men in rural Zambia 2015;19:492-504.22.Lee VJ, Tan SC, Earnest A, et al. User acceptability and feasibility of self-testing with HIV rapid tests. User acceptability and feasibility of self-testing with HIV rapid tests 2007;45:449-53.23.Terris-Prestholt F, Hanson K, MacPhail C, et al. How much demand for New HIV prevention technologies can we really expect? Results from a discrete choice experiment in South Africa. How much demand for New HIV prevention technologies can we really expect? Results from a discrete choice experiment in South Africa 2013;8:e83193.24.Ostermann J, Njau B, Brown DS, et al. Heterogeneous HIV Testing Preferences in an Urban Setting in Tanzania: Results from a Discrete Choice Experiment. Heterogeneous HIV Testing Preferences in an Urban Setting in Tanzania: Results from a Discrete Choice Experiment 2014;9:e92100.25.Phillips KA, Maddala T, and Johnson FR. Measuring preferences for health care interventions using conjoint analysis: an application to HIV testing. Measuring preferences for health care interventions using conjoint analysis: an application to HIV testing 2002;37:1681-1705.26.Ryan M. Discrete choice experiments in health care. BMJ 2004;328:360-361.27.Terris-Prestholt F, Quaiffe M, and Vickerman P. Parameterising user uptake in economic evaluations: the role of discrete choice experiments. Parameterising user uptake in economic evaluations: the role of discrete choice experiments In press.28.Philip P, Hickson F, Bonell C, et al. Men who have sex with men in Britain: comparison of estimates from a probability sample and community-based surveys. Men who have sex with men in Britain: comparison of estimates from a probability sample and community-based surveys In submission.29.Orme B. Sample size issues for conjoint analysis, in Getting started with conjoint analysis: strategies for product design and pricing research. 2010, Research Publishers LLC: Madison, Wisconson.)* [2012;](1.Public Health England. HIV in the United Kingdom: 2014 report. 2014.2.Public Health England. HIV new diagnosis, treatment and care: 2015 report.3.Williamson LM, Dodds JP, Mercey DE, et al. Sexual risk behaviour and knowledge of HIV status among community samples of gay men in the UK. Sexual risk behaviour and knowledge of HIV status among community samples of gay men in the UK 2008;22:1063-70.4.Holt M, Rawstorne P, Wilkinson J, et al. HIV testing, gay community involvement and internet use: social and behavioural correlates of HIV testing among Australian men who have sex with men. HIV testing, gay community involvement and internet use: social and behavioural correlates of HIV testing among Australian men who have sex with men 2012;16:13-22.5.Zablotska I, Holt M, de Wit J, et al. Gay men who are not getting tested for HIV. Gay men who are not getting tested for HIV 2012;16:1887-94.6.Witzel TC, Melendez-Torres GJ, Hickson F, et al. HIV testing history and preferences for future tests among gay men, bisexual men and other MSM in England. HIV testing history and preferences for future tests among gay men, bisexual men and other MSM in England In submission.7.UNSW Australia. 2015. Annual report of trends in behaviour 2015. HIV/AIDS, hepatitis and sexually transmissible infections in Australia [Online]. Available: https://csrh.arts.unsw.edu.au/media/CSRHFile/CSRH_Annual_Report_of_Trends_in_Behaviour_2015.pdf.8.Phillips AN, Cambiano V, Miners A, et al. Potential impact on HIV incidence of higher HIV testing rates and earlier antiretroviral therapy initiation in MSM. Potential impact on HIV incidence of higher HIV testing rates and earlier antiretroviral therapy initiation in MSM 2015;29:1855-62.9.Nakagawa F, Lodwick RK, Smith CJ, et al. Projected life expectancy of people with HIV according to timing of diagnosis. Projected life expectancy of people with HIV according to timing of diagnosis 2012;26:335-43.10.Chadborn TR, Delpech VC, Sabin CA, et al. The late diagnosis and consequent short-term mortality of HIV-infected heterosexuals (England and Wales, 2000–2004). The late diagnosis and consequent short-term mortality of HIV-infected heterosexuals (England and Wales, 2000–2004) 2006;20:2371-2379.11.Miners A, Phillips A, Kreif N, et al. Health-related quality-of-life of people with HIV in the era of combination antiretroviral treatment: a cross-sectional comparison with the general population. Health-related quality-of-life of people with HIV in the era of combination antiretroviral treatment: a cross-sectional comparison with the general population 2014;1:e32–e40.12.Cohen MS, Chen YQ, McCauley M, et al. Prevention of HIV-1 Infection with Early Antiretroviral Therapy. Prevention of HIV-1 Infection with Early Antiretroviral Therapy 2011;365:493-505.13.Marks G, Crepaz N, and Janssen RS. Estimating sexual transmission of HIV from persons aware and unaware that they are infected with the virus in the USA. Estimating sexual transmission of HIV from persons aware and unaware that they are infected with the virus in the USA 2006;20:1447-50.14.Nakagawa F, Miners A, Smith CJ, et al. Projected Lifetime Healthcare Costs Associated with HIV Infection. Projected Lifetime Healthcare Costs Associated with HIV Infection 2015;10:e0125018.15.Fleishman JA, Yehia BR, Moore RD, et al. The Economic Burden of Late Entry Into Medical Care for Patients With HIV Infection. The Economic Burden of Late Entry Into Medical Care for Patients With HIV Infection 2010;48:1071-1079.16.Krentz HB and Gill MJ. The Direct Medical Costs of Late Presentation (<350/mm(3)) of HIV Infection over a 15-Year Period. The Direct Medical Costs of Late Presentation (<350/mm(3)) of HIV Infection over a 15-Year Period 2012;2012:757135.17.Krause J, Subklew-Sehume F, Kenyon C, et al. Acceptability of HIV self-testing: a systematic literature review. Acceptability of HIV self-testing: a systematic literature review 2013;13:735.18.Frye V, Wilton L, Hirshfied S, et al. \"Just Because It's Out There, People Aren't Going to Use It.\" HIV Self-Testing Among Young, Black MSM, and Transgender Women. \"Just Because It's Out There, People Aren't Going to Use It.\" HIV Self-Testing Among Young, Black MSM, and Transgender Women 2015.19.Figueroa C, Johnson C, Verster A, et al. Attitudes and Acceptability on HIV Self-testing Among Key Populations: A Literature Review. Attitudes and Acceptability on HIV Self-testing Among Key Populations: A Literature Review 2015.20.Bavinton BR, Brown G, Hurley M, et al. Which gay men would increase their frequency of HIV testing with home self-testing? Which gay men would increase their frequency of HIV testing with home self-testing? 2013;17:2084-92.21.Hensen B, Lewis JJ, Schaap A, et al. Factors associated with HIV-testing and acceptance of an offer of home-based testing by men in rural Zambia. Factors associated with HIV-testing and acceptance of an offer of home-based testing by men in rural Zambia 2015;19:492-504.22.Lee VJ, Tan SC, Earnest A, et al. User acceptability and feasibility of self-testing with HIV rapid tests. User acceptability and feasibility of self-testing with HIV rapid tests 2007;45:449-53.23.Terris-Prestholt F, Hanson K, MacPhail C, et al. How much demand for New HIV prevention technologies can we really expect? Results from a discrete choice experiment in South Africa. How much demand for New HIV prevention technologies can we really expect? Results from a discrete choice experiment in South Africa 2013;8:e83193.24.Ostermann J, Njau B, Brown DS, et al. Heterogeneous HIV Testing Preferences in an Urban Setting in Tanzania: Results from a Discrete Choice Experiment. Heterogeneous HIV Testing Preferences in an Urban Setting in Tanzania: Results from a Discrete Choice Experiment 2014;9:e92100.25.Phillips KA, Maddala T, and Johnson FR. Measuring preferences for health care interventions using conjoint analysis: an application to HIV testing. Measuring preferences for health care interventions using conjoint analysis: an application to HIV testing 2002;37:1681-1705.26.Ryan M. Discrete choice experiments in health care. BMJ 2004;328:360-361.27.Terris-Prestholt F, Quaiffe M, and Vickerman P. Parameterising user uptake in economic evaluations: the role of discrete choice experiments. Parameterising user uptake in economic evaluations: the role of discrete choice experiments In press.28.Philip P, Hickson F, Bonell C, et al. Men who have sex with men in Britain: comparison of estimates from a probability sample and community-based surveys. Men who have sex with men in Britain: comparison of estimates from a probability sample and community-based surveys In submission.29.Orme B. Sample size issues for conjoint analysis, in Getting started with conjoint analysis: strategies for product design and pricing research. 2010, Research Publishers LLC: Madison, Wisconson.)**[16](1.Public Health England. HIV in the United Kingdom: 2014 report. 2014.2.Public Health England. HIV new diagnosis, treatment and care: 2015 report.3.Williamson LM, Dodds JP, Mercey DE, et al. Sexual risk behaviour and knowledge of HIV status among community samples of gay men in the UK. Sexual risk behaviour and knowledge of HIV status among community samples of gay men in the UK 2008;22:1063-70.4.Holt M, Rawstorne P, Wilkinson J, et al. HIV testing, gay community involvement and internet use: social and behavioural correlates of HIV testing among Australian men who have sex with men. HIV testing, gay community involvement and internet use: social and behavioural correlates of HIV testing among Australian men who have sex with men 2012;16:13-22.5.Zablotska I, Holt M, de Wit J, et al. Gay men who are not getting tested for HIV. Gay men who are not getting tested for HIV 2012;16:1887-94.6.Witzel TC, Melendez-Torres GJ, Hickson F, et al. HIV testing history and preferences for future tests among gay men, bisexual men and other MSM in England. HIV testing history and preferences for future tests among gay men, bisexual men and other MSM in England In submission.7.UNSW Australia. 2015. Annual report of trends in behaviour 2015. HIV/AIDS, hepatitis and sexually transmissible infections in Australia [Online]. Available: https://csrh.arts.unsw.edu.au/media/CSRHFile/CSRH_Annual_Report_of_Trends_in_Behaviour_2015.pdf.8.Phillips AN, Cambiano V, Miners A, et al. Potential impact on HIV incidence of higher HIV testing rates and earlier antiretroviral therapy initiation in MSM. Potential impact on HIV incidence of higher HIV testing rates and earlier antiretroviral therapy initiation in MSM 2015;29:1855-62.9.Nakagawa F, Lodwick RK, Smith CJ, et al. Projected life expectancy of people with HIV according to timing of diagnosis. Projected life expectancy of people with HIV according to timing of diagnosis 2012;26:335-43.10.Chadborn TR, Delpech VC, Sabin CA, et al. The late diagnosis and consequent short-term mortality of HIV-infected heterosexuals (England and Wales, 2000–2004). The late diagnosis and consequent short-term mortality of HIV-infected heterosexuals (England and Wales, 2000–2004) 2006;20:2371-2379.11.Miners A, Phillips A, Kreif N, et al. Health-related quality-of-life of people with HIV in the era of combination antiretroviral treatment: a cross-sectional comparison with the general population. Health-related quality-of-life of people with HIV in the era of combination antiretroviral treatment: a cross-sectional comparison with the general population 2014;1:e32–e40.12.Cohen MS, Chen YQ, McCauley M, et al. Prevention of HIV-1 Infection with Early Antiretroviral Therapy. Prevention of HIV-1 Infection with Early Antiretroviral Therapy 2011;365:493-505.13.Marks G, Crepaz N, and Janssen RS. Estimating sexual transmission of HIV from persons aware and unaware that they are infected with the virus in the USA. Estimating sexual transmission of HIV from persons aware and unaware that they are infected with the virus in the USA 2006;20:1447-50.14.Nakagawa F, Miners A, Smith CJ, et al. Projected Lifetime Healthcare Costs Associated with HIV Infection. Projected Lifetime Healthcare Costs Associated with HIV Infection 2015;10:e0125018.15.Fleishman JA, Yehia BR, Moore RD, et al. The Economic Burden of Late Entry Into Medical Care for Patients With HIV Infection. The Economic Burden of Late Entry Into Medical Care for Patients With HIV Infection 2010;48:1071-1079.16.Krentz HB and Gill MJ. The Direct Medical Costs of Late Presentation (<350/mm(3)) of HIV Infection over a 15-Year Period. The Direct Medical Costs of Late Presentation (<350/mm(3)) of HIV Infection over a 15-Year Period 2012;2012:757135.17.Krause J, Subklew-Sehume F, Kenyon C, et al. Acceptability of HIV self-testing: a systematic literature review. Acceptability of HIV self-testing: a systematic literature review 2013;13:735.18.Frye V, Wilton L, Hirshfied S, et al. \"Just Because It's Out There, People Aren't Going to Use It.\" HIV Self-Testing Among Young, Black MSM, and Transgender Women. \"Just Because It's Out There, People Aren't Going to Use It.\" HIV Self-Testing Among Young, Black MSM, and Transgender Women 2015.19.Figueroa C, Johnson C, Verster A, et al. Attitudes and Acceptability on HIV Self-testing Among Key Populations: A Literature Review. Attitudes and Acceptability on HIV Self-testing Among Key Populations: A Literature Review 2015.20.Bavinton BR, Brown G, Hurley M, et al. Which gay men would increase their frequency of HIV testing with home self-testing? Which gay men would increase their frequency of HIV testing with home self-testing? 2013;17:2084-92.21.Hensen B, Lewis JJ, Schaap A, et al. Factors associated with HIV-testing and acceptance of an offer of home-based testing by men in rural Zambia. Factors associated with HIV-testing and acceptance of an offer of home-based testing by men in rural Zambia 2015;19:492-504.22.Lee VJ, Tan SC, Earnest A, et al. User acceptability and feasibility of self-testing with HIV rapid tests. User acceptability and feasibility of self-testing with HIV rapid tests 2007;45:449-53.23.Terris-Prestholt F, Hanson K, MacPhail C, et al. How much demand for New HIV prevention technologies can we really expect? Results from a discrete choice experiment in South Africa. How much demand for New HIV prevention technologies can we really expect? Results from a discrete choice experiment in South Africa 2013;8:e83193.24.Ostermann J, Njau B, Brown DS, et al. Heterogeneous HIV Testing Preferences in an Urban Setting in Tanzania: Results from a Discrete Choice Experiment. Heterogeneous HIV Testing Preferences in an Urban Setting in Tanzania: Results from a Discrete Choice Experiment 2014;9:e92100.25.Phillips KA, Maddala T, and Johnson FR. Measuring preferences for health care interventions using conjoint analysis: an application to HIV testing. Measuring preferences for health care interventions using conjoint analysis: an application to HIV testing 2002;37:1681-1705.26.Ryan M. Discrete choice experiments in health care. BMJ 2004;328:360-361.27.Terris-Prestholt F, Quaiffe M, and Vickerman P. Parameterising user uptake in economic evaluations: the role of discrete choice experiments. Parameterising user uptake in economic evaluations: the role of discrete choice experiments In press.28.Philip P, Hickson F, Bonell C, et al. Men who have sex with men in Britain: comparison of estimates from a probability sample and community-based surveys. Men who have sex with men in Britain: comparison of estimates from a probability sample and community-based surveys In submission.29.Orme B. Sample size issues for conjoint analysis, in Getting started with conjoint analysis: strategies for product design and pricing research. 2010, Research Publishers LLC: Madison, Wisconson.)**[:1887-94.](1.Public Health England. HIV in the United Kingdom: 2014 report. 2014.2.Public Health England. HIV new diagnosis, treatment and care: 2015 report.3.Williamson LM, Dodds JP, Mercey DE, et al. Sexual risk behaviour and knowledge of HIV status among community samples of gay men in the UK. Sexual risk behaviour and knowledge of HIV status among community samples of gay men in the UK 2008;22:1063-70.4.Holt M, Rawstorne P, Wilkinson J, et al. HIV testing, gay community involvement and internet use: social and behavioural correlates of HIV testing among Australian men who have sex with men. HIV testing, gay community involvement and internet use: social and behavioural correlates of HIV testing among Australian men who have sex with men 2012;16:13-22.5.Zablotska I, Holt M, de Wit J, et al. Gay men who are not getting tested for HIV. Gay men who are not getting tested for HIV 2012;16:1887-94.6.Witzel TC, Melendez-Torres GJ, Hickson F, et al. HIV testing history and preferences for future tests among gay men, bisexual men and other MSM in England. HIV testing history and preferences for future tests among gay men, bisexual men and other MSM in England In submission.7.UNSW Australia. 2015. Annual report of trends in behaviour 2015. HIV/AIDS, hepatitis and sexually transmissible infections in Australia [Online]. Available: https://csrh.arts.unsw.edu.au/media/CSRHFile/CSRH_Annual_Report_of_Trends_in_Behaviour_2015.pdf.8.Phillips AN, Cambiano V, Miners A, et al. Potential impact on HIV incidence of higher HIV testing rates and earlier antiretroviral therapy initiation in MSM. Potential impact on HIV incidence of higher HIV testing rates and earlier antiretroviral therapy initiation in MSM 2015;29:1855-62.9.Nakagawa F, Lodwick RK, Smith CJ, et al. Projected life expectancy of people with HIV according to timing of diagnosis. Projected life expectancy of people with HIV according to timing of diagnosis 2012;26:335-43.10.Chadborn TR, Delpech VC, Sabin CA, et al. The late diagnosis and consequent short-term mortality of HIV-infected heterosexuals (England and Wales, 2000–2004). The late diagnosis and consequent short-term mortality of HIV-infected heterosexuals (England and Wales, 2000–2004) 2006;20:2371-2379.11.Miners A, Phillips A, Kreif N, et al. Health-related quality-of-life of people with HIV in the era of combination antiretroviral treatment: a cross-sectional comparison with the general population. Health-related quality-of-life of people with HIV in the era of combination antiretroviral treatment: a cross-sectional comparison with the general population 2014;1:e32–e40.12.Cohen MS, Chen YQ, McCauley M, et al. Prevention of HIV-1 Infection with Early Antiretroviral Therapy. Prevention of HIV-1 Infection with Early Antiretroviral Therapy 2011;365:493-505.13.Marks G, Crepaz N, and Janssen RS. Estimating sexual transmission of HIV from persons aware and unaware that they are infected with the virus in the USA. Estimating sexual transmission of HIV from persons aware and unaware that they are infected with the virus in the USA 2006;20:1447-50.14.Nakagawa F, Miners A, Smith CJ, et al. Projected Lifetime Healthcare Costs Associated with HIV Infection. Projected Lifetime Healthcare Costs Associated with HIV Infection 2015;10:e0125018.15.Fleishman JA, Yehia BR, Moore RD, et al. The Economic Burden of Late Entry Into Medical Care for Patients With HIV Infection. The Economic Burden of Late Entry Into Medical Care for Patients With HIV Infection 2010;48:1071-1079.16.Krentz HB and Gill MJ. The Direct Medical Costs of Late Presentation (<350/mm(3)) of HIV Infection over a 15-Year Period. The Direct Medical Costs of Late Presentation (<350/mm(3)) of HIV Infection over a 15-Year Period 2012;2012:757135.17.Krause J, Subklew-Sehume F, Kenyon C, et al. Acceptability of HIV self-testing: a systematic literature review. Acceptability of HIV self-testing: a systematic literature review 2013;13:735.18.Frye V, Wilton L, Hirshfied S, et al. \"Just Because It's Out There, People Aren't Going to Use It.\" HIV Self-Testing Among Young, Black MSM, and Transgender Women. \"Just Because It's Out There, People Aren't Going to Use It.\" HIV Self-Testing Among Young, Black MSM, and Transgender Women 2015.19.Figueroa C, Johnson C, Verster A, et al. Attitudes and Acceptability on HIV Self-testing Among Key Populations: A Literature Review. Attitudes and Acceptability on HIV Self-testing Among Key Populations: A Literature Review 2015.20.Bavinton BR, Brown G, Hurley M, et al. Which gay men would increase their frequency of HIV testing with home self-testing? Which gay men would increase their frequency of HIV testing with home self-testing? 2013;17:2084-92.21.Hensen B, Lewis JJ, Schaap A, et al. Factors associated with HIV-testing and acceptance of an offer of home-based testing by men in rural Zambia. Factors associated with HIV-testing and acceptance of an offer of home-based testing by men in rural Zambia 2015;19:492-504.22.Lee VJ, Tan SC, Earnest A, et al. User acceptability and feasibility of self-testing with HIV rapid tests. User acceptability and feasibility of self-testing with HIV rapid tests 2007;45:449-53.23.Terris-Prestholt F, Hanson K, MacPhail C, et al. How much demand for New HIV prevention technologies can we really expect? Results from a discrete choice experiment in South Africa. How much demand for New HIV prevention technologies can we really expect? Results from a discrete choice experiment in South Africa 2013;8:e83193.24.Ostermann J, Njau B, Brown DS, et al. Heterogeneous HIV Testing Preferences in an Urban Setting in Tanzania: Results from a Discrete Choice Experiment. Heterogeneous HIV Testing Preferences in an Urban Setting in Tanzania: Results from a Discrete Choice Experiment 2014;9:e92100.25.Phillips KA, Maddala T, and Johnson FR. Measuring preferences for health care interventions using conjoint analysis: an application to HIV testing. Measuring preferences for health care interventions using conjoint analysis: an application to HIV testing 2002;37:1681-1705.26.Ryan M. Discrete choice experiments in health care. BMJ 2004;328:360-361.27.Terris-Prestholt F, Quaiffe M, and Vickerman P. Parameterising user uptake in economic evaluations: the role of discrete choice experiments. Parameterising user uptake in economic evaluations: the role of discrete choice experiments In press.28.Philip P, Hickson F, Bonell C, et al. Men who have sex with men in Britain: comparison of estimates from a probability sample and community-based surveys. Men who have sex with men in Britain: comparison of estimates from a probability sample and community-based surveys In submission.29.Orme B. Sample size issues for conjoint analysis, in Getting started with conjoint analysis: strategies for product design and pricing research. 2010, Research Publishers LLC: Madison, Wisconson.)

[6. Witzel TC, Melendez-Torres GJ, Hickson F, et al. HIV testing history and preferences for future tests among gay men, bisexual men and other MSM in England](1.Public Health England. HIV in the United Kingdom: 2014 report. 2014.2.Public Health England. HIV new diagnosis, treatment and care: 2015 report.3.Williamson LM, Dodds JP, Mercey DE, et al. Sexual risk behaviour and knowledge of HIV status among community samples of gay men in the UK. Sexual risk behaviour and knowledge of HIV status among community samples of gay men in the UK 2008;22:1063-70.4.Holt M, Rawstorne P, Wilkinson J, et al. HIV testing, gay community involvement and internet use: social and behavioural correlates of HIV testing among Australian men who have sex with men. HIV testing, gay community involvement and internet use: social and behavioural correlates of HIV testing among Australian men who have sex with men 2012;16:13-22.5.Zablotska I, Holt M, de Wit J, et al. Gay men who are not getting tested for HIV. Gay men who are not getting tested for HIV 2012;16:1887-94.6.Witzel TC, Melendez-Torres GJ, Hickson F, et al. HIV testing history and preferences for future tests among gay men, bisexual men and other MSM in England. HIV testing history and preferences for future tests among gay men, bisexual men and other MSM in England In submission.7.UNSW Australia. 2015. Annual report of trends in behaviour 2015. HIV/AIDS, hepatitis and sexually transmissible infections in Australia [Online]. Available: https://csrh.arts.unsw.edu.au/media/CSRHFile/CSRH_Annual_Report_of_Trends_in_Behaviour_2015.pdf.8.Phillips AN, Cambiano V, Miners A, et al. Potential impact on HIV incidence of higher HIV testing rates and earlier antiretroviral therapy initiation in MSM. Potential impact on HIV incidence of higher HIV testing rates and earlier antiretroviral therapy initiation in MSM 2015;29:1855-62.9.Nakagawa F, Lodwick RK, Smith CJ, et al. Projected life expectancy of people with HIV according to timing of diagnosis. Projected life expectancy of people with HIV according to timing of diagnosis 2012;26:335-43.10.Chadborn TR, Delpech VC, Sabin CA, et al. The late diagnosis and consequent short-term mortality of HIV-infected heterosexuals (England and Wales, 2000–2004). The late diagnosis and consequent short-term mortality of HIV-infected heterosexuals (England and Wales, 2000–2004) 2006;20:2371-2379.11.Miners A, Phillips A, Kreif N, et al. Health-related quality-of-life of people with HIV in the era of combination antiretroviral treatment: a cross-sectional comparison with the general population. Health-related quality-of-life of people with HIV in the era of combination antiretroviral treatment: a cross-sectional comparison with the general population 2014;1:e32–e40.12.Cohen MS, Chen YQ, McCauley M, et al. Prevention of HIV-1 Infection with Early Antiretroviral Therapy. Prevention of HIV-1 Infection with Early Antiretroviral Therapy 2011;365:493-505.13.Marks G, Crepaz N, and Janssen RS. Estimating sexual transmission of HIV from persons aware and unaware that they are infected with the virus in the USA. Estimating sexual transmission of HIV from persons aware and unaware that they are infected with the virus in the USA 2006;20:1447-50.14.Nakagawa F, Miners A, Smith CJ, et al. Projected Lifetime Healthcare Costs Associated with HIV Infection. Projected Lifetime Healthcare Costs Associated with HIV Infection 2015;10:e0125018.15.Fleishman JA, Yehia BR, Moore RD, et al. The Economic Burden of Late Entry Into Medical Care for Patients With HIV Infection. The Economic Burden of Late Entry Into Medical Care for Patients With HIV Infection 2010;48:1071-1079.16.Krentz HB and Gill MJ. The Direct Medical Costs of Late Presentation (<350/mm(3)) of HIV Infection over a 15-Year Period. The Direct Medical Costs of Late Presentation (<350/mm(3)) of HIV Infection over a 15-Year Period 2012;2012:757135.17.Krause J, Subklew-Sehume F, Kenyon C, et al. Acceptability of HIV self-testing: a systematic literature review. Acceptability of HIV self-testing: a systematic literature review 2013;13:735.18.Frye V, Wilton L, Hirshfied S, et al. \"Just Because It's Out There, People Aren't Going to Use It.\" HIV Self-Testing Among Young, Black MSM, and Transgender Women. \"Just Because It's Out There, People Aren't Going to Use It.\" HIV Self-Testing Among Young, Black MSM, and Transgender Women 2015.19.Figueroa C, Johnson C, Verster A, et al. Attitudes and Acceptability on HIV Self-testing Among Key Populations: A Literature Review. Attitudes and Acceptability on HIV Self-testing Among Key Populations: A Literature Review 2015.20.Bavinton BR, Brown G, Hurley M, et al. Which gay men would increase their frequency of HIV testing with home self-testing? Which gay men would increase their frequency of HIV testing with home self-testing? 2013;17:2084-92.21.Hensen B, Lewis JJ, Schaap A, et al. Factors associated with HIV-testing and acceptance of an offer of home-based testing by men in rural Zambia. Factors associated with HIV-testing and acceptance of an offer of home-based testing by men in rural Zambia 2015;19:492-504.22.Lee VJ, Tan SC, Earnest A, et al. User acceptability and feasibility of self-testing with HIV rapid tests. User acceptability and feasibility of self-testing with HIV rapid tests 2007;45:449-53.23.Terris-Prestholt F, Hanson K, MacPhail C, et al. How much demand for New HIV prevention technologies can we really expect? Results from a discrete choice experiment in South Africa. How much demand for New HIV prevention technologies can we really expect? Results from a discrete choice experiment in South Africa 2013;8:e83193.24.Ostermann J, Njau B, Brown DS, et al. Heterogeneous HIV Testing Preferences in an Urban Setting in Tanzania: Results from a Discrete Choice Experiment. Heterogeneous HIV Testing Preferences in an Urban Setting in Tanzania: Results from a Discrete Choice Experiment 2014;9:e92100.25.Phillips KA, Maddala T, and Johnson FR. Measuring preferences for health care interventions using conjoint analysis: an application to HIV testing. Measuring preferences for health care interventions using conjoint analysis: an application to HIV testing 2002;37:1681-1705.26.Ryan M. Discrete choice experiments in health care. BMJ 2004;328:360-361.27.Terris-Prestholt F, Quaiffe M, and Vickerman P. Parameterising user uptake in economic evaluations: the role of discrete choice experiments. Parameterising user uptake in economic evaluations: the role of discrete choice experiments In press.28.Philip P, Hickson F, Bonell C, et al. Men who have sex with men in Britain: comparison of estimates from a probability sample and community-based surveys. Men who have sex with men in Britain: comparison of estimates from a probability sample and community-based surveys In submission.29.Orme B. Sample size issues for conjoint analysis, in Getting started with conjoint analysis: strategies for product design and pricing research. 2010, Research Publishers LLC: Madison, Wisconson.)*[.](1.Public Health England. HIV in the United Kingdom: 2014 report. 2014.2.Public Health England. HIV new diagnosis, treatment and care: 2015 report.3.Williamson LM, Dodds JP, Mercey DE, et al. Sexual risk behaviour and knowledge of HIV status among community samples of gay men in the UK. Sexual risk behaviour and knowledge of HIV status among community samples of gay men in the UK 2008;22:1063-70.4.Holt M, Rawstorne P, Wilkinson J, et al. HIV testing, gay community involvement and internet use: social and behavioural correlates of HIV testing among Australian men who have sex with men. HIV testing, gay community involvement and internet use: social and behavioural correlates of HIV testing among Australian men who have sex with men 2012;16:13-22.5.Zablotska I, Holt M, de Wit J, et al. Gay men who are not getting tested for HIV. Gay men who are not getting tested for HIV 2012;16:1887-94.6.Witzel TC, Melendez-Torres GJ, Hickson F, et al. HIV testing history and preferences for future tests among gay men, bisexual men and other MSM in England. HIV testing history and preferences for future tests among gay men, bisexual men and other MSM in England In submission.7.UNSW Australia. 2015. Annual report of trends in behaviour 2015. HIV/AIDS, hepatitis and sexually transmissible infections in Australia [Online]. Available: https://csrh.arts.unsw.edu.au/media/CSRHFile/CSRH_Annual_Report_of_Trends_in_Behaviour_2015.pdf.8.Phillips AN, Cambiano V, Miners A, et al. Potential impact on HIV incidence of higher HIV testing rates and earlier antiretroviral therapy initiation in MSM. Potential impact on HIV incidence of higher HIV testing rates and earlier antiretroviral therapy initiation in MSM 2015;29:1855-62.9.Nakagawa F, Lodwick RK, Smith CJ, et al. Projected life expectancy of people with HIV according to timing of diagnosis. Projected life expectancy of people with HIV according to timing of diagnosis 2012;26:335-43.10.Chadborn TR, Delpech VC, Sabin CA, et al. The late diagnosis and consequent short-term mortality of HIV-infected heterosexuals (England and Wales, 2000–2004). The late diagnosis and consequent short-term mortality of HIV-infected heterosexuals (England and Wales, 2000–2004) 2006;20:2371-2379.11.Miners A, Phillips A, Kreif N, et al. Health-related quality-of-life of people with HIV in the era of combination antiretroviral treatment: a cross-sectional comparison with the general population. Health-related quality-of-life of people with HIV in the era of combination antiretroviral treatment: a cross-sectional comparison with the general population 2014;1:e32–e40.12.Cohen MS, Chen YQ, McCauley M, et al. Prevention of HIV-1 Infection with Early Antiretroviral Therapy. Prevention of HIV-1 Infection with Early Antiretroviral Therapy 2011;365:493-505.13.Marks G, Crepaz N, and Janssen RS. Estimating sexual transmission of HIV from persons aware and unaware that they are infected with the virus in the USA. Estimating sexual transmission of HIV from persons aware and unaware that they are infected with the virus in the USA 2006;20:1447-50.14.Nakagawa F, Miners A, Smith CJ, et al. Projected Lifetime Healthcare Costs Associated with HIV Infection. Projected Lifetime Healthcare Costs Associated with HIV Infection 2015;10:e0125018.15.Fleishman JA, Yehia BR, Moore RD, et al. The Economic Burden of Late Entry Into Medical Care for Patients With HIV Infection. The Economic Burden of Late Entry Into Medical Care for Patients With HIV Infection 2010;48:1071-1079.16.Krentz HB and Gill MJ. The Direct Medical Costs of Late Presentation (<350/mm(3)) of HIV Infection over a 15-Year Period. The Direct Medical Costs of Late Presentation (<350/mm(3)) of HIV Infection over a 15-Year Period 2012;2012:757135.17.Krause J, Subklew-Sehume F, Kenyon C, et al. Acceptability of HIV self-testing: a systematic literature review. Acceptability of HIV self-testing: a systematic literature review 2013;13:735.18.Frye V, Wilton L, Hirshfied S, et al. \"Just Because It's Out There, People Aren't Going to Use It.\" HIV Self-Testing Among Young, Black MSM, and Transgender Women. \"Just Because It's Out There, People Aren't Going to Use It.\" HIV Self-Testing Among Young, Black MSM, and Transgender Women 2015.19.Figueroa C, Johnson C, Verster A, et al. Attitudes and Acceptability on HIV Self-testing Among Key Populations: A Literature Review. Attitudes and Acceptability on HIV Self-testing Among Key Populations: A Literature Review 2015.20.Bavinton BR, Brown G, Hurley M, et al. Which gay men would increase their frequency of HIV testing with home self-testing? Which gay men would increase their frequency of HIV testing with home self-testing? 2013;17:2084-92.21.Hensen B, Lewis JJ, Schaap A, et al. Factors associated with HIV-testing and acceptance of an offer of home-based testing by men in rural Zambia. Factors associated with HIV-testing and acceptance of an offer of home-based testing by men in rural Zambia 2015;19:492-504.22.Lee VJ, Tan SC, Earnest A, et al. User acceptability and feasibility of self-testing with HIV rapid tests. User acceptability and feasibility of self-testing with HIV rapid tests 2007;45:449-53.23.Terris-Prestholt F, Hanson K, MacPhail C, et al. How much demand for New HIV prevention technologies can we really expect? Results from a discrete choice experiment in South Africa. How much demand for New HIV prevention technologies can we really expect? Results from a discrete choice experiment in South Africa 2013;8:e83193.24.Ostermann J, Njau B, Brown DS, et al. Heterogeneous HIV Testing Preferences in an Urban Setting in Tanzania: Results from a Discrete Choice Experiment. Heterogeneous HIV Testing Preferences in an Urban Setting in Tanzania: Results from a Discrete Choice Experiment 2014;9:e92100.25.Phillips KA, Maddala T, and Johnson FR. Measuring preferences for health care interventions using conjoint analysis: an application to HIV testing. Measuring preferences for health care interventions using conjoint analysis: an application to HIV testing 2002;37:1681-1705.26.Ryan M. Discrete choice experiments in health care. BMJ 2004;328:360-361.27.Terris-Prestholt F, Quaiffe M, and Vickerman P. Parameterising user uptake in economic evaluations: the role of discrete choice experiments. Parameterising user uptake in economic evaluations: the role of discrete choice experiments In press.28.Philip P, Hickson F, Bonell C, et al. Men who have sex with men in Britain: comparison of estimates from a probability sample and community-based surveys. Men who have sex with men in Britain: comparison of estimates from a probability sample and community-based surveys In submission.29.Orme B. Sample size issues for conjoint analysis, in Getting started with conjoint analysis: strategies for product design and pricing research. 2010, Research Publishers LLC: Madison, Wisconson.)**[HIV testing history and preferences for future tests among gay men, bisexual men and other MSM in England](1.Public Health England. HIV in the United Kingdom: 2014 report. 2014.2.Public Health England. HIV new diagnosis, treatment and care: 2015 report.3.Williamson LM, Dodds JP, Mercey DE, et al. Sexual risk behaviour and knowledge of HIV status among community samples of gay men in the UK. Sexual risk behaviour and knowledge of HIV status among community samples of gay men in the UK 2008;22:1063-70.4.Holt M, Rawstorne P, Wilkinson J, et al. HIV testing, gay community involvement and internet use: social and behavioural correlates of HIV testing among Australian men who have sex with men. HIV testing, gay community involvement and internet use: social and behavioural correlates of HIV testing among Australian men who have sex with men 2012;16:13-22.5.Zablotska I, Holt M, de Wit J, et al. Gay men who are not getting tested for HIV. Gay men who are not getting tested for HIV 2012;16:1887-94.6.Witzel TC, Melendez-Torres GJ, Hickson F, et al. HIV testing history and preferences for future tests among gay men, bisexual men and other MSM in England. HIV testing history and preferences for future tests among gay men, bisexual men and other MSM in England In submission.7.UNSW Australia. 2015. Annual report of trends in behaviour 2015. HIV/AIDS, hepatitis and sexually transmissible infections in Australia [Online]. Available: https://csrh.arts.unsw.edu.au/media/CSRHFile/CSRH_Annual_Report_of_Trends_in_Behaviour_2015.pdf.8.Phillips AN, Cambiano V, Miners A, et al. Potential impact on HIV incidence of higher HIV testing rates and earlier antiretroviral therapy initiation in MSM. Potential impact on HIV incidence of higher HIV testing rates and earlier antiretroviral therapy initiation in MSM 2015;29:1855-62.9.Nakagawa F, Lodwick RK, Smith CJ, et al. Projected life expectancy of people with HIV according to timing of diagnosis. Projected life expectancy of people with HIV according to timing of diagnosis 2012;26:335-43.10.Chadborn TR, Delpech VC, Sabin CA, et al. The late diagnosis and consequent short-term mortality of HIV-infected heterosexuals (England and Wales, 2000–2004). The late diagnosis and consequent short-term mortality of HIV-infected heterosexuals (England and Wales, 2000–2004) 2006;20:2371-2379.11.Miners A, Phillips A, Kreif N, et al. Health-related quality-of-life of people with HIV in the era of combination antiretroviral treatment: a cross-sectional comparison with the general population. Health-related quality-of-life of people with HIV in the era of combination antiretroviral treatment: a cross-sectional comparison with the general population 2014;1:e32–e40.12.Cohen MS, Chen YQ, McCauley M, et al. Prevention of HIV-1 Infection with Early Antiretroviral Therapy. Prevention of HIV-1 Infection with Early Antiretroviral Therapy 2011;365:493-505.13.Marks G, Crepaz N, and Janssen RS. Estimating sexual transmission of HIV from persons aware and unaware that they are infected with the virus in the USA. Estimating sexual transmission of HIV from persons aware and unaware that they are infected with the virus in the USA 2006;20:1447-50.14.Nakagawa F, Miners A, Smith CJ, et al. Projected Lifetime Healthcare Costs Associated with HIV Infection. Projected Lifetime Healthcare Costs Associated with HIV Infection 2015;10:e0125018.15.Fleishman JA, Yehia BR, Moore RD, et al. The Economic Burden of Late Entry Into Medical Care for Patients With HIV Infection. The Economic Burden of Late Entry Into Medical Care for Patients With HIV Infection 2010;48:1071-1079.16.Krentz HB and Gill MJ. The Direct Medical Costs of Late Presentation (<350/mm(3)) of HIV Infection over a 15-Year Period. The Direct Medical Costs of Late Presentation (<350/mm(3)) of HIV Infection over a 15-Year Period 2012;2012:757135.17.Krause J, Subklew-Sehume F, Kenyon C, et al. Acceptability of HIV self-testing: a systematic literature review. Acceptability of HIV self-testing: a systematic literature review 2013;13:735.18.Frye V, Wilton L, Hirshfied S, et al. \"Just Because It's Out There, People Aren't Going to Use It.\" HIV Self-Testing Among Young, Black MSM, and Transgender Women. \"Just Because It's Out There, People Aren't Going to Use It.\" HIV Self-Testing Among Young, Black MSM, and Transgender Women 2015.19.Figueroa C, Johnson C, Verster A, et al. Attitudes and Acceptability on HIV Self-testing Among Key Populations: A Literature Review. Attitudes and Acceptability on HIV Self-testing Among Key Populations: A Literature Review 2015.20.Bavinton BR, Brown G, Hurley M, et al. Which gay men would increase their frequency of HIV testing with home self-testing? Which gay men would increase their frequency of HIV testing with home self-testing? 2013;17:2084-92.21.Hensen B, Lewis JJ, Schaap A, et al. Factors associated with HIV-testing and acceptance of an offer of home-based testing by men in rural Zambia. Factors associated with HIV-testing and acceptance of an offer of home-based testing by men in rural Zambia 2015;19:492-504.22.Lee VJ, Tan SC, Earnest A, et al. User acceptability and feasibility of self-testing with HIV rapid tests. User acceptability and feasibility of self-testing with HIV rapid tests 2007;45:449-53.23.Terris-Prestholt F, Hanson K, MacPhail C, et al. How much demand for New HIV prevention technologies can we really expect? Results from a discrete choice experiment in South Africa. How much demand for New HIV prevention technologies can we really expect? Results from a discrete choice experiment in South Africa 2013;8:e83193.24.Ostermann J, Njau B, Brown DS, et al. Heterogeneous HIV Testing Preferences in an Urban Setting in Tanzania: Results from a Discrete Choice Experiment. Heterogeneous HIV Testing Preferences in an Urban Setting in Tanzania: Results from a Discrete Choice Experiment 2014;9:e92100.25.Phillips KA, Maddala T, and Johnson FR. Measuring preferences for health care interventions using conjoint analysis: an application to HIV testing. Measuring preferences for health care interventions using conjoint analysis: an application to HIV testing 2002;37:1681-1705.26.Ryan M. Discrete choice experiments in health care. BMJ 2004;328:360-361.27.Terris-Prestholt F, Quaiffe M, and Vickerman P. Parameterising user uptake in economic evaluations: the role of discrete choice experiments. Parameterising user uptake in economic evaluations: the role of discrete choice experiments In press.28.Philip P, Hickson F, Bonell C, et al. Men who have sex with men in Britain: comparison of estimates from a probability sample and community-based surveys. Men who have sex with men in Britain: comparison of estimates from a probability sample and community-based surveys In submission.29.Orme B. Sample size issues for conjoint analysis, in Getting started with conjoint analysis: strategies for product design and pricing research. 2010, Research Publishers LLC: Madison, Wisconson.)* [In submission.](1.Public Health England. HIV in the United Kingdom: 2014 report. 2014.2.Public Health England. HIV new diagnosis, treatment and care: 2015 report.3.Williamson LM, Dodds JP, Mercey DE, et al. Sexual risk behaviour and knowledge of HIV status among community samples of gay men in the UK. Sexual risk behaviour and knowledge of HIV status among community samples of gay men in the UK 2008;22:1063-70.4.Holt M, Rawstorne P, Wilkinson J, et al. HIV testing, gay community involvement and internet use: social and behavioural correlates of HIV testing among Australian men who have sex with men. HIV testing, gay community involvement and internet use: social and behavioural correlates of HIV testing among Australian men who have sex with men 2012;16:13-22.5.Zablotska I, Holt M, de Wit J, et al. Gay men who are not getting tested for HIV. Gay men who are not getting tested for HIV 2012;16:1887-94.6.Witzel TC, Melendez-Torres GJ, Hickson F, et al. HIV testing history and preferences for future tests among gay men, bisexual men and other MSM in England. HIV testing history and preferences for future tests among gay men, bisexual men and other MSM in England In submission.7.UNSW Australia. 2015. Annual report of trends in behaviour 2015. HIV/AIDS, hepatitis and sexually transmissible infections in Australia [Online]. Available: https://csrh.arts.unsw.edu.au/media/CSRHFile/CSRH_Annual_Report_of_Trends_in_Behaviour_2015.pdf.8.Phillips AN, Cambiano V, Miners A, et al. Potential impact on HIV incidence of higher HIV testing rates and earlier antiretroviral therapy initiation in MSM. Potential impact on HIV incidence of higher HIV testing rates and earlier antiretroviral therapy initiation in MSM 2015;29:1855-62.9.Nakagawa F, Lodwick RK, Smith CJ, et al. Projected life expectancy of people with HIV according to timing of diagnosis. Projected life expectancy of people with HIV according to timing of diagnosis 2012;26:335-43.10.Chadborn TR, Delpech VC, Sabin CA, et al. The late diagnosis and consequent short-term mortality of HIV-infected heterosexuals (England and Wales, 2000–2004). The late diagnosis and consequent short-term mortality of HIV-infected heterosexuals (England and Wales, 2000–2004) 2006;20:2371-2379.11.Miners A, Phillips A, Kreif N, et al. Health-related quality-of-life of people with HIV in the era of combination antiretroviral treatment: a cross-sectional comparison with the general population. Health-related quality-of-life of people with HIV in the era of combination antiretroviral treatment: a cross-sectional comparison with the general population 2014;1:e32–e40.12.Cohen MS, Chen YQ, McCauley M, et al. Prevention of HIV-1 Infection with Early Antiretroviral Therapy. Prevention of HIV-1 Infection with Early Antiretroviral Therapy 2011;365:493-505.13.Marks G, Crepaz N, and Janssen RS. Estimating sexual transmission of HIV from persons aware and unaware that they are infected with the virus in the USA. Estimating sexual transmission of HIV from persons aware and unaware that they are infected with the virus in the USA 2006;20:1447-50.14.Nakagawa F, Miners A, Smith CJ, et al. Projected Lifetime Healthcare Costs Associated with HIV Infection. Projected Lifetime Healthcare Costs Associated with HIV Infection 2015;10:e0125018.15.Fleishman JA, Yehia BR, Moore RD, et al. The Economic Burden of Late Entry Into Medical Care for Patients With HIV Infection. The Economic Burden of Late Entry Into Medical Care for Patients With HIV Infection 2010;48:1071-1079.16.Krentz HB and Gill MJ. The Direct Medical Costs of Late Presentation (<350/mm(3)) of HIV Infection over a 15-Year Period. The Direct Medical Costs of Late Presentation (<350/mm(3)) of HIV Infection over a 15-Year Period 2012;2012:757135.17.Krause J, Subklew-Sehume F, Kenyon C, et al. Acceptability of HIV self-testing: a systematic literature review. Acceptability of HIV self-testing: a systematic literature review 2013;13:735.18.Frye V, Wilton L, Hirshfied S, et al. \"Just Because It's Out There, People Aren't Going to Use It.\" HIV Self-Testing Among Young, Black MSM, and Transgender Women. \"Just Because It's Out There, People Aren't Going to Use It.\" HIV Self-Testing Among Young, Black MSM, and Transgender Women 2015.19.Figueroa C, Johnson C, Verster A, et al. Attitudes and Acceptability on HIV Self-testing Among Key Populations: A Literature Review. Attitudes and Acceptability on HIV Self-testing Among Key Populations: A Literature Review 2015.20.Bavinton BR, Brown G, Hurley M, et al. Which gay men would increase their frequency of HIV testing with home self-testing? Which gay men would increase their frequency of HIV testing with home self-testing? 2013;17:2084-92.21.Hensen B, Lewis JJ, Schaap A, et al. Factors associated with HIV-testing and acceptance of an offer of home-based testing by men in rural Zambia. Factors associated with HIV-testing and acceptance of an offer of home-based testing by men in rural Zambia 2015;19:492-504.22.Lee VJ, Tan SC, Earnest A, et al. User acceptability and feasibility of self-testing with HIV rapid tests. User acceptability and feasibility of self-testing with HIV rapid tests 2007;45:449-53.23.Terris-Prestholt F, Hanson K, MacPhail C, et al. How much demand for New HIV prevention technologies can we really expect? Results from a discrete choice experiment in South Africa. How much demand for New HIV prevention technologies can we really expect? Results from a discrete choice experiment in South Africa 2013;8:e83193.24.Ostermann J, Njau B, Brown DS, et al. Heterogeneous HIV Testing Preferences in an Urban Setting in Tanzania: Results from a Discrete Choice Experiment. Heterogeneous HIV Testing Preferences in an Urban Setting in Tanzania: Results from a Discrete Choice Experiment 2014;9:e92100.25.Phillips KA, Maddala T, and Johnson FR. Measuring preferences for health care interventions using conjoint analysis: an application to HIV testing. Measuring preferences for health care interventions using conjoint analysis: an application to HIV testing 2002;37:1681-1705.26.Ryan M. Discrete choice experiments in health care. BMJ 2004;328:360-361.27.Terris-Prestholt F, Quaiffe M, and Vickerman P. Parameterising user uptake in economic evaluations: the role of discrete choice experiments. Parameterising user uptake in economic evaluations: the role of discrete choice experiments In press.28.Philip P, Hickson F, Bonell C, et al. Men who have sex with men in Britain: comparison of estimates from a probability sample and community-based surveys. Men who have sex with men in Britain: comparison of estimates from a probability sample and community-based surveys In submission.29.Orme B. Sample size issues for conjoint analysis, in Getting started with conjoint analysis: strategies for product design and pricing research. 2010, Research Publishers LLC: Madison, Wisconson.)

[7. UNSW Australia. 2015.](1.Public Health England. HIV in the United Kingdom: 2014 report. 2014.2.Public Health England. HIV new diagnosis, treatment and care: 2015 report.3.Williamson LM, Dodds JP, Mercey DE, et al. Sexual risk behaviour and knowledge of HIV status among community samples of gay men in the UK. Sexual risk behaviour and knowledge of HIV status among community samples of gay men in the UK 2008;22:1063-70.4.Holt M, Rawstorne P, Wilkinson J, et al. HIV testing, gay community involvement and internet use: social and behavioural correlates of HIV testing among Australian men who have sex with men. HIV testing, gay community involvement and internet use: social and behavioural correlates of HIV testing among Australian men who have sex with men 2012;16:13-22.5.Zablotska I, Holt M, de Wit J, et al. Gay men who are not getting tested for HIV. Gay men who are not getting tested for HIV 2012;16:1887-94.6.Witzel TC, Melendez-Torres GJ, Hickson F, et al. HIV testing history and preferences for future tests among gay men, bisexual men and other MSM in England. HIV testing history and preferences for future tests among gay men, bisexual men and other MSM in England In submission.7.UNSW Australia. 2015. Annual report of trends in behaviour 2015. HIV/AIDS, hepatitis and sexually transmissible infections in Australia [Online]. Available: https://csrh.arts.unsw.edu.au/media/CSRHFile/CSRH_Annual_Report_of_Trends_in_Behaviour_2015.pdf.8.Phillips AN, Cambiano V, Miners A, et al. Potential impact on HIV incidence of higher HIV testing rates and earlier antiretroviral therapy initiation in MSM. Potential impact on HIV incidence of higher HIV testing rates and earlier antiretroviral therapy initiation in MSM 2015;29:1855-62.9.Nakagawa F, Lodwick RK, Smith CJ, et al. Projected life expectancy of people with HIV according to timing of diagnosis. Projected life expectancy of people with HIV according to timing of diagnosis 2012;26:335-43.10.Chadborn TR, Delpech VC, Sabin CA, et al. The late diagnosis and consequent short-term mortality of HIV-infected heterosexuals (England and Wales, 2000–2004). The late diagnosis and consequent short-term mortality of HIV-infected heterosexuals (England and Wales, 2000–2004) 2006;20:2371-2379.11.Miners A, Phillips A, Kreif N, et al. Health-related quality-of-life of people with HIV in the era of combination antiretroviral treatment: a cross-sectional comparison with the general population. Health-related quality-of-life of people with HIV in the era of combination antiretroviral treatment: a cross-sectional comparison with the general population 2014;1:e32–e40.12.Cohen MS, Chen YQ, McCauley M, et al. Prevention of HIV-1 Infection with Early Antiretroviral Therapy. Prevention of HIV-1 Infection with Early Antiretroviral Therapy 2011;365:493-505.13.Marks G, Crepaz N, and Janssen RS. Estimating sexual transmission of HIV from persons aware and unaware that they are infected with the virus in the USA. Estimating sexual transmission of HIV from persons aware and unaware that they are infected with the virus in the USA 2006;20:1447-50.14.Nakagawa F, Miners A, Smith CJ, et al. Projected Lifetime Healthcare Costs Associated with HIV Infection. Projected Lifetime Healthcare Costs Associated with HIV Infection 2015;10:e0125018.15.Fleishman JA, Yehia BR, Moore RD, et al. The Economic Burden of Late Entry Into Medical Care for Patients With HIV Infection. The Economic Burden of Late Entry Into Medical Care for Patients With HIV Infection 2010;48:1071-1079.16.Krentz HB and Gill MJ. The Direct Medical Costs of Late Presentation (<350/mm(3)) of HIV Infection over a 15-Year Period. The Direct Medical Costs of Late Presentation (<350/mm(3)) of HIV Infection over a 15-Year Period 2012;2012:757135.17.Krause J, Subklew-Sehume F, Kenyon C, et al. Acceptability of HIV self-testing: a systematic literature review. Acceptability of HIV self-testing: a systematic literature review 2013;13:735.18.Frye V, Wilton L, Hirshfied S, et al. \"Just Because It's Out There, People Aren't Going to Use It.\" HIV Self-Testing Among Young, Black MSM, and Transgender Women. \"Just Because It's Out There, People Aren't Going to Use It.\" HIV Self-Testing Among Young, Black MSM, and Transgender Women 2015.19.Figueroa C, Johnson C, Verster A, et al. Attitudes and Acceptability on HIV Self-testing Among Key Populations: A Literature Review. Attitudes and Acceptability on HIV Self-testing Among Key Populations: A Literature Review 2015.20.Bavinton BR, Brown G, Hurley M, et al. Which gay men would increase their frequency of HIV testing with home self-testing? Which gay men would increase their frequency of HIV testing with home self-testing? 2013;17:2084-92.21.Hensen B, Lewis JJ, Schaap A, et al. Factors associated with HIV-testing and acceptance of an offer of home-based testing by men in rural Zambia. Factors associated with HIV-testing and acceptance of an offer of home-based testing by men in rural Zambia 2015;19:492-504.22.Lee VJ, Tan SC, Earnest A, et al. User acceptability and feasibility of self-testing with HIV rapid tests. User acceptability and feasibility of self-testing with HIV rapid tests 2007;45:449-53.23.Terris-Prestholt F, Hanson K, MacPhail C, et al. How much demand for New HIV prevention technologies can we really expect? Results from a discrete choice experiment in South Africa. How much demand for New HIV prevention technologies can we really expect? Results from a discrete choice experiment in South Africa 2013;8:e83193.24.Ostermann J, Njau B, Brown DS, et al. Heterogeneous HIV Testing Preferences in an Urban Setting in Tanzania: Results from a Discrete Choice Experiment. Heterogeneous HIV Testing Preferences in an Urban Setting in Tanzania: Results from a Discrete Choice Experiment 2014;9:e92100.25.Phillips KA, Maddala T, and Johnson FR. Measuring preferences for health care interventions using conjoint analysis: an application to HIV testing. Measuring preferences for health care interventions using conjoint analysis: an application to HIV testing 2002;37:1681-1705.26.Ryan M. Discrete choice experiments in health care. BMJ 2004;328:360-361.27.Terris-Prestholt F, Quaiffe M, and Vickerman P. Parameterising user uptake in economic evaluations: the role of discrete choice experiments. Parameterising user uptake in economic evaluations: the role of discrete choice experiments In press.28.Philip P, Hickson F, Bonell C, et al. Men who have sex with men in Britain: comparison of estimates from a probability sample and community-based surveys. Men who have sex with men in Britain: comparison of estimates from a probability sample and community-based surveys In submission.29.Orme B. Sample size issues for conjoint analysis, in Getting started with conjoint analysis: strategies for product design and pricing research. 2010, Research Publishers LLC: Madison, Wisconson.) *[Annual report of trends in behaviour 2015. HIV/AIDS, hepatitis and sexually transmissible infections in Australia](1.Public Health England. HIV in the United Kingdom: 2014 report. 2014.2.Public Health England. HIV new diagnosis, treatment and care: 2015 report.3.Williamson LM, Dodds JP, Mercey DE, et al. Sexual risk behaviour and knowledge of HIV status among community samples of gay men in the UK. Sexual risk behaviour and knowledge of HIV status among community samples of gay men in the UK 2008;22:1063-70.4.Holt M, Rawstorne P, Wilkinson J, et al. HIV testing, gay community involvement and internet use: social and behavioural correlates of HIV testing among Australian men who have sex with men. HIV testing, gay community involvement and internet use: social and behavioural correlates of HIV testing among Australian men who have sex with men 2012;16:13-22.5.Zablotska I, Holt M, de Wit J, et al. Gay men who are not getting tested for HIV. Gay men who are not getting tested for HIV 2012;16:1887-94.6.Witzel TC, Melendez-Torres GJ, Hickson F, et al. HIV testing history and preferences for future tests among gay men, bisexual men and other MSM in England. HIV testing history and preferences for future tests among gay men, bisexual men and other MSM in England In submission.7.UNSW Australia. 2015. Annual report of trends in behaviour 2015. HIV/AIDS, hepatitis and sexually transmissible infections in Australia [Online]. Available: https://csrh.arts.unsw.edu.au/media/CSRHFile/CSRH_Annual_Report_of_Trends_in_Behaviour_2015.pdf.8.Phillips AN, Cambiano V, Miners A, et al. Potential impact on HIV incidence of higher HIV testing rates and earlier antiretroviral therapy initiation in MSM. Potential impact on HIV incidence of higher HIV testing rates and earlier antiretroviral therapy initiation in MSM 2015;29:1855-62.9.Nakagawa F, Lodwick RK, Smith CJ, et al. Projected life expectancy of people with HIV according to timing of diagnosis. Projected life expectancy of people with HIV according to timing of diagnosis 2012;26:335-43.10.Chadborn TR, Delpech VC, Sabin CA, et al. The late diagnosis and consequent short-term mortality of HIV-infected heterosexuals (England and Wales, 2000–2004). The late diagnosis and consequent short-term mortality of HIV-infected heterosexuals (England and Wales, 2000–2004) 2006;20:2371-2379.11.Miners A, Phillips A, Kreif N, et al. Health-related quality-of-life of people with HIV in the era of combination antiretroviral treatment: a cross-sectional comparison with the general population. Health-related quality-of-life of people with HIV in the era of combination antiretroviral treatment: a cross-sectional comparison with the general population 2014;1:e32–e40.12.Cohen MS, Chen YQ, McCauley M, et al. Prevention of HIV-1 Infection with Early Antiretroviral Therapy. Prevention of HIV-1 Infection with Early Antiretroviral Therapy 2011;365:493-505.13.Marks G, Crepaz N, and Janssen RS. Estimating sexual transmission of HIV from persons aware and unaware that they are infected with the virus in the USA. Estimating sexual transmission of HIV from persons aware and unaware that they are infected with the virus in the USA 2006;20:1447-50.14.Nakagawa F, Miners A, Smith CJ, et al. Projected Lifetime Healthcare Costs Associated with HIV Infection. Projected Lifetime Healthcare Costs Associated with HIV Infection 2015;10:e0125018.15.Fleishman JA, Yehia BR, Moore RD, et al. The Economic Burden of Late Entry Into Medical Care for Patients With HIV Infection. The Economic Burden of Late Entry Into Medical Care for Patients With HIV Infection 2010;48:1071-1079.16.Krentz HB and Gill MJ. The Direct Medical Costs of Late Presentation (<350/mm(3)) of HIV Infection over a 15-Year Period. The Direct Medical Costs of Late Presentation (<350/mm(3)) of HIV Infection over a 15-Year Period 2012;2012:757135.17.Krause J, Subklew-Sehume F, Kenyon C, et al. Acceptability of HIV self-testing: a systematic literature review. Acceptability of HIV self-testing: a systematic literature review 2013;13:735.18.Frye V, Wilton L, Hirshfied S, et al. \"Just Because It's Out There, People Aren't Going to Use It.\" HIV Self-Testing Among Young, Black MSM, and Transgender Women. \"Just Because It's Out There, People Aren't Going to Use It.\" HIV Self-Testing Among Young, Black MSM, and Transgender Women 2015.19.Figueroa C, Johnson C, Verster A, et al. Attitudes and Acceptability on HIV Self-testing Among Key Populations: A Literature Review. Attitudes and Acceptability on HIV Self-testing Among Key Populations: A Literature Review 2015.20.Bavinton BR, Brown G, Hurley M, et al. Which gay men would increase their frequency of HIV testing with home self-testing? Which gay men would increase their frequency of HIV testing with home self-testing? 2013;17:2084-92.21.Hensen B, Lewis JJ, Schaap A, et al. Factors associated with HIV-testing and acceptance of an offer of home-based testing by men in rural Zambia. Factors associated with HIV-testing and acceptance of an offer of home-based testing by men in rural Zambia 2015;19:492-504.22.Lee VJ, Tan SC, Earnest A, et al. User acceptability and feasibility of self-testing with HIV rapid tests. User acceptability and feasibility of self-testing with HIV rapid tests 2007;45:449-53.23.Terris-Prestholt F, Hanson K, MacPhail C, et al. How much demand for New HIV prevention technologies can we really expect? Results from a discrete choice experiment in South Africa. How much demand for New HIV prevention technologies can we really expect? Results from a discrete choice experiment in South Africa 2013;8:e83193.24.Ostermann J, Njau B, Brown DS, et al. Heterogeneous HIV Testing Preferences in an Urban Setting in Tanzania: Results from a Discrete Choice Experiment. Heterogeneous HIV Testing Preferences in an Urban Setting in Tanzania: Results from a Discrete Choice Experiment 2014;9:e92100.25.Phillips KA, Maddala T, and Johnson FR. Measuring preferences for health care interventions using conjoint analysis: an application to HIV testing. Measuring preferences for health care interventions using conjoint analysis: an application to HIV testing 2002;37:1681-1705.26.Ryan M. Discrete choice experiments in health care. BMJ 2004;328:360-361.27.Terris-Prestholt F, Quaiffe M, and Vickerman P. Parameterising user uptake in economic evaluations: the role of discrete choice experiments. Parameterising user uptake in economic evaluations: the role of discrete choice experiments In press.28.Philip P, Hickson F, Bonell C, et al. Men who have sex with men in Britain: comparison of estimates from a probability sample and community-based surveys. Men who have sex with men in Britain: comparison of estimates from a probability sample and community-based surveys In submission.29.Orme B. Sample size issues for conjoint analysis, in Getting started with conjoint analysis: strategies for product design and pricing research. 2010, Research Publishers LLC: Madison, Wisconson.)* [[Online]. Available: https://csrh.arts.unsw.edu.au/media/CSRHFile/CSRH_Annual_Report_of_Trends_in_Behaviour_2015.pdf.](1.Public Health England. HIV in the United Kingdom: 2014 report. 2014.2.Public Health England. HIV new diagnosis, treatment and care: 2015 report.3.Williamson LM, Dodds JP, Mercey DE, et al. Sexual risk behaviour and knowledge of HIV status among community samples of gay men in the UK. Sexual risk behaviour and knowledge of HIV status among community samples of gay men in the UK 2008;22:1063-70.4.Holt M, Rawstorne P, Wilkinson J, et al. HIV testing, gay community involvement and internet use: social and behavioural correlates of HIV testing among Australian men who have sex with men. HIV testing, gay community involvement and internet use: social and behavioural correlates of HIV testing among Australian men who have sex with men 2012;16:13-22.5.Zablotska I, Holt M, de Wit J, et al. Gay men who are not getting tested for HIV. Gay men who are not getting tested for HIV 2012;16:1887-94.6.Witzel TC, Melendez-Torres GJ, Hickson F, et al. HIV testing history and preferences for future tests among gay men, bisexual men and other MSM in England. HIV testing history and preferences for future tests among gay men, bisexual men and other MSM in England In submission.7.UNSW Australia. 2015. Annual report of trends in behaviour 2015. HIV/AIDS, hepatitis and sexually transmissible infections in Australia [Online]. Available: https://csrh.arts.unsw.edu.au/media/CSRHFile/CSRH_Annual_Report_of_Trends_in_Behaviour_2015.pdf.8.Phillips AN, Cambiano V, Miners A, et al. Potential impact on HIV incidence of higher HIV testing rates and earlier antiretroviral therapy initiation in MSM. Potential impact on HIV incidence of higher HIV testing rates and earlier antiretroviral therapy initiation in MSM 2015;29:1855-62.9.Nakagawa F, Lodwick RK, Smith CJ, et al. Projected life expectancy of people with HIV according to timing of diagnosis. Projected life expectancy of people with HIV according to timing of diagnosis 2012;26:335-43.10.Chadborn TR, Delpech VC, Sabin CA, et al. The late diagnosis and consequent short-term mortality of HIV-infected heterosexuals (England and Wales, 2000–2004). The late diagnosis and consequent short-term mortality of HIV-infected heterosexuals (England and Wales, 2000–2004) 2006;20:2371-2379.11.Miners A, Phillips A, Kreif N, et al. Health-related quality-of-life of people with HIV in the era of combination antiretroviral treatment: a cross-sectional comparison with the general population. Health-related quality-of-life of people with HIV in the era of combination antiretroviral treatment: a cross-sectional comparison with the general population 2014;1:e32–e40.12.Cohen MS, Chen YQ, McCauley M, et al. Prevention of HIV-1 Infection with Early Antiretroviral Therapy. Prevention of HIV-1 Infection with Early Antiretroviral Therapy 2011;365:493-505.13.Marks G, Crepaz N, and Janssen RS. Estimating sexual transmission of HIV from persons aware and unaware that they are infected with the virus in the USA. Estimating sexual transmission of HIV from persons aware and unaware that they are infected with the virus in the USA 2006;20:1447-50.14.Nakagawa F, Miners A, Smith CJ, et al. Projected Lifetime Healthcare Costs Associated with HIV Infection. Projected Lifetime Healthcare Costs Associated with HIV Infection 2015;10:e0125018.15.Fleishman JA, Yehia BR, Moore RD, et al. The Economic Burden of Late Entry Into Medical Care for Patients With HIV Infection. The Economic Burden of Late Entry Into Medical Care for Patients With HIV Infection 2010;48:1071-1079.16.Krentz HB and Gill MJ. The Direct Medical Costs of Late Presentation (<350/mm(3)) of HIV Infection over a 15-Year Period. The Direct Medical Costs of Late Presentation (<350/mm(3)) of HIV Infection over a 15-Year Period 2012;2012:757135.17.Krause J, Subklew-Sehume F, Kenyon C, et al. Acceptability of HIV self-testing: a systematic literature review. Acceptability of HIV self-testing: a systematic literature review 2013;13:735.18.Frye V, Wilton L, Hirshfied S, et al. \"Just Because It's Out There, People Aren't Going to Use It.\" HIV Self-Testing Among Young, Black MSM, and Transgender Women. \"Just Because It's Out There, People Aren't Going to Use It.\" HIV Self-Testing Among Young, Black MSM, and Transgender Women 2015.19.Figueroa C, Johnson C, Verster A, et al. Attitudes and Acceptability on HIV Self-testing Among Key Populations: A Literature Review. Attitudes and Acceptability on HIV Self-testing Among Key Populations: A Literature Review 2015.20.Bavinton BR, Brown G, Hurley M, et al. Which gay men would increase their frequency of HIV testing with home self-testing? Which gay men would increase their frequency of HIV testing with home self-testing? 2013;17:2084-92.21.Hensen B, Lewis JJ, Schaap A, et al. Factors associated with HIV-testing and acceptance of an offer of home-based testing by men in rural Zambia. Factors associated with HIV-testing and acceptance of an offer of home-based testing by men in rural Zambia 2015;19:492-504.22.Lee VJ, Tan SC, Earnest A, et al. User acceptability and feasibility of self-testing with HIV rapid tests. User acceptability and feasibility of self-testing with HIV rapid tests 2007;45:449-53.23.Terris-Prestholt F, Hanson K, MacPhail C, et al. How much demand for New HIV prevention technologies can we really expect? Results from a discrete choice experiment in South Africa. How much demand for New HIV prevention technologies can we really expect? Results from a discrete choice experiment in South Africa 2013;8:e83193.24.Ostermann J, Njau B, Brown DS, et al. Heterogeneous HIV Testing Preferences in an Urban Setting in Tanzania: Results from a Discrete Choice Experiment. Heterogeneous HIV Testing Preferences in an Urban Setting in Tanzania: Results from a Discrete Choice Experiment 2014;9:e92100.25.Phillips KA, Maddala T, and Johnson FR. Measuring preferences for health care interventions using conjoint analysis: an application to HIV testing. Measuring preferences for health care interventions using conjoint analysis: an application to HIV testing 2002;37:1681-1705.26.Ryan M. Discrete choice experiments in health care. BMJ 2004;328:360-361.27.Terris-Prestholt F, Quaiffe M, and Vickerman P. Parameterising user uptake in economic evaluations: the role of discrete choice experiments. Parameterising user uptake in economic evaluations: the role of discrete choice experiments In press.28.Philip P, Hickson F, Bonell C, et al. Men who have sex with men in Britain: comparison of estimates from a probability sample and community-based surveys. Men who have sex with men in Britain: comparison of estimates from a probability sample and community-based surveys In submission.29.Orme B. Sample size issues for conjoint analysis, in Getting started with conjoint analysis: strategies for product design and pricing research. 2010, Research Publishers LLC: Madison, Wisconson.)

[8. Phillips AN, Cambiano V, Miners A, et al. Potential impact on HIV incidence of higher HIV testing rates and earlier antiretroviral therapy initiation in MSM](1.Public Health England. HIV in the United Kingdom: 2014 report. 2014.2.Public Health England. HIV new diagnosis, treatment and care: 2015 report.3.Williamson LM, Dodds JP, Mercey DE, et al. Sexual risk behaviour and knowledge of HIV status among community samples of gay men in the UK. Sexual risk behaviour and knowledge of HIV status among community samples of gay men in the UK 2008;22:1063-70.4.Holt M, Rawstorne P, Wilkinson J, et al. HIV testing, gay community involvement and internet use: social and behavioural correlates of HIV testing among Australian men who have sex with men. HIV testing, gay community involvement and internet use: social and behavioural correlates of HIV testing among Australian men who have sex with men 2012;16:13-22.5.Zablotska I, Holt M, de Wit J, et al. Gay men who are not getting tested for HIV. Gay men who are not getting tested for HIV 2012;16:1887-94.6.Witzel TC, Melendez-Torres GJ, Hickson F, et al. HIV testing history and preferences for future tests among gay men, bisexual men and other MSM in England. HIV testing history and preferences for future tests among gay men, bisexual men and other MSM in England In submission.7.UNSW Australia. 2015. Annual report of trends in behaviour 2015. HIV/AIDS, hepatitis and sexually transmissible infections in Australia [Online]. Available: https://csrh.arts.unsw.edu.au/media/CSRHFile/CSRH_Annual_Report_of_Trends_in_Behaviour_2015.pdf.8.Phillips AN, Cambiano V, Miners A, et al. Potential impact on HIV incidence of higher HIV testing rates and earlier antiretroviral therapy initiation in MSM. Potential impact on HIV incidence of higher HIV testing rates and earlier antiretroviral therapy initiation in MSM 2015;29:1855-62.9.Nakagawa F, Lodwick RK, Smith CJ, et al. Projected life expectancy of people with HIV according to timing of diagnosis. Projected life expectancy of people with HIV according to timing of diagnosis 2012;26:335-43.10.Chadborn TR, Delpech VC, Sabin CA, et al. The late diagnosis and consequent short-term mortality of HIV-infected heterosexuals (England and Wales, 2000–2004). The late diagnosis and consequent short-term mortality of HIV-infected heterosexuals (England and Wales, 2000–2004) 2006;20:2371-2379.11.Miners A, Phillips A, Kreif N, et al. Health-related quality-of-life of people with HIV in the era of combination antiretroviral treatment: a cross-sectional comparison with the general population. Health-related quality-of-life of people with HIV in the era of combination antiretroviral treatment: a cross-sectional comparison with the general population 2014;1:e32–e40.12.Cohen MS, Chen YQ, McCauley M, et al. Prevention of HIV-1 Infection with Early Antiretroviral Therapy. Prevention of HIV-1 Infection with Early Antiretroviral Therapy 2011;365:493-505.13.Marks G, Crepaz N, and Janssen RS. Estimating sexual transmission of HIV from persons aware and unaware that they are infected with the virus in the USA. Estimating sexual transmission of HIV from persons aware and unaware that they are infected with the virus in the USA 2006;20:1447-50.14.Nakagawa F, Miners A, Smith CJ, et al. Projected Lifetime Healthcare Costs Associated with HIV Infection. Projected Lifetime Healthcare Costs Associated with HIV Infection 2015;10:e0125018.15.Fleishman JA, Yehia BR, Moore RD, et al. The Economic Burden of Late Entry Into Medical Care for Patients With HIV Infection. The Economic Burden of Late Entry Into Medical Care for Patients With HIV Infection 2010;48:1071-1079.16.Krentz HB and Gill MJ. The Direct Medical Costs of Late Presentation (<350/mm(3)) of HIV Infection over a 15-Year Period. The Direct Medical Costs of Late Presentation (<350/mm(3)) of HIV Infection over a 15-Year Period 2012;2012:757135.17.Krause J, Subklew-Sehume F, Kenyon C, et al. Acceptability of HIV self-testing: a systematic literature review. Acceptability of HIV self-testing: a systematic literature review 2013;13:735.18.Frye V, Wilton L, Hirshfied S, et al. \"Just Because It's Out There, People Aren't Going to Use It.\" HIV Self-Testing Among Young, Black MSM, and Transgender Women. \"Just Because It's Out There, People Aren't Going to Use It.\" HIV Self-Testing Among Young, Black MSM, and Transgender Women 2015.19.Figueroa C, Johnson C, Verster A, et al. Attitudes and Acceptability on HIV Self-testing Among Key Populations: A Literature Review. Attitudes and Acceptability on HIV Self-testing Among Key Populations: A Literature Review 2015.20.Bavinton BR, Brown G, Hurley M, et al. Which gay men would increase their frequency of HIV testing with home self-testing? Which gay men would increase their frequency of HIV testing with home self-testing? 2013;17:2084-92.21.Hensen B, Lewis JJ, Schaap A, et al. Factors associated with HIV-testing and acceptance of an offer of home-based testing by men in rural Zambia. Factors associated with HIV-testing and acceptance of an offer of home-based testing by men in rural Zambia 2015;19:492-504.22.Lee VJ, Tan SC, Earnest A, et al. User acceptability and feasibility of self-testing with HIV rapid tests. User acceptability and feasibility of self-testing with HIV rapid tests 2007;45:449-53.23.Terris-Prestholt F, Hanson K, MacPhail C, et al. How much demand for New HIV prevention technologies can we really expect? Results from a discrete choice experiment in South Africa. How much demand for New HIV prevention technologies can we really expect? Results from a discrete choice experiment in South Africa 2013;8:e83193.24.Ostermann J, Njau B, Brown DS, et al. Heterogeneous HIV Testing Preferences in an Urban Setting in Tanzania: Results from a Discrete Choice Experiment. Heterogeneous HIV Testing Preferences in an Urban Setting in Tanzania: Results from a Discrete Choice Experiment 2014;9:e92100.25.Phillips KA, Maddala T, and Johnson FR. Measuring preferences for health care interventions using conjoint analysis: an application to HIV testing. Measuring preferences for health care interventions using conjoint analysis: an application to HIV testing 2002;37:1681-1705.26.Ryan M. Discrete choice experiments in health care. BMJ 2004;328:360-361.27.Terris-Prestholt F, Quaiffe M, and Vickerman P. Parameterising user uptake in economic evaluations: the role of discrete choice experiments. Parameterising user uptake in economic evaluations: the role of discrete choice experiments In press.28.Philip P, Hickson F, Bonell C, et al. Men who have sex with men in Britain: comparison of estimates from a probability sample and community-based surveys. Men who have sex with men in Britain: comparison of estimates from a probability sample and community-based surveys In submission.29.Orme B. Sample size issues for conjoint analysis, in Getting started with conjoint analysis: strategies for product design and pricing research. 2010, Research Publishers LLC: Madison, Wisconson.)*[.](1.Public Health England. HIV in the United Kingdom: 2014 report. 2014.2.Public Health England. HIV new diagnosis, treatment and care: 2015 report.3.Williamson LM, Dodds JP, Mercey DE, et al. Sexual risk behaviour and knowledge of HIV status among community samples of gay men in the UK. Sexual risk behaviour and knowledge of HIV status among community samples of gay men in the UK 2008;22:1063-70.4.Holt M, Rawstorne P, Wilkinson J, et al. HIV testing, gay community involvement and internet use: social and behavioural correlates of HIV testing among Australian men who have sex with men. HIV testing, gay community involvement and internet use: social and behavioural correlates of HIV testing among Australian men who have sex with men 2012;16:13-22.5.Zablotska I, Holt M, de Wit J, et al. Gay men who are not getting tested for HIV. Gay men who are not getting tested for HIV 2012;16:1887-94.6.Witzel TC, Melendez-Torres GJ, Hickson F, et al. HIV testing history and preferences for future tests among gay men, bisexual men and other MSM in England. HIV testing history and preferences for future tests among gay men, bisexual men and other MSM in England In submission.7.UNSW Australia. 2015. Annual report of trends in behaviour 2015. HIV/AIDS, hepatitis and sexually transmissible infections in Australia [Online]. Available: https://csrh.arts.unsw.edu.au/media/CSRHFile/CSRH_Annual_Report_of_Trends_in_Behaviour_2015.pdf.8.Phillips AN, Cambiano V, Miners A, et al. Potential impact on HIV incidence of higher HIV testing rates and earlier antiretroviral therapy initiation in MSM. Potential impact on HIV incidence of higher HIV testing rates and earlier antiretroviral therapy initiation in MSM 2015;29:1855-62.9.Nakagawa F, Lodwick RK, Smith CJ, et al. Projected life expectancy of people with HIV according to timing of diagnosis. Projected life expectancy of people with HIV according to timing of diagnosis 2012;26:335-43.10.Chadborn TR, Delpech VC, Sabin CA, et al. The late diagnosis and consequent short-term mortality of HIV-infected heterosexuals (England and Wales, 2000–2004). The late diagnosis and consequent short-term mortality of HIV-infected heterosexuals (England and Wales, 2000–2004) 2006;20:2371-2379.11.Miners A, Phillips A, Kreif N, et al. Health-related quality-of-life of people with HIV in the era of combination antiretroviral treatment: a cross-sectional comparison with the general population. Health-related quality-of-life of people with HIV in the era of combination antiretroviral treatment: a cross-sectional comparison with the general population 2014;1:e32–e40.12.Cohen MS, Chen YQ, McCauley M, et al. Prevention of HIV-1 Infection with Early Antiretroviral Therapy. Prevention of HIV-1 Infection with Early Antiretroviral Therapy 2011;365:493-505.13.Marks G, Crepaz N, and Janssen RS. Estimating sexual transmission of HIV from persons aware and unaware that they are infected with the virus in the USA. Estimating sexual transmission of HIV from persons aware and unaware that they are infected with the virus in the USA 2006;20:1447-50.14.Nakagawa F, Miners A, Smith CJ, et al. Projected Lifetime Healthcare Costs Associated with HIV Infection. Projected Lifetime Healthcare Costs Associated with HIV Infection 2015;10:e0125018.15.Fleishman JA, Yehia BR, Moore RD, et al. The Economic Burden of Late Entry Into Medical Care for Patients With HIV Infection. The Economic Burden of Late Entry Into Medical Care for Patients With HIV Infection 2010;48:1071-1079.16.Krentz HB and Gill MJ. The Direct Medical Costs of Late Presentation (<350/mm(3)) of HIV Infection over a 15-Year Period. The Direct Medical Costs of Late Presentation (<350/mm(3)) of HIV Infection over a 15-Year Period 2012;2012:757135.17.Krause J, Subklew-Sehume F, Kenyon C, et al. Acceptability of HIV self-testing: a systematic literature review. Acceptability of HIV self-testing: a systematic literature review 2013;13:735.18.Frye V, Wilton L, Hirshfied S, et al. \"Just Because It's Out There, People Aren't Going to Use It.\" HIV Self-Testing Among Young, Black MSM, and Transgender Women. \"Just Because It's Out There, People Aren't Going to Use It.\" HIV Self-Testing Among Young, Black MSM, and Transgender Women 2015.19.Figueroa C, Johnson C, Verster A, et al. Attitudes and Acceptability on HIV Self-testing Among Key Populations: A Literature Review. Attitudes and Acceptability on HIV Self-testing Among Key Populations: A Literature Review 2015.20.Bavinton BR, Brown G, Hurley M, et al. Which gay men would increase their frequency of HIV testing with home self-testing? Which gay men would increase their frequency of HIV testing with home self-testing? 2013;17:2084-92.21.Hensen B, Lewis JJ, Schaap A, et al. Factors associated with HIV-testing and acceptance of an offer of home-based testing by men in rural Zambia. Factors associated with HIV-testing and acceptance of an offer of home-based testing by men in rural Zambia 2015;19:492-504.22.Lee VJ, Tan SC, Earnest A, et al. User acceptability and feasibility of self-testing with HIV rapid tests. User acceptability and feasibility of self-testing with HIV rapid tests 2007;45:449-53.23.Terris-Prestholt F, Hanson K, MacPhail C, et al. How much demand for New HIV prevention technologies can we really expect? Results from a discrete choice experiment in South Africa. How much demand for New HIV prevention technologies can we really expect? Results from a discrete choice experiment in South Africa 2013;8:e83193.24.Ostermann J, Njau B, Brown DS, et al. Heterogeneous HIV Testing Preferences in an Urban Setting in Tanzania: Results from a Discrete Choice Experiment. Heterogeneous HIV Testing Preferences in an Urban Setting in Tanzania: Results from a Discrete Choice Experiment 2014;9:e92100.25.Phillips KA, Maddala T, and Johnson FR. Measuring preferences for health care interventions using conjoint analysis: an application to HIV testing. Measuring preferences for health care interventions using conjoint analysis: an application to HIV testing 2002;37:1681-1705.26.Ryan M. Discrete choice experiments in health care. BMJ 2004;328:360-361.27.Terris-Prestholt F, Quaiffe M, and Vickerman P. Parameterising user uptake in economic evaluations: the role of discrete choice experiments. Parameterising user uptake in economic evaluations: the role of discrete choice experiments In press.28.Philip P, Hickson F, Bonell C, et al. Men who have sex with men in Britain: comparison of estimates from a probability sample and community-based surveys. Men who have sex with men in Britain: comparison of estimates from a probability sample and community-based surveys In submission.29.Orme B. Sample size issues for conjoint analysis, in Getting started with conjoint analysis: strategies for product design and pricing research. 2010, Research Publishers LLC: Madison, Wisconson.)**[Potential impact on HIV incidence of higher HIV testing rates and earlier antiretroviral therapy initiation in MSM](1.Public Health England. HIV in the United Kingdom: 2014 report. 2014.2.Public Health England. HIV new diagnosis, treatment and care: 2015 report.3.Williamson LM, Dodds JP, Mercey DE, et al. Sexual risk behaviour and knowledge of HIV status among community samples of gay men in the UK. Sexual risk behaviour and knowledge of HIV status among community samples of gay men in the UK 2008;22:1063-70.4.Holt M, Rawstorne P, Wilkinson J, et al. HIV testing, gay community involvement and internet use: social and behavioural correlates of HIV testing among Australian men who have sex with men. HIV testing, gay community involvement and internet use: social and behavioural correlates of HIV testing among Australian men who have sex with men 2012;16:13-22.5.Zablotska I, Holt M, de Wit J, et al. Gay men who are not getting tested for HIV. Gay men who are not getting tested for HIV 2012;16:1887-94.6.Witzel TC, Melendez-Torres GJ, Hickson F, et al. HIV testing history and preferences for future tests among gay men, bisexual men and other MSM in England. HIV testing history and preferences for future tests among gay men, bisexual men and other MSM in England In submission.7.UNSW Australia. 2015. Annual report of trends in behaviour 2015. HIV/AIDS, hepatitis and sexually transmissible infections in Australia [Online]. Available: https://csrh.arts.unsw.edu.au/media/CSRHFile/CSRH_Annual_Report_of_Trends_in_Behaviour_2015.pdf.8.Phillips AN, Cambiano V, Miners A, et al. Potential impact on HIV incidence of higher HIV testing rates and earlier antiretroviral therapy initiation in MSM. Potential impact on HIV incidence of higher HIV testing rates and earlier antiretroviral therapy initiation in MSM 2015;29:1855-62.9.Nakagawa F, Lodwick RK, Smith CJ, et al. Projected life expectancy of people with HIV according to timing of diagnosis. Projected life expectancy of people with HIV according to timing of diagnosis 2012;26:335-43.10.Chadborn TR, Delpech VC, Sabin CA, et al. The late diagnosis and consequent short-term mortality of HIV-infected heterosexuals (England and Wales, 2000–2004). The late diagnosis and consequent short-term mortality of HIV-infected heterosexuals (England and Wales, 2000–2004) 2006;20:2371-2379.11.Miners A, Phillips A, Kreif N, et al. Health-related quality-of-life of people with HIV in the era of combination antiretroviral treatment: a cross-sectional comparison with the general population. Health-related quality-of-life of people with HIV in the era of combination antiretroviral treatment: a cross-sectional comparison with the general population 2014;1:e32–e40.12.Cohen MS, Chen YQ, McCauley M, et al. Prevention of HIV-1 Infection with Early Antiretroviral Therapy. Prevention of HIV-1 Infection with Early Antiretroviral Therapy 2011;365:493-505.13.Marks G, Crepaz N, and Janssen RS. Estimating sexual transmission of HIV from persons aware and unaware that they are infected with the virus in the USA. Estimating sexual transmission of HIV from persons aware and unaware that they are infected with the virus in the USA 2006;20:1447-50.14.Nakagawa F, Miners A, Smith CJ, et al. Projected Lifetime Healthcare Costs Associated with HIV Infection. Projected Lifetime Healthcare Costs Associated with HIV Infection 2015;10:e0125018.15.Fleishman JA, Yehia BR, Moore RD, et al. The Economic Burden of Late Entry Into Medical Care for Patients With HIV Infection. The Economic Burden of Late Entry Into Medical Care for Patients With HIV Infection 2010;48:1071-1079.16.Krentz HB and Gill MJ. The Direct Medical Costs of Late Presentation (<350/mm(3)) of HIV Infection over a 15-Year Period. The Direct Medical Costs of Late Presentation (<350/mm(3)) of HIV Infection over a 15-Year Period 2012;2012:757135.17.Krause J, Subklew-Sehume F, Kenyon C, et al. Acceptability of HIV self-testing: a systematic literature review. Acceptability of HIV self-testing: a systematic literature review 2013;13:735.18.Frye V, Wilton L, Hirshfied S, et al. \"Just Because It's Out There, People Aren't Going to Use It.\" HIV Self-Testing Among Young, Black MSM, and Transgender Women. \"Just Because It's Out There, People Aren't Going to Use It.\" HIV Self-Testing Among Young, Black MSM, and Transgender Women 2015.19.Figueroa C, Johnson C, Verster A, et al. Attitudes and Acceptability on HIV Self-testing Among Key Populations: A Literature Review. Attitudes and Acceptability on HIV Self-testing Among Key Populations: A Literature Review 2015.20.Bavinton BR, Brown G, Hurley M, et al. Which gay men would increase their frequency of HIV testing with home self-testing? Which gay men would increase their frequency of HIV testing with home self-testing? 2013;17:2084-92.21.Hensen B, Lewis JJ, Schaap A, et al. Factors associated with HIV-testing and acceptance of an offer of home-based testing by men in rural Zambia. Factors associated with HIV-testing and acceptance of an offer of home-based testing by men in rural Zambia 2015;19:492-504.22.Lee VJ, Tan SC, Earnest A, et al. User acceptability and feasibility of self-testing with HIV rapid tests. User acceptability and feasibility of self-testing with HIV rapid tests 2007;45:449-53.23.Terris-Prestholt F, Hanson K, MacPhail C, et al. How much demand for New HIV prevention technologies can we really expect? Results from a discrete choice experiment in South Africa. How much demand for New HIV prevention technologies can we really expect? Results from a discrete choice experiment in South Africa 2013;8:e83193.24.Ostermann J, Njau B, Brown DS, et al. Heterogeneous HIV Testing Preferences in an Urban Setting in Tanzania: Results from a Discrete Choice Experiment. Heterogeneous HIV Testing Preferences in an Urban Setting in Tanzania: Results from a Discrete Choice Experiment 2014;9:e92100.25.Phillips KA, Maddala T, and Johnson FR. Measuring preferences for health care interventions using conjoint analysis: an application to HIV testing. Measuring preferences for health care interventions using conjoint analysis: an application to HIV testing 2002;37:1681-1705.26.Ryan M. Discrete choice experiments in health care. BMJ 2004;328:360-361.27.Terris-Prestholt F, Quaiffe M, and Vickerman P. Parameterising user uptake in economic evaluations: the role of discrete choice experiments. Parameterising user uptake in economic evaluations: the role of discrete choice experiments In press.28.Philip P, Hickson F, Bonell C, et al. Men who have sex with men in Britain: comparison of estimates from a probability sample and community-based surveys. Men who have sex with men in Britain: comparison of estimates from a probability sample and community-based surveys In submission.29.Orme B. Sample size issues for conjoint analysis, in Getting started with conjoint analysis: strategies for product design and pricing research. 2010, Research Publishers LLC: Madison, Wisconson.)* [2015;](1.Public Health England. HIV in the United Kingdom: 2014 report. 2014.2.Public Health England. HIV new diagnosis, treatment and care: 2015 report.3.Williamson LM, Dodds JP, Mercey DE, et al. Sexual risk behaviour and knowledge of HIV status among community samples of gay men in the UK. Sexual risk behaviour and knowledge of HIV status among community samples of gay men in the UK 2008;22:1063-70.4.Holt M, Rawstorne P, Wilkinson J, et al. HIV testing, gay community involvement and internet use: social and behavioural correlates of HIV testing among Australian men who have sex with men. HIV testing, gay community involvement and internet use: social and behavioural correlates of HIV testing among Australian men who have sex with men 2012;16:13-22.5.Zablotska I, Holt M, de Wit J, et al. Gay men who are not getting tested for HIV. Gay men who are not getting tested for HIV 2012;16:1887-94.6.Witzel TC, Melendez-Torres GJ, Hickson F, et al. HIV testing history and preferences for future tests among gay men, bisexual men and other MSM in England. HIV testing history and preferences for future tests among gay men, bisexual men and other MSM in England In submission.7.UNSW Australia. 2015. Annual report of trends in behaviour 2015. HIV/AIDS, hepatitis and sexually transmissible infections in Australia [Online]. Available: https://csrh.arts.unsw.edu.au/media/CSRHFile/CSRH_Annual_Report_of_Trends_in_Behaviour_2015.pdf.8.Phillips AN, Cambiano V, Miners A, et al. Potential impact on HIV incidence of higher HIV testing rates and earlier antiretroviral therapy initiation in MSM. Potential impact on HIV incidence of higher HIV testing rates and earlier antiretroviral therapy initiation in MSM 2015;29:1855-62.9.Nakagawa F, Lodwick RK, Smith CJ, et al. Projected life expectancy of people with HIV according to timing of diagnosis. Projected life expectancy of people with HIV according to timing of diagnosis 2012;26:335-43.10.Chadborn TR, Delpech VC, Sabin CA, et al. The late diagnosis and consequent short-term mortality of HIV-infected heterosexuals (England and Wales, 2000–2004). The late diagnosis and consequent short-term mortality of HIV-infected heterosexuals (England and Wales, 2000–2004) 2006;20:2371-2379.11.Miners A, Phillips A, Kreif N, et al. Health-related quality-of-life of people with HIV in the era of combination antiretroviral treatment: a cross-sectional comparison with the general population. Health-related quality-of-life of people with HIV in the era of combination antiretroviral treatment: a cross-sectional comparison with the general population 2014;1:e32–e40.12.Cohen MS, Chen YQ, McCauley M, et al. Prevention of HIV-1 Infection with Early Antiretroviral Therapy. Prevention of HIV-1 Infection with Early Antiretroviral Therapy 2011;365:493-505.13.Marks G, Crepaz N, and Janssen RS. Estimating sexual transmission of HIV from persons aware and unaware that they are infected with the virus in the USA. Estimating sexual transmission of HIV from persons aware and unaware that they are infected with the virus in the USA 2006;20:1447-50.14.Nakagawa F, Miners A, Smith CJ, et al. Projected Lifetime Healthcare Costs Associated with HIV Infection. Projected Lifetime Healthcare Costs Associated with HIV Infection 2015;10:e0125018.15.Fleishman JA, Yehia BR, Moore RD, et al. The Economic Burden of Late Entry Into Medical Care for Patients With HIV Infection. The Economic Burden of Late Entry Into Medical Care for Patients With HIV Infection 2010;48:1071-1079.16.Krentz HB and Gill MJ. The Direct Medical Costs of Late Presentation (<350/mm(3)) of HIV Infection over a 15-Year Period. The Direct Medical Costs of Late Presentation (<350/mm(3)) of HIV Infection over a 15-Year Period 2012;2012:757135.17.Krause J, Subklew-Sehume F, Kenyon C, et al. Acceptability of HIV self-testing: a systematic literature review. Acceptability of HIV self-testing: a systematic literature review 2013;13:735.18.Frye V, Wilton L, Hirshfied S, et al. \"Just Because It's Out There, People Aren't Going to Use It.\" HIV Self-Testing Among Young, Black MSM, and Transgender Women. \"Just Because It's Out There, People Aren't Going to Use It.\" HIV Self-Testing Among Young, Black MSM, and Transgender Women 2015.19.Figueroa C, Johnson C, Verster A, et al. Attitudes and Acceptability on HIV Self-testing Among Key Populations: A Literature Review. Attitudes and Acceptability on HIV Self-testing Among Key Populations: A Literature Review 2015.20.Bavinton BR, Brown G, Hurley M, et al. Which gay men would increase their frequency of HIV testing with home self-testing? Which gay men would increase their frequency of HIV testing with home self-testing? 2013;17:2084-92.21.Hensen B, Lewis JJ, Schaap A, et al. Factors associated with HIV-testing and acceptance of an offer of home-based testing by men in rural Zambia. Factors associated with HIV-testing and acceptance of an offer of home-based testing by men in rural Zambia 2015;19:492-504.22.Lee VJ, Tan SC, Earnest A, et al. User acceptability and feasibility of self-testing with HIV rapid tests. User acceptability and feasibility of self-testing with HIV rapid tests 2007;45:449-53.23.Terris-Prestholt F, Hanson K, MacPhail C, et al. How much demand for New HIV prevention technologies can we really expect? Results from a discrete choice experiment in South Africa. How much demand for New HIV prevention technologies can we really expect? Results from a discrete choice experiment in South Africa 2013;8:e83193.24.Ostermann J, Njau B, Brown DS, et al. Heterogeneous HIV Testing Preferences in an Urban Setting in Tanzania: Results from a Discrete Choice Experiment. Heterogeneous HIV Testing Preferences in an Urban Setting in Tanzania: Results from a Discrete Choice Experiment 2014;9:e92100.25.Phillips KA, Maddala T, and Johnson FR. Measuring preferences for health care interventions using conjoint analysis: an application to HIV testing. Measuring preferences for health care interventions using conjoint analysis: an application to HIV testing 2002;37:1681-1705.26.Ryan M. Discrete choice experiments in health care. BMJ 2004;328:360-361.27.Terris-Prestholt F, Quaiffe M, and Vickerman P. Parameterising user uptake in economic evaluations: the role of discrete choice experiments. Parameterising user uptake in economic evaluations: the role of discrete choice experiments In press.28.Philip P, Hickson F, Bonell C, et al. Men who have sex with men in Britain: comparison of estimates from a probability sample and community-based surveys. Men who have sex with men in Britain: comparison of estimates from a probability sample and community-based surveys In submission.29.Orme B. Sample size issues for conjoint analysis, in Getting started with conjoint analysis: strategies for product design and pricing research. 2010, Research Publishers LLC: Madison, Wisconson.)**[29](1.Public Health England. HIV in the United Kingdom: 2014 report. 2014.2.Public Health England. HIV new diagnosis, treatment and care: 2015 report.3.Williamson LM, Dodds JP, Mercey DE, et al. Sexual risk behaviour and knowledge of HIV status among community samples of gay men in the UK. Sexual risk behaviour and knowledge of HIV status among community samples of gay men in the UK 2008;22:1063-70.4.Holt M, Rawstorne P, Wilkinson J, et al. HIV testing, gay community involvement and internet use: social and behavioural correlates of HIV testing among Australian men who have sex with men. HIV testing, gay community involvement and internet use: social and behavioural correlates of HIV testing among Australian men who have sex with men 2012;16:13-22.5.Zablotska I, Holt M, de Wit J, et al. Gay men who are not getting tested for HIV. Gay men who are not getting tested for HIV 2012;16:1887-94.6.Witzel TC, Melendez-Torres GJ, Hickson F, et al. HIV testing history and preferences for future tests among gay men, bisexual men and other MSM in England. HIV testing history and preferences for future tests among gay men, bisexual men and other MSM in England In submission.7.UNSW Australia. 2015. Annual report of trends in behaviour 2015. HIV/AIDS, hepatitis and sexually transmissible infections in Australia [Online]. Available: https://csrh.arts.unsw.edu.au/media/CSRHFile/CSRH_Annual_Report_of_Trends_in_Behaviour_2015.pdf.8.Phillips AN, Cambiano V, Miners A, et al. Potential impact on HIV incidence of higher HIV testing rates and earlier antiretroviral therapy initiation in MSM. Potential impact on HIV incidence of higher HIV testing rates and earlier antiretroviral therapy initiation in MSM 2015;29:1855-62.9.Nakagawa F, Lodwick RK, Smith CJ, et al. Projected life expectancy of people with HIV according to timing of diagnosis. Projected life expectancy of people with HIV according to timing of diagnosis 2012;26:335-43.10.Chadborn TR, Delpech VC, Sabin CA, et al. The late diagnosis and consequent short-term mortality of HIV-infected heterosexuals (England and Wales, 2000–2004). The late diagnosis and consequent short-term mortality of HIV-infected heterosexuals (England and Wales, 2000–2004) 2006;20:2371-2379.11.Miners A, Phillips A, Kreif N, et al. Health-related quality-of-life of people with HIV in the era of combination antiretroviral treatment: a cross-sectional comparison with the general population. Health-related quality-of-life of people with HIV in the era of combination antiretroviral treatment: a cross-sectional comparison with the general population 2014;1:e32–e40.12.Cohen MS, Chen YQ, McCauley M, et al. Prevention of HIV-1 Infection with Early Antiretroviral Therapy. Prevention of HIV-1 Infection with Early Antiretroviral Therapy 2011;365:493-505.13.Marks G, Crepaz N, and Janssen RS. Estimating sexual transmission of HIV from persons aware and unaware that they are infected with the virus in the USA. Estimating sexual transmission of HIV from persons aware and unaware that they are infected with the virus in the USA 2006;20:1447-50.14.Nakagawa F, Miners A, Smith CJ, et al. Projected Lifetime Healthcare Costs Associated with HIV Infection. Projected Lifetime Healthcare Costs Associated with HIV Infection 2015;10:e0125018.15.Fleishman JA, Yehia BR, Moore RD, et al. The Economic Burden of Late Entry Into Medical Care for Patients With HIV Infection. The Economic Burden of Late Entry Into Medical Care for Patients With HIV Infection 2010;48:1071-1079.16.Krentz HB and Gill MJ. The Direct Medical Costs of Late Presentation (<350/mm(3)) of HIV Infection over a 15-Year Period. The Direct Medical Costs of Late Presentation (<350/mm(3)) of HIV Infection over a 15-Year Period 2012;2012:757135.17.Krause J, Subklew-Sehume F, Kenyon C, et al. Acceptability of HIV self-testing: a systematic literature review. Acceptability of HIV self-testing: a systematic literature review 2013;13:735.18.Frye V, Wilton L, Hirshfied S, et al. \"Just Because It's Out There, People Aren't Going to Use It.\" HIV Self-Testing Among Young, Black MSM, and Transgender Women. \"Just Because It's Out There, People Aren't Going to Use It.\" HIV Self-Testing Among Young, Black MSM, and Transgender Women 2015.19.Figueroa C, Johnson C, Verster A, et al. Attitudes and Acceptability on HIV Self-testing Among Key Populations: A Literature Review. Attitudes and Acceptability on HIV Self-testing Among Key Populations: A Literature Review 2015.20.Bavinton BR, Brown G, Hurley M, et al. Which gay men would increase their frequency of HIV testing with home self-testing? Which gay men would increase their frequency of HIV testing with home self-testing? 2013;17:2084-92.21.Hensen B, Lewis JJ, Schaap A, et al. Factors associated with HIV-testing and acceptance of an offer of home-based testing by men in rural Zambia. Factors associated with HIV-testing and acceptance of an offer of home-based testing by men in rural Zambia 2015;19:492-504.22.Lee VJ, Tan SC, Earnest A, et al. User acceptability and feasibility of self-testing with HIV rapid tests. User acceptability and feasibility of self-testing with HIV rapid tests 2007;45:449-53.23.Terris-Prestholt F, Hanson K, MacPhail C, et al. How much demand for New HIV prevention technologies can we really expect? Results from a discrete choice experiment in South Africa. How much demand for New HIV prevention technologies can we really expect? Results from a discrete choice experiment in South Africa 2013;8:e83193.24.Ostermann J, Njau B, Brown DS, et al. Heterogeneous HIV Testing Preferences in an Urban Setting in Tanzania: Results from a Discrete Choice Experiment. Heterogeneous HIV Testing Preferences in an Urban Setting in Tanzania: Results from a Discrete Choice Experiment 2014;9:e92100.25.Phillips KA, Maddala T, and Johnson FR. Measuring preferences for health care interventions using conjoint analysis: an application to HIV testing. Measuring preferences for health care interventions using conjoint analysis: an application to HIV testing 2002;37:1681-1705.26.Ryan M. Discrete choice experiments in health care. BMJ 2004;328:360-361.27.Terris-Prestholt F, Quaiffe M, and Vickerman P. Parameterising user uptake in economic evaluations: the role of discrete choice experiments. Parameterising user uptake in economic evaluations: the role of discrete choice experiments In press.28.Philip P, Hickson F, Bonell C, et al. Men who have sex with men in Britain: comparison of estimates from a probability sample and community-based surveys. Men who have sex with men in Britain: comparison of estimates from a probability sample and community-based surveys In submission.29.Orme B. Sample size issues for conjoint analysis, in Getting started with conjoint analysis: strategies for product design and pricing research. 2010, Research Publishers LLC: Madison, Wisconson.)**[:1855-62.](1.Public Health England. HIV in the United Kingdom: 2014 report. 2014.2.Public Health England. HIV new diagnosis, treatment and care: 2015 report.3.Williamson LM, Dodds JP, Mercey DE, et al. Sexual risk behaviour and knowledge of HIV status among community samples of gay men in the UK. Sexual risk behaviour and knowledge of HIV status among community samples of gay men in the UK 2008;22:1063-70.4.Holt M, Rawstorne P, Wilkinson J, et al. HIV testing, gay community involvement and internet use: social and behavioural correlates of HIV testing among Australian men who have sex with men. HIV testing, gay community involvement and internet use: social and behavioural correlates of HIV testing among Australian men who have sex with men 2012;16:13-22.5.Zablotska I, Holt M, de Wit J, et al. Gay men who are not getting tested for HIV. Gay men who are not getting tested for HIV 2012;16:1887-94.6.Witzel TC, Melendez-Torres GJ, Hickson F, et al. HIV testing history and preferences for future tests among gay men, bisexual men and other MSM in England. HIV testing history and preferences for future tests among gay men, bisexual men and other MSM in England In submission.7.UNSW Australia. 2015. Annual report of trends in behaviour 2015. HIV/AIDS, hepatitis and sexually transmissible infections in Australia [Online]. Available: https://csrh.arts.unsw.edu.au/media/CSRHFile/CSRH_Annual_Report_of_Trends_in_Behaviour_2015.pdf.8.Phillips AN, Cambiano V, Miners A, et al. Potential impact on HIV incidence of higher HIV testing rates and earlier antiretroviral therapy initiation in MSM. Potential impact on HIV incidence of higher HIV testing rates and earlier antiretroviral therapy initiation in MSM 2015;29:1855-62.9.Nakagawa F, Lodwick RK, Smith CJ, et al. Projected life expectancy of people with HIV according to timing of diagnosis. Projected life expectancy of people with HIV according to timing of diagnosis 2012;26:335-43.10.Chadborn TR, Delpech VC, Sabin CA, et al. The late diagnosis and consequent short-term mortality of HIV-infected heterosexuals (England and Wales, 2000–2004). The late diagnosis and consequent short-term mortality of HIV-infected heterosexuals (England and Wales, 2000–2004) 2006;20:2371-2379.11.Miners A, Phillips A, Kreif N, et al. Health-related quality-of-life of people with HIV in the era of combination antiretroviral treatment: a cross-sectional comparison with the general population. Health-related quality-of-life of people with HIV in the era of combination antiretroviral treatment: a cross-sectional comparison with the general population 2014;1:e32–e40.12.Cohen MS, Chen YQ, McCauley M, et al. Prevention of HIV-1 Infection with Early Antiretroviral Therapy. Prevention of HIV-1 Infection with Early Antiretroviral Therapy 2011;365:493-505.13.Marks G, Crepaz N, and Janssen RS. Estimating sexual transmission of HIV from persons aware and unaware that they are infected with the virus in the USA. Estimating sexual transmission of HIV from persons aware and unaware that they are infected with the virus in the USA 2006;20:1447-50.14.Nakagawa F, Miners A, Smith CJ, et al. Projected Lifetime Healthcare Costs Associated with HIV Infection. Projected Lifetime Healthcare Costs Associated with HIV Infection 2015;10:e0125018.15.Fleishman JA, Yehia BR, Moore RD, et al. The Economic Burden of Late Entry Into Medical Care for Patients With HIV Infection. The Economic Burden of Late Entry Into Medical Care for Patients With HIV Infection 2010;48:1071-1079.16.Krentz HB and Gill MJ. The Direct Medical Costs of Late Presentation (<350/mm(3)) of HIV Infection over a 15-Year Period. The Direct Medical Costs of Late Presentation (<350/mm(3)) of HIV Infection over a 15-Year Period 2012;2012:757135.17.Krause J, Subklew-Sehume F, Kenyon C, et al. Acceptability of HIV self-testing: a systematic literature review. Acceptability of HIV self-testing: a systematic literature review 2013;13:735.18.Frye V, Wilton L, Hirshfied S, et al. \"Just Because It's Out There, People Aren't Going to Use It.\" HIV Self-Testing Among Young, Black MSM, and Transgender Women. \"Just Because It's Out There, People Aren't Going to Use It.\" HIV Self-Testing Among Young, Black MSM, and Transgender Women 2015.19.Figueroa C, Johnson C, Verster A, et al. Attitudes and Acceptability on HIV Self-testing Among Key Populations: A Literature Review. Attitudes and Acceptability on HIV Self-testing Among Key Populations: A Literature Review 2015.20.Bavinton BR, Brown G, Hurley M, et al. Which gay men would increase their frequency of HIV testing with home self-testing? Which gay men would increase their frequency of HIV testing with home self-testing? 2013;17:2084-92.21.Hensen B, Lewis JJ, Schaap A, et al. Factors associated with HIV-testing and acceptance of an offer of home-based testing by men in rural Zambia. Factors associated with HIV-testing and acceptance of an offer of home-based testing by men in rural Zambia 2015;19:492-504.22.Lee VJ, Tan SC, Earnest A, et al. User acceptability and feasibility of self-testing with HIV rapid tests. User acceptability and feasibility of self-testing with HIV rapid tests 2007;45:449-53.23.Terris-Prestholt F, Hanson K, MacPhail C, et al. How much demand for New HIV prevention technologies can we really expect? Results from a discrete choice experiment in South Africa. How much demand for New HIV prevention technologies can we really expect? Results from a discrete choice experiment in South Africa 2013;8:e83193.24.Ostermann J, Njau B, Brown DS, et al. Heterogeneous HIV Testing Preferences in an Urban Setting in Tanzania: Results from a Discrete Choice Experiment. Heterogeneous HIV Testing Preferences in an Urban Setting in Tanzania: Results from a Discrete Choice Experiment 2014;9:e92100.25.Phillips KA, Maddala T, and Johnson FR. Measuring preferences for health care interventions using conjoint analysis: an application to HIV testing. Measuring preferences for health care interventions using conjoint analysis: an application to HIV testing 2002;37:1681-1705.26.Ryan M. Discrete choice experiments in health care. BMJ 2004;328:360-361.27.Terris-Prestholt F, Quaiffe M, and Vickerman P. Parameterising user uptake in economic evaluations: the role of discrete choice experiments. Parameterising user uptake in economic evaluations: the role of discrete choice experiments In press.28.Philip P, Hickson F, Bonell C, et al. Men who have sex with men in Britain: comparison of estimates from a probability sample and community-based surveys. Men who have sex with men in Britain: comparison of estimates from a probability sample and community-based surveys In submission.29.Orme B. Sample size issues for conjoint analysis, in Getting started with conjoint analysis: strategies for product design and pricing research. 2010, Research Publishers LLC: Madison, Wisconson.)

[9. Nakagawa F, Lodwick RK, Smith CJ, et al. Projected life expectancy of people with HIV according to timing of diagnosis](1.Public Health England. HIV in the United Kingdom: 2014 report. 2014.2.Public Health England. HIV new diagnosis, treatment and care: 2015 report.3.Williamson LM, Dodds JP, Mercey DE, et al. Sexual risk behaviour and knowledge of HIV status among community samples of gay men in the UK. Sexual risk behaviour and knowledge of HIV status among community samples of gay men in the UK 2008;22:1063-70.4.Holt M, Rawstorne P, Wilkinson J, et al. HIV testing, gay community involvement and internet use: social and behavioural correlates of HIV testing among Australian men who have sex with men. HIV testing, gay community involvement and internet use: social and behavioural correlates of HIV testing among Australian men who have sex with men 2012;16:13-22.5.Zablotska I, Holt M, de Wit J, et al. Gay men who are not getting tested for HIV. Gay men who are not getting tested for HIV 2012;16:1887-94.6.Witzel TC, Melendez-Torres GJ, Hickson F, et al. HIV testing history and preferences for future tests among gay men, bisexual men and other MSM in England. HIV testing history and preferences for future tests among gay men, bisexual men and other MSM in England In submission.7.UNSW Australia. 2015. Annual report of trends in behaviour 2015. HIV/AIDS, hepatitis and sexually transmissible infections in Australia [Online]. Available: https://csrh.arts.unsw.edu.au/media/CSRHFile/CSRH_Annual_Report_of_Trends_in_Behaviour_2015.pdf.8.Phillips AN, Cambiano V, Miners A, et al. Potential impact on HIV incidence of higher HIV testing rates and earlier antiretroviral therapy initiation in MSM. Potential impact on HIV incidence of higher HIV testing rates and earlier antiretroviral therapy initiation in MSM 2015;29:1855-62.9.Nakagawa F, Lodwick RK, Smith CJ, et al. Projected life expectancy of people with HIV according to timing of diagnosis. Projected life expectancy of people with HIV according to timing of diagnosis 2012;26:335-43.10.Chadborn TR, Delpech VC, Sabin CA, et al. The late diagnosis and consequent short-term mortality of HIV-infected heterosexuals (England and Wales, 2000–2004). The late diagnosis and consequent short-term mortality of HIV-infected heterosexuals (England and Wales, 2000–2004) 2006;20:2371-2379.11.Miners A, Phillips A, Kreif N, et al. Health-related quality-of-life of people with HIV in the era of combination antiretroviral treatment: a cross-sectional comparison with the general population. Health-related quality-of-life of people with HIV in the era of combination antiretroviral treatment: a cross-sectional comparison with the general population 2014;1:e32–e40.12.Cohen MS, Chen YQ, McCauley M, et al. Prevention of HIV-1 Infection with Early Antiretroviral Therapy. Prevention of HIV-1 Infection with Early Antiretroviral Therapy 2011;365:493-505.13.Marks G, Crepaz N, and Janssen RS. Estimating sexual transmission of HIV from persons aware and unaware that they are infected with the virus in the USA. Estimating sexual transmission of HIV from persons aware and unaware that they are infected with the virus in the USA 2006;20:1447-50.14.Nakagawa F, Miners A, Smith CJ, et al. Projected Lifetime Healthcare Costs Associated with HIV Infection. Projected Lifetime Healthcare Costs Associated with HIV Infection 2015;10:e0125018.15.Fleishman JA, Yehia BR, Moore RD, et al. The Economic Burden of Late Entry Into Medical Care for Patients With HIV Infection. The Economic Burden of Late Entry Into Medical Care for Patients With HIV Infection 2010;48:1071-1079.16.Krentz HB and Gill MJ. The Direct Medical Costs of Late Presentation (<350/mm(3)) of HIV Infection over a 15-Year Period. The Direct Medical Costs of Late Presentation (<350/mm(3)) of HIV Infection over a 15-Year Period 2012;2012:757135.17.Krause J, Subklew-Sehume F, Kenyon C, et al. Acceptability of HIV self-testing: a systematic literature review. Acceptability of HIV self-testing: a systematic literature review 2013;13:735.18.Frye V, Wilton L, Hirshfied S, et al. \"Just Because It's Out There, People Aren't Going to Use It.\" HIV Self-Testing Among Young, Black MSM, and Transgender Women. \"Just Because It's Out There, People Aren't Going to Use It.\" HIV Self-Testing Among Young, Black MSM, and Transgender Women 2015.19.Figueroa C, Johnson C, Verster A, et al. Attitudes and Acceptability on HIV Self-testing Among Key Populations: A Literature Review. Attitudes and Acceptability on HIV Self-testing Among Key Populations: A Literature Review 2015.20.Bavinton BR, Brown G, Hurley M, et al. Which gay men would increase their frequency of HIV testing with home self-testing? Which gay men would increase their frequency of HIV testing with home self-testing? 2013;17:2084-92.21.Hensen B, Lewis JJ, Schaap A, et al. Factors associated with HIV-testing and acceptance of an offer of home-based testing by men in rural Zambia. Factors associated with HIV-testing and acceptance of an offer of home-based testing by men in rural Zambia 2015;19:492-504.22.Lee VJ, Tan SC, Earnest A, et al. User acceptability and feasibility of self-testing with HIV rapid tests. User acceptability and feasibility of self-testing with HIV rapid tests 2007;45:449-53.23.Terris-Prestholt F, Hanson K, MacPhail C, et al. How much demand for New HIV prevention technologies can we really expect? Results from a discrete choice experiment in South Africa. How much demand for New HIV prevention technologies can we really expect? Results from a discrete choice experiment in South Africa 2013;8:e83193.24.Ostermann J, Njau B, Brown DS, et al. Heterogeneous HIV Testing Preferences in an Urban Setting in Tanzania: Results from a Discrete Choice Experiment. Heterogeneous HIV Testing Preferences in an Urban Setting in Tanzania: Results from a Discrete Choice Experiment 2014;9:e92100.25.Phillips KA, Maddala T, and Johnson FR. Measuring preferences for health care interventions using conjoint analysis: an application to HIV testing. Measuring preferences for health care interventions using conjoint analysis: an application to HIV testing 2002;37:1681-1705.26.Ryan M. Discrete choice experiments in health care. BMJ 2004;328:360-361.27.Terris-Prestholt F, Quaiffe M, and Vickerman P. Parameterising user uptake in economic evaluations: the role of discrete choice experiments. Parameterising user uptake in economic evaluations: the role of discrete choice experiments In press.28.Philip P, Hickson F, Bonell C, et al. Men who have sex with men in Britain: comparison of estimates from a probability sample and community-based surveys. Men who have sex with men in Britain: comparison of estimates from a probability sample and community-based surveys In submission.29.Orme B. Sample size issues for conjoint analysis, in Getting started with conjoint analysis: strategies for product design and pricing research. 2010, Research Publishers LLC: Madison, Wisconson.)*[.](1.Public Health England. HIV in the United Kingdom: 2014 report. 2014.2.Public Health England. HIV new diagnosis, treatment and care: 2015 report.3.Williamson LM, Dodds JP, Mercey DE, et al. Sexual risk behaviour and knowledge of HIV status among community samples of gay men in the UK. Sexual risk behaviour and knowledge of HIV status among community samples of gay men in the UK 2008;22:1063-70.4.Holt M, Rawstorne P, Wilkinson J, et al. HIV testing, gay community involvement and internet use: social and behavioural correlates of HIV testing among Australian men who have sex with men. HIV testing, gay community involvement and internet use: social and behavioural correlates of HIV testing among Australian men who have sex with men 2012;16:13-22.5.Zablotska I, Holt M, de Wit J, et al. Gay men who are not getting tested for HIV. Gay men who are not getting tested for HIV 2012;16:1887-94.6.Witzel TC, Melendez-Torres GJ, Hickson F, et al. HIV testing history and preferences for future tests among gay men, bisexual men and other MSM in England. HIV testing history and preferences for future tests among gay men, bisexual men and other MSM in England In submission.7.UNSW Australia. 2015. Annual report of trends in behaviour 2015. HIV/AIDS, hepatitis and sexually transmissible infections in Australia [Online]. Available: https://csrh.arts.unsw.edu.au/media/CSRHFile/CSRH_Annual_Report_of_Trends_in_Behaviour_2015.pdf.8.Phillips AN, Cambiano V, Miners A, et al. Potential impact on HIV incidence of higher HIV testing rates and earlier antiretroviral therapy initiation in MSM. Potential impact on HIV incidence of higher HIV testing rates and earlier antiretroviral therapy initiation in MSM 2015;29:1855-62.9.Nakagawa F, Lodwick RK, Smith CJ, et al. Projected life expectancy of people with HIV according to timing of diagnosis. Projected life expectancy of people with HIV according to timing of diagnosis 2012;26:335-43.10.Chadborn TR, Delpech VC, Sabin CA, et al. The late diagnosis and consequent short-term mortality of HIV-infected heterosexuals (England and Wales, 2000–2004). The late diagnosis and consequent short-term mortality of HIV-infected heterosexuals (England and Wales, 2000–2004) 2006;20:2371-2379.11.Miners A, Phillips A, Kreif N, et al. Health-related quality-of-life of people with HIV in the era of combination antiretroviral treatment: a cross-sectional comparison with the general population. Health-related quality-of-life of people with HIV in the era of combination antiretroviral treatment: a cross-sectional comparison with the general population 2014;1:e32–e40.12.Cohen MS, Chen YQ, McCauley M, et al. Prevention of HIV-1 Infection with Early Antiretroviral Therapy. Prevention of HIV-1 Infection with Early Antiretroviral Therapy 2011;365:493-505.13.Marks G, Crepaz N, and Janssen RS. Estimating sexual transmission of HIV from persons aware and unaware that they are infected with the virus in the USA. Estimating sexual transmission of HIV from persons aware and unaware that they are infected with the virus in the USA 2006;20:1447-50.14.Nakagawa F, Miners A, Smith CJ, et al. Projected Lifetime Healthcare Costs Associated with HIV Infection. Projected Lifetime Healthcare Costs Associated with HIV Infection 2015;10:e0125018.15.Fleishman JA, Yehia BR, Moore RD, et al. The Economic Burden of Late Entry Into Medical Care for Patients With HIV Infection. The Economic Burden of Late Entry Into Medical Care for Patients With HIV Infection 2010;48:1071-1079.16.Krentz HB and Gill MJ. The Direct Medical Costs of Late Presentation (<350/mm(3)) of HIV Infection over a 15-Year Period. The Direct Medical Costs of Late Presentation (<350/mm(3)) of HIV Infection over a 15-Year Period 2012;2012:757135.17.Krause J, Subklew-Sehume F, Kenyon C, et al. Acceptability of HIV self-testing: a systematic literature review. Acceptability of HIV self-testing: a systematic literature review 2013;13:735.18.Frye V, Wilton L, Hirshfied S, et al. \"Just Because It's Out There, People Aren't Going to Use It.\" HIV Self-Testing Among Young, Black MSM, and Transgender Women. \"Just Because It's Out There, People Aren't Going to Use It.\" HIV Self-Testing Among Young, Black MSM, and Transgender Women 2015.19.Figueroa C, Johnson C, Verster A, et al. Attitudes and Acceptability on HIV Self-testing Among Key Populations: A Literature Review. Attitudes and Acceptability on HIV Self-testing Among Key Populations: A Literature Review 2015.20.Bavinton BR, Brown G, Hurley M, et al. Which gay men would increase their frequency of HIV testing with home self-testing? Which gay men would increase their frequency of HIV testing with home self-testing? 2013;17:2084-92.21.Hensen B, Lewis JJ, Schaap A, et al. Factors associated with HIV-testing and acceptance of an offer of home-based testing by men in rural Zambia. Factors associated with HIV-testing and acceptance of an offer of home-based testing by men in rural Zambia 2015;19:492-504.22.Lee VJ, Tan SC, Earnest A, et al. User acceptability and feasibility of self-testing with HIV rapid tests. User acceptability and feasibility of self-testing with HIV rapid tests 2007;45:449-53.23.Terris-Prestholt F, Hanson K, MacPhail C, et al. How much demand for New HIV prevention technologies can we really expect? Results from a discrete choice experiment in South Africa. How much demand for New HIV prevention technologies can we really expect? Results from a discrete choice experiment in South Africa 2013;8:e83193.24.Ostermann J, Njau B, Brown DS, et al. Heterogeneous HIV Testing Preferences in an Urban Setting in Tanzania: Results from a Discrete Choice Experiment. Heterogeneous HIV Testing Preferences in an Urban Setting in Tanzania: Results from a Discrete Choice Experiment 2014;9:e92100.25.Phillips KA, Maddala T, and Johnson FR. Measuring preferences for health care interventions using conjoint analysis: an application to HIV testing. Measuring preferences for health care interventions using conjoint analysis: an application to HIV testing 2002;37:1681-1705.26.Ryan M. Discrete choice experiments in health care. BMJ 2004;328:360-361.27.Terris-Prestholt F, Quaiffe M, and Vickerman P. Parameterising user uptake in economic evaluations: the role of discrete choice experiments. Parameterising user uptake in economic evaluations: the role of discrete choice experiments In press.28.Philip P, Hickson F, Bonell C, et al. Men who have sex with men in Britain: comparison of estimates from a probability sample and community-based surveys. Men who have sex with men in Britain: comparison of estimates from a probability sample and community-based surveys In submission.29.Orme B. Sample size issues for conjoint analysis, in Getting started with conjoint analysis: strategies for product design and pricing research. 2010, Research Publishers LLC: Madison, Wisconson.)**[Projected life expectancy of people with HIV according to timing of diagnosis](1.Public Health England. HIV in the United Kingdom: 2014 report. 2014.2.Public Health England. HIV new diagnosis, treatment and care: 2015 report.3.Williamson LM, Dodds JP, Mercey DE, et al. Sexual risk behaviour and knowledge of HIV status among community samples of gay men in the UK. Sexual risk behaviour and knowledge of HIV status among community samples of gay men in the UK 2008;22:1063-70.4.Holt M, Rawstorne P, Wilkinson J, et al. HIV testing, gay community involvement and internet use: social and behavioural correlates of HIV testing among Australian men who have sex with men. HIV testing, gay community involvement and internet use: social and behavioural correlates of HIV testing among Australian men who have sex with men 2012;16:13-22.5.Zablotska I, Holt M, de Wit J, et al. Gay men who are not getting tested for HIV. Gay men who are not getting tested for HIV 2012;16:1887-94.6.Witzel TC, Melendez-Torres GJ, Hickson F, et al. HIV testing history and preferences for future tests among gay men, bisexual men and other MSM in England. HIV testing history and preferences for future tests among gay men, bisexual men and other MSM in England In submission.7.UNSW Australia. 2015. Annual report of trends in behaviour 2015. HIV/AIDS, hepatitis and sexually transmissible infections in Australia [Online]. Available: https://csrh.arts.unsw.edu.au/media/CSRHFile/CSRH_Annual_Report_of_Trends_in_Behaviour_2015.pdf.8.Phillips AN, Cambiano V, Miners A, et al. Potential impact on HIV incidence of higher HIV testing rates and earlier antiretroviral therapy initiation in MSM. Potential impact on HIV incidence of higher HIV testing rates and earlier antiretroviral therapy initiation in MSM 2015;29:1855-62.9.Nakagawa F, Lodwick RK, Smith CJ, et al. Projected life expectancy of people with HIV according to timing of diagnosis. Projected life expectancy of people with HIV according to timing of diagnosis 2012;26:335-43.10.Chadborn TR, Delpech VC, Sabin CA, et al. The late diagnosis and consequent short-term mortality of HIV-infected heterosexuals (England and Wales, 2000–2004). The late diagnosis and consequent short-term mortality of HIV-infected heterosexuals (England and Wales, 2000–2004) 2006;20:2371-2379.11.Miners A, Phillips A, Kreif N, et al. Health-related quality-of-life of people with HIV in the era of combination antiretroviral treatment: a cross-sectional comparison with the general population. Health-related quality-of-life of people with HIV in the era of combination antiretroviral treatment: a cross-sectional comparison with the general population 2014;1:e32–e40.12.Cohen MS, Chen YQ, McCauley M, et al. Prevention of HIV-1 Infection with Early Antiretroviral Therapy. Prevention of HIV-1 Infection with Early Antiretroviral Therapy 2011;365:493-505.13.Marks G, Crepaz N, and Janssen RS. Estimating sexual transmission of HIV from persons aware and unaware that they are infected with the virus in the USA. Estimating sexual transmission of HIV from persons aware and unaware that they are infected with the virus in the USA 2006;20:1447-50.14.Nakagawa F, Miners A, Smith CJ, et al. Projected Lifetime Healthcare Costs Associated with HIV Infection. Projected Lifetime Healthcare Costs Associated with HIV Infection 2015;10:e0125018.15.Fleishman JA, Yehia BR, Moore RD, et al. The Economic Burden of Late Entry Into Medical Care for Patients With HIV Infection. The Economic Burden of Late Entry Into Medical Care for Patients With HIV Infection 2010;48:1071-1079.16.Krentz HB and Gill MJ. The Direct Medical Costs of Late Presentation (<350/mm(3)) of HIV Infection over a 15-Year Period. The Direct Medical Costs of Late Presentation (<350/mm(3)) of HIV Infection over a 15-Year Period 2012;2012:757135.17.Krause J, Subklew-Sehume F, Kenyon C, et al. Acceptability of HIV self-testing: a systematic literature review. Acceptability of HIV self-testing: a systematic literature review 2013;13:735.18.Frye V, Wilton L, Hirshfied S, et al. \"Just Because It's Out There, People Aren't Going to Use It.\" HIV Self-Testing Among Young, Black MSM, and Transgender Women. \"Just Because It's Out There, People Aren't Going to Use It.\" HIV Self-Testing Among Young, Black MSM, and Transgender Women 2015.19.Figueroa C, Johnson C, Verster A, et al. Attitudes and Acceptability on HIV Self-testing Among Key Populations: A Literature Review. Attitudes and Acceptability on HIV Self-testing Among Key Populations: A Literature Review 2015.20.Bavinton BR, Brown G, Hurley M, et al. Which gay men would increase their frequency of HIV testing with home self-testing? Which gay men would increase their frequency of HIV testing with home self-testing? 2013;17:2084-92.21.Hensen B, Lewis JJ, Schaap A, et al. Factors associated with HIV-testing and acceptance of an offer of home-based testing by men in rural Zambia. Factors associated with HIV-testing and acceptance of an offer of home-based testing by men in rural Zambia 2015;19:492-504.22.Lee VJ, Tan SC, Earnest A, et al. User acceptability and feasibility of self-testing with HIV rapid tests. User acceptability and feasibility of self-testing with HIV rapid tests 2007;45:449-53.23.Terris-Prestholt F, Hanson K, MacPhail C, et al. How much demand for New HIV prevention technologies can we really expect? Results from a discrete choice experiment in South Africa. How much demand for New HIV prevention technologies can we really expect? Results from a discrete choice experiment in South Africa 2013;8:e83193.24.Ostermann J, Njau B, Brown DS, et al. Heterogeneous HIV Testing Preferences in an Urban Setting in Tanzania: Results from a Discrete Choice Experiment. Heterogeneous HIV Testing Preferences in an Urban Setting in Tanzania: Results from a Discrete Choice Experiment 2014;9:e92100.25.Phillips KA, Maddala T, and Johnson FR. Measuring preferences for health care interventions using conjoint analysis: an application to HIV testing. Measuring preferences for health care interventions using conjoint analysis: an application to HIV testing 2002;37:1681-1705.26.Ryan M. Discrete choice experiments in health care. BMJ 2004;328:360-361.27.Terris-Prestholt F, Quaiffe M, and Vickerman P. Parameterising user uptake in economic evaluations: the role of discrete choice experiments. Parameterising user uptake in economic evaluations: the role of discrete choice experiments In press.28.Philip P, Hickson F, Bonell C, et al. Men who have sex with men in Britain: comparison of estimates from a probability sample and community-based surveys. Men who have sex with men in Britain: comparison of estimates from a probability sample and community-based surveys In submission.29.Orme B. Sample size issues for conjoint analysis, in Getting started with conjoint analysis: strategies for product design and pricing research. 2010, Research Publishers LLC: Madison, Wisconson.)* [2012;](1.Public Health England. HIV in the United Kingdom: 2014 report. 2014.2.Public Health England. HIV new diagnosis, treatment and care: 2015 report.3.Williamson LM, Dodds JP, Mercey DE, et al. Sexual risk behaviour and knowledge of HIV status among community samples of gay men in the UK. Sexual risk behaviour and knowledge of HIV status among community samples of gay men in the UK 2008;22:1063-70.4.Holt M, Rawstorne P, Wilkinson J, et al. HIV testing, gay community involvement and internet use: social and behavioural correlates of HIV testing among Australian men who have sex with men. HIV testing, gay community involvement and internet use: social and behavioural correlates of HIV testing among Australian men who have sex with men 2012;16:13-22.5.Zablotska I, Holt M, de Wit J, et al. Gay men who are not getting tested for HIV. Gay men who are not getting tested for HIV 2012;16:1887-94.6.Witzel TC, Melendez-Torres GJ, Hickson F, et al. HIV testing history and preferences for future tests among gay men, bisexual men and other MSM in England. HIV testing history and preferences for future tests among gay men, bisexual men and other MSM in England In submission.7.UNSW Australia. 2015. Annual report of trends in behaviour 2015. HIV/AIDS, hepatitis and sexually transmissible infections in Australia [Online]. Available: https://csrh.arts.unsw.edu.au/media/CSRHFile/CSRH_Annual_Report_of_Trends_in_Behaviour_2015.pdf.8.Phillips AN, Cambiano V, Miners A, et al. Potential impact on HIV incidence of higher HIV testing rates and earlier antiretroviral therapy initiation in MSM. Potential impact on HIV incidence of higher HIV testing rates and earlier antiretroviral therapy initiation in MSM 2015;29:1855-62.9.Nakagawa F, Lodwick RK, Smith CJ, et al. Projected life expectancy of people with HIV according to timing of diagnosis. Projected life expectancy of people with HIV according to timing of diagnosis 2012;26:335-43.10.Chadborn TR, Delpech VC, Sabin CA, et al. The late diagnosis and consequent short-term mortality of HIV-infected heterosexuals (England and Wales, 2000–2004). The late diagnosis and consequent short-term mortality of HIV-infected heterosexuals (England and Wales, 2000–2004) 2006;20:2371-2379.11.Miners A, Phillips A, Kreif N, et al. Health-related quality-of-life of people with HIV in the era of combination antiretroviral treatment: a cross-sectional comparison with the general population. Health-related quality-of-life of people with HIV in the era of combination antiretroviral treatment: a cross-sectional comparison with the general population 2014;1:e32–e40.12.Cohen MS, Chen YQ, McCauley M, et al. Prevention of HIV-1 Infection with Early Antiretroviral Therapy. Prevention of HIV-1 Infection with Early Antiretroviral Therapy 2011;365:493-505.13.Marks G, Crepaz N, and Janssen RS. Estimating sexual transmission of HIV from persons aware and unaware that they are infected with the virus in the USA. Estimating sexual transmission of HIV from persons aware and unaware that they are infected with the virus in the USA 2006;20:1447-50.14.Nakagawa F, Miners A, Smith CJ, et al. Projected Lifetime Healthcare Costs Associated with HIV Infection. Projected Lifetime Healthcare Costs Associated with HIV Infection 2015;10:e0125018.15.Fleishman JA, Yehia BR, Moore RD, et al. The Economic Burden of Late Entry Into Medical Care for Patients With HIV Infection. The Economic Burden of Late Entry Into Medical Care for Patients With HIV Infection 2010;48:1071-1079.16.Krentz HB and Gill MJ. The Direct Medical Costs of Late Presentation (<350/mm(3)) of HIV Infection over a 15-Year Period. The Direct Medical Costs of Late Presentation (<350/mm(3)) of HIV Infection over a 15-Year Period 2012;2012:757135.17.Krause J, Subklew-Sehume F, Kenyon C, et al. Acceptability of HIV self-testing: a systematic literature review. Acceptability of HIV self-testing: a systematic literature review 2013;13:735.18.Frye V, Wilton L, Hirshfied S, et al. \"Just Because It's Out There, People Aren't Going to Use It.\" HIV Self-Testing Among Young, Black MSM, and Transgender Women. \"Just Because It's Out There, People Aren't Going to Use It.\" HIV Self-Testing Among Young, Black MSM, and Transgender Women 2015.19.Figueroa C, Johnson C, Verster A, et al. Attitudes and Acceptability on HIV Self-testing Among Key Populations: A Literature Review. Attitudes and Acceptability on HIV Self-testing Among Key Populations: A Literature Review 2015.20.Bavinton BR, Brown G, Hurley M, et al. Which gay men would increase their frequency of HIV testing with home self-testing? Which gay men would increase their frequency of HIV testing with home self-testing? 2013;17:2084-92.21.Hensen B, Lewis JJ, Schaap A, et al. Factors associated with HIV-testing and acceptance of an offer of home-based testing by men in rural Zambia. Factors associated with HIV-testing and acceptance of an offer of home-based testing by men in rural Zambia 2015;19:492-504.22.Lee VJ, Tan SC, Earnest A, et al. User acceptability and feasibility of self-testing with HIV rapid tests. User acceptability and feasibility of self-testing with HIV rapid tests 2007;45:449-53.23.Terris-Prestholt F, Hanson K, MacPhail C, et al. How much demand for New HIV prevention technologies can we really expect? Results from a discrete choice experiment in South Africa. How much demand for New HIV prevention technologies can we really expect? Results from a discrete choice experiment in South Africa 2013;8:e83193.24.Ostermann J, Njau B, Brown DS, et al. Heterogeneous HIV Testing Preferences in an Urban Setting in Tanzania: Results from a Discrete Choice Experiment. Heterogeneous HIV Testing Preferences in an Urban Setting in Tanzania: Results from a Discrete Choice Experiment 2014;9:e92100.25.Phillips KA, Maddala T, and Johnson FR. Measuring preferences for health care interventions using conjoint analysis: an application to HIV testing. Measuring preferences for health care interventions using conjoint analysis: an application to HIV testing 2002;37:1681-1705.26.Ryan M. Discrete choice experiments in health care. BMJ 2004;328:360-361.27.Terris-Prestholt F, Quaiffe M, and Vickerman P. Parameterising user uptake in economic evaluations: the role of discrete choice experiments. Parameterising user uptake in economic evaluations: the role of discrete choice experiments In press.28.Philip P, Hickson F, Bonell C, et al. Men who have sex with men in Britain: comparison of estimates from a probability sample and community-based surveys. Men who have sex with men in Britain: comparison of estimates from a probability sample and community-based surveys In submission.29.Orme B. Sample size issues for conjoint analysis, in Getting started with conjoint analysis: strategies for product design and pricing research. 2010, Research Publishers LLC: Madison, Wisconson.)**[26](1.Public Health England. HIV in the United Kingdom: 2014 report. 2014.2.Public Health England. HIV new diagnosis, treatment and care: 2015 report.3.Williamson LM, Dodds JP, Mercey DE, et al. Sexual risk behaviour and knowledge of HIV status among community samples of gay men in the UK. Sexual risk behaviour and knowledge of HIV status among community samples of gay men in the UK 2008;22:1063-70.4.Holt M, Rawstorne P, Wilkinson J, et al. HIV testing, gay community involvement and internet use: social and behavioural correlates of HIV testing among Australian men who have sex with men. HIV testing, gay community involvement and internet use: social and behavioural correlates of HIV testing among Australian men who have sex with men 2012;16:13-22.5.Zablotska I, Holt M, de Wit J, et al. Gay men who are not getting tested for HIV. Gay men who are not getting tested for HIV 2012;16:1887-94.6.Witzel TC, Melendez-Torres GJ, Hickson F, et al. HIV testing history and preferences for future tests among gay men, bisexual men and other MSM in England. HIV testing history and preferences for future tests among gay men, bisexual men and other MSM in England In submission.7.UNSW Australia. 2015. Annual report of trends in behaviour 2015. HIV/AIDS, hepatitis and sexually transmissible infections in Australia [Online]. Available: https://csrh.arts.unsw.edu.au/media/CSRHFile/CSRH_Annual_Report_of_Trends_in_Behaviour_2015.pdf.8.Phillips AN, Cambiano V, Miners A, et al. Potential impact on HIV incidence of higher HIV testing rates and earlier antiretroviral therapy initiation in MSM. Potential impact on HIV incidence of higher HIV testing rates and earlier antiretroviral therapy initiation in MSM 2015;29:1855-62.9.Nakagawa F, Lodwick RK, Smith CJ, et al. Projected life expectancy of people with HIV according to timing of diagnosis. Projected life expectancy of people with HIV according to timing of diagnosis 2012;26:335-43.10.Chadborn TR, Delpech VC, Sabin CA, et al. The late diagnosis and consequent short-term mortality of HIV-infected heterosexuals (England and Wales, 2000–2004). The late diagnosis and consequent short-term mortality of HIV-infected heterosexuals (England and Wales, 2000–2004) 2006;20:2371-2379.11.Miners A, Phillips A, Kreif N, et al. Health-related quality-of-life of people with HIV in the era of combination antiretroviral treatment: a cross-sectional comparison with the general population. Health-related quality-of-life of people with HIV in the era of combination antiretroviral treatment: a cross-sectional comparison with the general population 2014;1:e32–e40.12.Cohen MS, Chen YQ, McCauley M, et al. Prevention of HIV-1 Infection with Early Antiretroviral Therapy. Prevention of HIV-1 Infection with Early Antiretroviral Therapy 2011;365:493-505.13.Marks G, Crepaz N, and Janssen RS. Estimating sexual transmission of HIV from persons aware and unaware that they are infected with the virus in the USA. Estimating sexual transmission of HIV from persons aware and unaware that they are infected with the virus in the USA 2006;20:1447-50.14.Nakagawa F, Miners A, Smith CJ, et al. Projected Lifetime Healthcare Costs Associated with HIV Infection. Projected Lifetime Healthcare Costs Associated with HIV Infection 2015;10:e0125018.15.Fleishman JA, Yehia BR, Moore RD, et al. The Economic Burden of Late Entry Into Medical Care for Patients With HIV Infection. The Economic Burden of Late Entry Into Medical Care for Patients With HIV Infection 2010;48:1071-1079.16.Krentz HB and Gill MJ. The Direct Medical Costs of Late Presentation (<350/mm(3)) of HIV Infection over a 15-Year Period. The Direct Medical Costs of Late Presentation (<350/mm(3)) of HIV Infection over a 15-Year Period 2012;2012:757135.17.Krause J, Subklew-Sehume F, Kenyon C, et al. Acceptability of HIV self-testing: a systematic literature review. Acceptability of HIV self-testing: a systematic literature review 2013;13:735.18.Frye V, Wilton L, Hirshfied S, et al. \"Just Because It's Out There, People Aren't Going to Use It.\" HIV Self-Testing Among Young, Black MSM, and Transgender Women. \"Just Because It's Out There, People Aren't Going to Use It.\" HIV Self-Testing Among Young, Black MSM, and Transgender Women 2015.19.Figueroa C, Johnson C, Verster A, et al. Attitudes and Acceptability on HIV Self-testing Among Key Populations: A Literature Review. Attitudes and Acceptability on HIV Self-testing Among Key Populations: A Literature Review 2015.20.Bavinton BR, Brown G, Hurley M, et al. Which gay men would increase their frequency of HIV testing with home self-testing? Which gay men would increase their frequency of HIV testing with home self-testing? 2013;17:2084-92.21.Hensen B, Lewis JJ, Schaap A, et al. Factors associated with HIV-testing and acceptance of an offer of home-based testing by men in rural Zambia. Factors associated with HIV-testing and acceptance of an offer of home-based testing by men in rural Zambia 2015;19:492-504.22.Lee VJ, Tan SC, Earnest A, et al. User acceptability and feasibility of self-testing with HIV rapid tests. User acceptability and feasibility of self-testing with HIV rapid tests 2007;45:449-53.23.Terris-Prestholt F, Hanson K, MacPhail C, et al. How much demand for New HIV prevention technologies can we really expect? Results from a discrete choice experiment in South Africa. How much demand for New HIV prevention technologies can we really expect? Results from a discrete choice experiment in South Africa 2013;8:e83193.24.Ostermann J, Njau B, Brown DS, et al. Heterogeneous HIV Testing Preferences in an Urban Setting in Tanzania: Results from a Discrete Choice Experiment. Heterogeneous HIV Testing Preferences in an Urban Setting in Tanzania: Results from a Discrete Choice Experiment 2014;9:e92100.25.Phillips KA, Maddala T, and Johnson FR. Measuring preferences for health care interventions using conjoint analysis: an application to HIV testing. Measuring preferences for health care interventions using conjoint analysis: an application to HIV testing 2002;37:1681-1705.26.Ryan M. Discrete choice experiments in health care. BMJ 2004;328:360-361.27.Terris-Prestholt F, Quaiffe M, and Vickerman P. Parameterising user uptake in economic evaluations: the role of discrete choice experiments. Parameterising user uptake in economic evaluations: the role of discrete choice experiments In press.28.Philip P, Hickson F, Bonell C, et al. Men who have sex with men in Britain: comparison of estimates from a probability sample and community-based surveys. Men who have sex with men in Britain: comparison of estimates from a probability sample and community-based surveys In submission.29.Orme B. Sample size issues for conjoint analysis, in Getting started with conjoint analysis: strategies for product design and pricing research. 2010, Research Publishers LLC: Madison, Wisconson.)**[:335-43.](1.Public Health England. HIV in the United Kingdom: 2014 report. 2014.2.Public Health England. HIV new diagnosis, treatment and care: 2015 report.3.Williamson LM, Dodds JP, Mercey DE, et al. Sexual risk behaviour and knowledge of HIV status among community samples of gay men in the UK. Sexual risk behaviour and knowledge of HIV status among community samples of gay men in the UK 2008;22:1063-70.4.Holt M, Rawstorne P, Wilkinson J, et al. HIV testing, gay community involvement and internet use: social and behavioural correlates of HIV testing among Australian men who have sex with men. HIV testing, gay community involvement and internet use: social and behavioural correlates of HIV testing among Australian men who have sex with men 2012;16:13-22.5.Zablotska I, Holt M, de Wit J, et al. Gay men who are not getting tested for HIV. Gay men who are not getting tested for HIV 2012;16:1887-94.6.Witzel TC, Melendez-Torres GJ, Hickson F, et al. HIV testing history and preferences for future tests among gay men, bisexual men and other MSM in England. HIV testing history and preferences for future tests among gay men, bisexual men and other MSM in England In submission.7.UNSW Australia. 2015. Annual report of trends in behaviour 2015. HIV/AIDS, hepatitis and sexually transmissible infections in Australia [Online]. Available: https://csrh.arts.unsw.edu.au/media/CSRHFile/CSRH_Annual_Report_of_Trends_in_Behaviour_2015.pdf.8.Phillips AN, Cambiano V, Miners A, et al. Potential impact on HIV incidence of higher HIV testing rates and earlier antiretroviral therapy initiation in MSM. Potential impact on HIV incidence of higher HIV testing rates and earlier antiretroviral therapy initiation in MSM 2015;29:1855-62.9.Nakagawa F, Lodwick RK, Smith CJ, et al. Projected life expectancy of people with HIV according to timing of diagnosis. Projected life expectancy of people with HIV according to timing of diagnosis 2012;26:335-43.10.Chadborn TR, Delpech VC, Sabin CA, et al. The late diagnosis and consequent short-term mortality of HIV-infected heterosexuals (England and Wales, 2000–2004). The late diagnosis and consequent short-term mortality of HIV-infected heterosexuals (England and Wales, 2000–2004) 2006;20:2371-2379.11.Miners A, Phillips A, Kreif N, et al. Health-related quality-of-life of people with HIV in the era of combination antiretroviral treatment: a cross-sectional comparison with the general population. Health-related quality-of-life of people with HIV in the era of combination antiretroviral treatment: a cross-sectional comparison with the general population 2014;1:e32–e40.12.Cohen MS, Chen YQ, McCauley M, et al. Prevention of HIV-1 Infection with Early Antiretroviral Therapy. Prevention of HIV-1 Infection with Early Antiretroviral Therapy 2011;365:493-505.13.Marks G, Crepaz N, and Janssen RS. Estimating sexual transmission of HIV from persons aware and unaware that they are infected with the virus in the USA. Estimating sexual transmission of HIV from persons aware and unaware that they are infected with the virus in the USA 2006;20:1447-50.14.Nakagawa F, Miners A, Smith CJ, et al. Projected Lifetime Healthcare Costs Associated with HIV Infection. Projected Lifetime Healthcare Costs Associated with HIV Infection 2015;10:e0125018.15.Fleishman JA, Yehia BR, Moore RD, et al. The Economic Burden of Late Entry Into Medical Care for Patients With HIV Infection. The Economic Burden of Late Entry Into Medical Care for Patients With HIV Infection 2010;48:1071-1079.16.Krentz HB and Gill MJ. The Direct Medical Costs of Late Presentation (<350/mm(3)) of HIV Infection over a 15-Year Period. The Direct Medical Costs of Late Presentation (<350/mm(3)) of HIV Infection over a 15-Year Period 2012;2012:757135.17.Krause J, Subklew-Sehume F, Kenyon C, et al. Acceptability of HIV self-testing: a systematic literature review. Acceptability of HIV self-testing: a systematic literature review 2013;13:735.18.Frye V, Wilton L, Hirshfied S, et al. \"Just Because It's Out There, People Aren't Going to Use It.\" HIV Self-Testing Among Young, Black MSM, and Transgender Women. \"Just Because It's Out There, People Aren't Going to Use It.\" HIV Self-Testing Among Young, Black MSM, and Transgender Women 2015.19.Figueroa C, Johnson C, Verster A, et al. Attitudes and Acceptability on HIV Self-testing Among Key Populations: A Literature Review. Attitudes and Acceptability on HIV Self-testing Among Key Populations: A Literature Review 2015.20.Bavinton BR, Brown G, Hurley M, et al. Which gay men would increase their frequency of HIV testing with home self-testing? Which gay men would increase their frequency of HIV testing with home self-testing? 2013;17:2084-92.21.Hensen B, Lewis JJ, Schaap A, et al. Factors associated with HIV-testing and acceptance of an offer of home-based testing by men in rural Zambia. Factors associated with HIV-testing and acceptance of an offer of home-based testing by men in rural Zambia 2015;19:492-504.22.Lee VJ, Tan SC, Earnest A, et al. User acceptability and feasibility of self-testing with HIV rapid tests. User acceptability and feasibility of self-testing with HIV rapid tests 2007;45:449-53.23.Terris-Prestholt F, Hanson K, MacPhail C, et al. How much demand for New HIV prevention technologies can we really expect? Results from a discrete choice experiment in South Africa. How much demand for New HIV prevention technologies can we really expect? Results from a discrete choice experiment in South Africa 2013;8:e83193.24.Ostermann J, Njau B, Brown DS, et al. Heterogeneous HIV Testing Preferences in an Urban Setting in Tanzania: Results from a Discrete Choice Experiment. Heterogeneous HIV Testing Preferences in an Urban Setting in Tanzania: Results from a Discrete Choice Experiment 2014;9:e92100.25.Phillips KA, Maddala T, and Johnson FR. Measuring preferences for health care interventions using conjoint analysis: an application to HIV testing. Measuring preferences for health care interventions using conjoint analysis: an application to HIV testing 2002;37:1681-1705.26.Ryan M. Discrete choice experiments in health care. BMJ 2004;328:360-361.27.Terris-Prestholt F, Quaiffe M, and Vickerman P. Parameterising user uptake in economic evaluations: the role of discrete choice experiments. Parameterising user uptake in economic evaluations: the role of discrete choice experiments In press.28.Philip P, Hickson F, Bonell C, et al. Men who have sex with men in Britain: comparison of estimates from a probability sample and community-based surveys. Men who have sex with men in Britain: comparison of estimates from a probability sample and community-based surveys In submission.29.Orme B. Sample size issues for conjoint analysis, in Getting started with conjoint analysis: strategies for product design and pricing research. 2010, Research Publishers LLC: Madison, Wisconson.)

[10. Chadborn TR, Delpech VC, Sabin CA, et al. The late diagnosis and consequent short-term mortality of HIV-infected heterosexuals (England and Wales, 2000–2004)](1.Public Health England. HIV in the United Kingdom: 2014 report. 2014.2.Public Health England. HIV new diagnosis, treatment and care: 2015 report.3.Williamson LM, Dodds JP, Mercey DE, et al. Sexual risk behaviour and knowledge of HIV status among community samples of gay men in the UK. Sexual risk behaviour and knowledge of HIV status among community samples of gay men in the UK 2008;22:1063-70.4.Holt M, Rawstorne P, Wilkinson J, et al. HIV testing, gay community involvement and internet use: social and behavioural correlates of HIV testing among Australian men who have sex with men. HIV testing, gay community involvement and internet use: social and behavioural correlates of HIV testing among Australian men who have sex with men 2012;16:13-22.5.Zablotska I, Holt M, de Wit J, et al. Gay men who are not getting tested for HIV. Gay men who are not getting tested for HIV 2012;16:1887-94.6.Witzel TC, Melendez-Torres GJ, Hickson F, et al. HIV testing history and preferences for future tests among gay men, bisexual men and other MSM in England. HIV testing history and preferences for future tests among gay men, bisexual men and other MSM in England In submission.7.UNSW Australia. 2015. Annual report of trends in behaviour 2015. HIV/AIDS, hepatitis and sexually transmissible infections in Australia [Online]. Available: https://csrh.arts.unsw.edu.au/media/CSRHFile/CSRH_Annual_Report_of_Trends_in_Behaviour_2015.pdf.8.Phillips AN, Cambiano V, Miners A, et al. Potential impact on HIV incidence of higher HIV testing rates and earlier antiretroviral therapy initiation in MSM. Potential impact on HIV incidence of higher HIV testing rates and earlier antiretroviral therapy initiation in MSM 2015;29:1855-62.9.Nakagawa F, Lodwick RK, Smith CJ, et al. Projected life expectancy of people with HIV according to timing of diagnosis. Projected life expectancy of people with HIV according to timing of diagnosis 2012;26:335-43.10.Chadborn TR, Delpech VC, Sabin CA, et al. The late diagnosis and consequent short-term mortality of HIV-infected heterosexuals (England and Wales, 2000–2004). The late diagnosis and consequent short-term mortality of HIV-infected heterosexuals (England and Wales, 2000–2004) 2006;20:2371-2379.11.Miners A, Phillips A, Kreif N, et al. Health-related quality-of-life of people with HIV in the era of combination antiretroviral treatment: a cross-sectional comparison with the general population. Health-related quality-of-life of people with HIV in the era of combination antiretroviral treatment: a cross-sectional comparison with the general population 2014;1:e32–e40.12.Cohen MS, Chen YQ, McCauley M, et al. Prevention of HIV-1 Infection with Early Antiretroviral Therapy. Prevention of HIV-1 Infection with Early Antiretroviral Therapy 2011;365:493-505.13.Marks G, Crepaz N, and Janssen RS. Estimating sexual transmission of HIV from persons aware and unaware that they are infected with the virus in the USA. Estimating sexual transmission of HIV from persons aware and unaware that they are infected with the virus in the USA 2006;20:1447-50.14.Nakagawa F, Miners A, Smith CJ, et al. Projected Lifetime Healthcare Costs Associated with HIV Infection. Projected Lifetime Healthcare Costs Associated with HIV Infection 2015;10:e0125018.15.Fleishman JA, Yehia BR, Moore RD, et al. The Economic Burden of Late Entry Into Medical Care for Patients With HIV Infection. The Economic Burden of Late Entry Into Medical Care for Patients With HIV Infection 2010;48:1071-1079.16.Krentz HB and Gill MJ. The Direct Medical Costs of Late Presentation (<350/mm(3)) of HIV Infection over a 15-Year Period. The Direct Medical Costs of Late Presentation (<350/mm(3)) of HIV Infection over a 15-Year Period 2012;2012:757135.17.Krause J, Subklew-Sehume F, Kenyon C, et al. Acceptability of HIV self-testing: a systematic literature review. Acceptability of HIV self-testing: a systematic literature review 2013;13:735.18.Frye V, Wilton L, Hirshfied S, et al. \"Just Because It's Out There, People Aren't Going to Use It.\" HIV Self-Testing Among Young, Black MSM, and Transgender Women. \"Just Because It's Out There, People Aren't Going to Use It.\" HIV Self-Testing Among Young, Black MSM, and Transgender Women 2015.19.Figueroa C, Johnson C, Verster A, et al. Attitudes and Acceptability on HIV Self-testing Among Key Populations: A Literature Review. Attitudes and Acceptability on HIV Self-testing Among Key Populations: A Literature Review 2015.20.Bavinton BR, Brown G, Hurley M, et al. Which gay men would increase their frequency of HIV testing with home self-testing? Which gay men would increase their frequency of HIV testing with home self-testing? 2013;17:2084-92.21.Hensen B, Lewis JJ, Schaap A, et al. Factors associated with HIV-testing and acceptance of an offer of home-based testing by men in rural Zambia. Factors associated with HIV-testing and acceptance of an offer of home-based testing by men in rural Zambia 2015;19:492-504.22.Lee VJ, Tan SC, Earnest A, et al. User acceptability and feasibility of self-testing with HIV rapid tests. User acceptability and feasibility of self-testing with HIV rapid tests 2007;45:449-53.23.Terris-Prestholt F, Hanson K, MacPhail C, et al. How much demand for New HIV prevention technologies can we really expect? Results from a discrete choice experiment in South Africa. How much demand for New HIV prevention technologies can we really expect? Results from a discrete choice experiment in South Africa 2013;8:e83193.24.Ostermann J, Njau B, Brown DS, et al. Heterogeneous HIV Testing Preferences in an Urban Setting in Tanzania: Results from a Discrete Choice Experiment. Heterogeneous HIV Testing Preferences in an Urban Setting in Tanzania: Results from a Discrete Choice Experiment 2014;9:e92100.25.Phillips KA, Maddala T, and Johnson FR. Measuring preferences for health care interventions using conjoint analysis: an application to HIV testing. Measuring preferences for health care interventions using conjoint analysis: an application to HIV testing 2002;37:1681-1705.26.Ryan M. Discrete choice experiments in health care. BMJ 2004;328:360-361.27.Terris-Prestholt F, Quaiffe M, and Vickerman P. Parameterising user uptake in economic evaluations: the role of discrete choice experiments. Parameterising user uptake in economic evaluations: the role of discrete choice experiments In press.28.Philip P, Hickson F, Bonell C, et al. Men who have sex with men in Britain: comparison of estimates from a probability sample and community-based surveys. Men who have sex with men in Britain: comparison of estimates from a probability sample and community-based surveys In submission.29.Orme B. Sample size issues for conjoint analysis, in Getting started with conjoint analysis: strategies for product design and pricing research. 2010, Research Publishers LLC: Madison, Wisconson.)*[.](1.Public Health England. HIV in the United Kingdom: 2014 report. 2014.2.Public Health England. HIV new diagnosis, treatment and care: 2015 report.3.Williamson LM, Dodds JP, Mercey DE, et al. Sexual risk behaviour and knowledge of HIV status among community samples of gay men in the UK. Sexual risk behaviour and knowledge of HIV status among community samples of gay men in the UK 2008;22:1063-70.4.Holt M, Rawstorne P, Wilkinson J, et al. HIV testing, gay community involvement and internet use: social and behavioural correlates of HIV testing among Australian men who have sex with men. HIV testing, gay community involvement and internet use: social and behavioural correlates of HIV testing among Australian men who have sex with men 2012;16:13-22.5.Zablotska I, Holt M, de Wit J, et al. Gay men who are not getting tested for HIV. Gay men who are not getting tested for HIV 2012;16:1887-94.6.Witzel TC, Melendez-Torres GJ, Hickson F, et al. HIV testing history and preferences for future tests among gay men, bisexual men and other MSM in England. HIV testing history and preferences for future tests among gay men, bisexual men and other MSM in England In submission.7.UNSW Australia. 2015. Annual report of trends in behaviour 2015. HIV/AIDS, hepatitis and sexually transmissible infections in Australia [Online]. Available: https://csrh.arts.unsw.edu.au/media/CSRHFile/CSRH_Annual_Report_of_Trends_in_Behaviour_2015.pdf.8.Phillips AN, Cambiano V, Miners A, et al. Potential impact on HIV incidence of higher HIV testing rates and earlier antiretroviral therapy initiation in MSM. Potential impact on HIV incidence of higher HIV testing rates and earlier antiretroviral therapy initiation in MSM 2015;29:1855-62.9.Nakagawa F, Lodwick RK, Smith CJ, et al. Projected life expectancy of people with HIV according to timing of diagnosis. Projected life expectancy of people with HIV according to timing of diagnosis 2012;26:335-43.10.Chadborn TR, Delpech VC, Sabin CA, et al. The late diagnosis and consequent short-term mortality of HIV-infected heterosexuals (England and Wales, 2000–2004). The late diagnosis and consequent short-term mortality of HIV-infected heterosexuals (England and Wales, 2000–2004) 2006;20:2371-2379.11.Miners A, Phillips A, Kreif N, et al. Health-related quality-of-life of people with HIV in the era of combination antiretroviral treatment: a cross-sectional comparison with the general population. Health-related quality-of-life of people with HIV in the era of combination antiretroviral treatment: a cross-sectional comparison with the general population 2014;1:e32–e40.12.Cohen MS, Chen YQ, McCauley M, et al. Prevention of HIV-1 Infection with Early Antiretroviral Therapy. Prevention of HIV-1 Infection with Early Antiretroviral Therapy 2011;365:493-505.13.Marks G, Crepaz N, and Janssen RS. Estimating sexual transmission of HIV from persons aware and unaware that they are infected with the virus in the USA. Estimating sexual transmission of HIV from persons aware and unaware that they are infected with the virus in the USA 2006;20:1447-50.14.Nakagawa F, Miners A, Smith CJ, et al. Projected Lifetime Healthcare Costs Associated with HIV Infection. Projected Lifetime Healthcare Costs Associated with HIV Infection 2015;10:e0125018.15.Fleishman JA, Yehia BR, Moore RD, et al. The Economic Burden of Late Entry Into Medical Care for Patients With HIV Infection. The Economic Burden of Late Entry Into Medical Care for Patients With HIV Infection 2010;48:1071-1079.16.Krentz HB and Gill MJ. The Direct Medical Costs of Late Presentation (<350/mm(3)) of HIV Infection over a 15-Year Period. The Direct Medical Costs of Late Presentation (<350/mm(3)) of HIV Infection over a 15-Year Period 2012;2012:757135.17.Krause J, Subklew-Sehume F, Kenyon C, et al. Acceptability of HIV self-testing: a systematic literature review. Acceptability of HIV self-testing: a systematic literature review 2013;13:735.18.Frye V, Wilton L, Hirshfied S, et al. \"Just Because It's Out There, People Aren't Going to Use It.\" HIV Self-Testing Among Young, Black MSM, and Transgender Women. \"Just Because It's Out There, People Aren't Going to Use It.\" HIV Self-Testing Among Young, Black MSM, and Transgender Women 2015.19.Figueroa C, Johnson C, Verster A, et al. Attitudes and Acceptability on HIV Self-testing Among Key Populations: A Literature Review. Attitudes and Acceptability on HIV Self-testing Among Key Populations: A Literature Review 2015.20.Bavinton BR, Brown G, Hurley M, et al. Which gay men would increase their frequency of HIV testing with home self-testing? Which gay men would increase their frequency of HIV testing with home self-testing? 2013;17:2084-92.21.Hensen B, Lewis JJ, Schaap A, et al. Factors associated with HIV-testing and acceptance of an offer of home-based testing by men in rural Zambia. Factors associated with HIV-testing and acceptance of an offer of home-based testing by men in rural Zambia 2015;19:492-504.22.Lee VJ, Tan SC, Earnest A, et al. User acceptability and feasibility of self-testing with HIV rapid tests. User acceptability and feasibility of self-testing with HIV rapid tests 2007;45:449-53.23.Terris-Prestholt F, Hanson K, MacPhail C, et al. How much demand for New HIV prevention technologies can we really expect? Results from a discrete choice experiment in South Africa. How much demand for New HIV prevention technologies can we really expect? Results from a discrete choice experiment in South Africa 2013;8:e83193.24.Ostermann J, Njau B, Brown DS, et al. Heterogeneous HIV Testing Preferences in an Urban Setting in Tanzania: Results from a Discrete Choice Experiment. Heterogeneous HIV Testing Preferences in an Urban Setting in Tanzania: Results from a Discrete Choice Experiment 2014;9:e92100.25.Phillips KA, Maddala T, and Johnson FR. Measuring preferences for health care interventions using conjoint analysis: an application to HIV testing. Measuring preferences for health care interventions using conjoint analysis: an application to HIV testing 2002;37:1681-1705.26.Ryan M. Discrete choice experiments in health care. BMJ 2004;328:360-361.27.Terris-Prestholt F, Quaiffe M, and Vickerman P. Parameterising user uptake in economic evaluations: the role of discrete choice experiments. Parameterising user uptake in economic evaluations: the role of discrete choice experiments In press.28.Philip P, Hickson F, Bonell C, et al. Men who have sex with men in Britain: comparison of estimates from a probability sample and community-based surveys. Men who have sex with men in Britain: comparison of estimates from a probability sample and community-based surveys In submission.29.Orme B. Sample size issues for conjoint analysis, in Getting started with conjoint analysis: strategies for product design and pricing research. 2010, Research Publishers LLC: Madison, Wisconson.)**[The late diagnosis and consequent short-term mortality of HIV-infected heterosexuals (England and Wales, 2000–2004)](1.Public Health England. HIV in the United Kingdom: 2014 report. 2014.2.Public Health England. HIV new diagnosis, treatment and care: 2015 report.3.Williamson LM, Dodds JP, Mercey DE, et al. Sexual risk behaviour and knowledge of HIV status among community samples of gay men in the UK. Sexual risk behaviour and knowledge of HIV status among community samples of gay men in the UK 2008;22:1063-70.4.Holt M, Rawstorne P, Wilkinson J, et al. HIV testing, gay community involvement and internet use: social and behavioural correlates of HIV testing among Australian men who have sex with men. HIV testing, gay community involvement and internet use: social and behavioural correlates of HIV testing among Australian men who have sex with men 2012;16:13-22.5.Zablotska I, Holt M, de Wit J, et al. Gay men who are not getting tested for HIV. Gay men who are not getting tested for HIV 2012;16:1887-94.6.Witzel TC, Melendez-Torres GJ, Hickson F, et al. HIV testing history and preferences for future tests among gay men, bisexual men and other MSM in England. HIV testing history and preferences for future tests among gay men, bisexual men and other MSM in England In submission.7.UNSW Australia. 2015. Annual report of trends in behaviour 2015. HIV/AIDS, hepatitis and sexually transmissible infections in Australia [Online]. Available: https://csrh.arts.unsw.edu.au/media/CSRHFile/CSRH_Annual_Report_of_Trends_in_Behaviour_2015.pdf.8.Phillips AN, Cambiano V, Miners A, et al. Potential impact on HIV incidence of higher HIV testing rates and earlier antiretroviral therapy initiation in MSM. Potential impact on HIV incidence of higher HIV testing rates and earlier antiretroviral therapy initiation in MSM 2015;29:1855-62.9.Nakagawa F, Lodwick RK, Smith CJ, et al. Projected life expectancy of people with HIV according to timing of diagnosis. Projected life expectancy of people with HIV according to timing of diagnosis 2012;26:335-43.10.Chadborn TR, Delpech VC, Sabin CA, et al. The late diagnosis and consequent short-term mortality of HIV-infected heterosexuals (England and Wales, 2000–2004). The late diagnosis and consequent short-term mortality of HIV-infected heterosexuals (England and Wales, 2000–2004) 2006;20:2371-2379.11.Miners A, Phillips A, Kreif N, et al. Health-related quality-of-life of people with HIV in the era of combination antiretroviral treatment: a cross-sectional comparison with the general population. Health-related quality-of-life of people with HIV in the era of combination antiretroviral treatment: a cross-sectional comparison with the general population 2014;1:e32–e40.12.Cohen MS, Chen YQ, McCauley M, et al. Prevention of HIV-1 Infection with Early Antiretroviral Therapy. Prevention of HIV-1 Infection with Early Antiretroviral Therapy 2011;365:493-505.13.Marks G, Crepaz N, and Janssen RS. Estimating sexual transmission of HIV from persons aware and unaware that they are infected with the virus in the USA. Estimating sexual transmission of HIV from persons aware and unaware that they are infected with the virus in the USA 2006;20:1447-50.14.Nakagawa F, Miners A, Smith CJ, et al. Projected Lifetime Healthcare Costs Associated with HIV Infection. Projected Lifetime Healthcare Costs Associated with HIV Infection 2015;10:e0125018.15.Fleishman JA, Yehia BR, Moore RD, et al. The Economic Burden of Late Entry Into Medical Care for Patients With HIV Infection. The Economic Burden of Late Entry Into Medical Care for Patients With HIV Infection 2010;48:1071-1079.16.Krentz HB and Gill MJ. The Direct Medical Costs of Late Presentation (<350/mm(3)) of HIV Infection over a 15-Year Period. The Direct Medical Costs of Late Presentation (<350/mm(3)) of HIV Infection over a 15-Year Period 2012;2012:757135.17.Krause J, Subklew-Sehume F, Kenyon C, et al. Acceptability of HIV self-testing: a systematic literature review. Acceptability of HIV self-testing: a systematic literature review 2013;13:735.18.Frye V, Wilton L, Hirshfied S, et al. \"Just Because It's Out There, People Aren't Going to Use It.\" HIV Self-Testing Among Young, Black MSM, and Transgender Women. \"Just Because It's Out There, People Aren't Going to Use It.\" HIV Self-Testing Among Young, Black MSM, and Transgender Women 2015.19.Figueroa C, Johnson C, Verster A, et al. Attitudes and Acceptability on HIV Self-testing Among Key Populations: A Literature Review. Attitudes and Acceptability on HIV Self-testing Among Key Populations: A Literature Review 2015.20.Bavinton BR, Brown G, Hurley M, et al. Which gay men would increase their frequency of HIV testing with home self-testing? Which gay men would increase their frequency of HIV testing with home self-testing? 2013;17:2084-92.21.Hensen B, Lewis JJ, Schaap A, et al. Factors associated with HIV-testing and acceptance of an offer of home-based testing by men in rural Zambia. Factors associated with HIV-testing and acceptance of an offer of home-based testing by men in rural Zambia 2015;19:492-504.22.Lee VJ, Tan SC, Earnest A, et al. User acceptability and feasibility of self-testing with HIV rapid tests. User acceptability and feasibility of self-testing with HIV rapid tests 2007;45:449-53.23.Terris-Prestholt F, Hanson K, MacPhail C, et al. How much demand for New HIV prevention technologies can we really expect? Results from a discrete choice experiment in South Africa. How much demand for New HIV prevention technologies can we really expect? Results from a discrete choice experiment in South Africa 2013;8:e83193.24.Ostermann J, Njau B, Brown DS, et al. Heterogeneous HIV Testing Preferences in an Urban Setting in Tanzania: Results from a Discrete Choice Experiment. Heterogeneous HIV Testing Preferences in an Urban Setting in Tanzania: Results from a Discrete Choice Experiment 2014;9:e92100.25.Phillips KA, Maddala T, and Johnson FR. Measuring preferences for health care interventions using conjoint analysis: an application to HIV testing. Measuring preferences for health care interventions using conjoint analysis: an application to HIV testing 2002;37:1681-1705.26.Ryan M. Discrete choice experiments in health care. BMJ 2004;328:360-361.27.Terris-Prestholt F, Quaiffe M, and Vickerman P. Parameterising user uptake in economic evaluations: the role of discrete choice experiments. Parameterising user uptake in economic evaluations: the role of discrete choice experiments In press.28.Philip P, Hickson F, Bonell C, et al. Men who have sex with men in Britain: comparison of estimates from a probability sample and community-based surveys. Men who have sex with men in Britain: comparison of estimates from a probability sample and community-based surveys In submission.29.Orme B. Sample size issues for conjoint analysis, in Getting started with conjoint analysis: strategies for product design and pricing research. 2010, Research Publishers LLC: Madison, Wisconson.)* [2006;](1.Public Health England. HIV in the United Kingdom: 2014 report. 2014.2.Public Health England. HIV new diagnosis, treatment and care: 2015 report.3.Williamson LM, Dodds JP, Mercey DE, et al. Sexual risk behaviour and knowledge of HIV status among community samples of gay men in the UK. Sexual risk behaviour and knowledge of HIV status among community samples of gay men in the UK 2008;22:1063-70.4.Holt M, Rawstorne P, Wilkinson J, et al. HIV testing, gay community involvement and internet use: social and behavioural correlates of HIV testing among Australian men who have sex with men. HIV testing, gay community involvement and internet use: social and behavioural correlates of HIV testing among Australian men who have sex with men 2012;16:13-22.5.Zablotska I, Holt M, de Wit J, et al. Gay men who are not getting tested for HIV. Gay men who are not getting tested for HIV 2012;16:1887-94.6.Witzel TC, Melendez-Torres GJ, Hickson F, et al. HIV testing history and preferences for future tests among gay men, bisexual men and other MSM in England. HIV testing history and preferences for future tests among gay men, bisexual men and other MSM in England In submission.7.UNSW Australia. 2015. Annual report of trends in behaviour 2015. HIV/AIDS, hepatitis and sexually transmissible infections in Australia [Online]. Available: https://csrh.arts.unsw.edu.au/media/CSRHFile/CSRH_Annual_Report_of_Trends_in_Behaviour_2015.pdf.8.Phillips AN, Cambiano V, Miners A, et al. Potential impact on HIV incidence of higher HIV testing rates and earlier antiretroviral therapy initiation in MSM. Potential impact on HIV incidence of higher HIV testing rates and earlier antiretroviral therapy initiation in MSM 2015;29:1855-62.9.Nakagawa F, Lodwick RK, Smith CJ, et al. Projected life expectancy of people with HIV according to timing of diagnosis. Projected life expectancy of people with HIV according to timing of diagnosis 2012;26:335-43.10.Chadborn TR, Delpech VC, Sabin CA, et al. The late diagnosis and consequent short-term mortality of HIV-infected heterosexuals (England and Wales, 2000–2004). The late diagnosis and consequent short-term mortality of HIV-infected heterosexuals (England and Wales, 2000–2004) 2006;20:2371-2379.11.Miners A, Phillips A, Kreif N, et al. Health-related quality-of-life of people with HIV in the era of combination antiretroviral treatment: a cross-sectional comparison with the general population. Health-related quality-of-life of people with HIV in the era of combination antiretroviral treatment: a cross-sectional comparison with the general population 2014;1:e32–e40.12.Cohen MS, Chen YQ, McCauley M, et al. Prevention of HIV-1 Infection with Early Antiretroviral Therapy. Prevention of HIV-1 Infection with Early Antiretroviral Therapy 2011;365:493-505.13.Marks G, Crepaz N, and Janssen RS. Estimating sexual transmission of HIV from persons aware and unaware that they are infected with the virus in the USA. Estimating sexual transmission of HIV from persons aware and unaware that they are infected with the virus in the USA 2006;20:1447-50.14.Nakagawa F, Miners A, Smith CJ, et al. Projected Lifetime Healthcare Costs Associated with HIV Infection. Projected Lifetime Healthcare Costs Associated with HIV Infection 2015;10:e0125018.15.Fleishman JA, Yehia BR, Moore RD, et al. The Economic Burden of Late Entry Into Medical Care for Patients With HIV Infection. The Economic Burden of Late Entry Into Medical Care for Patients With HIV Infection 2010;48:1071-1079.16.Krentz HB and Gill MJ. The Direct Medical Costs of Late Presentation (<350/mm(3)) of HIV Infection over a 15-Year Period. The Direct Medical Costs of Late Presentation (<350/mm(3)) of HIV Infection over a 15-Year Period 2012;2012:757135.17.Krause J, Subklew-Sehume F, Kenyon C, et al. Acceptability of HIV self-testing: a systematic literature review. Acceptability of HIV self-testing: a systematic literature review 2013;13:735.18.Frye V, Wilton L, Hirshfied S, et al. \"Just Because It's Out There, People Aren't Going to Use It.\" HIV Self-Testing Among Young, Black MSM, and Transgender Women. \"Just Because It's Out There, People Aren't Going to Use It.\" HIV Self-Testing Among Young, Black MSM, and Transgender Women 2015.19.Figueroa C, Johnson C, Verster A, et al. Attitudes and Acceptability on HIV Self-testing Among Key Populations: A Literature Review. Attitudes and Acceptability on HIV Self-testing Among Key Populations: A Literature Review 2015.20.Bavinton BR, Brown G, Hurley M, et al. Which gay men would increase their frequency of HIV testing with home self-testing? Which gay men would increase their frequency of HIV testing with home self-testing? 2013;17:2084-92.21.Hensen B, Lewis JJ, Schaap A, et al. Factors associated with HIV-testing and acceptance of an offer of home-based testing by men in rural Zambia. Factors associated with HIV-testing and acceptance of an offer of home-based testing by men in rural Zambia 2015;19:492-504.22.Lee VJ, Tan SC, Earnest A, et al. User acceptability and feasibility of self-testing with HIV rapid tests. User acceptability and feasibility of self-testing with HIV rapid tests 2007;45:449-53.23.Terris-Prestholt F, Hanson K, MacPhail C, et al. How much demand for New HIV prevention technologies can we really expect? Results from a discrete choice experiment in South Africa. How much demand for New HIV prevention technologies can we really expect? Results from a discrete choice experiment in South Africa 2013;8:e83193.24.Ostermann J, Njau B, Brown DS, et al. Heterogeneous HIV Testing Preferences in an Urban Setting in Tanzania: Results from a Discrete Choice Experiment. Heterogeneous HIV Testing Preferences in an Urban Setting in Tanzania: Results from a Discrete Choice Experiment 2014;9:e92100.25.Phillips KA, Maddala T, and Johnson FR. Measuring preferences for health care interventions using conjoint analysis: an application to HIV testing. Measuring preferences for health care interventions using conjoint analysis: an application to HIV testing 2002;37:1681-1705.26.Ryan M. Discrete choice experiments in health care. BMJ 2004;328:360-361.27.Terris-Prestholt F, Quaiffe M, and Vickerman P. Parameterising user uptake in economic evaluations: the role of discrete choice experiments. Parameterising user uptake in economic evaluations: the role of discrete choice experiments In press.28.Philip P, Hickson F, Bonell C, et al. Men who have sex with men in Britain: comparison of estimates from a probability sample and community-based surveys. Men who have sex with men in Britain: comparison of estimates from a probability sample and community-based surveys In submission.29.Orme B. Sample size issues for conjoint analysis, in Getting started with conjoint analysis: strategies for product design and pricing research. 2010, Research Publishers LLC: Madison, Wisconson.)**[20](1.Public Health England. HIV in the United Kingdom: 2014 report. 2014.2.Public Health England. HIV new diagnosis, treatment and care: 2015 report.3.Williamson LM, Dodds JP, Mercey DE, et al. Sexual risk behaviour and knowledge of HIV status among community samples of gay men in the UK. Sexual risk behaviour and knowledge of HIV status among community samples of gay men in the UK 2008;22:1063-70.4.Holt M, Rawstorne P, Wilkinson J, et al. HIV testing, gay community involvement and internet use: social and behavioural correlates of HIV testing among Australian men who have sex with men. HIV testing, gay community involvement and internet use: social and behavioural correlates of HIV testing among Australian men who have sex with men 2012;16:13-22.5.Zablotska I, Holt M, de Wit J, et al. Gay men who are not getting tested for HIV. Gay men who are not getting tested for HIV 2012;16:1887-94.6.Witzel TC, Melendez-Torres GJ, Hickson F, et al. HIV testing history and preferences for future tests among gay men, bisexual men and other MSM in England. HIV testing history and preferences for future tests among gay men, bisexual men and other MSM in England In submission.7.UNSW Australia. 2015. Annual report of trends in behaviour 2015. HIV/AIDS, hepatitis and sexually transmissible infections in Australia [Online]. Available: https://csrh.arts.unsw.edu.au/media/CSRHFile/CSRH_Annual_Report_of_Trends_in_Behaviour_2015.pdf.8.Phillips AN, Cambiano V, Miners A, et al. Potential impact on HIV incidence of higher HIV testing rates and earlier antiretroviral therapy initiation in MSM. Potential impact on HIV incidence of higher HIV testing rates and earlier antiretroviral therapy initiation in MSM 2015;29:1855-62.9.Nakagawa F, Lodwick RK, Smith CJ, et al. Projected life expectancy of people with HIV according to timing of diagnosis. Projected life expectancy of people with HIV according to timing of diagnosis 2012;26:335-43.10.Chadborn TR, Delpech VC, Sabin CA, et al. The late diagnosis and consequent short-term mortality of HIV-infected heterosexuals (England and Wales, 2000–2004). The late diagnosis and consequent short-term mortality of HIV-infected heterosexuals (England and Wales, 2000–2004) 2006;20:2371-2379.11.Miners A, Phillips A, Kreif N, et al. Health-related quality-of-life of people with HIV in the era of combination antiretroviral treatment: a cross-sectional comparison with the general population. Health-related quality-of-life of people with HIV in the era of combination antiretroviral treatment: a cross-sectional comparison with the general population 2014;1:e32–e40.12.Cohen MS, Chen YQ, McCauley M, et al. Prevention of HIV-1 Infection with Early Antiretroviral Therapy. Prevention of HIV-1 Infection with Early Antiretroviral Therapy 2011;365:493-505.13.Marks G, Crepaz N, and Janssen RS. Estimating sexual transmission of HIV from persons aware and unaware that they are infected with the virus in the USA. Estimating sexual transmission of HIV from persons aware and unaware that they are infected with the virus in the USA 2006;20:1447-50.14.Nakagawa F, Miners A, Smith CJ, et al. Projected Lifetime Healthcare Costs Associated with HIV Infection. Projected Lifetime Healthcare Costs Associated with HIV Infection 2015;10:e0125018.15.Fleishman JA, Yehia BR, Moore RD, et al. The Economic Burden of Late Entry Into Medical Care for Patients With HIV Infection. The Economic Burden of Late Entry Into Medical Care for Patients With HIV Infection 2010;48:1071-1079.16.Krentz HB and Gill MJ. The Direct Medical Costs of Late Presentation (<350/mm(3)) of HIV Infection over a 15-Year Period. The Direct Medical Costs of Late Presentation (<350/mm(3)) of HIV Infection over a 15-Year Period 2012;2012:757135.17.Krause J, Subklew-Sehume F, Kenyon C, et al. Acceptability of HIV self-testing: a systematic literature review. Acceptability of HIV self-testing: a systematic literature review 2013;13:735.18.Frye V, Wilton L, Hirshfied S, et al. \"Just Because It's Out There, People Aren't Going to Use It.\" HIV Self-Testing Among Young, Black MSM, and Transgender Women. \"Just Because It's Out There, People Aren't Going to Use It.\" HIV Self-Testing Among Young, Black MSM, and Transgender Women 2015.19.Figueroa C, Johnson C, Verster A, et al. Attitudes and Acceptability on HIV Self-testing Among Key Populations: A Literature Review. Attitudes and Acceptability on HIV Self-testing Among Key Populations: A Literature Review 2015.20.Bavinton BR, Brown G, Hurley M, et al. Which gay men would increase their frequency of HIV testing with home self-testing? Which gay men would increase their frequency of HIV testing with home self-testing? 2013;17:2084-92.21.Hensen B, Lewis JJ, Schaap A, et al. Factors associated with HIV-testing and acceptance of an offer of home-based testing by men in rural Zambia. Factors associated with HIV-testing and acceptance of an offer of home-based testing by men in rural Zambia 2015;19:492-504.22.Lee VJ, Tan SC, Earnest A, et al. User acceptability and feasibility of self-testing with HIV rapid tests. User acceptability and feasibility of self-testing with HIV rapid tests 2007;45:449-53.23.Terris-Prestholt F, Hanson K, MacPhail C, et al. How much demand for New HIV prevention technologies can we really expect? Results from a discrete choice experiment in South Africa. How much demand for New HIV prevention technologies can we really expect? Results from a discrete choice experiment in South Africa 2013;8:e83193.24.Ostermann J, Njau B, Brown DS, et al. Heterogeneous HIV Testing Preferences in an Urban Setting in Tanzania: Results from a Discrete Choice Experiment. Heterogeneous HIV Testing Preferences in an Urban Setting in Tanzania: Results from a Discrete Choice Experiment 2014;9:e92100.25.Phillips KA, Maddala T, and Johnson FR. Measuring preferences for health care interventions using conjoint analysis: an application to HIV testing. Measuring preferences for health care interventions using conjoint analysis: an application to HIV testing 2002;37:1681-1705.26.Ryan M. Discrete choice experiments in health care. BMJ 2004;328:360-361.27.Terris-Prestholt F, Quaiffe M, and Vickerman P. Parameterising user uptake in economic evaluations: the role of discrete choice experiments. Parameterising user uptake in economic evaluations: the role of discrete choice experiments In press.28.Philip P, Hickson F, Bonell C, et al. Men who have sex with men in Britain: comparison of estimates from a probability sample and community-based surveys. Men who have sex with men in Britain: comparison of estimates from a probability sample and community-based surveys In submission.29.Orme B. Sample size issues for conjoint analysis, in Getting started with conjoint analysis: strategies for product design and pricing research. 2010, Research Publishers LLC: Madison, Wisconson.)**[:2371-2379.](1.Public Health England. HIV in the United Kingdom: 2014 report. 2014.2.Public Health England. HIV new diagnosis, treatment and care: 2015 report.3.Williamson LM, Dodds JP, Mercey DE, et al. Sexual risk behaviour and knowledge of HIV status among community samples of gay men in the UK. Sexual risk behaviour and knowledge of HIV status among community samples of gay men in the UK 2008;22:1063-70.4.Holt M, Rawstorne P, Wilkinson J, et al. HIV testing, gay community involvement and internet use: social and behavioural correlates of HIV testing among Australian men who have sex with men. HIV testing, gay community involvement and internet use: social and behavioural correlates of HIV testing among Australian men who have sex with men 2012;16:13-22.5.Zablotska I, Holt M, de Wit J, et al. Gay men who are not getting tested for HIV. Gay men who are not getting tested for HIV 2012;16:1887-94.6.Witzel TC, Melendez-Torres GJ, Hickson F, et al. HIV testing history and preferences for future tests among gay men, bisexual men and other MSM in England. HIV testing history and preferences for future tests among gay men, bisexual men and other MSM in England In submission.7.UNSW Australia. 2015. Annual report of trends in behaviour 2015. HIV/AIDS, hepatitis and sexually transmissible infections in Australia [Online]. Available: https://csrh.arts.unsw.edu.au/media/CSRHFile/CSRH_Annual_Report_of_Trends_in_Behaviour_2015.pdf.8.Phillips AN, Cambiano V, Miners A, et al. Potential impact on HIV incidence of higher HIV testing rates and earlier antiretroviral therapy initiation in MSM. Potential impact on HIV incidence of higher HIV testing rates and earlier antiretroviral therapy initiation in MSM 2015;29:1855-62.9.Nakagawa F, Lodwick RK, Smith CJ, et al. Projected life expectancy of people with HIV according to timing of diagnosis. Projected life expectancy of people with HIV according to timing of diagnosis 2012;26:335-43.10.Chadborn TR, Delpech VC, Sabin CA, et al. The late diagnosis and consequent short-term mortality of HIV-infected heterosexuals (England and Wales, 2000–2004). The late diagnosis and consequent short-term mortality of HIV-infected heterosexuals (England and Wales, 2000–2004) 2006;20:2371-2379.11.Miners A, Phillips A, Kreif N, et al. Health-related quality-of-life of people with HIV in the era of combination antiretroviral treatment: a cross-sectional comparison with the general population. Health-related quality-of-life of people with HIV in the era of combination antiretroviral treatment: a cross-sectional comparison with the general population 2014;1:e32–e40.12.Cohen MS, Chen YQ, McCauley M, et al. Prevention of HIV-1 Infection with Early Antiretroviral Therapy. Prevention of HIV-1 Infection with Early Antiretroviral Therapy 2011;365:493-505.13.Marks G, Crepaz N, and Janssen RS. Estimating sexual transmission of HIV from persons aware and unaware that they are infected with the virus in the USA. Estimating sexual transmission of HIV from persons aware and unaware that they are infected with the virus in the USA 2006;20:1447-50.14.Nakagawa F, Miners A, Smith CJ, et al. Projected Lifetime Healthcare Costs Associated with HIV Infection. Projected Lifetime Healthcare Costs Associated with HIV Infection 2015;10:e0125018.15.Fleishman JA, Yehia BR, Moore RD, et al. The Economic Burden of Late Entry Into Medical Care for Patients With HIV Infection. The Economic Burden of Late Entry Into Medical Care for Patients With HIV Infection 2010;48:1071-1079.16.Krentz HB and Gill MJ. The Direct Medical Costs of Late Presentation (<350/mm(3)) of HIV Infection over a 15-Year Period. The Direct Medical Costs of Late Presentation (<350/mm(3)) of HIV Infection over a 15-Year Period 2012;2012:757135.17.Krause J, Subklew-Sehume F, Kenyon C, et al. Acceptability of HIV self-testing: a systematic literature review. Acceptability of HIV self-testing: a systematic literature review 2013;13:735.18.Frye V, Wilton L, Hirshfied S, et al. \"Just Because It's Out There, People Aren't Going to Use It.\" HIV Self-Testing Among Young, Black MSM, and Transgender Women. \"Just Because It's Out There, People Aren't Going to Use It.\" HIV Self-Testing Among Young, Black MSM, and Transgender Women 2015.19.Figueroa C, Johnson C, Verster A, et al. Attitudes and Acceptability on HIV Self-testing Among Key Populations: A Literature Review. Attitudes and Acceptability on HIV Self-testing Among Key Populations: A Literature Review 2015.20.Bavinton BR, Brown G, Hurley M, et al. Which gay men would increase their frequency of HIV testing with home self-testing? Which gay men would increase their frequency of HIV testing with home self-testing? 2013;17:2084-92.21.Hensen B, Lewis JJ, Schaap A, et al. Factors associated with HIV-testing and acceptance of an offer of home-based testing by men in rural Zambia. Factors associated with HIV-testing and acceptance of an offer of home-based testing by men in rural Zambia 2015;19:492-504.22.Lee VJ, Tan SC, Earnest A, et al. User acceptability and feasibility of self-testing with HIV rapid tests. User acceptability and feasibility of self-testing with HIV rapid tests 2007;45:449-53.23.Terris-Prestholt F, Hanson K, MacPhail C, et al. How much demand for New HIV prevention technologies can we really expect? Results from a discrete choice experiment in South Africa. How much demand for New HIV prevention technologies can we really expect? Results from a discrete choice experiment in South Africa 2013;8:e83193.24.Ostermann J, Njau B, Brown DS, et al. Heterogeneous HIV Testing Preferences in an Urban Setting in Tanzania: Results from a Discrete Choice Experiment. Heterogeneous HIV Testing Preferences in an Urban Setting in Tanzania: Results from a Discrete Choice Experiment 2014;9:e92100.25.Phillips KA, Maddala T, and Johnson FR. Measuring preferences for health care interventions using conjoint analysis: an application to HIV testing. Measuring preferences for health care interventions using conjoint analysis: an application to HIV testing 2002;37:1681-1705.26.Ryan M. Discrete choice experiments in health care. BMJ 2004;328:360-361.27.Terris-Prestholt F, Quaiffe M, and Vickerman P. Parameterising user uptake in economic evaluations: the role of discrete choice experiments. Parameterising user uptake in economic evaluations: the role of discrete choice experiments In press.28.Philip P, Hickson F, Bonell C, et al. Men who have sex with men in Britain: comparison of estimates from a probability sample and community-based surveys. Men who have sex with men in Britain: comparison of estimates from a probability sample and community-based surveys In submission.29.Orme B. Sample size issues for conjoint analysis, in Getting started with conjoint analysis: strategies for product design and pricing research. 2010, Research Publishers LLC: Madison, Wisconson.)

[11. Miners A, Phillips A, Kreif N, et al. Health-related quality-of-life of people with HIV in the era of combination antiretroviral treatment: a cross-sectional comparison with the general population](1.Public Health England. HIV in the United Kingdom: 2014 report. 2014.2.Public Health England. HIV new diagnosis, treatment and care: 2015 report.3.Williamson LM, Dodds JP, Mercey DE, et al. Sexual risk behaviour and knowledge of HIV status among community samples of gay men in the UK. Sexual risk behaviour and knowledge of HIV status among community samples of gay men in the UK 2008;22:1063-70.4.Holt M, Rawstorne P, Wilkinson J, et al. HIV testing, gay community involvement and internet use: social and behavioural correlates of HIV testing among Australian men who have sex with men. HIV testing, gay community involvement and internet use: social and behavioural correlates of HIV testing among Australian men who have sex with men 2012;16:13-22.5.Zablotska I, Holt M, de Wit J, et al. Gay men who are not getting tested for HIV. Gay men who are not getting tested for HIV 2012;16:1887-94.6.Witzel TC, Melendez-Torres GJ, Hickson F, et al. HIV testing history and preferences for future tests among gay men, bisexual men and other MSM in England. HIV testing history and preferences for future tests among gay men, bisexual men and other MSM in England In submission.7.UNSW Australia. 2015. Annual report of trends in behaviour 2015. HIV/AIDS, hepatitis and sexually transmissible infections in Australia [Online]. Available: https://csrh.arts.unsw.edu.au/media/CSRHFile/CSRH_Annual_Report_of_Trends_in_Behaviour_2015.pdf.8.Phillips AN, Cambiano V, Miners A, et al. Potential impact on HIV incidence of higher HIV testing rates and earlier antiretroviral therapy initiation in MSM. Potential impact on HIV incidence of higher HIV testing rates and earlier antiretroviral therapy initiation in MSM 2015;29:1855-62.9.Nakagawa F, Lodwick RK, Smith CJ, et al. Projected life expectancy of people with HIV according to timing of diagnosis. Projected life expectancy of people with HIV according to timing of diagnosis 2012;26:335-43.10.Chadborn TR, Delpech VC, Sabin CA, et al. The late diagnosis and consequent short-term mortality of HIV-infected heterosexuals (England and Wales, 2000–2004). The late diagnosis and consequent short-term mortality of HIV-infected heterosexuals (England and Wales, 2000–2004) 2006;20:2371-2379.11.Miners A, Phillips A, Kreif N, et al. Health-related quality-of-life of people with HIV in the era of combination antiretroviral treatment: a cross-sectional comparison with the general population. Health-related quality-of-life of people with HIV in the era of combination antiretroviral treatment: a cross-sectional comparison with the general population 2014;1:e32–e40.12.Cohen MS, Chen YQ, McCauley M, et al. Prevention of HIV-1 Infection with Early Antiretroviral Therapy. Prevention of HIV-1 Infection with Early Antiretroviral Therapy 2011;365:493-505.13.Marks G, Crepaz N, and Janssen RS. Estimating sexual transmission of HIV from persons aware and unaware that they are infected with the virus in the USA. Estimating sexual transmission of HIV from persons aware and unaware that they are infected with the virus in the USA 2006;20:1447-50.14.Nakagawa F, Miners A, Smith CJ, et al. Projected Lifetime Healthcare Costs Associated with HIV Infection. Projected Lifetime Healthcare Costs Associated with HIV Infection 2015;10:e0125018.15.Fleishman JA, Yehia BR, Moore RD, et al. The Economic Burden of Late Entry Into Medical Care for Patients With HIV Infection. The Economic Burden of Late Entry Into Medical Care for Patients With HIV Infection 2010;48:1071-1079.16.Krentz HB and Gill MJ. The Direct Medical Costs of Late Presentation (<350/mm(3)) of HIV Infection over a 15-Year Period. The Direct Medical Costs of Late Presentation (<350/mm(3)) of HIV Infection over a 15-Year Period 2012;2012:757135.17.Krause J, Subklew-Sehume F, Kenyon C, et al. Acceptability of HIV self-testing: a systematic literature review. Acceptability of HIV self-testing: a systematic literature review 2013;13:735.18.Frye V, Wilton L, Hirshfied S, et al. \"Just Because It's Out There, People Aren't Going to Use It.\" HIV Self-Testing Among Young, Black MSM, and Transgender Women. \"Just Because It's Out There, People Aren't Going to Use It.\" HIV Self-Testing Among Young, Black MSM, and Transgender Women 2015.19.Figueroa C, Johnson C, Verster A, et al. Attitudes and Acceptability on HIV Self-testing Among Key Populations: A Literature Review. Attitudes and Acceptability on HIV Self-testing Among Key Populations: A Literature Review 2015.20.Bavinton BR, Brown G, Hurley M, et al. Which gay men would increase their frequency of HIV testing with home self-testing? Which gay men would increase their frequency of HIV testing with home self-testing? 2013;17:2084-92.21.Hensen B, Lewis JJ, Schaap A, et al. Factors associated with HIV-testing and acceptance of an offer of home-based testing by men in rural Zambia. Factors associated with HIV-testing and acceptance of an offer of home-based testing by men in rural Zambia 2015;19:492-504.22.Lee VJ, Tan SC, Earnest A, et al. User acceptability and feasibility of self-testing with HIV rapid tests. User acceptability and feasibility of self-testing with HIV rapid tests 2007;45:449-53.23.Terris-Prestholt F, Hanson K, MacPhail C, et al. How much demand for New HIV prevention technologies can we really expect? Results from a discrete choice experiment in South Africa. How much demand for New HIV prevention technologies can we really expect? Results from a discrete choice experiment in South Africa 2013;8:e83193.24.Ostermann J, Njau B, Brown DS, et al. Heterogeneous HIV Testing Preferences in an Urban Setting in Tanzania: Results from a Discrete Choice Experiment. Heterogeneous HIV Testing Preferences in an Urban Setting in Tanzania: Results from a Discrete Choice Experiment 2014;9:e92100.25.Phillips KA, Maddala T, and Johnson FR. Measuring preferences for health care interventions using conjoint analysis: an application to HIV testing. Measuring preferences for health care interventions using conjoint analysis: an application to HIV testing 2002;37:1681-1705.26.Ryan M. Discrete choice experiments in health care. BMJ 2004;328:360-361.27.Terris-Prestholt F, Quaiffe M, and Vickerman P. Parameterising user uptake in economic evaluations: the role of discrete choice experiments. Parameterising user uptake in economic evaluations: the role of discrete choice experiments In press.28.Philip P, Hickson F, Bonell C, et al. Men who have sex with men in Britain: comparison of estimates from a probability sample and community-based surveys. Men who have sex with men in Britain: comparison of estimates from a probability sample and community-based surveys In submission.29.Orme B. Sample size issues for conjoint analysis, in Getting started with conjoint analysis: strategies for product design and pricing research. 2010, Research Publishers LLC: Madison, Wisconson.)*[.](1.Public Health England. HIV in the United Kingdom: 2014 report. 2014.2.Public Health England. HIV new diagnosis, treatment and care: 2015 report.3.Williamson LM, Dodds JP, Mercey DE, et al. Sexual risk behaviour and knowledge of HIV status among community samples of gay men in the UK. Sexual risk behaviour and knowledge of HIV status among community samples of gay men in the UK 2008;22:1063-70.4.Holt M, Rawstorne P, Wilkinson J, et al. HIV testing, gay community involvement and internet use: social and behavioural correlates of HIV testing among Australian men who have sex with men. HIV testing, gay community involvement and internet use: social and behavioural correlates of HIV testing among Australian men who have sex with men 2012;16:13-22.5.Zablotska I, Holt M, de Wit J, et al. Gay men who are not getting tested for HIV. Gay men who are not getting tested for HIV 2012;16:1887-94.6.Witzel TC, Melendez-Torres GJ, Hickson F, et al. HIV testing history and preferences for future tests among gay men, bisexual men and other MSM in England. HIV testing history and preferences for future tests among gay men, bisexual men and other MSM in England In submission.7.UNSW Australia. 2015. Annual report of trends in behaviour 2015. HIV/AIDS, hepatitis and sexually transmissible infections in Australia [Online]. Available: https://csrh.arts.unsw.edu.au/media/CSRHFile/CSRH_Annual_Report_of_Trends_in_Behaviour_2015.pdf.8.Phillips AN, Cambiano V, Miners A, et al. Potential impact on HIV incidence of higher HIV testing rates and earlier antiretroviral therapy initiation in MSM. Potential impact on HIV incidence of higher HIV testing rates and earlier antiretroviral therapy initiation in MSM 2015;29:1855-62.9.Nakagawa F, Lodwick RK, Smith CJ, et al. Projected life expectancy of people with HIV according to timing of diagnosis. Projected life expectancy of people with HIV according to timing of diagnosis 2012;26:335-43.10.Chadborn TR, Delpech VC, Sabin CA, et al. The late diagnosis and consequent short-term mortality of HIV-infected heterosexuals (England and Wales, 2000–2004). The late diagnosis and consequent short-term mortality of HIV-infected heterosexuals (England and Wales, 2000–2004) 2006;20:2371-2379.11.Miners A, Phillips A, Kreif N, et al. Health-related quality-of-life of people with HIV in the era of combination antiretroviral treatment: a cross-sectional comparison with the general population. Health-related quality-of-life of people with HIV in the era of combination antiretroviral treatment: a cross-sectional comparison with the general population 2014;1:e32–e40.12.Cohen MS, Chen YQ, McCauley M, et al. Prevention of HIV-1 Infection with Early Antiretroviral Therapy. Prevention of HIV-1 Infection with Early Antiretroviral Therapy 2011;365:493-505.13.Marks G, Crepaz N, and Janssen RS. Estimating sexual transmission of HIV from persons aware and unaware that they are infected with the virus in the USA. Estimating sexual transmission of HIV from persons aware and unaware that they are infected with the virus in the USA 2006;20:1447-50.14.Nakagawa F, Miners A, Smith CJ, et al. Projected Lifetime Healthcare Costs Associated with HIV Infection. Projected Lifetime Healthcare Costs Associated with HIV Infection 2015;10:e0125018.15.Fleishman JA, Yehia BR, Moore RD, et al. The Economic Burden of Late Entry Into Medical Care for Patients With HIV Infection. The Economic Burden of Late Entry Into Medical Care for Patients With HIV Infection 2010;48:1071-1079.16.Krentz HB and Gill MJ. The Direct Medical Costs of Late Presentation (<350/mm(3)) of HIV Infection over a 15-Year Period. The Direct Medical Costs of Late Presentation (<350/mm(3)) of HIV Infection over a 15-Year Period 2012;2012:757135.17.Krause J, Subklew-Sehume F, Kenyon C, et al. Acceptability of HIV self-testing: a systematic literature review. Acceptability of HIV self-testing: a systematic literature review 2013;13:735.18.Frye V, Wilton L, Hirshfied S, et al. \"Just Because It's Out There, People Aren't Going to Use It.\" HIV Self-Testing Among Young, Black MSM, and Transgender Women. \"Just Because It's Out There, People Aren't Going to Use It.\" HIV Self-Testing Among Young, Black MSM, and Transgender Women 2015.19.Figueroa C, Johnson C, Verster A, et al. Attitudes and Acceptability on HIV Self-testing Among Key Populations: A Literature Review. Attitudes and Acceptability on HIV Self-testing Among Key Populations: A Literature Review 2015.20.Bavinton BR, Brown G, Hurley M, et al. Which gay men would increase their frequency of HIV testing with home self-testing? Which gay men would increase their frequency of HIV testing with home self-testing? 2013;17:2084-92.21.Hensen B, Lewis JJ, Schaap A, et al. Factors associated with HIV-testing and acceptance of an offer of home-based testing by men in rural Zambia. Factors associated with HIV-testing and acceptance of an offer of home-based testing by men in rural Zambia 2015;19:492-504.22.Lee VJ, Tan SC, Earnest A, et al. User acceptability and feasibility of self-testing with HIV rapid tests. User acceptability and feasibility of self-testing with HIV rapid tests 2007;45:449-53.23.Terris-Prestholt F, Hanson K, MacPhail C, et al. How much demand for New HIV prevention technologies can we really expect? Results from a discrete choice experiment in South Africa. How much demand for New HIV prevention technologies can we really expect? Results from a discrete choice experiment in South Africa 2013;8:e83193.24.Ostermann J, Njau B, Brown DS, et al. Heterogeneous HIV Testing Preferences in an Urban Setting in Tanzania: Results from a Discrete Choice Experiment. Heterogeneous HIV Testing Preferences in an Urban Setting in Tanzania: Results from a Discrete Choice Experiment 2014;9:e92100.25.Phillips KA, Maddala T, and Johnson FR. Measuring preferences for health care interventions using conjoint analysis: an application to HIV testing. Measuring preferences for health care interventions using conjoint analysis: an application to HIV testing 2002;37:1681-1705.26.Ryan M. Discrete choice experiments in health care. BMJ 2004;328:360-361.27.Terris-Prestholt F, Quaiffe M, and Vickerman P. Parameterising user uptake in economic evaluations: the role of discrete choice experiments. Parameterising user uptake in economic evaluations: the role of discrete choice experiments In press.28.Philip P, Hickson F, Bonell C, et al. Men who have sex with men in Britain: comparison of estimates from a probability sample and community-based surveys. Men who have sex with men in Britain: comparison of estimates from a probability sample and community-based surveys In submission.29.Orme B. Sample size issues for conjoint analysis, in Getting started with conjoint analysis: strategies for product design and pricing research. 2010, Research Publishers LLC: Madison, Wisconson.)**[Health-related quality-of-life of people with HIV in the era of combination antiretroviral treatment: a cross-sectional comparison with the general population](1.Public Health England. HIV in the United Kingdom: 2014 report. 2014.2.Public Health England. HIV new diagnosis, treatment and care: 2015 report.3.Williamson LM, Dodds JP, Mercey DE, et al. Sexual risk behaviour and knowledge of HIV status among community samples of gay men in the UK. Sexual risk behaviour and knowledge of HIV status among community samples of gay men in the UK 2008;22:1063-70.4.Holt M, Rawstorne P, Wilkinson J, et al. HIV testing, gay community involvement and internet use: social and behavioural correlates of HIV testing among Australian men who have sex with men. HIV testing, gay community involvement and internet use: social and behavioural correlates of HIV testing among Australian men who have sex with men 2012;16:13-22.5.Zablotska I, Holt M, de Wit J, et al. Gay men who are not getting tested for HIV. Gay men who are not getting tested for HIV 2012;16:1887-94.6.Witzel TC, Melendez-Torres GJ, Hickson F, et al. HIV testing history and preferences for future tests among gay men, bisexual men and other MSM in England. HIV testing history and preferences for future tests among gay men, bisexual men and other MSM in England In submission.7.UNSW Australia. 2015. Annual report of trends in behaviour 2015. HIV/AIDS, hepatitis and sexually transmissible infections in Australia [Online]. Available: https://csrh.arts.unsw.edu.au/media/CSRHFile/CSRH_Annual_Report_of_Trends_in_Behaviour_2015.pdf.8.Phillips AN, Cambiano V, Miners A, et al. Potential impact on HIV incidence of higher HIV testing rates and earlier antiretroviral therapy initiation in MSM. Potential impact on HIV incidence of higher HIV testing rates and earlier antiretroviral therapy initiation in MSM 2015;29:1855-62.9.Nakagawa F, Lodwick RK, Smith CJ, et al. Projected life expectancy of people with HIV according to timing of diagnosis. Projected life expectancy of people with HIV according to timing of diagnosis 2012;26:335-43.10.Chadborn TR, Delpech VC, Sabin CA, et al. The late diagnosis and consequent short-term mortality of HIV-infected heterosexuals (England and Wales, 2000–2004). The late diagnosis and consequent short-term mortality of HIV-infected heterosexuals (England and Wales, 2000–2004) 2006;20:2371-2379.11.Miners A, Phillips A, Kreif N, et al. Health-related quality-of-life of people with HIV in the era of combination antiretroviral treatment: a cross-sectional comparison with the general population. Health-related quality-of-life of people with HIV in the era of combination antiretroviral treatment: a cross-sectional comparison with the general population 2014;1:e32–e40.12.Cohen MS, Chen YQ, McCauley M, et al. Prevention of HIV-1 Infection with Early Antiretroviral Therapy. Prevention of HIV-1 Infection with Early Antiretroviral Therapy 2011;365:493-505.13.Marks G, Crepaz N, and Janssen RS. Estimating sexual transmission of HIV from persons aware and unaware that they are infected with the virus in the USA. Estimating sexual transmission of HIV from persons aware and unaware that they are infected with the virus in the USA 2006;20:1447-50.14.Nakagawa F, Miners A, Smith CJ, et al. Projected Lifetime Healthcare Costs Associated with HIV Infection. Projected Lifetime Healthcare Costs Associated with HIV Infection 2015;10:e0125018.15.Fleishman JA, Yehia BR, Moore RD, et al. The Economic Burden of Late Entry Into Medical Care for Patients With HIV Infection. The Economic Burden of Late Entry Into Medical Care for Patients With HIV Infection 2010;48:1071-1079.16.Krentz HB and Gill MJ. The Direct Medical Costs of Late Presentation (<350/mm(3)) of HIV Infection over a 15-Year Period. The Direct Medical Costs of Late Presentation (<350/mm(3)) of HIV Infection over a 15-Year Period 2012;2012:757135.17.Krause J, Subklew-Sehume F, Kenyon C, et al. Acceptability of HIV self-testing: a systematic literature review. Acceptability of HIV self-testing: a systematic literature review 2013;13:735.18.Frye V, Wilton L, Hirshfied S, et al. \"Just Because It's Out There, People Aren't Going to Use It.\" HIV Self-Testing Among Young, Black MSM, and Transgender Women. \"Just Because It's Out There, People Aren't Going to Use It.\" HIV Self-Testing Among Young, Black MSM, and Transgender Women 2015.19.Figueroa C, Johnson C, Verster A, et al. Attitudes and Acceptability on HIV Self-testing Among Key Populations: A Literature Review. Attitudes and Acceptability on HIV Self-testing Among Key Populations: A Literature Review 2015.20.Bavinton BR, Brown G, Hurley M, et al. Which gay men would increase their frequency of HIV testing with home self-testing? Which gay men would increase their frequency of HIV testing with home self-testing? 2013;17:2084-92.21.Hensen B, Lewis JJ, Schaap A, et al. Factors associated with HIV-testing and acceptance of an offer of home-based testing by men in rural Zambia. Factors associated with HIV-testing and acceptance of an offer of home-based testing by men in rural Zambia 2015;19:492-504.22.Lee VJ, Tan SC, Earnest A, et al. User acceptability and feasibility of self-testing with HIV rapid tests. User acceptability and feasibility of self-testing with HIV rapid tests 2007;45:449-53.23.Terris-Prestholt F, Hanson K, MacPhail C, et al. How much demand for New HIV prevention technologies can we really expect? Results from a discrete choice experiment in South Africa. How much demand for New HIV prevention technologies can we really expect? Results from a discrete choice experiment in South Africa 2013;8:e83193.24.Ostermann J, Njau B, Brown DS, et al. Heterogeneous HIV Testing Preferences in an Urban Setting in Tanzania: Results from a Discrete Choice Experiment. Heterogeneous HIV Testing Preferences in an Urban Setting in Tanzania: Results from a Discrete Choice Experiment 2014;9:e92100.25.Phillips KA, Maddala T, and Johnson FR. Measuring preferences for health care interventions using conjoint analysis: an application to HIV testing. Measuring preferences for health care interventions using conjoint analysis: an application to HIV testing 2002;37:1681-1705.26.Ryan M. Discrete choice experiments in health care. BMJ 2004;328:360-361.27.Terris-Prestholt F, Quaiffe M, and Vickerman P. Parameterising user uptake in economic evaluations: the role of discrete choice experiments. Parameterising user uptake in economic evaluations: the role of discrete choice experiments In press.28.Philip P, Hickson F, Bonell C, et al. Men who have sex with men in Britain: comparison of estimates from a probability sample and community-based surveys. Men who have sex with men in Britain: comparison of estimates from a probability sample and community-based surveys In submission.29.Orme B. Sample size issues for conjoint analysis, in Getting started with conjoint analysis: strategies for product design and pricing research. 2010, Research Publishers LLC: Madison, Wisconson.)* [2014;](1.Public Health England. HIV in the United Kingdom: 2014 report. 2014.2.Public Health England. HIV new diagnosis, treatment and care: 2015 report.3.Williamson LM, Dodds JP, Mercey DE, et al. Sexual risk behaviour and knowledge of HIV status among community samples of gay men in the UK. Sexual risk behaviour and knowledge of HIV status among community samples of gay men in the UK 2008;22:1063-70.4.Holt M, Rawstorne P, Wilkinson J, et al. HIV testing, gay community involvement and internet use: social and behavioural correlates of HIV testing among Australian men who have sex with men. HIV testing, gay community involvement and internet use: social and behavioural correlates of HIV testing among Australian men who have sex with men 2012;16:13-22.5.Zablotska I, Holt M, de Wit J, et al. Gay men who are not getting tested for HIV. Gay men who are not getting tested for HIV 2012;16:1887-94.6.Witzel TC, Melendez-Torres GJ, Hickson F, et al. HIV testing history and preferences for future tests among gay men, bisexual men and other MSM in England. HIV testing history and preferences for future tests among gay men, bisexual men and other MSM in England In submission.7.UNSW Australia. 2015. Annual report of trends in behaviour 2015. HIV/AIDS, hepatitis and sexually transmissible infections in Australia [Online]. Available: https://csrh.arts.unsw.edu.au/media/CSRHFile/CSRH_Annual_Report_of_Trends_in_Behaviour_2015.pdf.8.Phillips AN, Cambiano V, Miners A, et al. Potential impact on HIV incidence of higher HIV testing rates and earlier antiretroviral therapy initiation in MSM. Potential impact on HIV incidence of higher HIV testing rates and earlier antiretroviral therapy initiation in MSM 2015;29:1855-62.9.Nakagawa F, Lodwick RK, Smith CJ, et al. Projected life expectancy of people with HIV according to timing of diagnosis. Projected life expectancy of people with HIV according to timing of diagnosis 2012;26:335-43.10.Chadborn TR, Delpech VC, Sabin CA, et al. The late diagnosis and consequent short-term mortality of HIV-infected heterosexuals (England and Wales, 2000–2004). The late diagnosis and consequent short-term mortality of HIV-infected heterosexuals (England and Wales, 2000–2004) 2006;20:2371-2379.11.Miners A, Phillips A, Kreif N, et al. Health-related quality-of-life of people with HIV in the era of combination antiretroviral treatment: a cross-sectional comparison with the general population. Health-related quality-of-life of people with HIV in the era of combination antiretroviral treatment: a cross-sectional comparison with the general population 2014;1:e32–e40.12.Cohen MS, Chen YQ, McCauley M, et al. Prevention of HIV-1 Infection with Early Antiretroviral Therapy. Prevention of HIV-1 Infection with Early Antiretroviral Therapy 2011;365:493-505.13.Marks G, Crepaz N, and Janssen RS. Estimating sexual transmission of HIV from persons aware and unaware that they are infected with the virus in the USA. Estimating sexual transmission of HIV from persons aware and unaware that they are infected with the virus in the USA 2006;20:1447-50.14.Nakagawa F, Miners A, Smith CJ, et al. Projected Lifetime Healthcare Costs Associated with HIV Infection. Projected Lifetime Healthcare Costs Associated with HIV Infection 2015;10:e0125018.15.Fleishman JA, Yehia BR, Moore RD, et al. The Economic Burden of Late Entry Into Medical Care for Patients With HIV Infection. The Economic Burden of Late Entry Into Medical Care for Patients With HIV Infection 2010;48:1071-1079.16.Krentz HB and Gill MJ. The Direct Medical Costs of Late Presentation (<350/mm(3)) of HIV Infection over a 15-Year Period. The Direct Medical Costs of Late Presentation (<350/mm(3)) of HIV Infection over a 15-Year Period 2012;2012:757135.17.Krause J, Subklew-Sehume F, Kenyon C, et al. Acceptability of HIV self-testing: a systematic literature review. Acceptability of HIV self-testing: a systematic literature review 2013;13:735.18.Frye V, Wilton L, Hirshfied S, et al. \"Just Because It's Out There, People Aren't Going to Use It.\" HIV Self-Testing Among Young, Black MSM, and Transgender Women. \"Just Because It's Out There, People Aren't Going to Use It.\" HIV Self-Testing Among Young, Black MSM, and Transgender Women 2015.19.Figueroa C, Johnson C, Verster A, et al. Attitudes and Acceptability on HIV Self-testing Among Key Populations: A Literature Review. Attitudes and Acceptability on HIV Self-testing Among Key Populations: A Literature Review 2015.20.Bavinton BR, Brown G, Hurley M, et al. Which gay men would increase their frequency of HIV testing with home self-testing? Which gay men would increase their frequency of HIV testing with home self-testing? 2013;17:2084-92.21.Hensen B, Lewis JJ, Schaap A, et al. Factors associated with HIV-testing and acceptance of an offer of home-based testing by men in rural Zambia. Factors associated with HIV-testing and acceptance of an offer of home-based testing by men in rural Zambia 2015;19:492-504.22.Lee VJ, Tan SC, Earnest A, et al. User acceptability and feasibility of self-testing with HIV rapid tests. User acceptability and feasibility of self-testing with HIV rapid tests 2007;45:449-53.23.Terris-Prestholt F, Hanson K, MacPhail C, et al. How much demand for New HIV prevention technologies can we really expect? Results from a discrete choice experiment in South Africa. How much demand for New HIV prevention technologies can we really expect? Results from a discrete choice experiment in South Africa 2013;8:e83193.24.Ostermann J, Njau B, Brown DS, et al. Heterogeneous HIV Testing Preferences in an Urban Setting in Tanzania: Results from a Discrete Choice Experiment. Heterogeneous HIV Testing Preferences in an Urban Setting in Tanzania: Results from a Discrete Choice Experiment 2014;9:e92100.25.Phillips KA, Maddala T, and Johnson FR. Measuring preferences for health care interventions using conjoint analysis: an application to HIV testing. Measuring preferences for health care interventions using conjoint analysis: an application to HIV testing 2002;37:1681-1705.26.Ryan M. Discrete choice experiments in health care. BMJ 2004;328:360-361.27.Terris-Prestholt F, Quaiffe M, and Vickerman P. Parameterising user uptake in economic evaluations: the role of discrete choice experiments. Parameterising user uptake in economic evaluations: the role of discrete choice experiments In press.28.Philip P, Hickson F, Bonell C, et al. Men who have sex with men in Britain: comparison of estimates from a probability sample and community-based surveys. Men who have sex with men in Britain: comparison of estimates from a probability sample and community-based surveys In submission.29.Orme B. Sample size issues for conjoint analysis, in Getting started with conjoint analysis: strategies for product design and pricing research. 2010, Research Publishers LLC: Madison, Wisconson.)**[1](1.Public Health England. HIV in the United Kingdom: 2014 report. 2014.2.Public Health England. HIV new diagnosis, treatment and care: 2015 report.3.Williamson LM, Dodds JP, Mercey DE, et al. Sexual risk behaviour and knowledge of HIV status among community samples of gay men in the UK. Sexual risk behaviour and knowledge of HIV status among community samples of gay men in the UK 2008;22:1063-70.4.Holt M, Rawstorne P, Wilkinson J, et al. HIV testing, gay community involvement and internet use: social and behavioural correlates of HIV testing among Australian men who have sex with men. HIV testing, gay community involvement and internet use: social and behavioural correlates of HIV testing among Australian men who have sex with men 2012;16:13-22.5.Zablotska I, Holt M, de Wit J, et al. Gay men who are not getting tested for HIV. Gay men who are not getting tested for HIV 2012;16:1887-94.6.Witzel TC, Melendez-Torres GJ, Hickson F, et al. HIV testing history and preferences for future tests among gay men, bisexual men and other MSM in England. HIV testing history and preferences for future tests among gay men, bisexual men and other MSM in England In submission.7.UNSW Australia. 2015. Annual report of trends in behaviour 2015. HIV/AIDS, hepatitis and sexually transmissible infections in Australia [Online]. Available: https://csrh.arts.unsw.edu.au/media/CSRHFile/CSRH_Annual_Report_of_Trends_in_Behaviour_2015.pdf.8.Phillips AN, Cambiano V, Miners A, et al. Potential impact on HIV incidence of higher HIV testing rates and earlier antiretroviral therapy initiation in MSM. Potential impact on HIV incidence of higher HIV testing rates and earlier antiretroviral therapy initiation in MSM 2015;29:1855-62.9.Nakagawa F, Lodwick RK, Smith CJ, et al. Projected life expectancy of people with HIV according to timing of diagnosis. Projected life expectancy of people with HIV according to timing of diagnosis 2012;26:335-43.10.Chadborn TR, Delpech VC, Sabin CA, et al. The late diagnosis and consequent short-term mortality of HIV-infected heterosexuals (England and Wales, 2000–2004). The late diagnosis and consequent short-term mortality of HIV-infected heterosexuals (England and Wales, 2000–2004) 2006;20:2371-2379.11.Miners A, Phillips A, Kreif N, et al. Health-related quality-of-life of people with HIV in the era of combination antiretroviral treatment: a cross-sectional comparison with the general population. Health-related quality-of-life of people with HIV in the era of combination antiretroviral treatment: a cross-sectional comparison with the general population 2014;1:e32–e40.12.Cohen MS, Chen YQ, McCauley M, et al. Prevention of HIV-1 Infection with Early Antiretroviral Therapy. Prevention of HIV-1 Infection with Early Antiretroviral Therapy 2011;365:493-505.13.Marks G, Crepaz N, and Janssen RS. Estimating sexual transmission of HIV from persons aware and unaware that they are infected with the virus in the USA. Estimating sexual transmission of HIV from persons aware and unaware that they are infected with the virus in the USA 2006;20:1447-50.14.Nakagawa F, Miners A, Smith CJ, et al. Projected Lifetime Healthcare Costs Associated with HIV Infection. Projected Lifetime Healthcare Costs Associated with HIV Infection 2015;10:e0125018.15.Fleishman JA, Yehia BR, Moore RD, et al. The Economic Burden of Late Entry Into Medical Care for Patients With HIV Infection. The Economic Burden of Late Entry Into Medical Care for Patients With HIV Infection 2010;48:1071-1079.16.Krentz HB and Gill MJ. The Direct Medical Costs of Late Presentation (<350/mm(3)) of HIV Infection over a 15-Year Period. The Direct Medical Costs of Late Presentation (<350/mm(3)) of HIV Infection over a 15-Year Period 2012;2012:757135.17.Krause J, Subklew-Sehume F, Kenyon C, et al. Acceptability of HIV self-testing: a systematic literature review. Acceptability of HIV self-testing: a systematic literature review 2013;13:735.18.Frye V, Wilton L, Hirshfied S, et al. \"Just Because It's Out There, People Aren't Going to Use It.\" HIV Self-Testing Among Young, Black MSM, and Transgender Women. \"Just Because It's Out There, People Aren't Going to Use It.\" HIV Self-Testing Among Young, Black MSM, and Transgender Women 2015.19.Figueroa C, Johnson C, Verster A, et al. Attitudes and Acceptability on HIV Self-testing Among Key Populations: A Literature Review. Attitudes and Acceptability on HIV Self-testing Among Key Populations: A Literature Review 2015.20.Bavinton BR, Brown G, Hurley M, et al. Which gay men would increase their frequency of HIV testing with home self-testing? Which gay men would increase their frequency of HIV testing with home self-testing? 2013;17:2084-92.21.Hensen B, Lewis JJ, Schaap A, et al. Factors associated with HIV-testing and acceptance of an offer of home-based testing by men in rural Zambia. Factors associated with HIV-testing and acceptance of an offer of home-based testing by men in rural Zambia 2015;19:492-504.22.Lee VJ, Tan SC, Earnest A, et al. User acceptability and feasibility of self-testing with HIV rapid tests. User acceptability and feasibility of self-testing with HIV rapid tests 2007;45:449-53.23.Terris-Prestholt F, Hanson K, MacPhail C, et al. How much demand for New HIV prevention technologies can we really expect? Results from a discrete choice experiment in South Africa. How much demand for New HIV prevention technologies can we really expect? Results from a discrete choice experiment in South Africa 2013;8:e83193.24.Ostermann J, Njau B, Brown DS, et al. Heterogeneous HIV Testing Preferences in an Urban Setting in Tanzania: Results from a Discrete Choice Experiment. Heterogeneous HIV Testing Preferences in an Urban Setting in Tanzania: Results from a Discrete Choice Experiment 2014;9:e92100.25.Phillips KA, Maddala T, and Johnson FR. Measuring preferences for health care interventions using conjoint analysis: an application to HIV testing. Measuring preferences for health care interventions using conjoint analysis: an application to HIV testing 2002;37:1681-1705.26.Ryan M. Discrete choice experiments in health care. BMJ 2004;328:360-361.27.Terris-Prestholt F, Quaiffe M, and Vickerman P. Parameterising user uptake in economic evaluations: the role of discrete choice experiments. Parameterising user uptake in economic evaluations: the role of discrete choice experiments In press.28.Philip P, Hickson F, Bonell C, et al. Men who have sex with men in Britain: comparison of estimates from a probability sample and community-based surveys. Men who have sex with men in Britain: comparison of estimates from a probability sample and community-based surveys In submission.29.Orme B. Sample size issues for conjoint analysis, in Getting started with conjoint analysis: strategies for product design and pricing research. 2010, Research Publishers LLC: Madison, Wisconson.)**[:e32–e40.](1.Public Health England. HIV in the United Kingdom: 2014 report. 2014.2.Public Health England. HIV new diagnosis, treatment and care: 2015 report.3.Williamson LM, Dodds JP, Mercey DE, et al. Sexual risk behaviour and knowledge of HIV status among community samples of gay men in the UK. Sexual risk behaviour and knowledge of HIV status among community samples of gay men in the UK 2008;22:1063-70.4.Holt M, Rawstorne P, Wilkinson J, et al. HIV testing, gay community involvement and internet use: social and behavioural correlates of HIV testing among Australian men who have sex with men. HIV testing, gay community involvement and internet use: social and behavioural correlates of HIV testing among Australian men who have sex with men 2012;16:13-22.5.Zablotska I, Holt M, de Wit J, et al. Gay men who are not getting tested for HIV. Gay men who are not getting tested for HIV 2012;16:1887-94.6.Witzel TC, Melendez-Torres GJ, Hickson F, et al. HIV testing history and preferences for future tests among gay men, bisexual men and other MSM in England. HIV testing history and preferences for future tests among gay men, bisexual men and other MSM in England In submission.7.UNSW Australia. 2015. Annual report of trends in behaviour 2015. HIV/AIDS, hepatitis and sexually transmissible infections in Australia [Online]. Available: https://csrh.arts.unsw.edu.au/media/CSRHFile/CSRH_Annual_Report_of_Trends_in_Behaviour_2015.pdf.8.Phillips AN, Cambiano V, Miners A, et al. Potential impact on HIV incidence of higher HIV testing rates and earlier antiretroviral therapy initiation in MSM. Potential impact on HIV incidence of higher HIV testing rates and earlier antiretroviral therapy initiation in MSM 2015;29:1855-62.9.Nakagawa F, Lodwick RK, Smith CJ, et al. Projected life expectancy of people with HIV according to timing of diagnosis. Projected life expectancy of people with HIV according to timing of diagnosis 2012;26:335-43.10.Chadborn TR, Delpech VC, Sabin CA, et al. The late diagnosis and consequent short-term mortality of HIV-infected heterosexuals (England and Wales, 2000–2004). The late diagnosis and consequent short-term mortality of HIV-infected heterosexuals (England and Wales, 2000–2004) 2006;20:2371-2379.11.Miners A, Phillips A, Kreif N, et al. Health-related quality-of-life of people with HIV in the era of combination antiretroviral treatment: a cross-sectional comparison with the general population. Health-related quality-of-life of people with HIV in the era of combination antiretroviral treatment: a cross-sectional comparison with the general population 2014;1:e32–e40.12.Cohen MS, Chen YQ, McCauley M, et al. Prevention of HIV-1 Infection with Early Antiretroviral Therapy. Prevention of HIV-1 Infection with Early Antiretroviral Therapy 2011;365:493-505.13.Marks G, Crepaz N, and Janssen RS. Estimating sexual transmission of HIV from persons aware and unaware that they are infected with the virus in the USA. Estimating sexual transmission of HIV from persons aware and unaware that they are infected with the virus in the USA 2006;20:1447-50.14.Nakagawa F, Miners A, Smith CJ, et al. Projected Lifetime Healthcare Costs Associated with HIV Infection. Projected Lifetime Healthcare Costs Associated with HIV Infection 2015;10:e0125018.15.Fleishman JA, Yehia BR, Moore RD, et al. The Economic Burden of Late Entry Into Medical Care for Patients With HIV Infection. The Economic Burden of Late Entry Into Medical Care for Patients With HIV Infection 2010;48:1071-1079.16.Krentz HB and Gill MJ. The Direct Medical Costs of Late Presentation (<350/mm(3)) of HIV Infection over a 15-Year Period. The Direct Medical Costs of Late Presentation (<350/mm(3)) of HIV Infection over a 15-Year Period 2012;2012:757135.17.Krause J, Subklew-Sehume F, Kenyon C, et al. Acceptability of HIV self-testing: a systematic literature review. Acceptability of HIV self-testing: a systematic literature review 2013;13:735.18.Frye V, Wilton L, Hirshfied S, et al. \"Just Because It's Out There, People Aren't Going to Use It.\" HIV Self-Testing Among Young, Black MSM, and Transgender Women. \"Just Because It's Out There, People Aren't Going to Use It.\" HIV Self-Testing Among Young, Black MSM, and Transgender Women 2015.19.Figueroa C, Johnson C, Verster A, et al. Attitudes and Acceptability on HIV Self-testing Among Key Populations: A Literature Review. Attitudes and Acceptability on HIV Self-testing Among Key Populations: A Literature Review 2015.20.Bavinton BR, Brown G, Hurley M, et al. Which gay men would increase their frequency of HIV testing with home self-testing? Which gay men would increase their frequency of HIV testing with home self-testing? 2013;17:2084-92.21.Hensen B, Lewis JJ, Schaap A, et al. Factors associated with HIV-testing and acceptance of an offer of home-based testing by men in rural Zambia. Factors associated with HIV-testing and acceptance of an offer of home-based testing by men in rural Zambia 2015;19:492-504.22.Lee VJ, Tan SC, Earnest A, et al. User acceptability and feasibility of self-testing with HIV rapid tests. User acceptability and feasibility of self-testing with HIV rapid tests 2007;45:449-53.23.Terris-Prestholt F, Hanson K, MacPhail C, et al. How much demand for New HIV prevention technologies can we really expect? Results from a discrete choice experiment in South Africa. How much demand for New HIV prevention technologies can we really expect? Results from a discrete choice experiment in South Africa 2013;8:e83193.24.Ostermann J, Njau B, Brown DS, et al. Heterogeneous HIV Testing Preferences in an Urban Setting in Tanzania: Results from a Discrete Choice Experiment. Heterogeneous HIV Testing Preferences in an Urban Setting in Tanzania: Results from a Discrete Choice Experiment 2014;9:e92100.25.Phillips KA, Maddala T, and Johnson FR. Measuring preferences for health care interventions using conjoint analysis: an application to HIV testing. Measuring preferences for health care interventions using conjoint analysis: an application to HIV testing 2002;37:1681-1705.26.Ryan M. Discrete choice experiments in health care. BMJ 2004;328:360-361.27.Terris-Prestholt F, Quaiffe M, and Vickerman P. Parameterising user uptake in economic evaluations: the role of discrete choice experiments. Parameterising user uptake in economic evaluations: the role of discrete choice experiments In press.28.Philip P, Hickson F, Bonell C, et al. Men who have sex with men in Britain: comparison of estimates from a probability sample and community-based surveys. Men who have sex with men in Britain: comparison of estimates from a probability sample and community-based surveys In submission.29.Orme B. Sample size issues for conjoint analysis, in Getting started with conjoint analysis: strategies for product design and pricing research. 2010, Research Publishers LLC: Madison, Wisconson.)
[truncated: 677,674 more chars]
