# Supplementary material for: Preferences for HIV testing services among men who have sex with men in the UK: A discrete choice experiment
Source: PLoS Med. 2019 Apr 11;16(4):e1002779. doi: 10.1371/journal.pmed.1002779 (PMC6459507; doi:10.1371/journal.pmed.1002779)
Supplement: S1 Text — (DOCX) [file pmed.1002779.s004.docx]

**Question 1, version A**

Imagine that yesterday you had condomless anal sex with someone whose HIV status you're unsure of. What are you most likely to do?

|  | **Take an HIV test by yourself** | **Get tested for HIV by a health care professional** | **Not take an HIV test** |
| --- | --- | --- | --- |
| **How long you have to wait before you can take a test** | 4 weeks time | 4 weeks time |  |
| **Where you can take the test** | Somewhere convenient to you, such as your home | At a sexual health clinic |  |
| **How the test is taken** | Using an oral swab (which is similar to a cotton bud) | A blood sample via a syringe |  |
| **How you get the test** | 'Click and collect' from a pharmacy or health clinic | Book and attend an appointment |  |
| **How long you have to wait for a test result once you have taken it** | You need to put your sample in the post and will receive a call with your result in 3 days from a health care professional | There and then in 30 minutes |  |
| **Who you can talk with about your result** | You can talk with the person who gives you your result and advice is available online | You can talk with the person who gives you your result and advice is available online |  |
| **Test accuracy** | There is a 95% chance the test result is accurate | There is a 95% chance the test result is accurate |  |
| **Whether you can also test for other infections such as syphilis and gonorrhoea at the same time** | Yes | Yes |  |
| **How much you have to pay for the test** | £10 | Its free |  |
| **Which option would you most prefer?**  **(tick one box)** |  |  |  |

**Question 2, version A**

Imagine that yesterday you had condomless anal sex with someone whose HIV status you're unsure of. What are you most likely to do?

|  | **Take an HIV test by yourself** | **Get tested for HIV by a health care professional** | **Not take an HIV test** |
| --- | --- | --- | --- |
| **How long you have to wait before you can take a test** | 12 weeks time | 4 weeks time |  |
| **Where you can take the test** | Somewhere convenient to you, such as your home | At your GPs |  |
| **How the test is taken** | Using an oral swab (which is similar to a cotton bud) | A drop of blood from a skin prick |  |
| **How you get the test** | Order it online | Just drop in and wait your turn |  |
| **How long you have to wait for a test result once you have taken it** | There and then in 10 minutes | You will receive a call with your result in 3 days time from a health care professional |  |
| **Who you can talk with about your result** | A free phone number to a health care professional is provided and advice is available online | You can talk with the person who gives you your result and advice is available online |  |
| **Test accuracy** | There is a 99% chance the test result is accurate | There is a 95% chance the test result is accurate |  |
| **Whether you can also test for other infections such as syphilis and gonorrhoea at the same time** | No | Yes |  |
| **How much you have to pay for the test** | £10 | Its free |  |
| **Which option would you most prefer?**  **(tick one box)** |  |  |  |

**Question 3, version A**

Imagine that yesterday you had condomless anal sex with someone whose HIV status you're unsure of. What are you most likely to do?

|  | **Take an HIV test by yourself** | **Get tested for HIV by a health care professional** | **Not take an HIV test** |
| --- | --- | --- | --- |
| **How long you have to wait before you can take a test** | 4 weeks time | 4 weeks time |  |
| **Where you can take the test** | Somewhere convenient to you, such as your home | At a community location such as an HIV charity |  |
| **How the test is taken** | A drop of blood from a skin prick | A drop of blood from a skin prick |  |
| **How you get the test** | 'Click and collect' from a pharmacy or health clinic | Just drop in and wait your turn |  |
| **How long you have to wait for a test result once you have taken it** | There and then in 10 minutes | There and then in 10 minutes |  |
| **Who you can talk with about your result** | A free phone number to a health care professional is provided and advice is available online | You can talk with the person who gives you your results and advice is available online |  |
| **Test accuracy** | There is a 95% chance the test result is accurate | There is a 95% chance the test result is accurate |  |
| **Whether you can also test for other infections such as syphilis and gonorrhoea at the same time** | No | No |  |
| **How much you have to pay for the test** | £30 | Its free |  |
| **Which option would you most prefer?**  **(tick one box)** |  |  |  |

**Question 4, version A**

Imagine that yesterday you had condomless anal sex with someone whose HIV status you're unsure of. What are you most likely to do?

|  | **Take an HIV test by yourself** | **Get tested for HIV by a health care professional** | **Not take an HIV test** |
| --- | --- | --- | --- |
| **How long you have to wait before you can take a test** | 4 weeks time | 4 weeks time |  |
| **Where you can take the test** | Somewhere convenient to you, such as your home | At a mobile clinic based at a bar, club or sauna |  |
| **How the test is taken** | Using an oral swab (which is similar to a cotton bud) | A drop of blood from a skin prick |  |
| **How you get the test** | 'Click and collect' from a pharmacy or health clinic | Just drop in and wait your turn |  |
| **How long you have to wait for a test result once you have taken it** | There and then in 10 minutes | You will receive a call with your result in 3 days time from a health care professional |  |
| **Who you can talk with about your result** | A free phone number to a health care professional is provided and advice is available online | You can talk with the person who gives you your result and advice is available online |  |
| **Test accuracy** | There is a 99% chance the test result is accurate | There is a 99% chance the test result is accurate |  |
| **Whether you can also test for other infections such as syphilis and gonorrhoea at the same time** | No | No |  |
| **How much you have to pay for the test** | Its free | Its free |  |
| **Which option would you most prefer?**  **(tick one box)** |  |  |  |

**Question 5, version A**

Imagine that yesterday you had condomless anal sex with someone whose HIV status you're unsure of. What are you most likely to do?

|  | **Take an HIV test by yourself** | **Get tested for HIV by a health care professional** | **Not take an HIV test** |
| --- | --- | --- | --- |
| **How long you have to wait before you can take a test** | 12 weeks time | 4 weeks time |  |
| **Where you can take the test** | Somewhere convenient to you, such as your home | At your GPs |  |
| **How the test is taken** | A drop of blood from a skin prick | A drop of blood from a skin prick |  |
| **How you get the test** | 'Click and collect' from a pharmacy or health clinic | Just drop in and wait your turn |  |
| **How long you have to wait for a test result once you have taken it** | You need to put your sample in the post and will receive a call with your result in 3 days from a health care professional. | There and then in 30 minutes. |  |
| **Who you can talk with about your result** | You can talk with the person who gives you your result and advice is available online | You can talk with the person who gives you your result and advice is available online |  |
| **Test accuracy** | There is a 99% chance the test result is accurate | There is a 95% chance the test result is accurate |  |
| **Whether you can also test for other infections such as syphilis and gonorrhoea at the same time** | No | No |  |
| **How much you have to pay for the test** | Its free | Its free |  |
| **Which option would you most prefer?**  **(tick one box)** |  |  |  |

**Question 6, version A**

Imagine that yesterday you had condomless anal sex with someone whose HIV status you're unsure of. What are you most likely to do?

|  | **Take an HIV test by yourself** | **Get tested for HIV by a health care professional** | **Not take an HIV test** |
| --- | --- | --- | --- |
| **How long you have to wait before you can take a test** | 4 weeks time | 4 weeks time |  |
| **Where you can take the test** | Somewhere convenient to you, such as your home | At your GPs |  |
| **How the test is taken** | Using an oral swab (which is similar to a cotton bud) | A blood sample via a syringe |  |
| **How you get the test** | 'Click and collect' from a pharmacy or health clinic | Just drop in and wait your turn |  |
| **How long you have to wait for a test result once you have taken it** | There and then in 30 minutes | You will receive a call with your result sometime the same day from a health care professional |  |
| **Who you can talk with about your result** | A free phone number to a health care professional is provided and advice is available online | You can talk with the person who gives you your result and advice is available online |  |
| **Test accuracy** | There is a 99% chance the test result is accurate | There is a 99% chance the test result is accurate |  |
| **Whether you can also test for other infections such as syphilis and gonorrhoea at the same time** | Yes | Yes |  |
| **How much you have to pay for the test** | £30 | Its free |  |
| **Which option would you most prefer?**  **(tick one box)** |  |  |  |

**Question 7, version A**

Imagine that yesterday you had condomless anal sex with someone whose HIV status you're unsure of. What are you most likely to do?

|  | **Take an HIV test by yourself** | **Get tested for HIV by a health care professional** | **Not take an HIV test** |
| --- | --- | --- | --- |
| **How long you have to wait before you can take a test** | 12 weeks time | 4 weeks time |  |
| **Where you can take the test** | Somewhere convenient to you, such as your home | At your GPs |  |
| **How the test is taken** | A drop of blood from a skin prick | A drop of blood from a skin prick |  |
| **How you get the test** | Order it online | Book and attend an appointment |  |
| **How long you have to wait for a test result once you have taken it** | There and then in 30 minutes | There and then in 10 minutes |  |
| **Who you can talk with about your result** | A free phone number to a health care professional is provided and advice is available online | You can talk with the person who gives you your result and advice is available online |  |
| **Test accuracy** | There is a 95% chance the test result is accurate | There is a 95% chance the test result is accurate |  |
| **Whether you can also test for other infections such as syphilis and gonorrhoea at the same time** | Yes | Yes |  |
| **How much you have to pay for the test** | Its free | Its free |  |
| **Which option would you most prefer?**  **(tick one box)** |  |  |  |

**Question 8, version A**

Imagine that yesterday you had condomless anal sex with someone whose HIV status you're unsure of. What are you most likely to do?

|  | **Take an HIV test by yourself** | **Get tested for HIV by a health care professional** | **Not take an HIV test** |
| --- | --- | --- | --- |
| **How long you have to wait before you can take a test** | 12 weeks time | 4 weeks time |  |
| **Where you can take the test** | Somewhere convenient to you, such as your home | At a community location such as an HIV charity |  |
| **How the test is taken** | A drop of blood from a skin prick | A drop of blood from a skin prick |  |
| **How you get the test** | 'Click and collect' from a pharmacy or health clinic | Just drop in and wait your turn |  |
| **How long you have to wait for a test result once you have taken it** | There and then in 30 minutes | You will receive a call with your result sometime the same day from a health care professional |  |
| **Who you can talk with about your result** | A free phone number to a health care professional is provided and advice is available online | You can talk with the person who gives you your result and advice is available online |  |
| **Test accuracy** | There is a 99% chance the test result is accurate | There is a 99% chance the test result is accurate |  |
| **Whether you can also test for other infections such as syphilis and gonorrhoea at the same time** | No | No |  |
| **How much you have to pay for the test** | £10 | Its free |  |
| **Which option would you most prefer?**  **(tick one box)** |  |  |  |

**Question 9, version A**

Imagine that yesterday you had condomless anal sex with someone whose HIV status you're unsure of. What are you most likely to do?

|  | **Take an HIV test by yourself** | **Get tested for HIV by a health care professional** | **Not take an HIV test** |
| --- | --- | --- | --- |
| **How long you have to wait before you can take a test** | 4 weeks time | 4 weeks time |  |
| **Where you can take the test** | Somewhere convenient to you, such as your home | At your GPs |  |
| **How the test is taken** | Using an oral swab (which is similar to a cotton bud) | A drop of blood from a skin prick |  |
| **How you get the test** | Order it online | Book and attend an appointment |  |
| **How long you have to wait for a test result once you have taken it** | You need to put your sample in the post and will receive a call with your result in 3 days from a health care professional | You will receive a call with your result sometime the same day from a health care professional. |  |
| **Who you can talk with about your result** | You can talk with the person who gives you your result and advice is available online | You can talk with the person who gives you your result and advice is available online |  |
| **Test accuracy** | There is a 95% chance the test result is accurate | There is a 99% chance the test result is accurate |  |
| **Whether you can also test for other infections such as syphilis and gonorrhoea at the same time** | No | Yes |  |
| **How much you have to pay for the test** | £10 | Its free |  |
| **Which option would you most prefer?**  **(tick one box)** |  |  |  |

**Question 10, version A**

Imagine that yesterday you had condomless anal sex with someone whose HIV status you're unsure of. What are you most likely to do?

|  | **Take an HIV test by yourself** | **Get tested for HIV by a health care professional** | **Not take an HIV test** |
| --- | --- | --- | --- |
| **How long you have to wait before you can take a test** | 4 weeks time | 4 weeks time |  |
| **Where you can take the test** | Somewhere convenient to you, such as your home | At a sexual health clinic |  |
| **How the test is taken** | Using an oral swab (which is similar to a cotton bud) | A drop of blood from a skin prick |  |
| **How you get the test** | Order it online | Just drop in and wait your turn |  |
| **How long you have to wait for a test result once you have taken it** | There and then in 30 minutes | You will receive a call with your result in 3 days time from a health care professional |  |
| **Who you can talk with about your result** | A free phone number to a health care professional is provided and advice is available online | You can talk with the person who gives you your result and advice is available online |  |
| **Test accuracy** | There is a 95% chance the test result is accurate | There is a 95% chance the test result is accurate |  |
| **Whether you can also test for other infections such as syphilis and gonorrhoea at the same time** | Yes | No |  |
| **How much you have to pay for the test** | £20 | Its free |  |
| **Which option would you most prefer?**  **(tick one box)** |  |  |  |

**Question 11, version A**

Imagine that yesterday you had condomless anal sex with someone whose HIV status you're unsure of. What are you most likely to do?

|  | **Take an HIV test by yourself** | **Get tested for HIV by a health care professional** | **Not take an HIV test** |
| --- | --- | --- | --- |
| **How long you have to wait before you can take a test** | 12 weeks time | 4 weeks time |  |
| **Where you can take the test** | Somewhere convenient to you, such as your home | At a sexual health clinic |  |
| **How the test is taken** | A drop of blood from a skin prick | A drop of blood from a skin prick |  |
| **How you get the test** | Order it online | Book and attend an appointment |  |
| **How long you have to wait for a test result once you have taken it** | You need to put your sample in the post and will receive a call with your result in 3 days from a health care professional. | You will receive a call with your result in 3 days time from a health care professional |  |
| **Who you can talk with about your result** | You can talk with the person who gives you your result and advice is available online | You can talk with the person who gives you your result and advice is available online |  |
| **Test accuracy** | There is a 99% chance the test result is accurate | There is a 99% chance the test result is accurate |  |
| **Whether you can also test for other infections such as syphilis and gonorrhoea at the same time** | Yes | Yes |  |
| **How much you have to pay for the test** | £30 | Its free |  |
| **Which option would you most prefer?**  **(tick one box)** |  |  |  |

**Question 12, version A**

Imagine that yesterday you had condomless anal sex with someone whose HIV status you're unsure of. What are you most likely to do?

|  | **Take an HIV test by yourself** | **Get tested for HIV by a health care professional** | **Not take an HIV test** |
| --- | --- | --- | --- |
| **How long you have to wait before you can take a test** | 12 weeks time | 4 weeks time |  |
| **Where you can take the test** | Somewhere convenient to you, such as your home | At your GPs |  |
| **How the test is taken** | A drop of blood from a skin prick | A blood sample via a syringe |  |
| **How you get the test** | Order it online | Book and attend an appointment |  |
| **How long you have to wait for a test result once you have taken it** | There and then in 10 minutes | There and then in 30 minutes |  |
| **Who you can talk with about your result** | A free phone number to a health care professional is provided and advice is available online | You can talk with the person who gives you your result and advice is available online |  |
| **Test accuracy** | There is a 95% chance the test result is accurate | There is a 99% chance the test result is accurate |  |
| **Whether you can also test for other infections such as syphilis and gonorrhoea at the same time** | Yes | No |  |
| **How much you have to pay for the test** | £20 | Its free |  |
| **Which option would you most prefer?**  **(tick one box)** |  |  |  |

**Question 1, version B**

Imagine that yesterday you had condomless anal sex with someone whose HIV status you're unsure of. What are you most likely to do?

|  | **Take an HIV test by yourself** | **Get tested for HIV by a health care professional** | **Not take an HIV test** |
| --- | --- | --- | --- |
| **How long you have to wait before you can take a test** | 12 weeks time | 4 weeks time |  |
| **Where you can take the test** | Somewhere convenient to you, such as your home | At a community location such as an HIV charity |  |
| **How the test is taken** | Using an oral swab (which is similar to a cotton bud) | A drop of blood from a skin prick |  |
| **How you get the test** | 'Click and collect' from a pharmacy or health clinic | Just drop in and wait your turn |  |
| **How long you have to wait for a test result once you have taken it** | You need to put your sample in the post and will receive a call with your result in 7 days from a health care professional | You will receive a call with your result in 3 days time from a health care professional |  |
| **Who you can talk with about your result** | You can talk with the person who gives you your result and advice is available online | You can talk with the person who gives you your result and advice is available online |  |
| **Test accuracy** | There is a 95% chance the test result is accurate | There is a 95% chance the test result is accurate |  |
| **Whether you can also test for other infections such as syphilis and gonorrhoea at the same time** | No | Yes |  |
| **How much you have to pay for the test** | £20 | Its free |  |
| **Which option would you most prefer?**  **(tick one box)** |  |    |    |

**Question 2, version B**

Imagine that yesterday you had condomless anal sex with someone whose HIV status you're unsure of. What are you most likely to do?

|  | **Take an HIV test by yourself** | **Get tested for HIV by a health care professional** | **Not take an HIV test** |
| --- | --- | --- | --- |
| **How long you have to wait before you can take a test** | 12 weeks time | 4 weeks time |  |
| **Where you can take the test** | Somewhere convenient to you, such as your home | At a sexual health clinic |  |
| **How the test is taken** | A drop of blood from a skin prick | A drop of blood from a skin prick |  |
| **How you get the test** | 'Click and collect' from a pharmacy or health clinic | Just drop in and wait your turn |  |
| **How long you have to wait for a test result once you have taken it** | You need to put your sample in the post and will receive a call with your result in 7 days from a health care professional | There and then in 10 minutes |  |
| **Who you can talk with about your result** | You can talk with the person who gives you your result and advice is available online | You can talk with the person who gives you your result and advice is available online |  |
| **Test accuracy** | There is a 95% chance the test result is accurate | There is a 99% chance the test result is accurate |  |
| **Whether you can also test for other infections such as syphilis and gonorrhoea at the same time** | Yes | No |  |
| **How much you have to pay for the test** | £10 | Its free |  |
| **Which option would you most prefer?**  **(tick one box)** |  |  |  |

**Question 3, version B**

Imagine that yesterday you had condomless anal sex with someone whose HIV status you're unsure of. What are you most likely to do?

|  | **Take an HIV test by yourself** | **Get tested for HIV by a health care professional** | **Not take an HIV test** |
| --- | --- | --- | --- |
| **How long you have to wait before you can take a test** | 4 weeks time | 4 weeks time |  |
| **Where you can take the test** | Somewhere convenient to you, such as your home | At your GPs |  |
| **How the test is taken** | A drop of blood from a skin prick | A blood sample via a syringe |  |
| **How you get the test** | Order it online | Book and attend an appointment |  |
| **How long you have to wait for a test result once you have taken it** | You need to put your sample in the post and will receive a call with your result in 7 days from a health care professional | You will receive a call with your result sometime the same day from a health care professional |  |
| **Who you can talk with about your result** | You can talk with the person who gives you your result and advice is available online | You can talk with the person who gives you your result and advice is available online |  |
| **Test accuracy** | There is a 99% chance the test result is accurate | There is a 95% chance the test result is accurate |  |
| **Whether you can also test for other infections such as syphilis and gonorrhoea at the same time** | No | No |  |
| **How much you have to pay for the test** | £20 | Its free |  |
| **Which option would you most prefer?**  **(tick one box)** |  |  |  |

**Question 4, version B**

Imagine that yesterday you had condomless anal sex with someone whose HIV status you're unsure of. What are you most likely to do?

|  | **Take an HIV test by yourself** | **Get tested for HIV by a health care professional** | **Not take an HIV test** |
| --- | --- | --- | --- |
| **How long you have to wait before you can take a test** | 12 weeks time | 4 weeks time |  |
| **Where you can take the test** | Somewhere convenient to you, such as your home | At a mobile clinic based at a bar, club or sauna |  |
| **How the test is taken** | Using an oral swab (which is similar to a cotton bud) | A drop of blood from a skin prick |  |
| **How you get the test** | 'Click and collect' from a pharmacy or health clinic | Just drop in and wait your turn |  |
| **How long you have to wait for a test result once you have taken it** | You need to put your sample in the post and will receive a call with your result in 3 days from a health care professional | There and then in 10 minutes. |  |
| **Who you can talk with about your result** | . You can talk with the person who gives you your result and advice is available online | You can talk with the person who gives you your result and advice is available online |  |
| **Test accuracy** | There is a 99% chance the test result is accurate | There is a 99% chance the test result is accurate |  |
| **Whether you can also test for other infections such as syphilis and gonorrhoea at the same time** | Yes | No |  |
| **How much you have to pay for the test** | £20 | Its free |  |
| **Which option would you most prefer?**  **(tick one box)** |  |  |  |

**Question 5, version B**

Imagine that yesterday you had condomless anal sex with someone whose HIV status you're unsure of. What are you most likely to do?

|  | **Take an HIV test by yourself** | **Get tested for HIV by a health care professional** | **Not take an HIV test** |
| --- | --- | --- | --- |
| **How long you have to wait before you can take a test** | 4 weeks time | 4 weeks time |  |
| **Where you can take the test** | Somewhere convenient to you, such as your home | At a sexual health clinic |  |
| **How the test is taken** | A drop of blood from a skin prick | A drop of blood from a skin prick |  |
| **How you get the test** | 'Click and collect' from a pharmacy or health clinic | Book and attend an appointment |  |
| **How long you have to wait for a test result once you have taken it** | There and then in 30 minutes | There and then in 10 minutes |  |
| **Who you can talk with about your result** | A free phone number to a health care professional is provided and advice is available online | You can talk with the person who gives you your result and advice is available online |  |
| **Test accuracy** | There is a 99% chance the test result is accurate | There is a 99% chance the test result is accurate |  |
| **Whether you can also test for other infections such as syphilis and gonorrhoea at the same time** | No | Yes |  |
| **How much you have to pay for the test** | £20 | Its free |  |
| **Which option would you most prefer?**  **(tick one box)** |  |  |  |

**Question 6, version B**

Imagine that yesterday you had condomless anal sex with someone whose HIV status you're unsure of. What are you most likely to do?

|  | **Take an HIV test by yourself** | **Get tested for HIV by a health care professional** | **Not take an HIV test** |
| --- | --- | --- | --- |
| **How long you have to wait before you can take a test** | 12 weeks time | 4 weeks time |  |
| **Where you can take the test** | Somewhere convenient to you, such as your home | At a sexual health clinic |  |
| **How the test is taken** | Using an oral swab (which is similar to a cotton bud) | A blood sample via a syringe |  |
| **How you get the test** | Order it online | Book and attend an appointment |  |
| **How long you have to wait for a test result once you have taken it** | You need to put your sample in the post and will receive a call with your result in 7 days from a health care professional | You will receive a call with your result sometime the same day from a health care professional |  |
| **Who you can talk with about your result** | You can talk with the person who gives you your result and advice is available online | You can talk with the person who gives you your result and advice is available online |  |
| **Test accuracy** | There is a 99% chance the test result is accurate | There is a 95% chance the test result is accurate |  |
| **Whether you can also test for other infections such as syphilis and gonorrhoea at the same time** | No | No |  |
| **How much you have to pay for the test** | £30 | Its free |  |
| **Which option would you most prefer?**  **(tick one box)** |  |  |  |

**Question 7, version B**

Imagine that yesterday you had condomless anal sex with someone whose HIV status you're unsure of. What are you most likely to do?

|  | **Take an HIV test by yourself** | **Get tested for HIV by a health care professional** | **Not take an HIV test** |
| --- | --- | --- | --- |
| **How long you have to wait before you can take a test** | 4 weeks time | 4 weeks time |  |
| **Where you can take the test** | Somewhere convenient to you, such as your home | At a community location such as an HIV charity |  |
| **How the test is taken** | Using an oral swab (which is similar to a cotton bud) | A drop of blood from a skin prick |  |
| **How you get the test** | Order it online | Just drop in and wait your turn |  |
| **How long you have to wait for a test result once you have taken it** | You need to put your sample in the post and will receive a call with your result in 7 days from a health care professional | There and then in 30 minutes |  |
| **Who you can talk with about your result** | You can talk with the person who gives you your result and advice is available online | You can talk with the person who gives you your result and advice is available online |  |
| **Test accuracy** | There is a 99% chance the test result is accurate | There is a 99% chance the test result is accurate |  |
| **Whether you can also test for other infections such as syphilis and gonorrhoea at the same time** | Yes | Yes |  |
| **How much you have to pay for the test** | Its free | Its free |  |
| **Which option would you most prefer?**  **(tick one box)** |  |  |  |

**Question 8, version B**

Imagine that yesterday you had condomless anal sex with someone whose HIV status you're unsure of. What are you most likely to do?

|  | **Take an HIV test by yourself** | **Get tested for HIV by a health care professional** | **Not take an HIV test** |
| --- | --- | --- | --- |
| **How long you have to wait before you can take a test** | 4 weeks time | 4 weeks time |  |
| **Where you can take the test** | Somewhere convenient to you, such as your home | At a sexual health clinic |  |
| **How the test is taken** | A drop of blood from a skin prick | A blood sample via a syringe |  |
| **How you get the test** | Order it online | Just drop in and wait your turn |  |
| **How long you have to wait for a test result once you have taken it** | You need to put your sample in the post and will receive a call with your result in 3 days from a health care professional | You will receive a call with your result sometime the same day from a health care professional |  |
| **Who you can talk with about your result** | You can talk with the person who gives you your result and advice is available online | You can talk with the person who gives you your result and advice is available online |  |
| **Test accuracy** | There is a 95% chance the test result is accurate | There is a 99% chance the test result is accurate |  |
| **Whether you can also test for other infections such as syphilis and gonorrhoea at the same time** | No | Yes |  |
| **How much you have to pay for the test** | Its free | Its free |  |
| **Which option would you most prefer?**  **(tick one box)** |  |  |  |

**Question 9, version B**

Imagine that yesterday you had condomless anal sex with someone whose HIV status you're unsure of. What are you most likely to do?

|  | **Take an HIV test by yourself** | **Get tested for HIV by a health care professional** | **Not take an HIV test** |
| --- | --- | --- | --- |
| **How long you have to wait before you can take a test** | 4 weeks time | 4 weeks time |  |
| **Where you can take the test** | Somewhere convenient to you, such as your home | At your GPs |  |
| **How the test is taken** | A drop of blood from a skin prick | A drop of blood from a skin prick |  |
| **How you get the test** | 'Click and collect' from a pharmacy or health clinic | Book and attend an appointment |  |
| **How long you have to wait for a test result once you have taken it** | You need to put your sample in the post and will receive a call with your result in 7 days from a health care professional | You will receive a call with your result in 3 days time from a health care professional |  |
| **Who you can talk with about your result** | You can talk with the person who gives you your result and advice is available online | You can talk with the person who gives you your result and advice is available online |  |
| **Test accuracy** | There is a 95% chance the test result is accurate | There is a 99% chance the test result is accurate |  |
| **Whether you can also test for other infections such as syphilis and gonorrhoea at the same time** | Yes | No |  |
| **How much you have to pay for the test** | £30 | Its free |  |
| **Which option would you most prefer?**  **(tick one box)** |  |  |  |

**Question 10, version B**

Imagine that yesterday you had condomless anal sex with someone whose HIV status you're unsure of. What are you most likely to do?

|  | **Take an HIV test by yourself** | **Get tested for HIV by a health care professional** | **Not take an HIV test** |
| --- | --- | --- | --- |
| **How long you have to wait before you can take a test** | 12 weeks time | 4 weeks time |  |
| **Where you can take the test** | Somewhere convenient to you, such as your home | At a sexual health clinic |  |
| **How the test is taken** | Using an oral swab (which is similar to a cotton bud) | A drop of blood from a skin prick |  |
| **How you get the test** | 'Click and collect' from a pharmacy or health clinic | Book and attend an appointment |  |
| **How long you have to wait for a test result once you have taken it** | There and then in 10 minutes | You will receive a call with your result sometime the same day from a health care professional |  |
| **Who you can talk with about your result** | A free phone number to a health care professional is provided and advice is available online | You can talk with the person who gives you your result and advice is available online |  |
| **Test accuracy** | There is a 95% chance the test result is accurate | There is a 95% chance the test result is accurate |  |
| **Whether you can also test for other infections such as syphilis and gonorrhoea at the same time** | Yes | No |  |
| **How much you have to pay for the test** | Its free | Its free |  |
| **Which option would you most prefer?**  **(tick one box)** |  |  |  |

**Question 11, version B**

Imagine that yesterday you had condomless anal sex with someone whose HIV status you're unsure of. What are you most likely to do?

|  | **Take an HIV test by yourself** | **Get tested for HIV by a health care professional** | **Not take an HIV test** |
| --- | --- | --- | --- |
| **How long you have to wait before you can take a test** | 4 weeks time | 4 weeks time |  |
| **Where you can take the test** | Somewhere convenient to you, such as your home | At a mobile clinic based at a bar, club or sauna |  |
| **How the test is taken** | A drop of blood from a skin prick | A drop of blood from a skin prick |  |
| **How you get the test** | Order it online | Just drop in and wait your turn |  |
| **How long you have to wait for a test result once you have taken it** | There and then in 10 minutes. | You will receive a call with your result sometime the same day from a health care professional. |  |
| **Who you can talk with about your result** | A free phone number to a health care professional is provided and advice is available online | You can talk with the person who gives you your result and advice is available online |  |
| **Test accuracy** | There is a 99% chance the test result is accurate | There is a 95% chance the test result is accurate |  |
| **Whether you can also test for other infections such as syphilis and gonorrhoea at the same time** | Yes | Yes |  |
| **How much you have to pay for the test** | £10 | Its free |  |
| **Which option would you most prefer?**  **(tick one box)** |  |  |  |

**Question 12, version B**

Imagine that yesterday you had condomless anal sex with someone whose HIV status you're unsure of. What are you most likely to do?

|  | **Take an HIV test by yourself** | **Get tested for HIV by a health care professional** | **Not take an HIV test** |
| --- | --- | --- | --- |
| **How long you have to wait before you can take a test** | 12 weeks time | 4 weeks time |  |
| **Where you can take the test** | Somewhere convenient to you, such as your home | At a mobile clinic based at a bar, club or sauna |  |
| **How the test is taken** | Using an oral swab (which is similar to a cotton bud) | A drop of blood from a skin prick |  |
| **How you get the test** | Order it online | Just drop in and wait your turn |  |
| **How long you have to wait for a test result once you have taken it** | There and then in 30 minutes | There and then in 30 minutes |  |
| **Who you can talk with about your result** | A free phone number to a health care professional is provided and advice is available online | You can talk with the person who gives you your result and advice is available online |  |
| **Test accuracy** | There is a 95% chance the test result is accurate | There is a 95% chance the test result is accurate |  |
| **Whether you can also test for other infections such as syphilis and gonorrhoea at the same time** | No | Yes |  |
| **How much you have to pay for the test** | £30 | Its free |  |
| **Which option would you most prefer?**  **(tick one box)** |  |  |  |

**Question 1, version C**

Imagine that yesterday you had condomless anal sex with someone whose HIV status you're unsure of. What are you most likely to do?

|  | **Take an HIV test by yourself** | **Get tested for HIV by a health care professional** | **Not take an HIV test** |
| --- | --- | --- | --- |
| **How long you have to wait before you can take a test** | 12 weeks time | 4 weeks time |  |
| **Where you can take the test** | Somewhere convenient to you, such as your home | At your GPs |  |
| **How the test is taken** | A drop of blood from a skin prick | A blood sample via a syringe |  |
| **How you get the test** | Order it online | Book and attend an appointment |  |
| **How long you have to wait for a test result once you have taken it** | There and then in 10 minutes | There and then in 30 minutes |  |
| **Who you can talk with about your result** | A free phone number to a health care professional is provided and advice is available online | You can talk with the person who gives you your result and advice is available online |  |
| **Test accuracy** | There is a 95% chance the test result is accurate | There is a 99% chance the test result is accurate |  |
| **Whether you can also test for other infections such as syphilis and gonorrhoea at the same time** | Yes | No |  |
| **How much you have to pay for the test** | £20 | Its free |  |
| **Which option would you most prefer?**  **(tick one box)** |  |  |  |

**Question 2, version C**

Imagine that yesterday you had condomless anal sex with someone whose HIV status you're unsure of. What are you most likely to do?

|  | **Take an HIV test by yourself** | **Get tested for HIV by a health care professional** | **Not take an HIV test** |
| --- | --- | --- | --- |
| **How long you have to wait before you can take a test** | 12 weeks time | 4 weeks time |  |
| **Where you can take the test** | Somewhere convenient to you, such as your home | At a sexual health clinic |  |
| **How the test is taken** | A drop of blood from a skin prick | A drop of blood from a skin prick |  |
| **How you get the test** | Order it online | Book and attend an appointment |  |
| **How long you have to wait for a test result once you have taken it** | You need to put your sample in the post and will receive a call with your result in 3 days from a health care professional. | You will receive a call with your result in 3 days time from a health care professional |  |
| **Who you can talk with about your result** | You can talk with the person who gives you your result and advice is available online | You can talk with the person who gives you your result and advice is available online |  |
| **Test accuracy** | There is a 99% chance the test result is accurate | There is a 99% chance the test result is accurate |  |
| **Whether you can also test for other infections such as syphilis and gonorrhoea at the same time** | Yes | Yes |  |
| **How much you have to pay for the test** | £30 | Its free |  |
| **Which option would you most prefer?**  **(tick one box)** |  |  |  |

**Question 3, version C**

Imagine that yesterday you had condomless anal sex with someone whose HIV status you're unsure of. What are you most likely to do?

|  | **Take an HIV test by yourself** | **Get tested for HIV by a health care professional** | **Not take an HIV test** |
| --- | --- | --- | --- |
| **How long you have to wait before you can take a test** | 4 weeks time | 4 weeks time |  |
| **Where you can take the test** | Somewhere convenient to you, such as your home | At a sexual health clinic |  |
| **How the test is taken** | Using an oral swab (which is similar to a cotton bud) | A drop of blood from a skin prick |  |
| **How you get the test** | Order it online | Just drop in and wait your turn |  |
| **How long you have to wait for a test result once you have taken it** | There and then in 30 minutes | You will receive a call with your result in 3 days time from a health care professional |  |
| **Who you can talk with about your result** | A free phone number to a health care professional is provided and advice is available online | You can talk with the person who gives you your result and advice is available online |  |
| **Test accuracy** | There is a 95% chance the test result is accurate | There is a 95% chance the test result is accurate |  |
| **Whether you can also test for other infections such as syphilis and gonorrhoea at the same time** | Yes | No |  |
| **How much you have to pay for the test** | £20 | Its free |  |
| **Which option would you most prefer?**  **(tick one box)** |  |  |  |

**Question 4, version C**

Imagine that yesterday you had condomless anal sex with someone whose HIV status you're unsure of. What are you most likely to do?

|  | **Take an HIV test by yourself** | **Get tested for HIV by a health care professional** | **Not take an HIV test** |
| --- | --- | --- | --- |
| **How long you have to wait before you can take a test** | 4 weeks time | 4 weeks time |  |
| **Where you can take the test** | Somewhere convenient to you, such as your home | At your GPs |  |
| **How the test is taken** | Using an oral swab (which is similar to a cotton bud) | A drop of blood from a skin prick |  |
| **How you get the test** | Order it online | Book and attend an appointment |  |
| **How long you have to wait for a test result once you have taken it** | You need to put your sample in the post and will receive a call with your result in 3 days from a health care professional | You will receive a call with your result sometime the same day from a health care professional. |  |
| **Who you can talk with about your result** | You can talk with the person who gives you your result and advice is available online | You can talk with the person who gives you your result and advice is available online |  |
| **Test accuracy** | There is a 95% chance the test result is accurate | There is a 99% chance the test result is accurate |  |
| **Whether you can also test for other infections such as syphilis and gonorrhoea at the same time** | No | Yes |  |
| **How much you have to pay for the test** | £10 | Its free |  |
| **Which option would you most prefer?**  **(tick one box)** |  |  |  |

**Question 5, version C**

Imagine that yesterday you had condomless anal sex with someone whose HIV status you're unsure of. What are you most likely to do?

|  | **Take an HIV test by yourself** | **Get tested for HIV by a health care professional** | **Not take an HIV test** |
| --- | --- | --- | --- |
| **How long you have to wait before you can take a test** | 12 weeks time | 4 weeks time |  |
| **Where you can take the test** | Somewhere convenient to you, such as your home | At a community location such as an HIV charity |  |
| **How the test is taken** | A drop of blood from a skin prick | A drop of blood from a skin prick |  |
| **How you get the test** | 'Click and collect' from a pharmacy or health clinic | Just drop in and wait your turn |  |
| **How long you have to wait for a test result once you have taken it** | There and then in 30 minutes | You will receive a call with your result sometime the same day from a health care professional |  |
| **Who you can talk with about your result** | A free phone number to a health care professional is provided and advice is available online | You can talk with the person who gives you your result and advice is available online |  |
| **Test accuracy** | There is a 99% chance the test result is accurate | There is a 99% chance the test result is accurate |  |
| **Whether you can also test for other infections such as syphilis and gonorrhoea at the same time** | No | No |  |
| **How much you have to pay for the test** | £10 | Its free |  |
| **Which option would you most prefer?**  **(tick one box)** |  |  |  |

**Question 6, version C**

Imagine that yesterday you had condomless anal sex with someone whose HIV status you're unsure of. What are you most likely to do?

|  | **Take an HIV test by yourself** | **Get tested for HIV by a health care professional** | **Not take an HIV test** |
| --- | --- | --- | --- |
| **How long you have to wait before you can take a test** | 12 weeks time | 4 weeks time |  |
| **Where you can take the test** | Somewhere convenient to you, such as your home | At your GPs |  |
| **How the test is taken** | A drop of blood from a skin prick | A drop of blood from a skin prick |  |
| **How you get the test** | Order it online | Book and attend an appointment |  |
| **How long you have to wait for a test result once you have taken it** | There and then in 30 minutes | There and then in 10 minutes |  |
| **Who you can talk with about your result** | A free phone number to a health care professional is provided and advice is available online | You can talk with the person who gives you your result and advice is available online |  |
| **Test accuracy** | There is a 95% chance the test result is accurate | There is a 95% chance the test result is accurate |  |
| **Whether you can also test for other infections such as syphilis and gonorrhoea at the same time** | Yes | Yes |  |
| **How much you have to pay for the test** | Its free | Its free |  |
| **Which option would you most prefer?**  **(tick one box)** |  |  |  |

**Question 7, version C**

Imagine that yesterday you had condomless anal sex with someone whose HIV status you're unsure of. What are you most likely to do?

|  | **Take an HIV test by yourself** | **Get tested for HIV by a health care professional** | **Not take an HIV test** |
| --- | --- | --- | --- |
| **How long you have to wait before you can take a test** | 4 weeks time | 4 weeks time |  |
| **Where you can take the test** | Somewhere convenient to you, such as your home | At your GPs |  |
| **How the test is taken** | Using an oral swab (which is similar to a cotton bud) | A blood sample via a syringe |  |
| **How you get the test** | 'Click and collect' from a pharmacy or health clinic | Just drop in and wait your turn |  |
| **How long you have to wait for a test result once you have taken it** | There and then in 30 minutes | You will receive a call with your result sometime the same day from a health care professional |  |
| **Who you can talk with about your result** | A free phone number to a health care professional is provided and advice is available online | You can talk with the person who gives you your result and advice is available online |  |
| **Test accuracy** | There is a 99% chance the test result is accurate | There is a 99% chance the test result is accurate |  |
| **Whether you can also test for other infections such as syphilis and gonorrhoea at the same time** | Yes | Yes |  |
| **How much you have to pay for the test** | £30 | Its free |  |
| **Which option would you most prefer?**  **(tick one box)** |  |  |  |

**Question 8, version C**

Imagine that yesterday you had condomless anal sex with someone whose HIV status you're unsure of. What are you most likely to do?

|  | **Take an HIV test by yourself** | **Get tested for HIV by a health care professional** | **Not take an HIV test** |
| --- | --- | --- | --- |
| **How long you have to wait before you can take a test** | 12 weeks time | 4 weeks time |  |
| **Where you can take the test** | Somewhere convenient to you, such as your home | At your GPs |  |
| **How the test is taken** | A drop of blood from a skin prick | A drop of blood from a skin prick |  |
| **How you get the test** | 'Click and collect' from a pharmacy or health clinic | Just drop in and wait your turn |  |
| **How long you have to wait for a test result once you have taken it** | You need to put your sample in the post and will receive a call with your result in 3 days from a health care professional. | There and then in 30 minutes. |  |
| **Who you can talk with about your result** | You can talk with the person who gives you your result and advice is available online | You can talk with the person who gives you your result and advice is available online |  |
| **Test accuracy** | There is a 99% chance the test result is accurate | There is a 95% chance the test result is accurate |  |
| **Whether you can also test for other infections such as syphilis and gonorrhoea at the same time** | No | No |  |
| **How much you have to pay for the test** | Its free | Its free |  |
| **Which option would you most prefer?**  **(tick one box)** |  |  |  |

**Question 9, version C**

Imagine that yesterday you had condomless anal sex with someone whose HIV status you're unsure of. What are you most likely to do?

|  | **Take an HIV test by yourself** | **Get tested for HIV by a health care professional** | **Not take an HIV test** |
| --- | --- | --- | --- |
| **How long you have to wait before you can take a test** | 4 weeks time | 4 weeks time |  |
| **Where you can take the test** | Somewhere convenient to you, such as your home | At a mobile clinic based at a bar, club or sauna |  |
| **How the test is taken** | Using an oral swab (which is similar to a cotton bud) | A drop of blood from a skin prick |  |
| **How you get the test** | 'Click and collect' from a pharmacy or health clinic | Just drop in and wait your turn |  |
| **How long you have to wait for a test result once you have taken it** | There and then in 10 minutes | You will receive a call with your result in 3 days time from a health care professional |  |
| **Who you can talk with about your result** | A free phone number to a health care professional is provided and advice is available online | You can talk with the person who gives you your result and advice is available online |  |
| **Test accuracy** | There is a 99% chance the test result is accurate | There is a 99% chance the test result is accurate |  |
| **Whether you can also test for other infections such as syphilis and gonorrhoea at the same time** | No | No |  |
| **How much you have to pay for the test** | Its free | Its free |  |
| **Which option would you most prefer?**  **(tick one box)** |  |  |  |

**Question 10, version C**

Imagine that yesterday you had condomless anal sex with someone whose HIV status you're unsure of. What are you most likely to do?

|  | **Take an HIV test by yourself** | **Get tested for HIV by a health care professional** | **Not take an HIV test** |
| --- | --- | --- | --- |
| **How long you have to wait before you can take a test** | 4 weeks time | 4 weeks time |  |
| **Where you can take the test** | Somewhere convenient to you, such as your home | At a community location such as an HIV charity |  |
| **How the test is taken** | A drop of blood from a skin prick | A drop of blood from a skin prick |  |
| **How you get the test** | 'Click and collect' from a pharmacy or health clinic | Just drop in and wait your turn |  |
| **How long you have to wait for a test result once you have taken it** | There and then in 10 minutes | There and then in 10 minutes |  |
| **Who you can talk with about your result** | A free phone number to a health care professional is provided and advice is available online | You can talk with the person who gives you your results and advice is available online |  |
| **Test accuracy** | There is a 95% chance the test result is accurate | There is a 95% chance the test result is accurate |  |
| **Whether you can also test for other infections such as syphilis and gonorrhoea at the same time** | No | No |  |
| **How much you have to pay for the test** | £30 | Its free |  |
| **Which option would you most prefer?**  **(tick one box)** |  |  |  |

**Question 11, version C**

Imagine that yesterday you had condomless anal sex with someone whose HIV status you're unsure of. What are you most likely to do?

|  | **Take an HIV test by yourself** | **Get tested for HIV by a health care professional** | **Not take an HIV test** |
| --- | --- | --- | --- |
| **How long you have to wait before you can take a test** | 12 weeks time | 4 weeks time |  |
| **Where you can take the test** | Somewhere convenient to you, such as your home | At your GPs |  |
| **How the test is taken** | Using an oral swab (which is similar to a cotton bud) | A drop of blood from a skin prick |  |
| **How you get the test** | Order it online | Just drop in and wait your turn |  |
| **How long you have to wait for a test result once you have taken it** | There and then in 10 minutes | You will receive a call with your result in 3 days time from a health care professional |  |
| **Who you can talk with about your result** | A free phone number to a health care professional is provided and advice is available online | You can talk with the person who gives you your result and advice is available online |  |
| **Test accuracy** | There is a 99% chance the test result is accurate | There is a 95% chance the test result is accurate |  |
| **Whether you can also test for other infections such as syphilis and gonorrhoea at the same time** | No | Yes |  |
| **How much you have to pay for the test** | £10 | Its free |  |
| **Which option would you most prefer?**  **(tick one box)** |  |  |  |

**Question 12, version C**

Imagine that yesterday you had condomless anal sex with someone whose HIV status you're unsure of. What are you most likely to do?

|  | **Take an HIV test by yourself** | **Get tested for HIV by a health care professional** | **Not take an HIV test** |
| --- | --- | --- | --- |
| **How long you have to wait before you can take a test** | 4 weeks time | 4 weeks time |  |
| **Where you can take the test** | Somewhere convenient to you, such as your home | At a sexual health clinic |  |
| **How the test is taken** | Using an oral swab (which is similar to a cotton bud) | A blood sample via a syringe |  |
| **How you get the test** | 'Click and collect' from a pharmacy or health clinic | Book and attend an appointment |  |
| **How long you have to wait for a test result once you have taken it** | You need to put your sample in the post and will receive a call with your result in 3 days from a health care professional | There and then in 30 minutes |  |
| **Who you can talk with about your result** | You can talk with the person who gives you your result and advice is available online | You can talk with the person who gives you your result and advice is available online |  |
| **Test accuracy** | There is a 95% chance the test result is accurate | There is a 95% chance the test result is accurate |  |
| **Whether you can also test for other infections such as syphilis and gonorrhoea at the same time** | Yes | Yes |  |
| **How much you have to pay for the test** | £10 | Its free |  |
| **Which option would you most prefer?**  **(tick one box)** |  |  |  |

**Question 1, version D**

Imagine that yesterday you had condomless anal sex with someone whose HIV status you're unsure of. What are you most likely to do?

|  | **Take an HIV test by yourself** | **Get tested for HIV by a health care professional** | **Not take an HIV test** |
| --- | --- | --- | --- |
| **How long you have to wait before you can take a test** | 12 weeks time | 4 weeks time |  |
| **Where you can take the test** | Somewhere convenient to you, such as your home | At a mobile clinic based at a bar, club or sauna |  |
| **How the test is taken** | Using an oral swab (which is similar to a cotton bud) | A drop of blood from a skin prick |  |
| **How you get the test** | Order it online | Just drop in and wait your turn |  |
| **How long you have to wait for a test result once you have taken it** | There and then in 30 minutes | There and then in 30 minutes |  |
| **Who you can talk with about your result** | A free phone number to a health care professional is provided and advice is available online | You can talk with the person who gives you your result and advice is available online |  |
| **Test accuracy** | There is a 95% chance the test result is accurate | There is a 95% chance the test result is accurate |  |
| **Whether you can also test for other infections such as syphilis and gonorrhoea at the same time** | No | Yes |  |
| **How much you have to pay for the test** | £30 | Its free |  |
| **Which option would you most prefer?**  **(tick one box)** |  |  |  |

**Question 2, version D**

Imagine that yesterday you had condomless anal sex with someone whose HIV status you're unsure of. What are you most likely to do?

|  | **Take an HIV test by yourself** | **Get tested for HIV by a health care professional** | **Not take an HIV test** |
| --- | --- | --- | --- |
| **How long you have to wait before you can take a test** | 4 weeks time | 4 weeks time |  |
| **Where you can take the test** | Somewhere convenient to you, such as your home | At a mobile clinic based at a bar, club or sauna |  |
| **How the test is taken** | A drop of blood from a skin prick | A drop of blood from a skin prick |  |
| **How you get the test** | Order it online | Just drop in and wait your turn |  |
| **How long you have to wait for a test result once you have taken it** | There and then in 10 minutes. | You will receive a call with your result sometime the same day from a health care professional. |  |
| **Who you can talk with about your result** | A free phone number to a health care professional is provided and advice is available online | You can talk with the person who gives you your result and advice is available online |  |
| **Test accuracy** | There is a 99% chance the test result is accurate | There is a 95% chance the test result is accurate |  |
| **Whether you can also test for other infections such as syphilis and gonorrhoea at the same time** | Yes | Yes |  |
| **How much you have to pay for the test** | £10 | Its free |  |
| **Which option would you most prefer?**  **(tick one box)** |  |  |  |

**Question 3, version D**

Imagine that yesterday you had condomless anal sex with someone whose HIV status you're unsure of. What are you most likely to do?

|  | **Take an HIV test by yourself** | **Get tested for HIV by a health care professional** | **Not take an HIV test** |
| --- | --- | --- | --- |
| **How long you have to wait before you can take a test** | 12 weeks time | 4 weeks time |  |
| **Where you can take the test** | Somewhere convenient to you, such as your home | At a sexual health clinic |  |
| **How the test is taken** | Using an oral swab (which is similar to a cotton bud) | A drop of blood from a skin prick |  |
| **How you get the test** | 'Click and collect' from a pharmacy or health clinic | Book and attend an appointment |  |
| **How long you have to wait for a test result once you have taken it** | There and then in 10 minutes | You will receive a call with your result sometime the same day from a health care professional |  |
| **Who you can talk with about your result** | A free phone number to a health care professional is provided and advice is available online | You can talk with the person who gives you your result and advice is available online |  |
| **Test accuracy** | There is a 95% chance the test result is accurate | There is a 95% chance the test result is accurate |  |
| **Whether you can also test for other infections such as syphilis and gonorrhoea at the same time** | Yes | No |  |
| **How much you have to pay for the test** | Its free | Its free |  |
| **Which option would you most prefer?**  **(tick one box)** |  |  |  |

**Question 4, version D**

Imagine that yesterday you had condomless anal sex with someone whose HIV status you're unsure of. What are you most likely to do?

|  | **Take an HIV test by yourself** | **Get tested for HIV by a health care professional** | **Not take an HIV test** |
| --- | --- | --- | --- |
| **How long you have to wait before you can take a test** | 4 weeks time | 4 weeks time |  |
| **Where you can take the test** | Somewhere convenient to you, such as your home | At your GPs |  |
| **How the test is taken** | A drop of blood from a skin prick | A drop of blood from a skin prick |  |
| **How you get the test** | 'Click and collect' from a pharmacy or health clinic | Book and attend an appointment |  |
| **How long you have to wait for a test result once you have taken it** | You need to put your sample in the post and will receive a call with your result in 7 days from a health care professional | You will receive a call with your result in 3 days time from a health care professional |  |
| **Who you can talk with about your result** | You can talk with the person who gives you your result and advice is available online | You can talk with the person who gives you your result and advice is available online |  |
| **Test accuracy** | There is a 95% chance the test result is accurate | There is a 99% chance the test result is accurate |  |
| **Whether you can also test for other infections such as syphilis and gonorrhoea at the same time** | Yes | No |  |
| **How much you have to pay for the test** | £30 | Its free |  |
| **Which option would you most prefer?**  **(tick one box)** |  |  |  |

**Question 5, version D**

Imagine that yesterday you had condomless anal sex with someone whose HIV status you're unsure of. What are you most likely to do?

|  | **Take an HIV test by yourself** | **Get tested for HIV by a health care professional** | **Not take an HIV test** |
| --- | --- | --- | --- |
| **How long you have to wait before you can take a test** | 4 weeks time | 4 weeks time |  |
| **Where you can take the test** | Somewhere convenient to you, such as your home | At a sexual health clinic |  |
| **How the test is taken** | A drop of blood from a skin prick | A blood sample via a syringe |  |
| **How you get the test** | Order it online | Just drop in and wait your turn |  |
| **How long you have to wait for a test result once you have taken it** | You need to put your sample in the post and will receive a call with your result in 3 days from a health care professional | You will receive a call with your result sometime the same day from a health care professional |  |
| **Who you can talk with about your result** | You can talk with the person who gives you your result and advice is available online | You can talk with the person who gives you your result and advice is available online |  |
| **Test accuracy** | There is a 95% chance the test result is accurate | There is a 99% chance the test result is accurate |  |
| **Whether you can also test for other infections such as syphilis and gonorrhoea at the same time** | No | Yes |  |
| **How much you have to pay for the test** | Its free | Its free |  |
| **Which option would you most prefer?**  **(tick one box)** |  |  |  |

**Question 6, version D**

Imagine that yesterday you had condomless anal sex with someone whose HIV status you're unsure of. What are you most likely to do?

|  | **Take an HIV test by yourself** | **Get tested for HIV by a health care professional** | **Not take an HIV test** |
| --- | --- | --- | --- |
| **How long you have to wait before you can take a test** | 4 weeks time | 4 weeks time |  |
| **Where you can take the test** | Somewhere convenient to you, such as your home | At a community location such as an HIV charity |  |
| **How the test is taken** | Using an oral swab (which is similar to a cotton bud) | A drop of blood from a skin prick |  |
| **How you get the test** | Order it online | Just drop in and wait your turn |  |
| **How long you have to wait for a test result once you have taken it** | You need to put your sample in the post and will receive a call with your result in 7 days from a health care professional | There and then in 30 minutes |  |
| **Who you can talk with about your result** | You can talk with the person who gives you your result and advice is available online | You can talk with the person who gives you your result and advice is available online |  |
| **Test accuracy** | There is a 99% chance the test result is accurate | There is a 99% chance the test result is accurate |  |
| **Whether you can also test for other infections such as syphilis and gonorrhoea at the same time** | Yes | Yes |  |
| **How much you have to pay for the test** | Its free | Its free |  |
| **Which option would you most prefer?**  **(tick one box)** |  |  |  |

**Question 7, version D**

Imagine that yesterday you had condomless anal sex with someone whose HIV status you're unsure of. What are you most likely to do?

|  | **Take an HIV test by yourself** | **Get tested for HIV by a health care professional** | **Not take an HIV test** |
| --- | --- | --- | --- |
| **How long you have to wait before you can take a test** | 12 weeks time | 4 weeks time |  |
| **Where you can take the test** | Somewhere convenient to you, such as your home | At a sexual health clinic |  |
| **How the test is taken** | Using an oral swab (which is similar to a cotton bud) | A blood sample via a syringe |  |
| **How you get the test** | Order it online | Book and attend an appointment |  |
| **How long you have to wait for a test result once you have taken it** | You need to put your sample in the post and will receive a call with your result in 7 days from a health care professional | You will receive a call with your result sometime the same day from a health care professional |  |
| **Who you can talk with about your result** | You can talk with the person who gives you your result and advice is available online | You can talk with the person who gives you your result and advice is available online |  |
| **Test accuracy** | There is a 99% chance the test result is accurate | There is a 95% chance the test result is accurate |  |
| **Whether you can also test for other infections such as syphilis and gonorrhoea at the same time** | No | No |  |
| **How much you have to pay for the test** | £30 | Its free |  |
| **Which option would you most prefer?**  **(tick one box)** |  |  |  |

**Question 8, version D**

Imagine that yesterday you had condomless anal sex with someone whose HIV status you're unsure of. What are you most likely to do?

|  | **Take an HIV test by yourself** | **Get tested for HIV by a health care professional** | **Not take an HIV test** |
| --- | --- | --- | --- |
| **How long you have to wait before you can take a test** | 4 weeks time | 4 weeks time |  |
| **Where you can take the test** | Somewhere convenient to you, such as your home | At a sexual health clinic |  |
| **How the test is taken** | A drop of blood from a skin prick | A drop of blood from a skin prick |  |
| **How you get the test** | 'Click and collect' from a pharmacy or health clinic | Book and attend an appointment |  |
| **How long you have to wait for a test result once you have taken it** | There and then in 30 minutes | There and then in 10 minutes |  |
| **Who you can talk with about your result** | A free phone number to a health care professional is provided and advice is available online | You can talk with the person who gives you your result and advice is available online |  |
| **Test accuracy** | There is a 99% chance the test result is accurate | There is a 99% chance the test result is accurate |  |
| **Whether you can also test for other infections such as syphilis and gonorrhoea at the same time** | No | Yes |  |
| **How much you have to pay for the test** | £20 | Its free |  |
| **Which option would you most prefer?**  **(tick one box)** |  |  |  |

**Question 9, version D**

Imagine that yesterday you had condomless anal sex with someone whose HIV status you're unsure of. What are you most likely to do?

|  | **Take an HIV test by yourself** | **Get tested for HIV by a health care professional** | **Not take an HIV test** |
| --- | --- | --- | --- |
| **How long you have to wait before you can take a test** | 12 weeks time | 4 weeks time |  |
| **Where you can take the test** | Somewhere convenient to you, such as your home | At a mobile clinic based at a bar, club or sauna |  |
| **How the test is taken** | Using an oral swab (which is similar to a cotton bud) | A drop of blood from a skin prick |  |
| **How you get the test** | 'Click and collect' from a pharmacy or health clinic | Just drop in and wait your turn |  |
| **How long you have to wait for a test result once you have taken it** | You need to put your sample in the post and will receive a call with your result in 3 days from a health care professional | There and then in 10 minutes. |  |
| **Who you can talk with about your result** | . You can talk with the person who gives you your result and advice is available online | You can talk with the person who gives you your result and advice is available online |  |
| **Test accuracy** | There is a 99% chance the test result is accurate | There is a 99% chance the test result is accurate |  |
| **Whether you can also test for other infections such as syphilis and gonorrhoea at the same time** | Yes | No |  |
| **How much you have to pay for the test** | £20 | Its free |  |
| **Which option would you most prefer?**  **(tick one box)** |  |  |  |

**Question 10, version D**

Imagine that yesterday you had condomless anal sex with someone whose HIV status you're unsure of. What are you most likely to do?

|  | **Take an HIV test by yourself** | **Get tested for HIV by a health care professional** | **Not take an HIV test** |
| --- | --- | --- | --- |
| **How long you have to wait before you can take a test** | 4 weeks time | 4 weeks time |  |
| **Where you can take the test** | Somewhere convenient to you, such as your home | At your GPs |  |
| **How the test is taken** | A drop of blood from a skin prick | A blood sample via a syringe |  |
| **How you get the test** | Order it online | Book and attend an appointment |  |
| **How long you have to wait for a test result once you have taken it** | You need to put your sample in the post and will receive a call with your result in 7 days from a health care professional | You will receive a call with your result sometime the same day from a health care professional |  |
| **Who you can talk with about your result** | You can talk with the person who gives you your result and advice is available online | You can talk with the person who gives you your result and advice is available online |  |
| **Test accuracy** | There is a 99% chance the test result is accurate | There is a 95% chance the test result is accurate |  |
| **Whether you can also test for other infections such as syphilis and gonorrhoea at the same time** | No | No |  |
| **How much you have to pay for the test** | £20 | Its free |  |
| **Which option would you most prefer?**  **(tick one box)** |  |  |  |

**Question 11, version D**

Imagine that yesterday you had condomless anal sex with someone whose HIV status you're unsure of. What are you most likely to do?

|  | **Take an HIV test by yourself** | **Get tested for HIV by a health care professional** | **Not take an HIV test** |
| --- | --- | --- | --- |
| **How long you have to wait before you can take a test** | 12 weeks time | 4 weeks time |  |
| **Where you can take the test** | Somewhere convenient to you, such as your home | At a sexual health clinic |  |
| **How the test is taken** | A drop of blood from a skin prick | A drop of blood from a skin prick |  |
| **How you get the test** | 'Click and collect' from a pharmacy or health clinic | Just drop in and wait your turn |  |
| **How long you have to wait for a test result once you have taken it** | You need to put your sample in the post and will receive a call with your result in 7 days from a health care professional | There and then in 10 minutes |  |
| **Who you can talk with about your result** | You can talk with the person who gives you your result and advice is available online | You can talk with the person who gives you your result and advice is available online |  |
| **Test accuracy** | There is a 95% chance the test result is accurate | There is a 99% chance the test result is accurate |  |
| **Whether you can also test for other infections such as syphilis and gonorrhoea at the same time** | Yes | No |  |
| **How much you have to pay for the test** | £10 | Its free |  |
| **Which option would you most prefer?**  **(tick one box)** |  |  |  |

**Question 12, version D**

Imagine that yesterday you had condomless anal sex with someone whose HIV status you're unsure of. What are you most likely to do?

|  | **Take an HIV test by yourself** | **Get tested for HIV by a health care professional** | **Not take an HIV test** |
| --- | --- | --- | --- |
| **How long you have to wait before you can take a test** | 12 weeks time | 4 weeks time |  |
| **Where you can take the test** | Somewhere convenient to you, such as your home | At a community location such as an HIV charity |  |
| **How the test is taken** | Using an oral swab (which is similar to a cotton bud) | A drop of blood from a skin prick |  |
| **How you get the test** | 'Click and collect' from a pharmacy or health clinic | Just drop in and wait your turn |  |
| **How long you have to wait for a test result once you have taken it** | You need to put your sample in the post and will receive a call with your result in 7 days from a health care professional | You will receive a call with your result in 3 days time from a health care professional |  |
| **Who you can talk with about your result** | You can talk with the person who gives you your result and advice is available online | You can talk with the person who gives you your result and advice is available online |  |
| **Test accuracy** | There is a 95% chance the test result is accurate | There is a 95% chance the test result is accurate |  |
| **Whether you can also test for other infections such as syphilis and gonorrhoea at the same time** | No | Yes |  |
| **How much you have to pay for the test** | £20 | Its free |  |
| **Which option would you most prefer?**  **(tick one box)** |  |  |  |
